# Global Burden of Thyroid Cancer From 1990 to 2017

**DOI:** 10.1001/jamanetworkopen.2020.8759

**Published:** 2020-06-26

**Authors:** YuJiao Deng, HongTao Li, Meng Wang, Na Li, Tian Tian, Ying Wu, Peng Xu, Si Yang, Zhen Zhai, LingHui Zhou, Qian Hao, DingLi Song, TianBo Jin, Jun Lyu, ZhiJun Dai

**Affiliations:** 1 Department of Breast Surgery, The First Affiliated Hospital, College of Medicine, Zhejiang University, Hangzhou, China; 2 Department of Oncology, The Second Affiliated Hospital of Xi’an Jiaotong University, Xi’an, China; 3 Department of Breast, Head and Neck Surgery, The Third Affiliated Teaching Hospital of Xinjiang Medical University (Affiliated Tumor Hospital), Urumqi, China; 4 Key Laboratory of Resource Biology and Biotechnology in Western China, Ministry of Education, School of Life Sciences, Northwest University, Xi’an, China; 5 Department of Clinical Research, The First Affiliated Hospital of Jinan University, Guangzhou, China

## Abstract

**Importance:**

Thyroid cancer is the most pervasive endocrine cancer worldwide. Studies examining the association between thyroid cancer and country, sex, age, sociodemographic index (SDI), and other factors are lacking.

**Objective:**

To examine the thyroid cancer burden and variation trends at the global, regional, and national levels using data on sex, age, and SDI.

**Design, Setting, and Participants:**

In this cross-sectional study, epidemiologic data were gathered using the Global Health Data Exchange query tool, covering persons of all ages with thyroid cancer in 195 countries and 21 regions from January 1, 1990, to December 31, 2017; data analysis was completed on October 1, 2019. All participants met the Global Burden of Disease Study inclusion criteria.

**Main Outcomes and Measures:**

Outcomes included incidence, deaths, and disability-adjusted life-years (DALYs) of thyroid cancer. Measures were stratified by sex, region, country, age, and SDI. The estimated annual percentage changes (EAPCs) and age-standardized rates were calculated to evaluate the temporal trends.

**Results:**

Increases of thyroid cancer were noted in incident cases (169%), deaths (87%), and DALYs (75%). Age-standardized incidence rate (ASIR) showed an upward trend over time, with an EAPC of 1.59 (95% CI, 1.51-1.67); decreases were noted in EAPCs of age-standardized death rate (−0.15; 95% CI, −0.19 to −0.12) and age-standardized DALY rate (−0.11; 95% CI, −0.15 to −0.08). Almost half (41.73% for incidence, 50.92% for deaths, and 54.39% for DALYs) of the thyroid cancer burden was noted in Southern and Eastern Asia. In addition, females accounted for most of the thyroid cancer burden (70.22% for incidence, 58.39% for deaths, and 58.68% for DALYs) and increased by years in this population, although the ASIR of males with thyroid cancer (EAPC, 2.18; 95% CI, 2.07-2.28) increased faster than that of females (EAPC, 1.38; 95% CI, 1.30-1.46). A third (34%) of patients with thyroid cancer resided in countries with a high SDI, and most patients were aged 50 to 69 years, which was older than the age in other quintiles (high SDI quintile compared with all other quintiles, *P*<.05). The most common age at onset of thyroid cancer worldwide was 15 to 49 years in female individuals compared with 50 to 69 years in male individuals (*P*<.05). Death from thyroid cancer was concentrated in participants aged 70 years or older and increased by years (average annual percentage change, 0.10; 95% CI, 0.01-0.21; *P*<.05). Furthermore, people in lower SDI quintiles developed thyroid cancer and died from it earlier than those in other quintiles (high and high-middle SDI vs low and low-middle SDI, *P*<.05).

**Conclusions and Relevance:**

Data from this study suggest considerable heterogeneity in the epidemiologic patterns of thyroid cancer across sex, age, SDI, region, and country, providing information for governments that may help improve national and local cancer control policies.

## Key Points

**Question:**

What were the epidemiologic patterns and variation in the trends of thyroid cancer worldwide from 1990 to 2017?

**Findings:**

In this cross-sectional study covering data on incidence, deaths, and disability-adjusted life-years and their temporal trends from 195 countries and 21 regions, increasing trends of thyroid cancer burden were observed, with significant differences by sex, region, country, age, and sociodemographic index. Almost half of the thyroid cancer burden was noted in Southern and Eastern Asia, and a third of patients with thyroid cancer resided in countries with a high sociodemographic index.

**Meaning:**

This study suggests an increasing global burden of thyroid cancer; the geographic disparities may provide support for cancer health care planning and resource allocation.

## Teaser

This cross-sectional study examines the worldwide temporal trends of thyroid cancer according to geographic location, sex, age, and socioeconomic index.

## Introduction

Thyroid cancer is the most pervasive endocrine cancer worldwide.^1^ During the past decades, published studies reported that the incidence of thyroid cancer continues to increase^2^ in countries and regions such as Canada,^3^ the US,^4^ Australia,^5^ Asia,^6,7,8^ South America,^9^ and Europe.^10,11,12,13^ Although some regional studies have provided data on the incidence and mortality associated with thyroid cancer,^14,15^ studies on thyroid cancer examining the association between the disease and country, sex, age, sociodemographic index (SDI), and other factors are lacking. Comprehensive, in-depth analysis of thyroid cancer in all regions of the world based on a variety of factors may be beneficial for health care planning and resource allocation.

The Global Health Data Exchange is a public website available for querying the burden of 354 human diseases and injuries in 195 countries and territories worldwide, providing an opportunity to investigate the distribution and changes in the patterns of thyroid cancer.^16^ Analyses based on age-standardized rates may help policy makers assess the burden of thyroid cancer, measure the progress of specific treatments, allocate resources, and formulate relevant policies. This study aimed to explore the current pattern and alteration of thyroid cancer incidence, deaths, and disability-adjusted life-years (DALYs).

## Methods

### Study Population and Data Collection

The data were obtained using the Global Health Data Exchange, covering annual incidence, deaths, DALYs, and age-standardized rate of thyroid cancer in 21 regions and 195 countries, from January 1, 1990, to December 31, 2017.^17^ Data on both sexes of 4 age groups (5-14, 15-49, 50-69, and ≥70 years) were collected. Detailed descriptions of the methods are presented in the eAppendix in the Supplement. Data analysis was completed on October 1, 2019. To consider the association between development status and thyroid cancer burden, the SDI of each country was calculated in the Global Burden of Disease Study (GBD) 2017.^18^ Sociodemographic index is a comprehensive measurement of educational level, income per capita, and fertility rate, with scores ranging from 0 to 1. Countries were divided into 5 SDI quintiles (high, high-middle, middle, low-middle, and low). We analyzed the thyroid cancer data at the global, regional, and national levels among different SDI, sex, and age groups.

The institutional review board of the First Affiliated Hospital of Zhejiang University in Zhejiang Province, Hangzhou, China, determined that the study did not need approval because it used publicly available data. This study followed the Guidelines for Accurate and Transparent Health Estimates Reporting (GATHER) reporting guideline for cross-sectional studies.^19^

### Statistical Analysis

The age-standardized rates and their estimated annual percentage changes (EAPCs) were calculated to assess the incidence and mortality trends of thyroid cancer using linear regression analysis, and Pearson product-moment correlation analysis was performed to assess correlation. All rates are reported per 100 000 person-years.

The trends of age-standardized rates were reflected in EAPC values: age-standardized rate is in an upward trend when the EAPCs and the lower boundary of the 95% CI are positive; conversely, age-standardized rate is in a downward trend when EAPCs and the upper boundary of the 95% CI are negative. DisMod-MR, version, 2.1, a bayesian meta-regression framework, was used in modeling epidemiologic outcomes and ascertaining the burden of thyroid cancer in different regions, sexes, countries, and age groups. Moreover, the correlation between EAPCs and age-standardized rate in 1990 as well as SDI in 2017 in different countries was evaluated with Pearson correlation analyses to define the potential factors affecting EAPCs. Statistical analyses were performed using R, version 3.5.2 (R Project for Statistical Computing). A 2-tailed *P* value <.05 was considered statistically significant.

## Results

### Thyroid Cancer Worldwide

**Figure 1.**
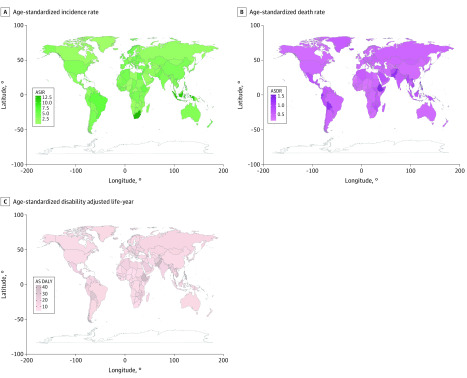
Age-Standardized Rates (per 100 000 Person-Years) of Thyroid Cancer Worldwide Age-standardized incidence rate (ASIR) (A), age-standardized death rate (ASDR) (B), and age-standardized disability-adjusted life-year rate (AS DALY) (C).

**Figure 2.**
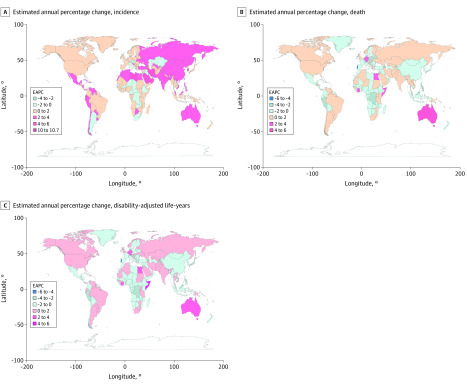
The Estimated Annual Percentage Changes (EAPCs) of Thyroid Cancer Age-Standardized Rates Worldwide Changes shown in incidence (A), death (B), and disability-adjusted life-years (C).

Globally, there were 95 030 incident cases of thyroid cancer (95% uncertainty interval [UI], 90 070-100 720 cases) and 22 070 deaths (95% UI, 20 810-24 220 deaths) in 1990 and 255 490 incident cases (95% UI, 245 710-272 470 cases) and 41 240 deaths (95% UI, 39 910-44 140 deaths) in 2017 (Table). In total, thyroid cancer was responsible for 1 133 170 DALYs (95% UI, 1 073 440-1 277 490 DALYs) in 2017. From 1990 to 2017, increases were noted in incident cases (169%), deaths (87%), and DALYs (75%) of thyroid cancer. In addition, the age-standardized rates (Figure 1) and their changing trends varied among different countries (Figure 2). The age-standardized incidence rate (ASIR) showed an upward trend worldwide (EAPC, 1.59; 95% CI, 1.51-1.67), while age-standardized death rate (ASDR) (EAPC, −0.15; 95% CI, −0.19 to −0.12) and age-standardized DALY rate (EAPC, −0.11; 95% CI, −0.15 to −0.08) presented a downward trend (eTable 1 and eTable 2 in the Supplement). In addition, the ASIR for both sexes (males: EAPC, 2.18; 95% CI, 2.07-2.28; females: EAPC, 1.38; 95% CI, 1.30-1.46) as well as ASDR (EAPC, 0.70; 95% CI, 0.61-0.78) and age-standardized DALY rate (EAPC, 0.59; 95% CI, 0.52-0.66) for males continued to increase, whereas the ASDR (EAPC, −0.63; 95% CI, −0.66 to −0.59) and age-standardized DALY rate (EAPC, −0.53; 95% CI, −0.57 to −0.48) for females showed a decreasing trend.

**Table.** Incidence of Thyroid Cancer and Trends From 1990 to 2017

Characteristics	1990				2017				1990-2017
	Incident cases		ASIR (per 100 000)		Incident cases		ASIR (per 100 000)		EAPC
	No. (95% UI)	Female/male ratio	No. (95% UI)	Female/male ratio	No. (95% UI)	Female/male ratio	No. (95% UI)	Female/male ratio	No. (95% CI)
Overall	95 030 (90 070-100 720)	1.92	2.11 (2.01-2.24)	2.65	255 490 (245 710-272 470)	2.36	3.15 (3.03-3.36)	2.23	1.59 (1.51-1.67)
Sex									
Male	24 170 (23 330-25 210)	NA	1.15 (1.11-1.19)	NA	76 090 (72 580-79 290)	NA	1.94 (1.86-2.02)	NA	2.18 (2.07-2.28)
Female	70 850 (66 090-76 420)	NA	3.04 (2.85-3.28)	NA	179 400 (170 400-195 540)	NA	4.34 (4.12-4.73)	NA	1.38 (1.30-1.46)
Sociodemographic index									
Low	5710 (3970-7390)	1.84	1.23 (0.90-1.58)	2.97	15 400 (13 730-17 480)	3.21	1.63 (1.46-1.85)	2.79	1.01 (0.88-1.13)
Low-middle	9650 (8290-11 620)	2.01	1.28 (1.12-1.54)	3.36	31 540 (28 460-36 750)	3.58	2.14 (1.95-2.47)	3.13	1.91 (1.84-1.98)
Middle	15 470 (14 500-17 770)	1.90	1.31 (1.24-1.52)	2.98	60 930 (57 100-69 460)	2.46	2.61 (2.45-2.97)	2.33	2.65 (2.54-2.75)
High-middle	20 990 (19 080-22 130)	1.94	2.03 (1.87-2.14)	3.05	58 680 (55 410-62 290)	2.38	3.30 (3.12-3.50)	2.28	1.81 (1.64-1.98)
High	42 950 (42 080-43 830)	1.92	3.66 (3.58- 3.73)	2.19	88 070 (85 080-91 930)	1.91	5.17 (4.98-5.42)	1.88	1.61 (1.33-1.89)
Region									
Andean Latin America	420 (370-490)	2.40	1.67 (1.50-1.92)	3.49	2350 (2020-2680)	3.93	4.12 (3.56-4.70)	3.73	3.69 (3.29-4.09)
Australasia	640 (600-690)	1.56	2.82 (2.61-3.03)	1.77	2030 (1770-2310)	1.85	5.29 (4.61-6.04)	1.74	2.87 (2.68-3.05)
Caribbean	560 (520-600)	1.85	1.94 (1.79-2.08)	3.06	1440 (1300-1600)	2.57	2.86 (2.58-3.16)	2.40	1.53 (1.30-1.77)
Central Asia	1020 (930-1160)	1.79	1.88 (1.71-2.13)	2.82	1740 (1610-1900)	3.47	2.01 (1.85-2.18)	2.86	−0.11 (−0.56 to 0.34)
Central Europe	4810 (4620-5010)	1.77	3.28 (3.14-3.42)	2.86	650 (6110-6950)	3.32	3.77 (3.54-4.06)	3.16	0.40 (0.27-0.54)
Central Latin America	2030 (1960-2110)	2.14	1.95 (1.89-2.02)	3.35	8520 (8090-9030)	3.84	3.44 (3.27-3.65)	3.38	1.93 (1.81-2.05)
Central sub-Saharan Africa	200 (150-270)	1.98	0.71 (0.57-0.92)	2.56	480 (380-680)	2.84	0.74 (0.59-1.04)	2.31	0.02 (−0.14 to 0.18)
East Asia	12 090 (10 540-13 220)	1.69	1.11 (0.99-1.22)	2.98	44 770 (41 550-50 710)	1.58	2.22 (2.06-2.53)	1.56	2.66 (2.31-3.01)
Eastern Europe	7850 (7250-8780)	2.40	2.88 (2.67-3.22)	2.53	14 500 (13 610-15530)	3.40	5.03 (4.70-5.40)	2.69	2.19 (1.88-2.51)
Eastern sub-Saharan Africa	1990 (1340-2640)	1.86	1.75 (1.24-2.28)	2.66	4590 (3910-5460)	2.57	1.84 (1.59-2.15)	2.01	−0.01 (−0.15 to 0.14)
High-income Asia Pacific	6680 (6400-7020)	2.08	3.25 (3.11-3.41)	3.10	20 620 (18 890-22 710)	2.69	7.21 (6.50-8.08)	2.81	4.43 (3.69-5.18)
High-income North America	11 980 (11 670-12 320)	1.48	3.68 (3.59-3.79)	1.52	28 280 (27 190-29 350)	1.53	5.44 (5.22-5.65)	1.47	1.32 (1.06-1.57)
North Africa and Middle East	3560 (2890-4410)	1.68	1.56 (1.27-1.96)	3.67	17 470 (15 990-19 890)	3.10	3.19 (2.93-3.65)	3.20	2.98 (2.86-3.11)
Oceania	70 (50-80)	1.81	1.74 (1.45-2.09)	2.87	190 (140-230)	2.90	2.14 (1.70-2.65)	2.98	0.73 (0.57-0.90)
South Asia	10 540 (8750-12 910)	1.88	1.25 (1.06-1.52)	3.37	37 970 (33 680-43 030)	3.55	2.29 (2.03-2.58)	3.30	2.30 (2.16-2.43)
Southeast Asia	6840 (5860-7840)	2.40	2.12 (1.82-2.47)	3.20	23 880 (21 430-29 120)	3.40	3.55 (3.21-4.33)	2.99	2.00 (1.95-2.04)
Southern Latin America	1100 (1030-1180)	1.82	2.31 (2.16-2.47)	2.57	2310 (2090-2590)	2.76	3.06 (2.75-3.44)	2.47	0.85 (0.66-1.04)
Southern Sub-Saharan Africa	390 (330-430)	2.07	1.09 (0.94-1.20)	3.05	760 (680-860)	2.95	1.16 (1.05-1.32)	2.36	−0.10 (−0.58 to 0.39)
Tropical Latin America	1830 (1750-1910)	2.79	1.65 (1.59-1.72)	2.48	5540 (5270-5770)	2.31	2.28 (2.17-2.38)	2.02	1.19 (1.00-1.38)
Western Europe	19 900 (19 190-20 630)	2.07	3.99 (3.85-4.14)	2.31	30 260 (28 580-32 030)	1.73	4.57 (4.31-4.87)	1.68	0.58 (0.36-0.80)
Western sub-Saharan Africa	530 (410-610)	1.57	0.49 (0.38-0.57)	2.29	1300 (1110-1560)	2.46	0.55 (0.47-0.66)	1.91	0.39 (0.30-0.47)

Abbreviations: ASIR, age-standardized incidence rate; EAPC, estimated annual percentage change; NA, not available; UI, uncertainty interval.

The 3 countries with the highest incident cases of thyroid cancer were the same from the beginning to the end of the study: China (11 016 in 1990 and 41 511 in 2017), the US (10 833 in 1990 and 25 896 in 2017), and India (7369 in 1990 and 25 675 in 2017) (eTable 3 in the Supplement). China had the highest number of deaths associated with thyroid cancer worldwide (3109; 95% UI, 2890-3636 in 1990; 6801; 95% UI, 6381-7433 in 2017) (eTable 4 in the Supplement). The country with the highest thyroid cancer DALYs changed from China (97 404.96; 95% CI, 89 652.29-110 236.18) in 1990 to India (202 323.54; 95% CI, 181 444.20-220 531.59) in 2017 (eTable 5 in the Supplement).

### Thyroid Cancer Incidence

**Figure 3.**
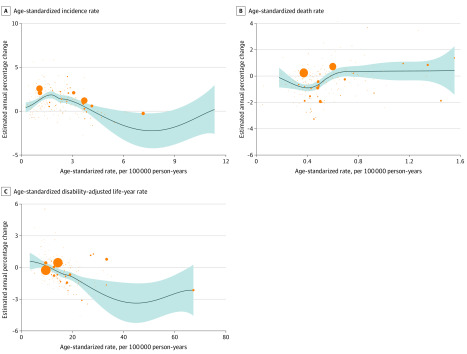
Correlation Between Estimated Annual Percentage Change and Thyroid Cancer Age-Standardized Rates Correlation with age-standardized incidence rate (A), death rate (B), and disability-adjusted life-year rate (C). The orange circles represent countries that were available on SDI data. The size of circle represents increases in the cases of thyroid cancer. The ρ indices and *P* values were derived from Pearson correlation analysis. Blue line and shaded area represent ρ and its 95%CI.

At a global level, from 1990 to 2017, thyroid cancer ASIR in most countries presented an upward trend (Figure 1A). Incident cases were greater among females than among males (female to male ratio, 1.92 in 1990 and 2.36 in 2017), as was the ASIR of thyroid cancer (female to male ratio, 2.65 in 1990 and 2.23 in 2017); however, the EAPC was larger in males (2.18; 95% CI, 2.07-2.28) than in females (EAPC, 1.38; 95% CI, 1.30-1.46) (Table). As reported in eTable 3 in the Supplement, the country with the highest ASIR changed from Iceland in 1990 (total: 30.18; 95% UI, 27.26-33.49; females: 15.64; 95% UI, 13.49-17.76; males: 6.97; 95% UI, 5.93-8.11) to South Korea in 2017 (total: 12.87; 95% UI, 11.04-15.23; females: 19.85; 95% UI, 16.41-24.29). However, the ASIR among males in Iceland was still the highest in 2017 (12.31; 95% UI, 10.22-14.80). The ASIR decreased the most in Qatar (total: EAPC, −2.50; 95% CI, −2.96 to −2.04; females: EAPC, −3.34; 95% CI, −4.01 to −2.67) and increased the most in South Korea (total: EAPC, 10.70; 95% CI, 8.85-12.59; females: EAPC, 11.05; 95% CI, 9.06-13.08; males: EAPC, 10.26; 95% CI, 8.87-11.67). The ASIR among males decreased most in Kazakhstan (EAPC, −2.25; 95% CI, −2.65 to −1.83). In addition, the EAPC was negatively correlated with ASIR (ρ = −0.18, *P* = .01) (Figure 3A) and positively associated with SDI (ρ = 0.21, *P* < .01) (eFigure 1 in the Supplement). The incident cases of thyroid cancer varied greatly among different regions. The region with the largest number of incident cases had changed from Western Europe (19 900; 95% UI, 19 190-20 630) in 1990 to East Asia (44 770; 95% UI, 41 550-50 710) in 2017. Oceania had the smallest number of incident cases (70; 95% UI, 50-80 in 1990 and 190; 95% UI, 140-230 in 2017) (Table). The ASIR increased most in high-income Asia Pacific among females (EAPC, 4.43; 95% CI, 3.69-5.18) and in East Asia for males (EAPC, 5.01; 95% CI, 4.56-5.47) (eTable 6 in the Supplement).

**Figure 4.**
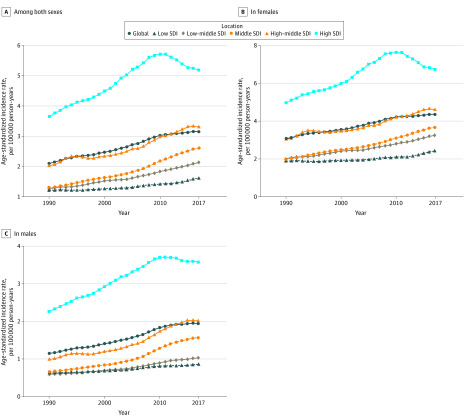
Change Trends of Thyroid Cancer Age-Standardized Incidence Rate Among Sex and Sociodemographic Index (SDI) Quintiles Changes shown in overall (A), females (B), and males (C) trends.

A third (34%) of patients with thyroid cancer resided in countries with a high SDI. As for various SDI quintiles, the fastest growth of ASIR was in the middle SDI quintile (EAPC, 2.65; 95% CI, 2.54-2.75) (Table). Previously, in both females and males, the countries with high SDI had the highest thyroid cancer incident cases (42 950 in 1990 and 88 070 in 2017) and ASIR (3.66 in 1990 and 5.17 in 2017), whereas the low SDI countries had the lowest (5710 in 1990 and 15 400 in 2017; 1.23 in 1990 and 1.63 in 2017) (eTable 6 in the Supplement). In addition, patients with thyroid cancer in the high SDI quintile were mostly aged 50 to 69 years (all ages, incident cases: 88070; 95% CI, 85081-91934); age 50-69 years, incident cases: 41666; 95% CI, 39921-43409), while those in the low (all ages, incident cases: 15404; 95% CI, 13734-17481); age 15-49 years, incident cases:9493; 95% CI, 8331-10916) and low-middle (all ages, incident cases: 31536; 95% CI, 28461-36747); age 15-49 years, incident cases: 19128; 95% CI,16514-22873) SDI quintiles were mostly aged 15 to 49 years (incident cases proportion of age 50-69 years in high SDI compared with that in the other 4 quintiles, *P* < .05) (eFigures 2-7 in the Supplement). Although the EAPC was positive (1.61; 95% CI, 1.33-1.89), the ASIR of the high SDI quintile started decreasing from 2010 among both females and males. The ASIR in the other 4 SDI quintiles (high-middle, middle, low-middle, and low) continued to increase over time (Figure 4). As for the age of individuals with thyroid cancer, the disease remained concentrated in the subgroup aged 15 to 69 years (eFigure 8 in the Supplement).

### Thyroid Cancer Death

As presented in eTable 4 in the Supplement, the country with the highest ASDR was Ethiopia (2.24; 95% UI, 1.46-3.21) in 1990 and the Philippines (1.55; 95% UI, 1.33-1.82) in 2017. The ASDR decreased most in Qatar (total: EAPC, −5.95; 95% CI, −6.52 to −5.37: females: EAPC, −7.08; 95% CI, −6.36 to −7.80) and increased most in Armenia (total: EAPC, 4.13; 95% CI, 3.12-5.15; females: EAPC, 4.29; 95% CI, 3.28-5.32; males: EAPC, 3.84; 95% CI, 2.83-4.85) and South Korea (total: EAPC, 3.70; 95% CI, 2.48-4.93; females: EAPC, 3.53; 95% CI, 2.13-4.93; males: EAPC, 4.10; 95% CI, 3.21-4.99). The ASDR decreased most in Kazakhstan (EAPC, –3.96; 95% CI, −4.33 to –3.58) among males. For females, the country with the highest ASDR changed from Ethiopia (2.72; 95% UI, 1.65-3.86) in 1990 to the Philippines (1.97; 95% UI, 1.60-2.44) in 2017; for males, it was the Maldives (1.93 in 1990 and 1.37 in 2017). The EAPC was positively correlated with ASDR (ρ = 0.28, *P* < .01) (Figure 3B) and negatively correlated with SDI (ρ = –0.17, *P* = .02) (eFigure 9 in the Supplement).

South Asia (3630 in 1990 and 8930 in 2017) and Oceania (20 in 1990 and 50 in 2017) had the largest and smallest numbers of thyroid cancer deaths, respectively (eTable 1 in the Supplement). As presented in eTable 6 in the Supplement, the ASDR increased most in Andean Latin America (total: EAPC, 1.08; 95% CI, 0.85-1.31; females: EAPC, 0.99; 95% CI, 0.78-1.19) and decreased most in Central Europe (total: EAPC, −2.15; 95% CI, −2.35 to −1.96; females: EAPC, −2.20; 95% CI, −2.36 to −2.04; males: EAPC, −2.08; 95% CI, −2.38 to −1.79). The ASDR of males increased most in East Asia (EAPC, 2.32; 95% CI, 1.94-2.70).

As reported in eTable 1 in the Supplement, the deaths from thyroid cancer increased in all SDI quintiles, but ASDRs increased only in the middle (EAPC, 0.33; 95% CI, 0.25-0.41) and low-middle (EAPC, 0.28; 95% CI, 0.23-0.33) SDI quintiles. From 1990 to 2017, the SDI quintile with the highest deaths had changed from high SDI (6900; 95% UI, 6810-6990) to middle SDI (11 610; 95% UI, 11 040-12 890). The low SDI quintile had the lowest number of deaths (2400 in 1990 and 4530 in 2017) and highest ASDRs (0.65 in 1990 and 0.62 in 2017). The ASDR decreased the most in the high SDI quintile (EAPC, −0.68; 95% CI, −0.74 to −0.62). Deaths associated with thyroid cancer in the high and high-middle SDI quintiles occurred mainly in persons older than 70 years; in other SDI quintiles, deaths were mainly in those aged 15 to 69 years (eg, high SDI: EAPC, −1.03; 95% CI, −1.09 to −0.97) (eTable 6 and eFigures 10-15 in the Supplement). The ASDRs of females decreased in all SDI quintiles. For males, the ASDR increased in all but the high SDI quintile (EAPC, −0.09; 95% CI, −0.18 to −0.01) (eTable 6 in the Supplement). However, from 2010, the ASDRs of males in all SDI quintiles started decreasing (high SDI, EAPC: −1.1; 95% CI, −1.2 to −0.9; high middle SDI, EAPC: −0.5; 95% CI, −1.1 to −0.2; middle SDI, EAPC: −0.2; 95% CI, −0.6 to 0.3; low-middle SDI, EAPC: −0.2; 95% CI, −0.7 to 0.3; low SDI, EAPC: −0.6; 95% CI, −0.9 to −0.3) (eFigures 16-18 in the Supplement). Thyroid cancer–associated deaths were seen most in people older than 70 years and showed a slightly increasing trend (high SDI, EAPC: −1.1; 95% CI, −1.2 to −0.9; high middle SDI, EAPC: −0.5; 95% CI, −1.1 to 0.2; middle SDI, EAPC: −0.2; 95% CI, −0.6 to 0.3; low-middle SDI, EAPC: −0.2; 95% CI, −0.7 to 0.3; low SDI, EAPC: −0.6; 95% CI, −0.9 to −0.3) (average annual percentage change, 0.10; 95% CI, 0.01-0.21; *P*<.05), followed by age 50 to 69 years (eFigure 19 in the Supplement).

### Thyroid Cancer DALYs

As shown in eTable 5 in the Supplement, females in India (63 063.09 in 1990 and 124 747.31 in 2017) and males in China (36 108.73 in 1990 and 97 467.41 in 2017) had the highest DALYs. The country with the highest age-standardized DALY rate changed from Ethiopia (67.28; 95% UI, 40.33-99.04) in 1990 to Pakistan (42.46; 95% UI, 31.33-58.20) in 2017. The age-standardized DALY rate decreased most in Qatar (EAPC, −4.59; 95% CI, −5.02 to −4.16) and increased most in South Korea (total: EAPC, 5.49; 95% CI, 4.18-6.82; females: EAPC, 5.66; 95% CI, 4.16-7.19; males: EAPC, 5.52; 95% CI, 4.50-6.55). The age-standardized DALY rate decreased most in Qatar among females (EAPC, −6.36; 95% CI, −7.02 to −5.68) and in Kazakhstan among males (EAPC, −4.13; 95% CI, −4.52 to −3.73). In addition, the EAPC was correlated with ASIR (ρ = −0.35, *P* < .01) (Figure 3A) but was not correlated with SDI (ρ = −0.08, *P* = .29) (eFigure 20 in the Supplement).

The region with the highest DALYs of thyroid cancer changed from East Asia (104 010) in 1990 to South Asia (297 710) in 2017. Conversely, the number of DALYs in Oceania remained the lowest (770 in 1990 and 1670 in 2017) (eTable 2 in the Supplement). The age-standardized DALY rate increased most in the high-income Asia-Pacific area (total: EAPC, 1.45; 95% CI, 0.99-1.91; females: EAPC, 1.34; 95% CI, 0.83-1.90; males: EAPC, 1.79; 95% CI, 1.40-2.19), and decreased most in Central Europe (EAPC, −2.03; 95% CI, −2.24 to −1.83). The EAPC was the lowest in Eastern sub-Saharan Africa (EAPC, −2.04, 95% CI, −2.20 to −1.88) among females and Central Europe among males (EAPC, −2.10; 95% CI, −2.38 to −1.82) (eTable 6 in the Supplement).

From 1990 to 2017, the SDI quintile with the highest DALYs changed from the high SDI (162 780) to the middle SDI quintile (314 770), while the lowest DALYs remained in the low SDI quintile (89 840 in 1990 and 153 320 in 2017) (eTable 2 in the Supplement). The EAPCs among females in all SDI quintiles were less than zero. The EAPCs among males were positive in all SDI quintiles except for the high SDI (EAPC, 0.03; 95% CI, −0.09 to 0.15) (eTable 6 in the Supplement). However, the age-standardized DALY rates of males in all SDI quintiles started decreasing in 2010 (eFigures 21-23 in the Supplement). In the high SDI quintile, the DALYs were concentrated in the age groups older than 50 years and 50 to 69 years in the high-middle and middle SDI quintiles, and in the age group of 15 to 49 years in the low-middle and low SDI quintiles (eFigures 24-29 in the Supplement).

The DALYs of global thyroid cancer were seen mainly among individuals aged 50 to 69 years, followed by 15 to 49 years. From 1990 to 2017, the proportion of DALYs in individuals older than 70 and 50 to 69 years increased, while those noted in the age groups of 15 to 49 and 5 to 14 years decreased (eFigure 30 in the Supplement).

## Discussion

In this study, from 1990 through 2017, the incidence, deaths, and DALYs of thyroid cancer and ASIR increased by 60% to 200%, whereas the ASDR and age-standardized DALY rates decreased. The increasing incidence of thyroid cancer in all SDI quintiles raises notable points. The ASIR in areas within a high SDI quintile continued to increase until 2010 and then began decreasing. Compared with the decreased age-standardized rates among females in all SDI quintiles, those rates among males continued to increase until 2010. In 2009, the American Thyroid Association’s guidelines on thyroid cancer diagnosis and treatment were revised substantially and other countries developed guidelines, which might be associated with the change noted from 2010.^20^ A previous study reported that males in communities with low socioeconomic status had poorer thyroid cancer–specific survival, but these findings did not appear to apply to women.^21^ Similarly, Nilubol et al^22^ suggested that males with thyroid cancer presented at an older age and had more advanced and aggressive disease, which is consistent with our results that the main age at onset of thyroid cancer in females (15-49 years) was younger than in males (50-69 years). Men appear to have advanced thyroid cancer at the time of diagnosis, leading to earlier cause-specific deaths associated with thyroid cancer^23^ possibly owing to sex differences in biology and behavioral attitudes in seeking medical care.^24^ However, established risk factors, such as radiation exposure and a family history of thyroid cancer, could not explain the increased incidence.^25^ There was no significant association between reproductive factors (menstrual, reproductive, or hormonal history) and thyroid cancer risk.^23,26,27^ Another study noted differences in the expression of estrogen receptor subtypes based on thyroid cancer histologic factors.^28^

Almost half of the incident cases of thyroid cancer were in Southern and Eastern Asia, which may be partly associated with the large population base. In South Korea, the increased incidence, ASIR, and age-standardized DALY rate were almost the highest seen in all regions; this increase might be associated with a cancer screening program begun there in 1999 that showed a 15-foled increase in the incidence of diagnosis of thyroid cancer.^29,30^ This increasing incidence of thyroid cancer in South Korea is an example of the change in health care policy. Most of the DALYs associated with thyroid cancer were also noted in Asia, where most middle- and low-middle SDI countries are located. Findings from Korea suggested that thyroid cancer has become 1 of the 3 most significant cancers affecting DALYs, which may have continued during 2000-2020.^31^ An analysis from the US Preventive Services Task Force^32^ observed that harms of thyroid cancer screening outweighed the benefits.^29,33^ Thyroid cancer is one of the most overdiagnosed and overtreated cancers^34^; however, overdiagnosis and overtreatment may not fully explain the cause of the increase in thyroid cancer burden. In Fukushima, Japan, the results of the thyroid ultrasonographic examination program may have been affected by a combination of overdiagnosis and radiation exposure.^35^ Therefore, policy makers there should consider the contemporary etiologic data, treatment information, iodine nutrition status, and living environment to establish targeted and specific strategies.

The anxiety of patients may lead to overtreatment, such as total thyroidectomy and thyroid replacement therapy, accompanied by various adverse effects, including hypoparathyroidism and vocal cord paralysis, as noted by McLeod et al.^36^ In addition, a Japanese study suggested that there is no substantial difference between immediate surgery and watchful waiting in preventing deaths from thyroid cancer.^37^ Therefore, for small papillary nodules, active periodic surveillance may be a reasonable and correct choice. The proportion of thyroid cancer deaths among individuals older than 70 years increased significantly, whereas the proportions of death among 3 other age groups decreased, which might be due to the improvement of treatment and aging of the population.^38^ In our study, the low SDI quintile presented the lowest number of deaths and highest DALYs associated with thyroid cancer. Nevertheless, the reason for the relatively low burden of thyroid cancer in districts with lower SDI cannot exclude the lack of advanced medical services and accurate laboratory investigations. The main age group in which thyroid cancer was associated with mortality was proportional to the SDI value, similar to the gap in medical standards and income worldwide.^39,40,41,42^

To our knowledge, this study presents the latest epidemiologic patterns of thyroid cancer burden at global, regional, and national levels among different sex, age, and SDI categories. Asia apparently carries the heaviest burden of thyroid cancer, while Oceania has the lowest. The most common onset age in persons who developed thyroid cancer decreased, and the age at death of those with thyroid cancer increased worldwide. Furthermore, people in lower SDI quintiles developed thyroid cancer and died from it earlier than those in other quintiles. In addition, growth patterns were significantly different between sexes and seemed to be reversed in the later years of the study. In our analysis, epidemiologic profiles of the thyroid cancer burden showed large heterogeneities. Investments in cancer prevention and treatment need to recognize the interdependence between socioeconomic status and health. To ensure balanced development of health services in all countries, greater efforts are needed to reduce these health inequities. Our research may provide data to support policy makers and other stakeholders in efforts to achieve equitable allocation of health care resources.

### Limitations

This study has limitations. As with other estimates of disease burden, the most important limitation of GBD is the lack of data at many sites. The key principle of GBD is to make full use of the data sources of all relevant resources. Data are available from a wide range of sources (>90 000 data sources). Although the diagnosis of thyroid cancer with inadequate, insufficiently specific, or unreliable registration has been corrected by a redistribution algorithm, the accuracy of diagnosis still has some unreliability. In addition, information bias regarding the epidemiologic evaluation of thyroid cancer was inevitable, as data are scarce in a few parts of the world. Given the restrictions of data type, to our knowledge, further investigation on thyroid cancer stratified by histologic characteristics, grade, and risk factors has not been conducted.

## Conclusions

In this study, the incidence, deaths, DALYs, and ASIR for thyroid cancer appeared to increase globally, signifying a larger burden on global health care systems, especially in females and countries with a high SDI. Nevertheless, the ASDR and age-standardized DALY rate in thyroid cancer decreased, which may be associated with improvement in therapeutic approaches. The thyroid cancer burden was largely heterogeneous across various categories evaluated, possibly reflecting differences in the corresponding genetic and environmental risk factors, as well as levels of economic status, education, lifestyle, and access to medical screening and therapeutic care. These factors need further investigation to ascertain detailed mechanisms.

## Notes

Supplement. **eAppendix.** GBD Overview **eFigure 1.** The Correlation Between EAPC of Thyroid Cancer Incidence and SDI in 2017 **eFigure 2.** The Age Subgroups of Thyroid Cancer Incident Cases Over 28 Years Worldwide **eFigure 3.** The Age Subgroups of Thyroid Cancer Incident Cases Among High SDI Quintiles Over 28 Years **eFigure 4.** The Age Subgroups of Thyroid Cancer Incident Cases Among High-Middle SDI Quintiles Over 28 Years **eFigure 5.** The Age Subgroups of Thyroid Cancer Incident Cases Among Middle SDI Quintiles Over 28 Years **eFigure 6.** The Age Subgroups of Thyroid Cancer Incident Cases Among Low-Middle SDI Quintiles Over 28 Years **eFigure 7.** The Age Subgroups of Thyroid Cancer Incident Cases Among Low SDI Quintiles Over 28 Years **eFigure 8.** The Proportion of Different Age Subgroups in Thyroid Cancer Incidence by Years **eFigure 9.** The Correlation Between EAPC of Thyroid Cancer Deaths and SDI in 2017 **eFigure 10.** The Age Subgroups of Thyroid Cancer Deaths Over 28 Years Worldwide **eFigure 11. **The Age Subgroups of Thyroid Cancer Deaths Among High SDI Quintiles Over 28 Years **eFigure 12. **The Age Subgroups of Thyroid Cancer Deaths Among High-Middle SDI Quintiles Over 28 Years **eFigure 13. **The Age Subgroups of Thyroid Cancer Deaths Among Middle SDI Quintiles Over 28 Years **eFigure 14. **The Age Subgroups of Thyroid Cancer Deaths Among Low-Middle SDI Quintiles Over 28 Years **eFigure 15.** The Age Subgroups of Thyroid Cancer Deaths Among Low SDI Quintiles Over 28 Years **eFigure 16.** The Change Trends of Thyroid Cancer ASDR Among Both Gender and SDI Quintiles **eFigure 17.** The Change Trends of Thyroid Cancer ASDR Among Females and SDI Quintiles **eFigure 18.** The Change Trends of Thyroid Cancer ASDR Among Males and SDI Quintiles **eFigure 19.** The Proportion of Different Age Subgroups in Thyroid Cancer Incidence by Years **eFigure 20.** The Correlation Between EAPC of Thyroid Cancer DALYs and SDI in 2017 **eFigure 21.** The Change Trends of Thyroid Cancer Age-Standardized DALY Rate Among Both Gender and SDI Quintiles **eFigure 22.** The Change Trends of Thyroid Cancer Age-Standardized DALY Rate Among Females and SDI Quintiles **eFigure 23.** The Change Trends of Thyroid Cancer Age-Standardized DALY Rate Among Males and SDI Quintiles **eFigure 24.** The Age Subgroups of Thyroid Cancer DALYs Over 28 Years Worldwide **eFigure 25.** The Age Subgroups of Thyroid Cancer DALYs Among High SDI Quintiles Over 28 Years **eFigure 26.** The Age Subgroups of Thyroid Cancer DALYs Among High-Middle SDI Quintiles Over 28 Years **eFigure 27.** The Age Subgroups of Thyroid Cancer DALYs Among Middle SDI Quintiles Over 28 Years **eFigure 28.** The Age Subgroups of Thyroid Cancer DALYs Among Low-Middle SDI Quintiles Over 28 Years **eFigure 29.** The Age Subgroups of Thyroid Cancer DALYs Among Low SDI Quintiles Over 28 Years **eFigure 30.** The Proportion of Different Age Subgroups in Thyroid Cancer DALYs by Years **eTable 1.** The Deaths From Thyroid Cancer and Its Temporal Trends From 1990 to 2017 **eTable 2.** The Disability-Adjusted Life-Years of Thyroid Cancer and Its Temporal Trends From 1990 to 2017 **eTable 3.** The Incidence of Thyroid Cancer Its Temporal Trends From 1990 to 2017 Among 195 Countries **eTable 4.** The Deaths From Thyroid Cancer and Its Temporal Trends From 1990 to 2017 Among 195 Countries **eTable 5.** The DALYs of Thyroid Cancer and Its Temporal Trends From 1990 to 2017 Among 195 Countries **eTable 6.** The EAPC of Thyroid Cancer Among SDI Quintiles and 21 Regions

## References

[1] Bray F, Ferlay J, Soerjomataram I, Siegel RL, Torre LA, Jemal A Global cancer statistics 2018: GLOBOCAN estimates of incidence and mortality worldwide for 36 cancers in 185 countries. CA Cancer J Clin. 2018;68(6):394-424. doi:10.3322/caac.21492 30207593

[2] Kitahara CM, Sosa JA The changing incidence of thyroid cancer. Nat Rev Endocrinol. 2016;12(11):646-653. doi:10.1038/nrendo.2016.110 27418023 PMC10311569

[3] Kent WD, Hall SF, Isotalo PA, Houlden RL, George RL, Groome PA Increased incidence of differentiated thyroid carcinoma and detection of subclinical disease. CMAJ. 2007;177(11):1357-1361. doi:10.1503/cmaj.061730 18025426 PMC2072986

[4] Cabanillas ME, McFadden DG, Durante C Thyroid cancer. Lancet. 2016;388(10061):2783-2795. doi:10.1016/S0140-6736(16)30172-6 27240885

[5] Pandeya N, McLeod DS, Balasubramaniam K, Increasing thyroid cancer incidence in Queensland, Australia 1982-2008—true increase or overdiagnosis? Clin Endocrinol (Oxf). 2016;84(2):257-264. doi:10.1111/cen.12724 25597380

[6] Keinan-Boker L, Silverman BG Trends of thyroid cancer in Israel: 1980-2012. Rambam Maimonides Med J. 2016;7(1). doi:10.5041/RMMJ.10228 26886958 PMC4737507

[7] Ahn HS, Kim HJ, Kim KH, Thyroid cancer screening in South Korea increases detection of papillary cancers with no impact on other subtypes or thyroid cancer mortality. Thyroid. 2016;26(11):1535-1540. doi:10.1089/thy.2016.0075 27627550

[8] Wang Y, Wang W Increasing incidence of thyroid cancer in Shanghai, China, 1983-2007. Asia Pac J Public Health. 2015;27(2):NP223-NP229. doi:10.1177/1010539512436874 22345304

[9] Veiga LH, Neta G, Aschebrook-Kilfoy B, Ron E, Devesa SS Thyroid cancer incidence patterns in Sao Paulo, Brazil, and the U.S. SEER program, 1997-2008. Thyroid. 2013;23(6):748-757. doi:10.1089/thy.2012.0532 23410185 PMC3675840

[10] Uhry Z, Colonna M, Remontet L, Estimating infra-national and national thyroid cancer incidence in France from cancer registries data and national hospital discharge database. Eur J Epidemiol. 2007;22(9):607-614. doi:10.1007/s10654-007-9158-6 17636414

[11] Colonna M, Uhry Z, Guizard AV, ; FRANCIM network Recent trends in incidence, geographical distribution, and survival of papillary thyroid cancer in France. Cancer Epidemiol. 2015;39(4):511-518. doi:10.1016/j.canep.2015.04.015 26003877

[12] Reynolds RM, Weir J, Stockton DL, Brewster DH, Sandeep TC, Strachan MW Changing trends in incidence and mortality of thyroid cancer in Scotland. Clin Endocrinol (Oxf). 2005;62(2):156-162. doi:10.1111/j.1365-2265.2004.02187.x 15670190

[13] Smailyte G, Miseikyte-Kaubriene E, Kurtinaitis J Increasing thyroid cancer incidence in Lithuania in 1978-2003. BMC Cancer. 2006;6:284. doi:10.1186/1471-2407-6-284 17156468 PMC1764427

[14] Ahn HS, Welch HG South Korea’s thyroid-cancer “epidemic”—turning the tide. N Engl J Med. 2015;373(24):2389-2390. doi:10.1056/NEJMc1507622 26650173

[15] Lubina A, Cohen O, Barchana M, Time trends of incidence rates of thyroid cancer in Israel: what might explain the sharp increase. Thyroid. 2006;16(10):1033-1040. doi:10.1089/thy.2006.16.1033 17042690

[16] Fitzmaurice C, Akinyemiju TF, Al Lami FH, ; Global Burden of Disease Cancer Collaboration Global, regional, and national cancer incidence, mortality, years of life lost, years lived with disability, and disability-adjusted life-years for 29 cancer groups, 1990 to 2016: a systematic analysis for the Global Burden of Disease Study. JAMA Oncol. 2018;4(11):1553-1568. doi:10.1001/jamaoncol.2018.2706 29860482 PMC6248091

[17] Global Health Data Exchange Accessed August 1, 2019. http://ghdx.healthdata.org/gbd-results-tool

[18] GBD 2017 Causes of Death Collaborators Global, regional, and national age-sex-specific mortality for 282 causes of death in 195 countries and territories, 1980-2017: a systematic analysis for the Global Burden of Disease Study 2017. Lancet. 2018;392(10159):1736-1788. doi:10.1016/S0140-6736(18)32203-7 30496103 PMC6227606

[19] Stevens GA, Alkema L, Black RE, ; The GATHER Working Group Guidelines for Accurate and Transparent Health Estimates Reporting: the GATHER statement. Lancet. 2016;388(10062):e19-e23. doi:10.1016/S0140-6736(16)30388-9 27371184

[20] Haugen BR, Alexander EK, Bible KC, 2015 American Thyroid Association management guidelines for adult patients with thyroid nodules and differentiated thyroid cancer: the American Thyroid Association Guidelines Task Force on thyroid nodules and differentiated thyroid cancer. Thyroid. 2016;26(1):1-133. doi:10.1089/thy.2015.0020 26462967 PMC4739132

[21] Asban A, Chung SK, Xie R, Gender and racial disparities in survival after surgery among papillary and patients with follicular thyroid cancer: a 45-year experience. Clin Med Insights Endocrinol Diabetes. 2019;12:1179551419866196. doi:10.1177/1179551419866196 31598065 PMC6764040

[22] Nilubol N, Zhang L, Kebebew E Multivariate analysis of the relationship between male sex, disease-specific survival, and features of tumor aggressiveness in thyroid cancer of follicular cell origin. Thyroid. 2013;23(6):695-702. doi:10.1089/thy.2012.0269 23194434 PMC3675841

[23] Kilfoy BA, Devesa SS, Ward MH, Gender is an age-specific effect modifier for papillary cancers of the thyroid gland. Cancer Epidemiol Biomarkers Prev. 2009;18(4):1092-1100. doi:10.1158/1055-9965.EPI-08-0976 19293311 PMC2667567

[24] Mazzaferri EL, Jhiang SM Long-term impact of initial surgical and medical therapy on papillary and follicular thyroid cancer. Am J Med. 1994;97(5):418-428. doi:10.1016/0002-9343(94)90321-2 7977430

[25] Dal Maso L, Bosetti C, La Vecchia C, Franceschi S Risk factors for thyroid cancer: an epidemiological review focused on nutritional factors. Cancer Causes Control. 2009;20(1):75-86. doi:10.1007/s10552-008-9219-5 18766448

[26] Brindel P, Doyon F, Rachédi F, Menstrual and reproductive factors in the risk of differentiated thyroid carcinoma in native women in French Polynesia: a population-based case-control study. Am J Epidemiol. 2008;167(2):219-229. doi:10.1093/aje/kwm288 17965111

[27] Megwalu UC, Saini AT Racial disparities in papillary thyroid microcarcinoma survival. J Laryngol Otol. 2017;131(1):83-87. doi:10.1017/S0022215116009737 27917722

[28] Rahbari R, Zhang L, Kebebew E Thyroid cancer gender disparity. Future Oncol. 2010;6(11):1771-1779. doi:10.2217/fon.10.127 21142662 PMC3077966

[29] Vaccarella S, Dal Maso L, Laversanne M, Bray F, Plummer M, Franceschi S The impact of diagnostic changes on the rise in thyroid cancer incidence: a population-based study in selected high-resource countries. Thyroid. 2015;25(10):1127-1136. doi:10.1089/thy.2015.0116 26133012

[30] Lee JH, Shin SW Overdiagnosis and screening for thyroid cancer in Korea. Lancet. 2014;384(9957):1848. doi:10.1016/S0140-6736(14)62242-X 25457916

[31] Park JH, Lee KS, Choi KS Burden of cancer in Korea during 2000-2020. Cancer Epidemiol. 2013;37(4):353-359. doi:10.1016/j.canep.2013.03.015 23643195

[32] The Lancet Thyroid cancer screening. Lancet. 2017;389(10083):1954. doi:10.1016/S0140-6736(17)31349-1 28534740

[33] Etzioni R, Gulati R Recognizing the limitations of cancer overdiagnosis studies: a first step towards overcoming them. J Natl Cancer Inst. 2015;108(3):djv345. doi:10.1093/jnci/djv345 26582245 PMC5072370

[34] Vaccarella S, Franceschi S, Bray F, Wild CP, Plummer M, Dal Maso L Worldwide thyroid-cancer epidemic? the increasing impact of overdiagnosis. N Engl J Med. 2016;375(7):614-617. doi:10.1056/NEJMp1604412 27532827

[35] Shibuya K, Gilmour S, Oshima A Time to reconsider thyroid cancer screening in Fukushima. Lancet. 2014;383(9932):1883-1884. doi:10.1016/S0140-6736(14)60909-0 24881983

[36] McLeod DS, Sawka AM, Cooper DS Controversies in primary treatment of low-risk papillary thyroid cancer. Lancet. 2013;381(9871):1046-1057. doi:10.1016/S0140-6736(12)62205-3 23668555

[37] Ito Y, Miyauchi A, Kihara M, Higashiyama T, Kobayashi K, Miya A Patient age is significantly related to the progression of papillary microcarcinoma of the thyroid under observation. Thyroid. 2014;24(1):27-34. doi:10.1089/thy.2013.0367 24001104 PMC3887422

[38] Lee R, Mason A; members of the NTA Network Is low fertility really a problem? population aging, dependency, and consumption. Science. 2014;346(6206):229-234. doi:10.1126/science.1250542 25301626 PMC4545628

[39] Mayor S UK children have “alarming gap” in health between rich and poor, report finds. BMJ. 2017;356:j377. doi:10.1136/bmj.j377 28126824

[40] Morris LG, Sikora AG, Tosteson TD, Davies L The increasing incidence of thyroid cancer: the influence of access to care. Thyroid. 2013;23(7):885-891. doi:10.1089/thy.2013.0045 23517343 PMC3704124

[41] Iacobucci G Life expectancy gap between rich and poor in England widens. BMJ. 2019;364:l1492. doi:10.1136/bmj.l1492 30923046

[42] Lorenzoni L, Belloni A, Sassi F Health-care expenditure and health policy in the USA versus other high-spending OECD countries. Lancet. 2014;384(9937):83-92. doi:10.1016/S0140-6736(14)60571-7 24993914

