# Supplementary material for: Global Burden of Thyroid Cancer From 1990 to 2017
Source: JAMA Netw Open. 2020 Jun 26;3(6):e208759. doi: 10.1001/jamanetworkopen.2020.8759 (PMC7320301; doi:10.1001/jamanetworkopen.2020.8759)
Supplement: Supplement. — eAppendix. GBD Overview eFigure 1. The Correlation Between EAPC of Thyroid Cancer Incidence and SDI in 2017 eFigure 2. The Age Subgroups of Thyroid Cancer Incident Cases Over 28 Years Worldwide eFigure 3. The Age Subgroups of Thyroid Cancer Incident Cases Among High SDI Quintiles Over 28 Years eFigure 4. The Age Subgroups of Thyroid Cancer Incident Cases Among High-Middle SDI Quintiles Over 28 Years eFigure 5. The Age Subgroups of Thyroid Cancer Incident Cases Among Middle SDI Quintiles Over 28 Years eFigure 6. The Age Subgroups of Thyroid Cancer Incident Cases Among Low-Middle SDI Quintiles Over 28 Years eFigure 7. The Age Subgroups of Thyroid Cancer Incident Cases Among Low SDI Quintiles Over 28 Years eFigure 8. The Proportion of Different Age Subgroups in Thyroid Cancer Incidence by Years eFigure 9. The Correlation Between EAPC of Thyroid Cancer Deaths and SDI in 2017 eFigure 10. The Age Subgroups of Thyroid Cancer Deaths Over 28 Years Worldwide eFigure 11. The Age Subgroups of Thyroid Cancer Deaths Among High SDI Quintiles Over 28 Years eFigure 12. The Age Subgroups of Thyroid Cancer Deaths Among High-Middle SDI Quintiles Over 28 Years eFigure 13. The Age Subgroups of Thyroid Cancer Deaths Among Middle SDI Quintiles Over 28 Years eFigure 14. The Age Subgroups of Thyroid Cancer Deaths Among Low-Middle SDI Quintiles Over 28 Years eFigure 15. The Age Subgroups of Thyroid Cancer Deaths Among Low SDI Quintiles Over 28 Years eFigure 16. The Change Trends of Thyroid Cancer ASDR Among Both Gender and SDI Quintiles eFigure 17. The Change Trends of Thyroid Cancer ASDR Among Females and SDI Quintiles eFigure 18. The Change Trends of Thyroid Cancer ASDR Among Males and SDI Quintiles eFigure 19. The Proportion of Different Age Subgroups in Thyroid Cancer Incidence by Years eFigure 20. The Correlation Between EAPC of Thyroid Cancer DALYs and SDI in 2017 eFigure 21. The Change Trends of Thyroid Cancer Age-Standardized DALY Rate Among Both Gender and SDI Quintiles eFigure 22. T [file jamanetwopen-3-e208759-s001.pdf]

Supplementary Online Content

Deng Y, Li H, Wang M, et al. Global burden of thyroid cancer from 1990 to 2017. *JAMA Netw Open*. 2020;3(6):e208759. doi:10.1001/jamanetworkopen.2020.8759

eAppendix. GBD Overview

eFigure 1. The Correlation Between EAPC of Thyroid Cancer Incidence and SDI in 2017

eFigure 2. The Age Subgroups of Thyroid Cancer Incident Cases Over 28 Years Worldwide

eFigure 3. The Age Subgroups of Thyroid Cancer Incident Cases Among High SDI Quintiles Over 28 Years

eFigure 4. The Age Subgroups of Thyroid Cancer Incident Cases Among High-Middle SDI Quintiles Over 28 Years

eFigure 5. The Age Subgroups of Thyroid Cancer Incident Cases Among Middle SDI Quintiles Over 28 Years

eFigure 6. The Age Subgroups of Thyroid Cancer Incident Cases Among Low-Middle SDI Quintiles Over 28 Years

eFigure 7. The Age Subgroups of Thyroid Cancer Incident Cases Among Low SDI Quintiles Over 28 Years

eFigure 8. The Proportion of Different Age Subgroups in Thyroid Cancer Incidence by Years

eFigure 9. The Correlation Between EAPC of Thyroid Cancer Deaths and SDI in 2017

eFigure 10. The Age Subgroups of Thyroid Cancer Deaths Over 28 Years Worldwide

eFigure 11. The Age Subgroups of Thyroid Cancer Deaths Among High SDI Quintiles Over 28 Years

eFigure 12. The Age Subgroups of Thyroid Cancer Deaths Among High-Middle SDI Quintiles Over 28 Years

eFigure 13. The Age Subgroups of Thyroid Cancer Deaths Among Middle SDI Quintiles Over 28 Years

eFigure 14. The Age Subgroups of Thyroid Cancer Deaths Among Low-Middle SDI Quintiles Over 28 Years

eFigure 15. The Age Subgroups of Thyroid Cancer Deaths Among Low SDI Quintiles Over 28 Years

eFigure 16. The Change Trends of Thyroid Cancer ASDR Among Both Gender and SDI Quintiles

eFigure 17. The Change Trends of Thyroid Cancer ASDR Among Females and SDI Quintiles

eFigure 18. The Change Trends of Thyroid Cancer ASDR Among Males and SDI Quintiles

eFigure 19. The Proportion of Different Age Subgroups in Thyroid Cancer Incidence by Years

eFigure 20. The Correlation Between EAPC of Thyroid Cancer DALYs and SDI in 2017

eFigure 21. The Change Trends of Thyroid Cancer Age-Standardized DALY Rate Among Both Gender and SDI Quintiles

eFigure 22. The Change Trends of Thyroid Cancer Age-Standardized DALY Rate Among Females and SDI Quintiles

eFigure 23. The Change Trends of Thyroid Cancer Age-Standardized DALY Rate Among Males and SDI Quintiles

eFigure 24. The Age Subgroups of Thyroid Cancer DALYs Over 28 Years Worldwide

eFigure 25. The Age Subgroups of Thyroid Cancer DALYs Among High SDI Quintiles Over 28 Years

eFigure 26. The Age Subgroups of Thyroid Cancer DALYs Among High-Middle SDI Quintiles Over 28 Years

eFigure 27. The Age Subgroups of Thyroid Cancer DALYs Among Middle SDI Quintiles Over 28 Years

eFigure 28. The Age Subgroups of Thyroid Cancer DALYs Among Low-Middle SDI Quintiles Over 28 Years

eFigure 29. The Age Subgroups of Thyroid Cancer DALYs Among Low SDI Quintiles Over 28 Years

eFigure 30. The Proportion of Different Age Subgroups in Thyroid Cancer DALYs by Years

eTable 1. The Deaths From Thyroid Cancer and Its Temporal Trends From 1990 to 2017

eTable 2. The Disability-Adjusted Life-Years of Thyroid Cancer and Its Temporal Trends From 1990 to 2017

eTable 3. The Incidence of Thyroid Cancer Its Temporal Trends From 1990 to 2017 Among 195 Countries

eTable 4. The Deaths From Thyroid Cancer and Its Temporal Trends From 1990 to 2017 Among 195 Countries

eTable 5. The DALYs of Thyroid Cancer and Its Temporal Trends From 1990 to 2017 Among 195 Countries

eTable 6. The EAPC of Thyroid Cancer Among SDI Quintiles and 21 Regions

© 2020 Deng Y et al. *JAMA Network Open.*

This supplementary material has been provided by the authors to give readers additional information about their work.

© 2020 Deng Y et al. *JAMA Network Open.*

eAppendix. GBD Overview

The Global Burden of Disease (GBD) is an approach to global descriptive epidemiology. It is a systematic, scientific effort to quantify the comparative magnitude of health loss due to diseases, injuries, and risk factors by age, sex, and geographies for specific points in time. IHME serves as the coordinating center for the GBD and affiliated projects. Incidence data is obtained from individual cancer registries or aggregated databases of cancer registries, such as CI5 (cancer incidence of five continents), SEER, EUERG or NORDCAN. GBD study relies on a lot of data – over 90,000 data sources. GBD produces regular estimates of all-cause mortality, deaths by cause, years of life lost due to premature mortality (YLLs), years lived with disability (YLDs), and disability-adjusted life years (DALYs) for a cause list. The cause list is agreed upon annually by the Scientific Council. The critical milestones for ongoing estimation include regular updates to the GBD estimates, referred to as the "GBD round." For each round, the entire time series back to 1990 is re-estimated using all available data to ensure the most complete and highly comparable set of estimates possible. Previous results will be archived every time new results are released. The GBD provides cutting-edge and timely results through scientific papers, policy reports, web content, and interactive visualizations.

Published in November 2018, GBD 2017 provides for the first time an independent estimation of population, for each of 195 countries and territories and the globe, using a standardized, replicable approach, as well as a comprehensive update on fertility. GBD 2017 incorporates major data additions and improvements, and methodological refinements. Mortality and life expectancy estimates have been extended back to 1950, and new causes have been added to the fatal and non-fatal cause lists, for a total of 359 diseases and injuries (http://www.healthdata.org/gbd/about/protocol).

Definition of indicator

The GBD cause list is organized in a hierarchy. Levels 1 and 2 represent general groupings. The broad group "neoplasms." which includes all malignant and benign neoplasms, is at Level 2 under the Level 1 group "Non-communicable diseases." Level 3 includes 29 cancer groups, and Level 4 includes 37 groups since in Level 4, leukemia, liver cancer, and non-melanoma skin cancer are further subdivided. In this publication, estimates for the GBD cancer groups, for both sexes, for the time from 1980 to 2017, and for the 5-year GBD age groups (0-5; 5-9; etc. until 95+) are presented for 195 countries or territories. Thyroid cancer with ICD10(C73, Z85.850) and ICD9 (193-193.9).

Data analysis

ASRs were calculated on the basis of the following formula:

$$ASR = \frac{\sum_{i=1}^{A} a_i w_i}{\sum_{i=1}^{A} w_i} \times 100{,}000$$

The ASR (per 100,000 population) is equal to the sum of the product of the specific age ratio (ai) in age group i and the number (or weight) (wi) of the selected reference standard population group i divided by the sum of number (or weight) of the standard population, i.e.

Meanwhile, EAPC, which is approximately equal to the annual change for a specified range, was calculated using the following regression model to assess the trends in ASR:

Y=α+βX+ε, where y refers to ln(ASR), x represents calendar year, ε means error term, and β determines the positive or negative trends in ASR. The EAPC could be given by 100*(exp(β)-1), as well as its 95% confidence interval (CI).

© 2020 Deng Y et al. *JAMA Network Open.*

Disability adjusted life-years were also defined as years of healthy life lost, estimated by the sum of the YLLs and the YLDs. YLLs, the years of life lost due to premature mortality, are the multiplication of deaths and a standard life expectancy at each age of death. In each population greater than 5 million, the standard life expectancy was estimated from a life table which includes the lowest observed mortality at each age group. YLDs were defined as the years lived with any short-term or long-term health loss weighted for severity by the disability weights, calculated by multiplying prevalence estimate and the disability weight of each mutually exclusive sequela for prostate cancer, such as disability due to incontinence and impotence from prostatectomy.

Data sources

Cancer incidence data sources

Cancer incidence was sought from individual cancer registries or aggregated databases of cancer registry data like "Cancer Incidence In Five Continents" (CI5)," EUREG, or NORDCAN. Data were excluded if they were not representative of the coverage population (e.g., hospital-based registries), if they did not cover all malignant neoplasms as defined in ICD9 (140-208) or ICD10 (C00-C96) (e.g., specialty cancer registry), if they did not include data for both sexes and all age groups, if the data were limited to years prior to 1980, or if the source did not provide details on the population covered. Preference was given to registries with national coverage over those with only local coverage, except those from countries where the GBD study provides subnational estimates. Additional metadata for each source are available in the online GBD citation tool, http://ghdx.healthdata.org/gbd-2017.

Cancer mortality data sources

A detailed description of the data sources and processing steps for the cause of death database can be found in the appendix to the GBD 2017 paper "Global, regional, and national age-sex-specific mortality for 282 causes of death in 195 countries and territories, 1980–2017: a systematic analysis for the Global Burden of Disease Study 2017."

Incidence estimation

GBD cancer incidence estimates were generated by dividing final mortality estimates (after CoDCorrect adjustment) by the MI ratio for the specific cancer. To propagate uncertainty from the MI ratios and the mortality estimates to incidence, this process was done at the 1,000-draw level. It was assumed that uncertainty in the MI ratio is independent of uncertainty in the estimated age-specific death rates.

Bias of categories of input data

Bias of the input data included for the COD database is described elsewhere. Cancer registry data can be biased in multiple ways. A high proportion of ill-defined cancer cases in the registry data requires redistribution of these cases to other cancers, which introduces a potential for bias. Changes between coding systems can lead to artificial differences in disease estimates; however, we adjust for this bias by mapping the different coding systems to the GBD causes. Since many cancer registries are located in urban areas, the representativeness of the registry for the general population can also be problematic. The accuracy of mortality data reported in cancer registries usually depends on the quality of the vital registration system. If the vital registration system is incomplete or of poor quality, the mortality-to-incidence ratio can be biased to lower ratios.

Input data

To estimate the prevalence of each of these categories for all locations, by age, year, and sex, the prevalence of these neoplasms from hospital data was used as input for a prevalence model in DisMod-MR 2.1. These inputs included MarketScan claims data from the United States in the years 2000, 2010, and 2012, as well as hospital and outpatient data from other health systems worldwide. Each of these data sources were crosswalked to the 2012 MarketScan data.

[e

© 2020 Deng Y et al. *JAMA Network Open.*

Figure 1. The correlation between EAPC of thyroid cancer incidence and SDI in 2017.

Figure legends: The circles represent countries that were available on SDI data. The size of circle is increased with the cases of thyroid cancer. The ρ indices and P values presented in and were derived from Pearson correlation analysis. EAPC, estimated annual percentage change; SDI, socio-demographic index.

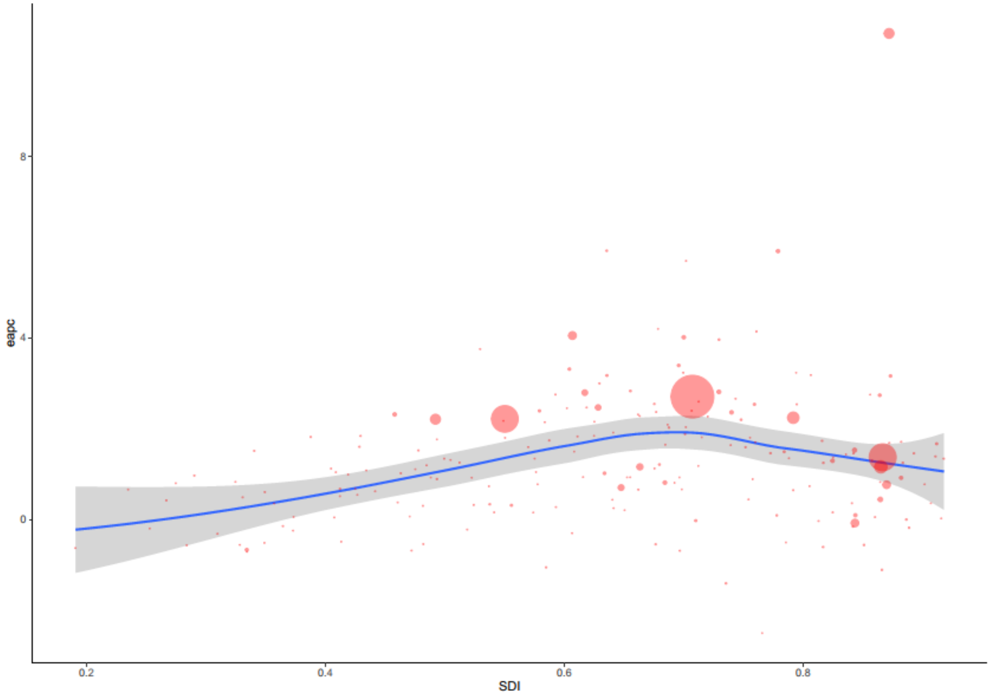

© 2020 Deng Y et al. *JAMA Network Open.*

eFigure 2. The age subgroups of thyroid cancer incident cases over 28 years worldwide.

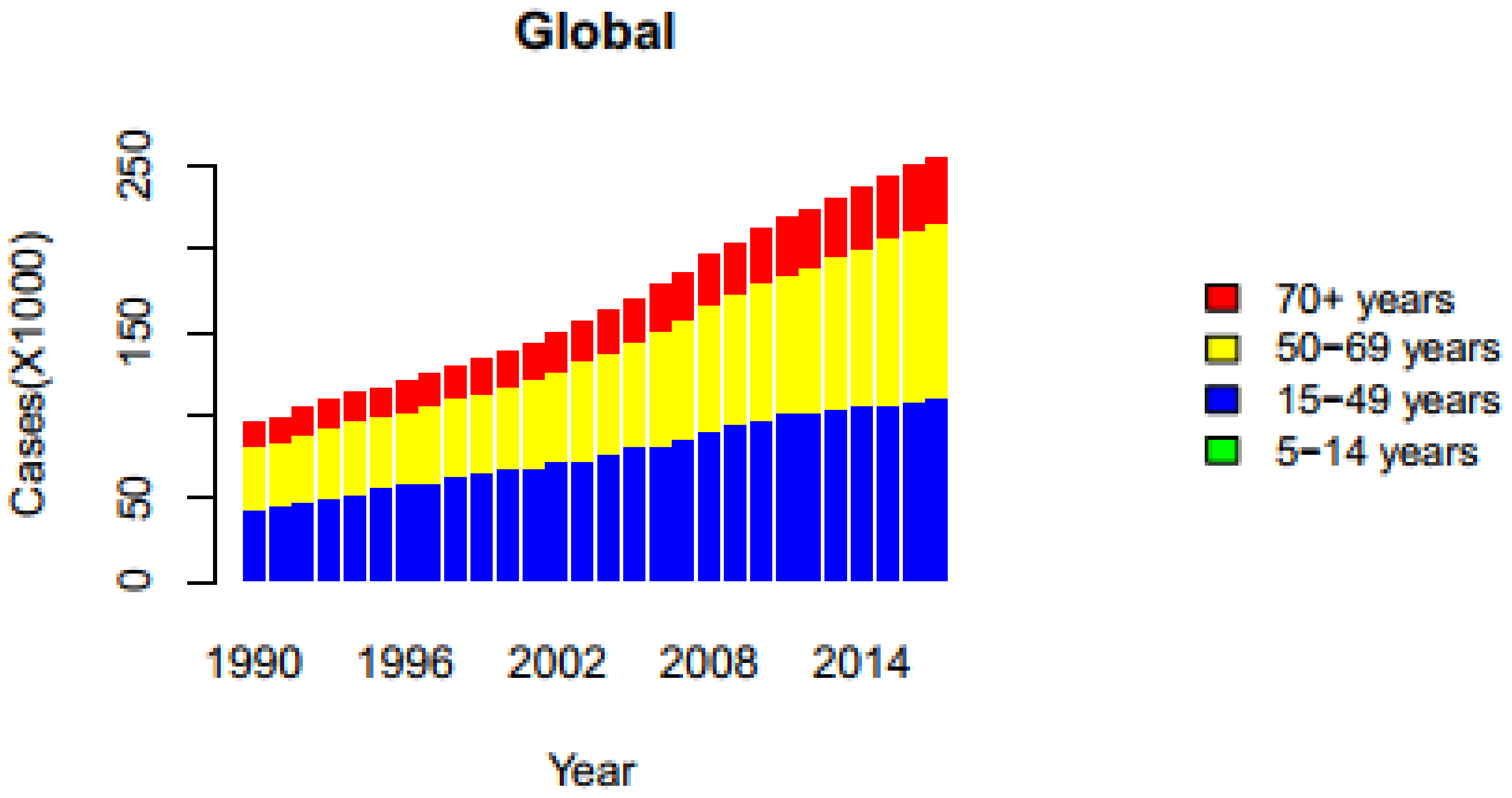

© 2020 Deng Y et al. *JAMA Network Open.*

eFigure 3.The age subgroups of thyroid cancer incident cases among high SDI quintiles over 28 years.

Figure legends: SDI, socio-demographic index.

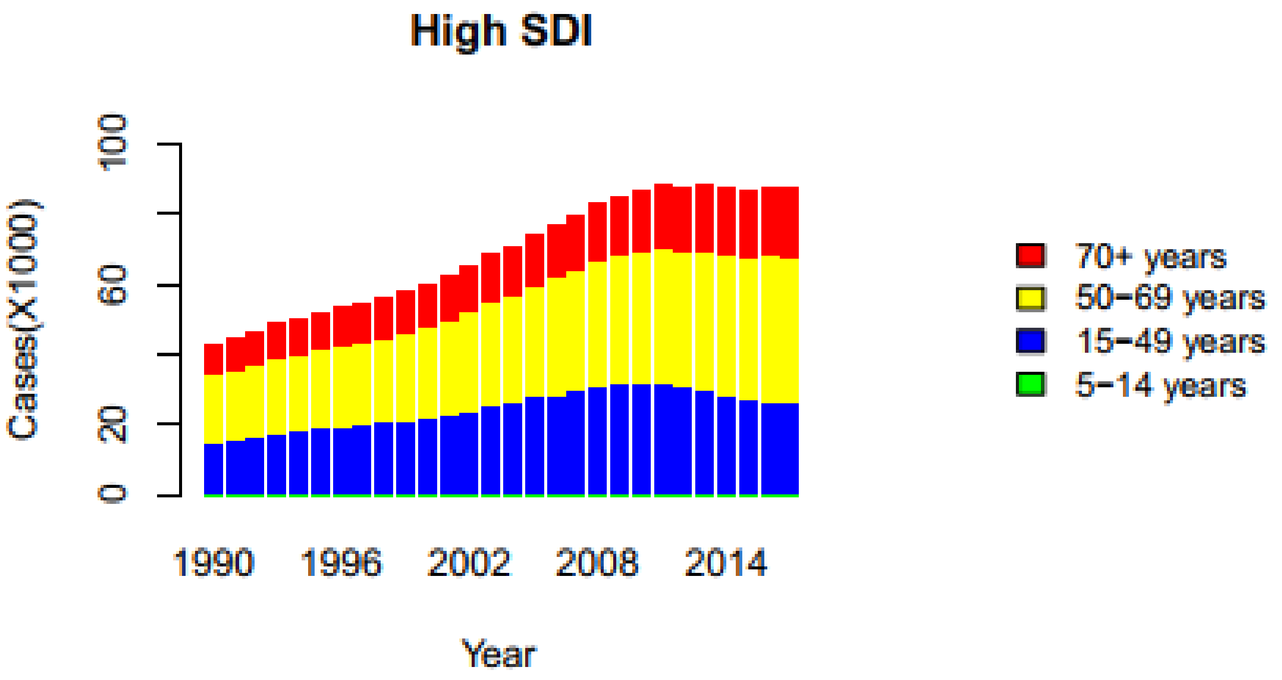

© 2020 Deng Y et al. *JAMA Network Open.*

eFigure 4.The age subgroups of thyroid cancer incident cases among high-middle SDI quintiles over 28 years.

Figure legends: SDI, socio-demographic index.

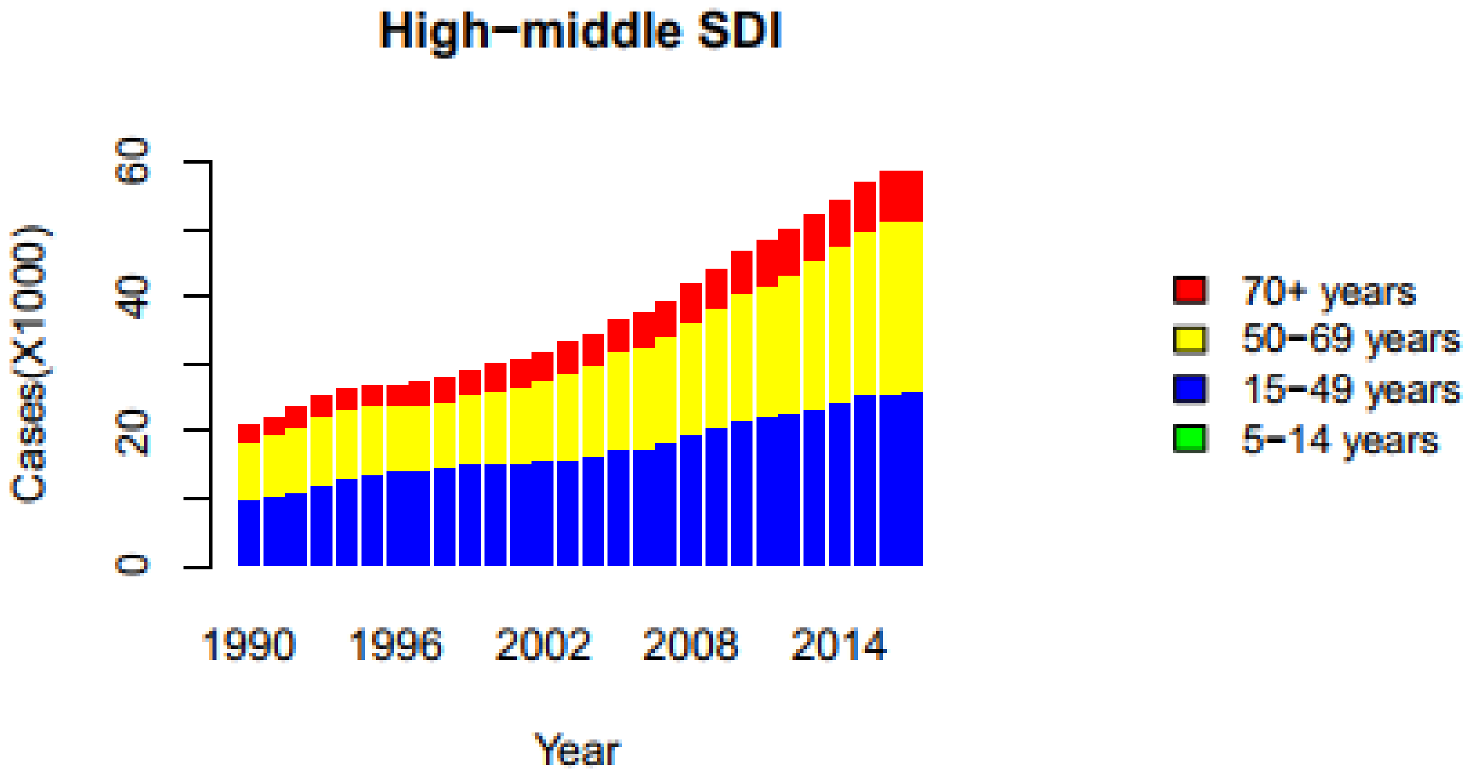

© 2020 Deng Y et al. *JAMA Network Open.*

eFigure 5.The age subgroups of thyroid cancer incident cases among middle SDI quintiles over 28 years.

Figure legends: SDI, socio-demographic index.

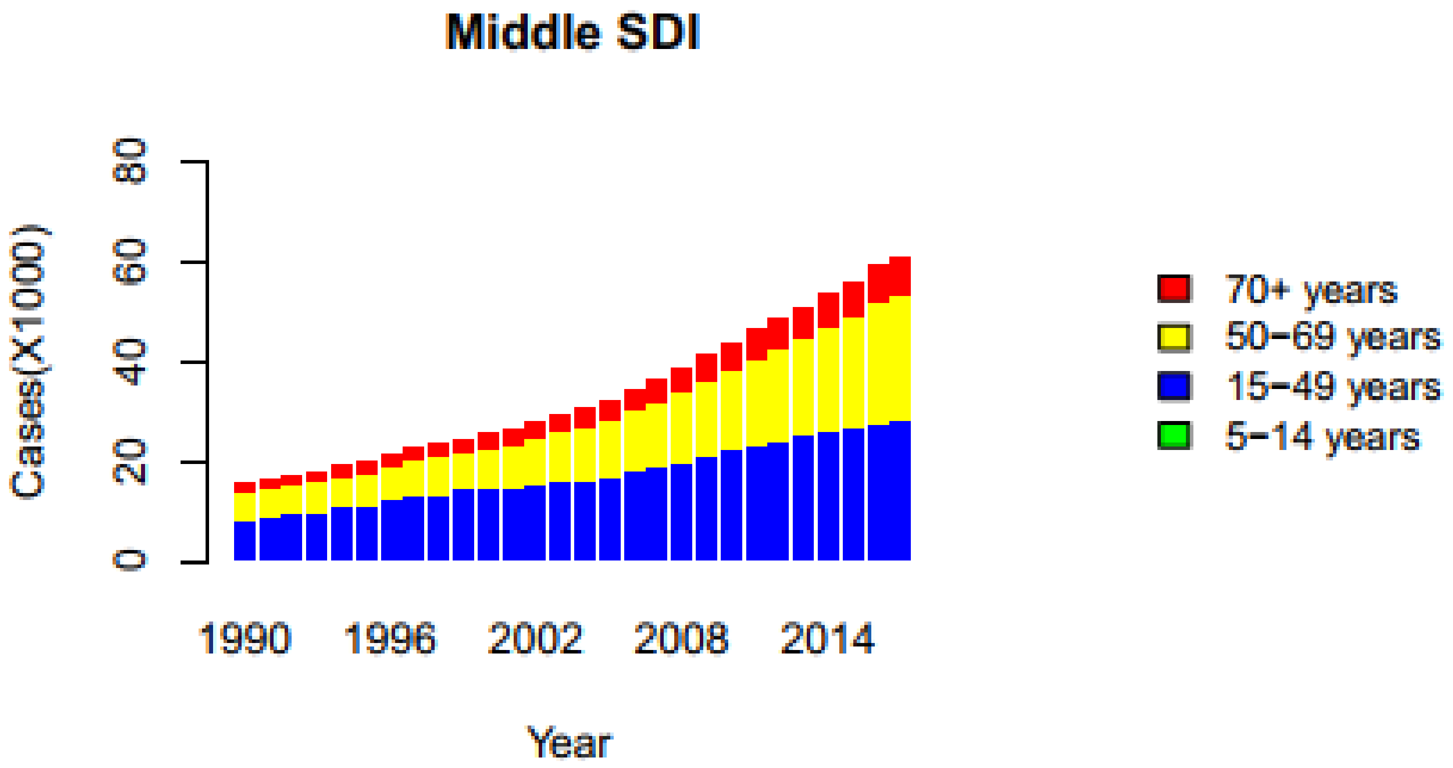

© 2020 Deng Y et al. *JAMA Network Open.*

eFigure 6.The age subgroups of thyroid cancer incident cases among low-middle SDI quintiles over 28 years.

Figure legends: SDI, socio-demographic index.

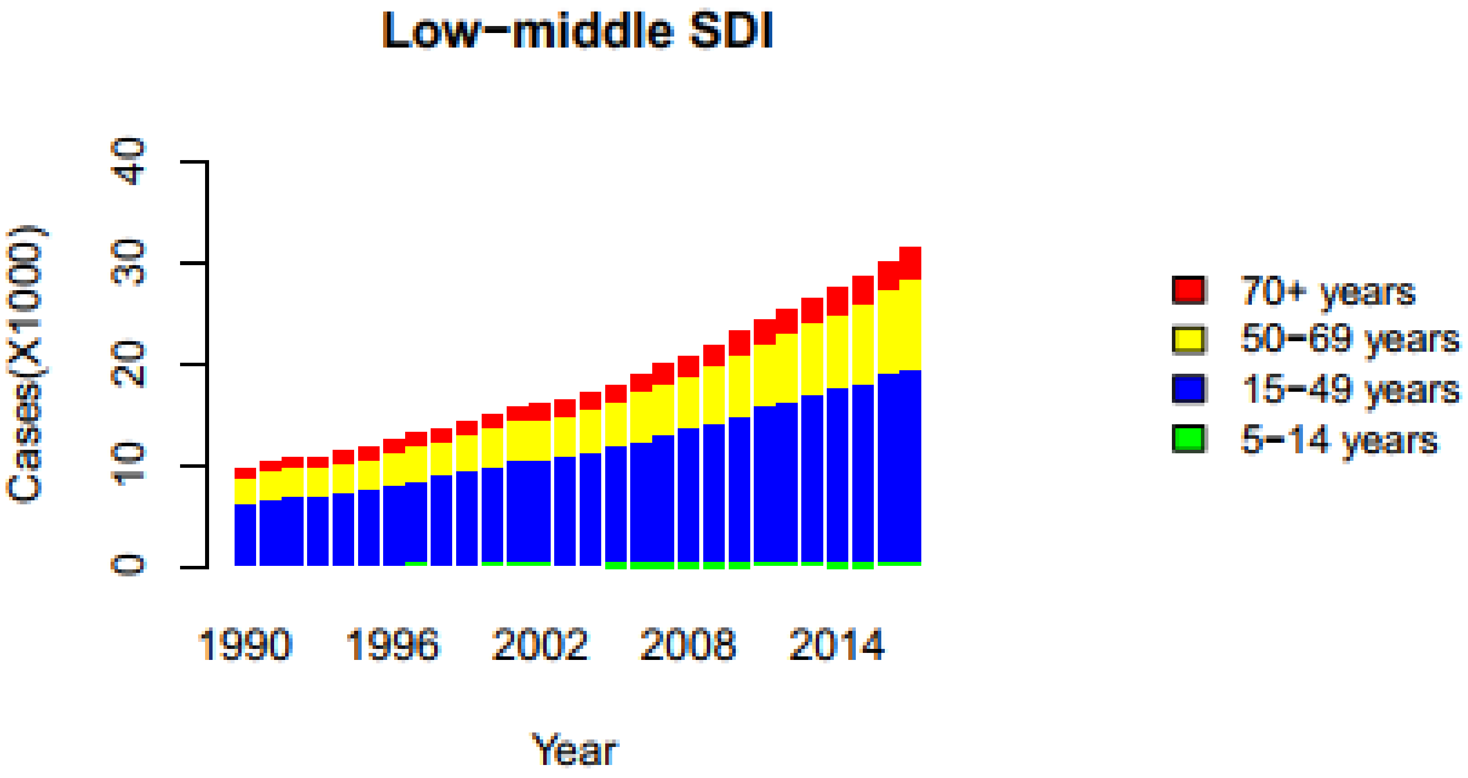

© 2020 Deng Y et al. *JAMA Network Open.*

eFigure 7.The age subgroups of thyroid cancer incident cases among low SDI quintiles over 28 years.

Figure legends: SDI, socio-demographic index.

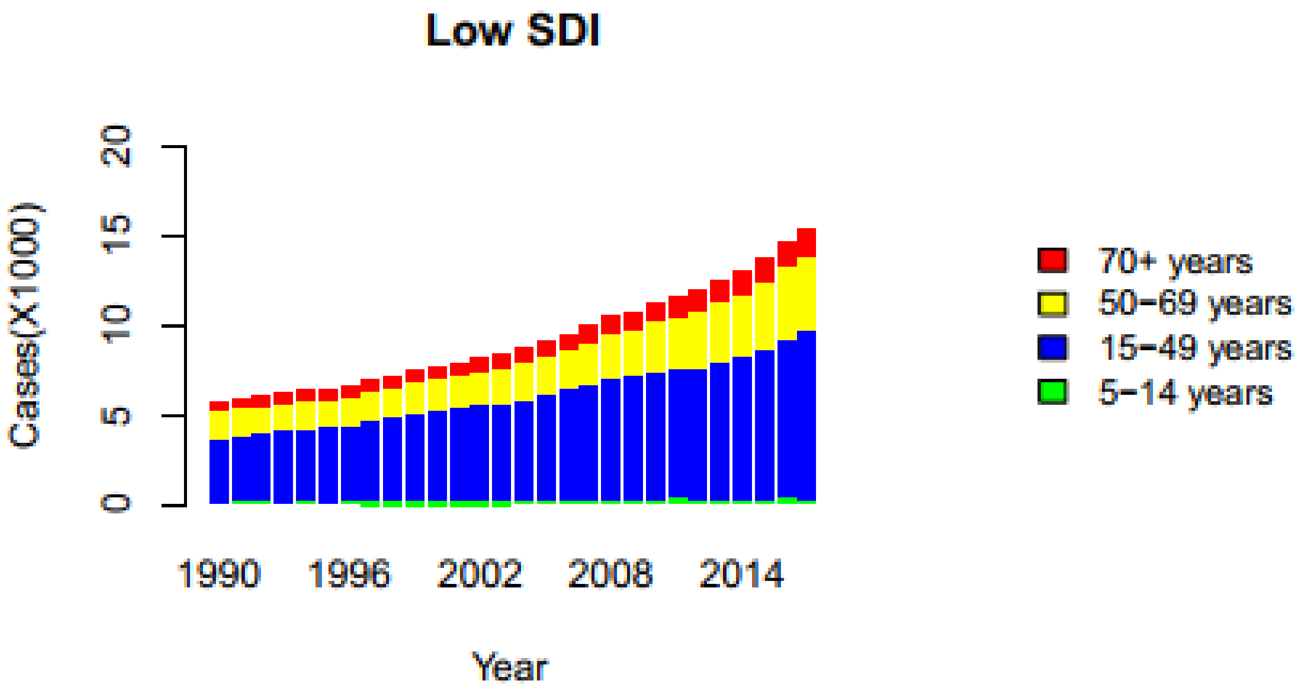

© 2020 Deng Y et al. *JAMA Network Open.*

eFigure 8. The proportion of different age subgroups in thyroid cancer incidence by years.

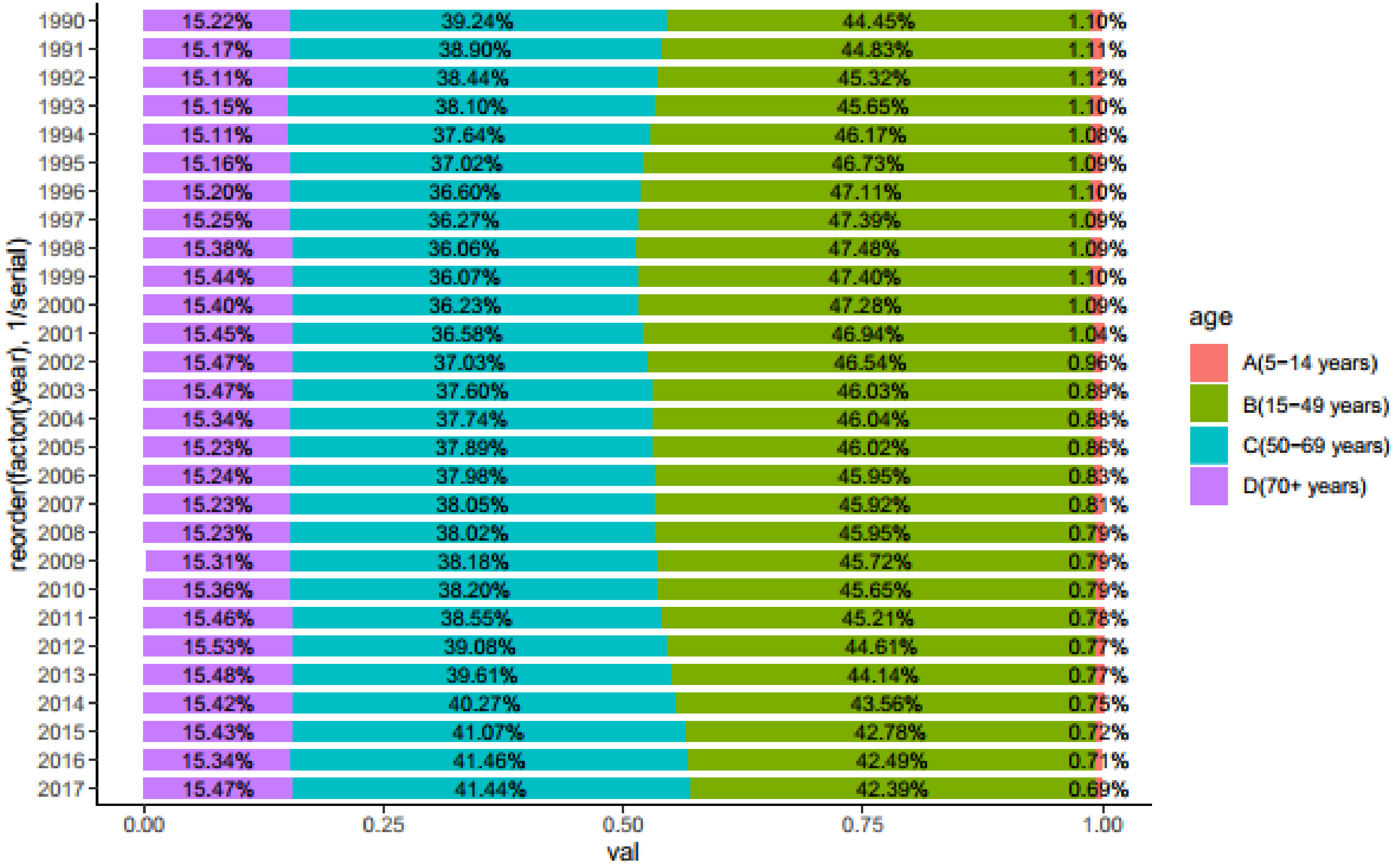

© 2020 Deng Y et al. *JAMA Network Open.*

eFigure 9. The correlation between EAPC of thyroid cancer deaths and SDI in 2017.

Figure legends: The circles represent countries that were available on SDI data. The size of circle is increased with the cases of thyroid cancer. The ρ indices and P values presented in and were derived from Pearson correlation analysis. EAPC, estimated annual percentage change; SDI, socio-demographic index.

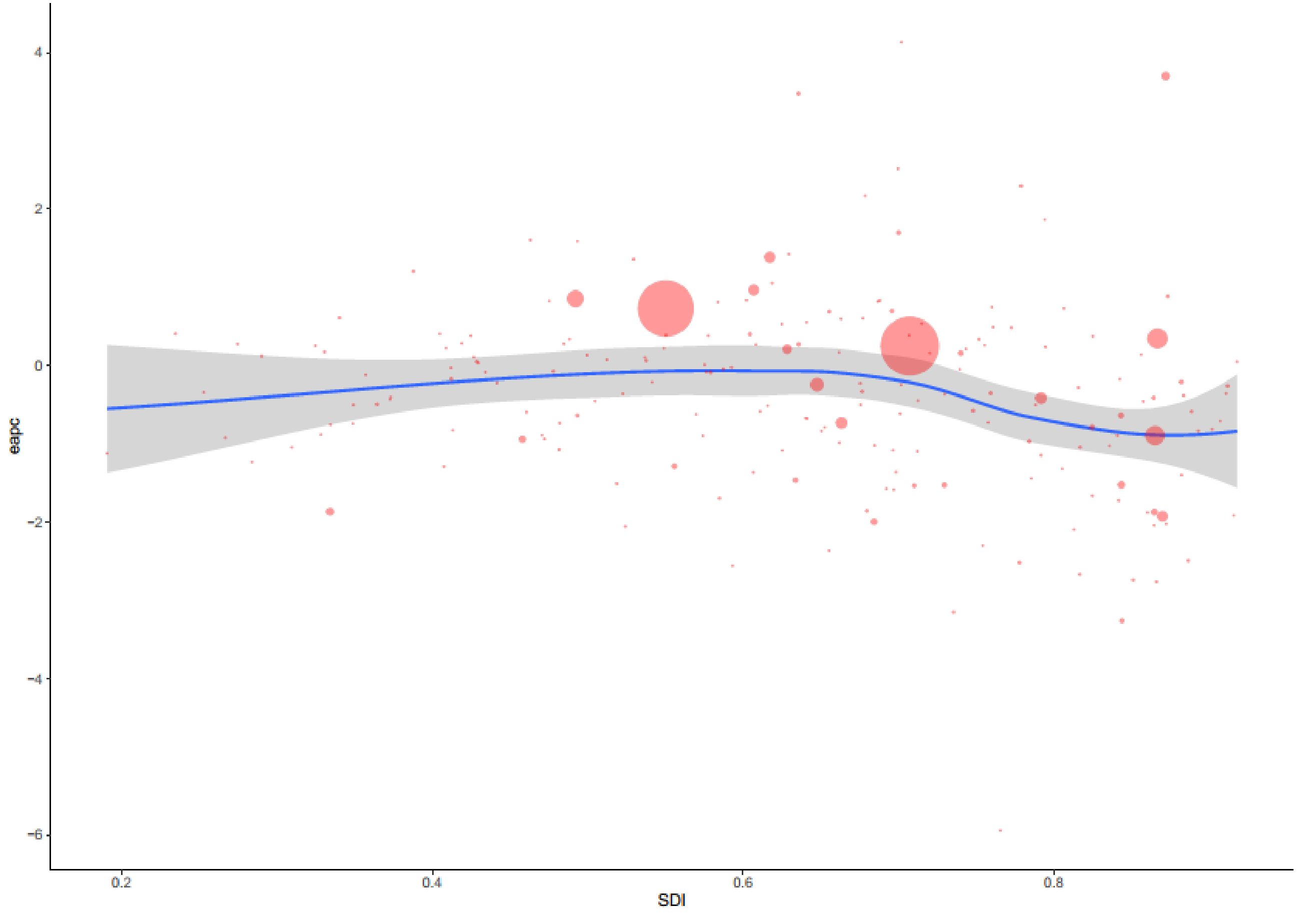

© 2020 Deng Y et al. *JAMA Network Open.*

.eFigure 10. The age subgroups of thyroid cancer deaths over 28 years worldwide.

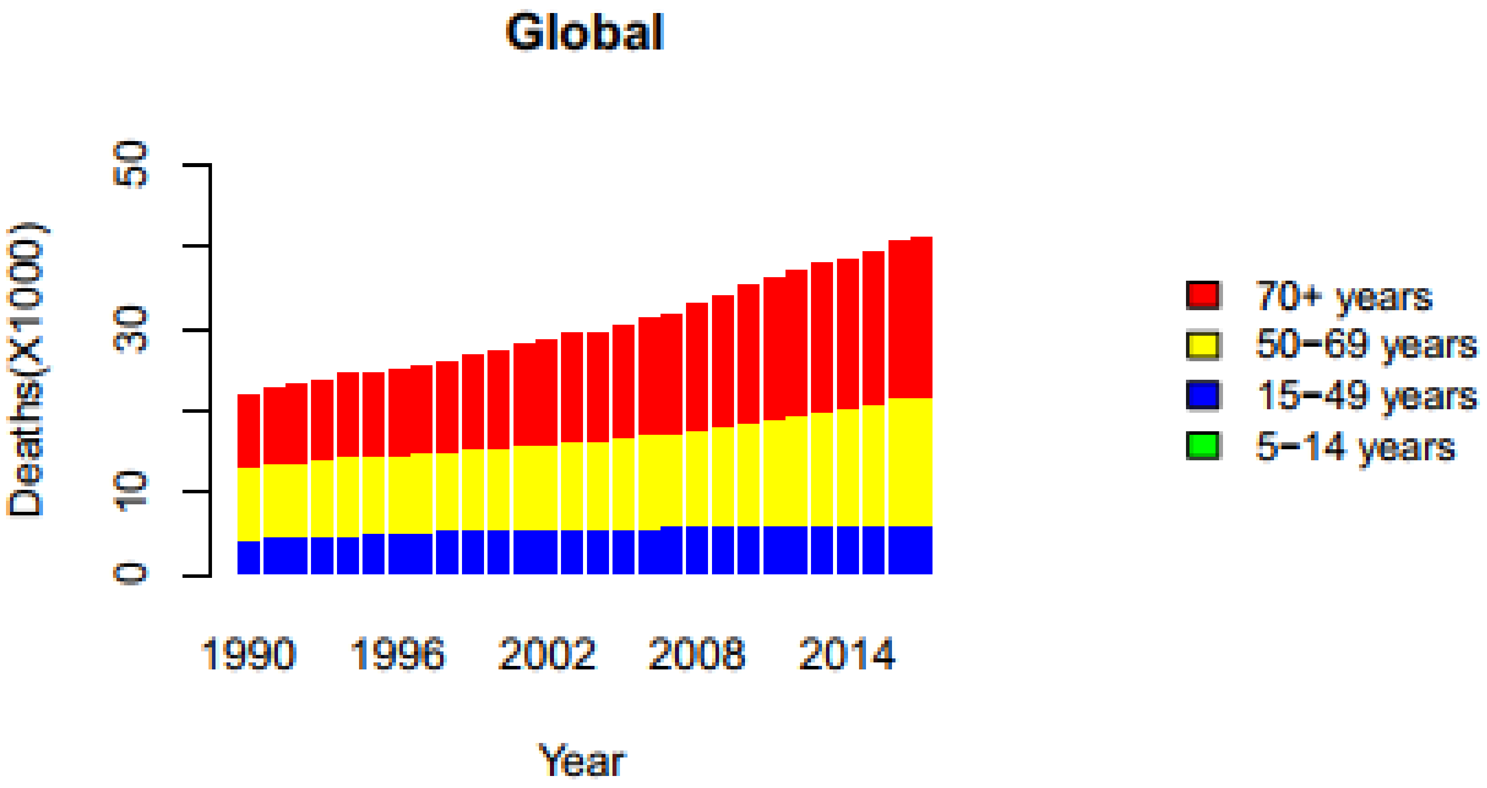

© 2020 Deng Y et al. *JAMA Network Open.*

eFigure 11.The age subgroups of thyroid cancer deaths among high SDI quintiles over 28 years.

Figure legends: SDI, socio-demographic index.

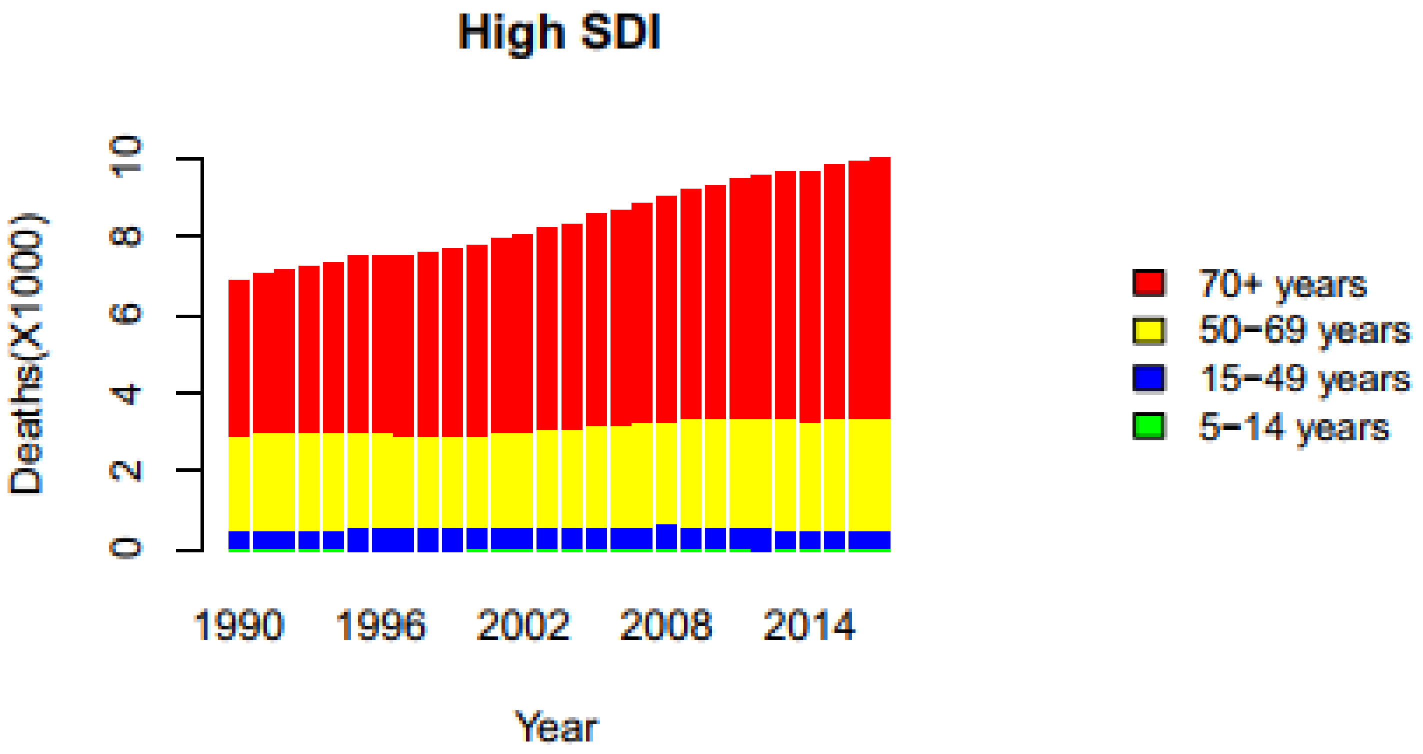

© 2020 Deng Y et al. *JAMA Network Open.*

eFigure 12.The age subgroups of thyroid cancer deaths among high-middle SDI quintiles over 28 years.

Figure legends: SDI, socio-demographic index.

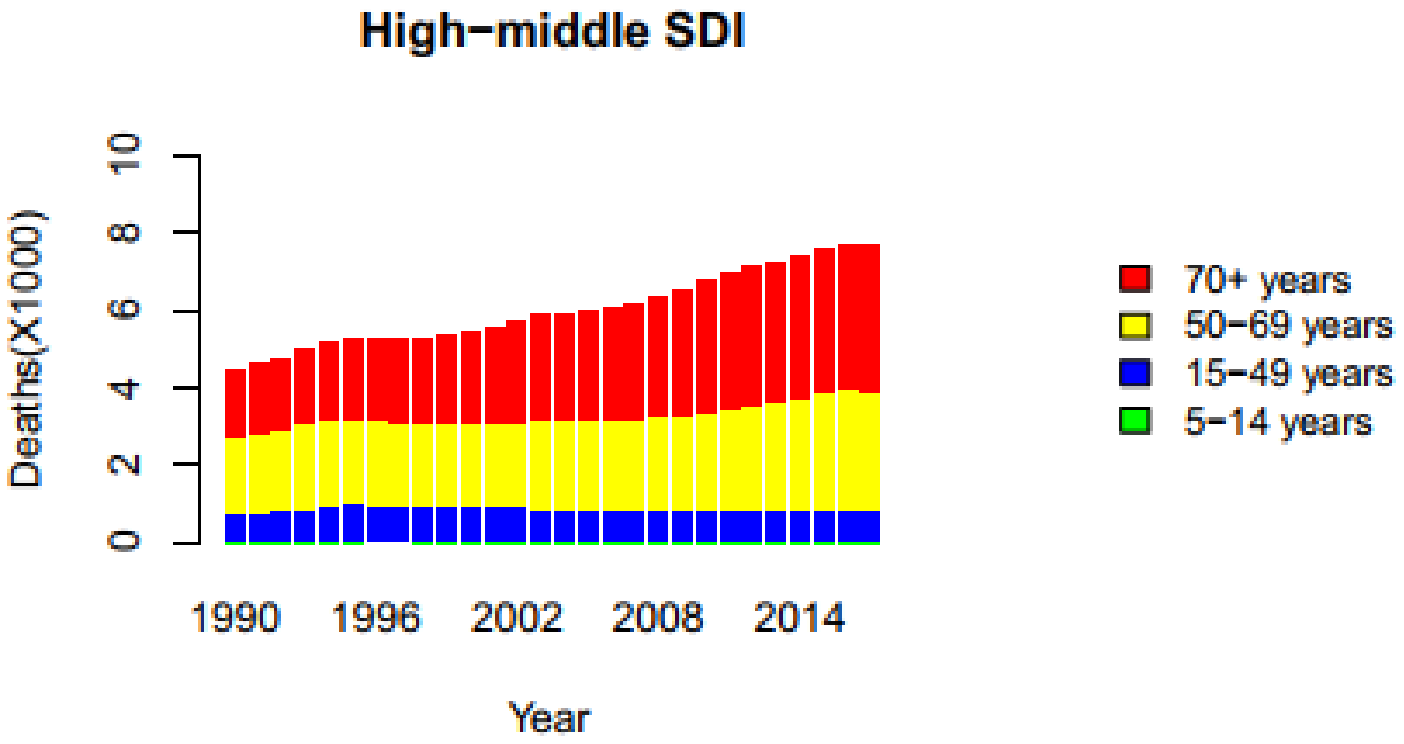

© 2020 Deng Y et al. *JAMA Network Open.*

eFigure 13.The age subgroups of thyroid cancer deaths among middle SDI quintiles over 28 years.

Figure legends: SDI, socio-demographic index.

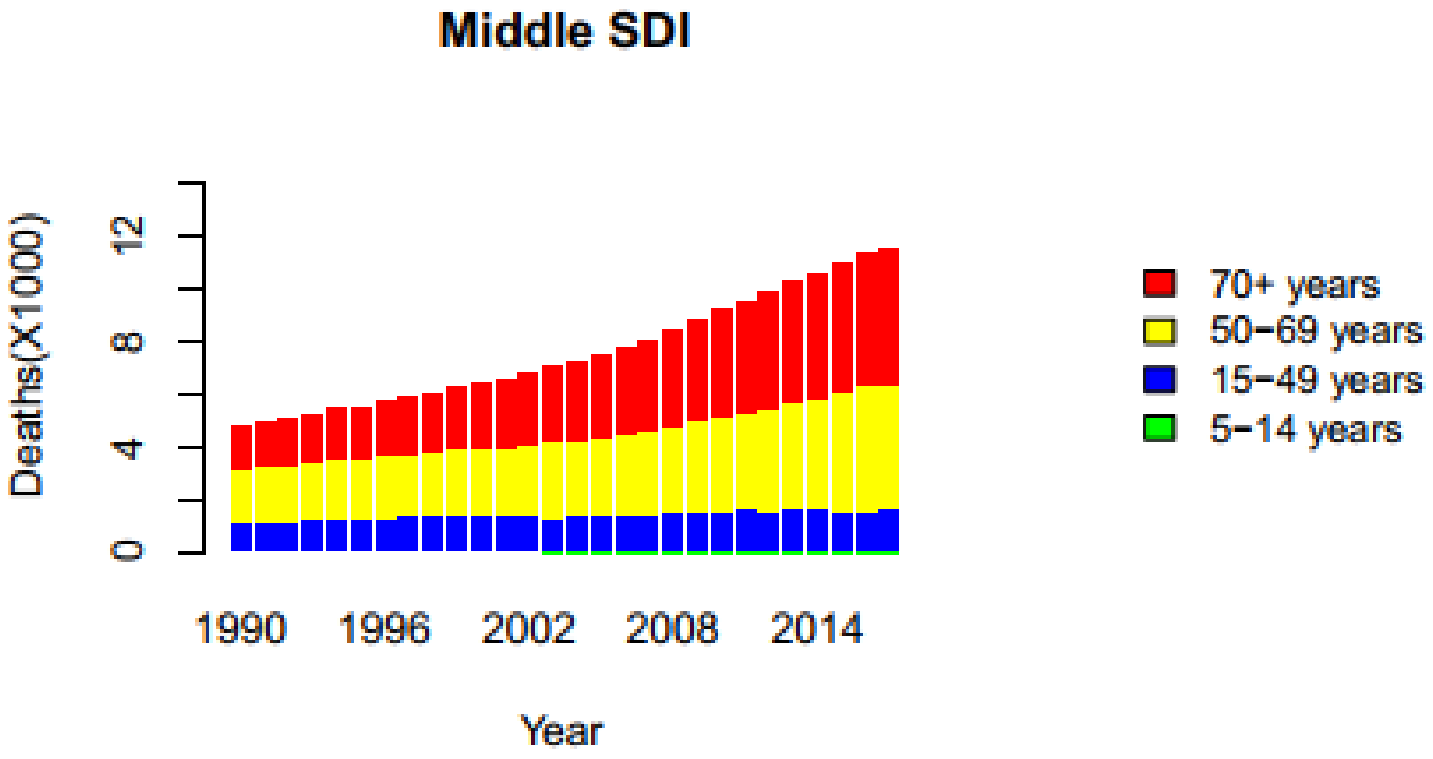

© 2020 Deng Y et al. *JAMA Network Open.*

eFigure 14.The age subgroups of thyroid cancer deaths among low-middle SDI quintiles over 28 years.

Figure legends: SDI, socio-demographic index.

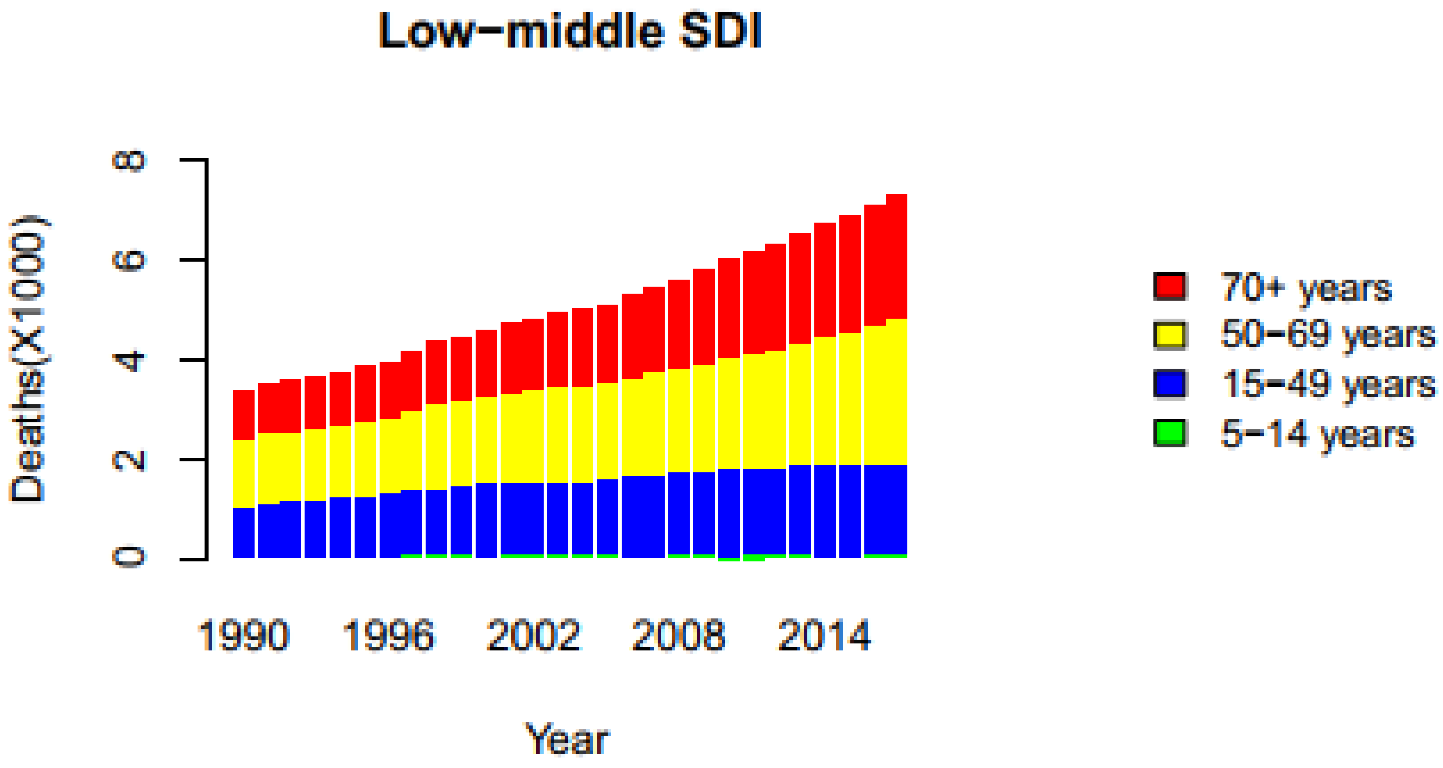

© 2020 Deng Y et al. *JAMA Network Open.*

eFigure 15.The age subgroups of thyroid cancer deaths among low SDI quintiles over 28 years.

Figure legends: SDI, socio-demographic index.

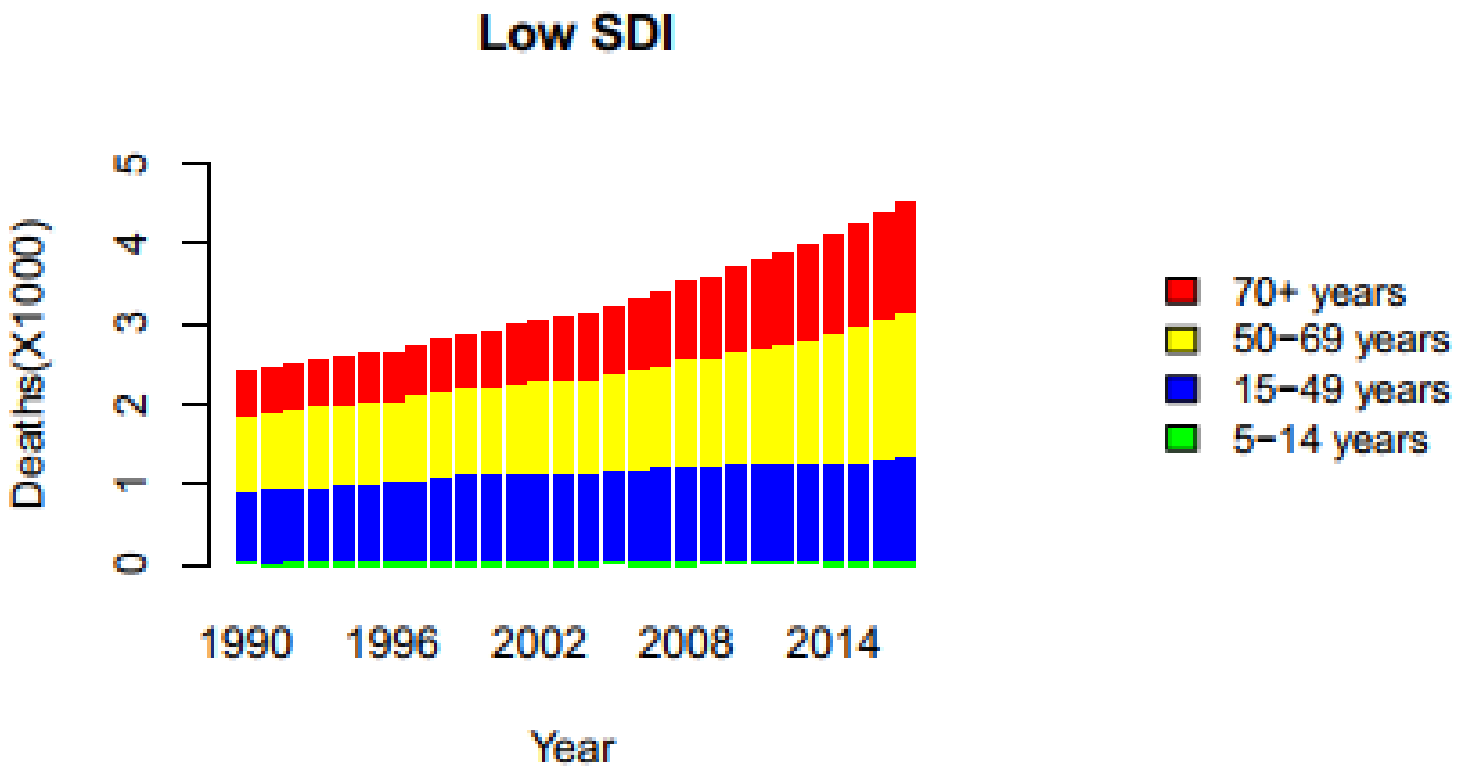

© 2020 Deng Y et al. *JAMA Network Open.*

eFigure 16. The change trends of thyroid cancer ASDR among both gender and SDI quintiles.

Figure legends: ASDR, age-standardized death rate; SDI, socio-demographic index.

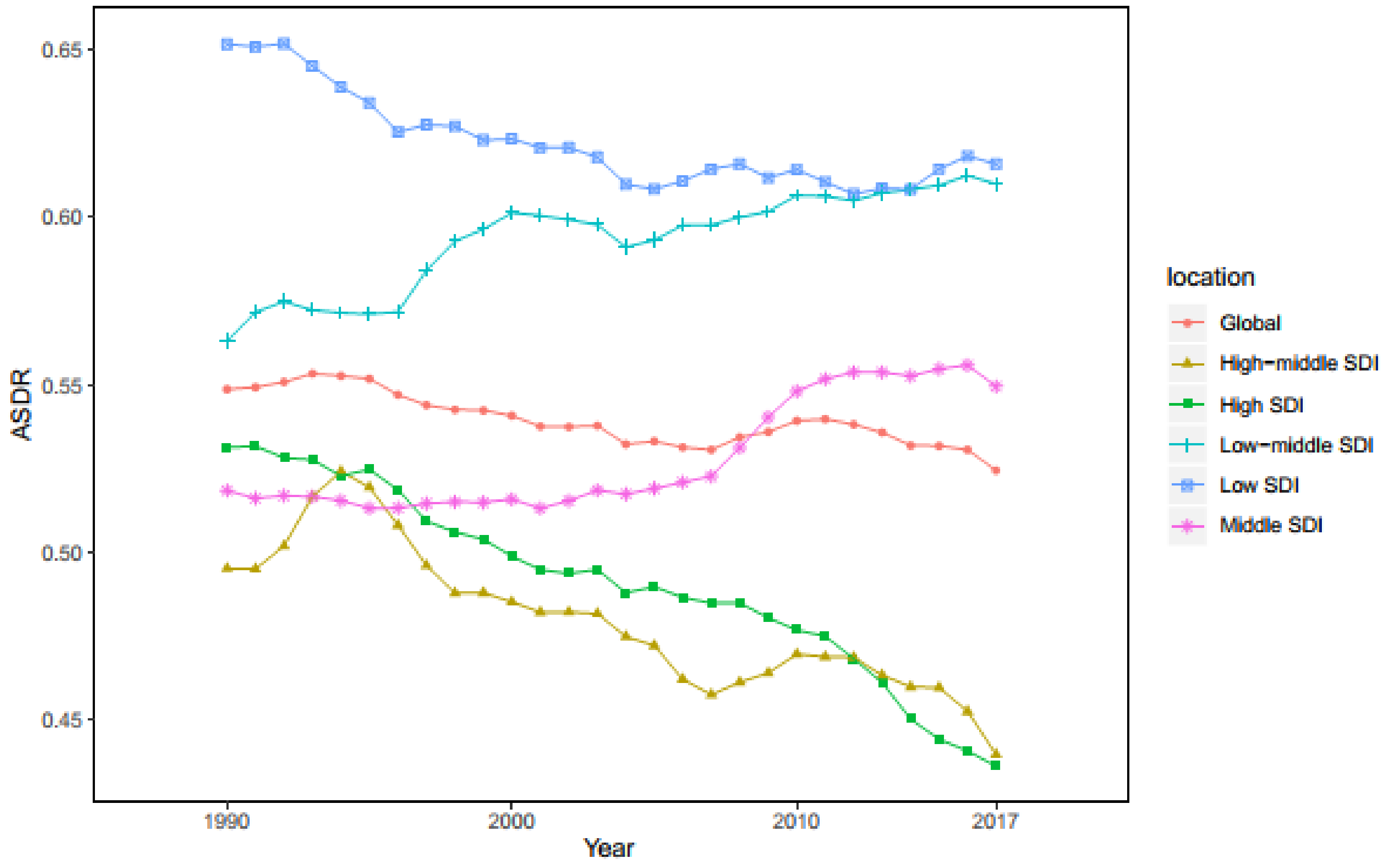

© 2020 Deng Y et al. *JAMA Network Open.*

eFigure 17. The change trends of thyroid cancer ASDR among females and SDI quintiles.

Figure legends: ASDR, age-standardized death rate; SDI, socio-demographic index.

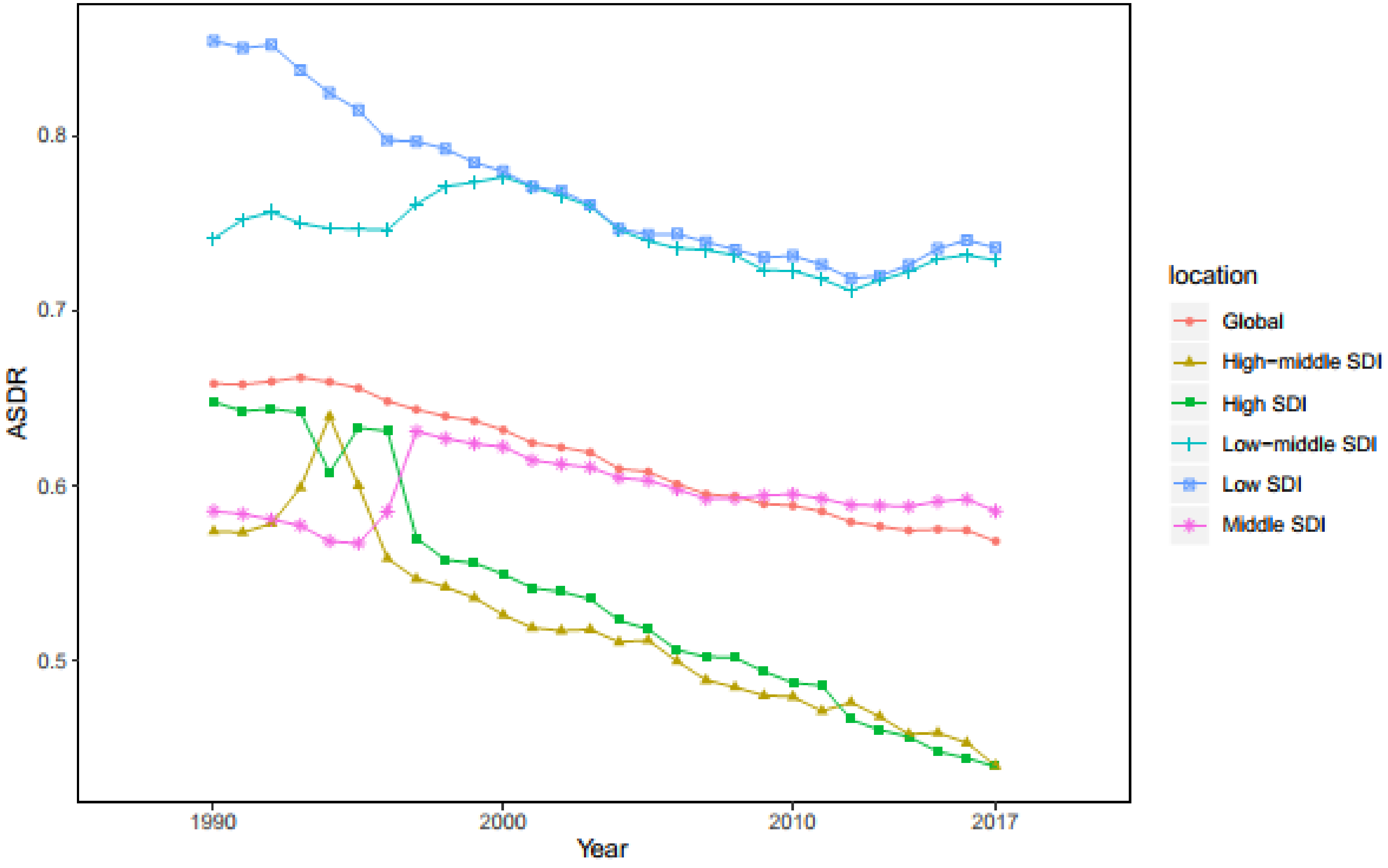

© 2020 Deng Y et al. *JAMA Network Open.*

eFigure 18. The change trends of thyroid cancer ASDR among males and SDI quintiles.

Figure legends: ASDR, age-standardized death rate; SDI, socio-demographic index.

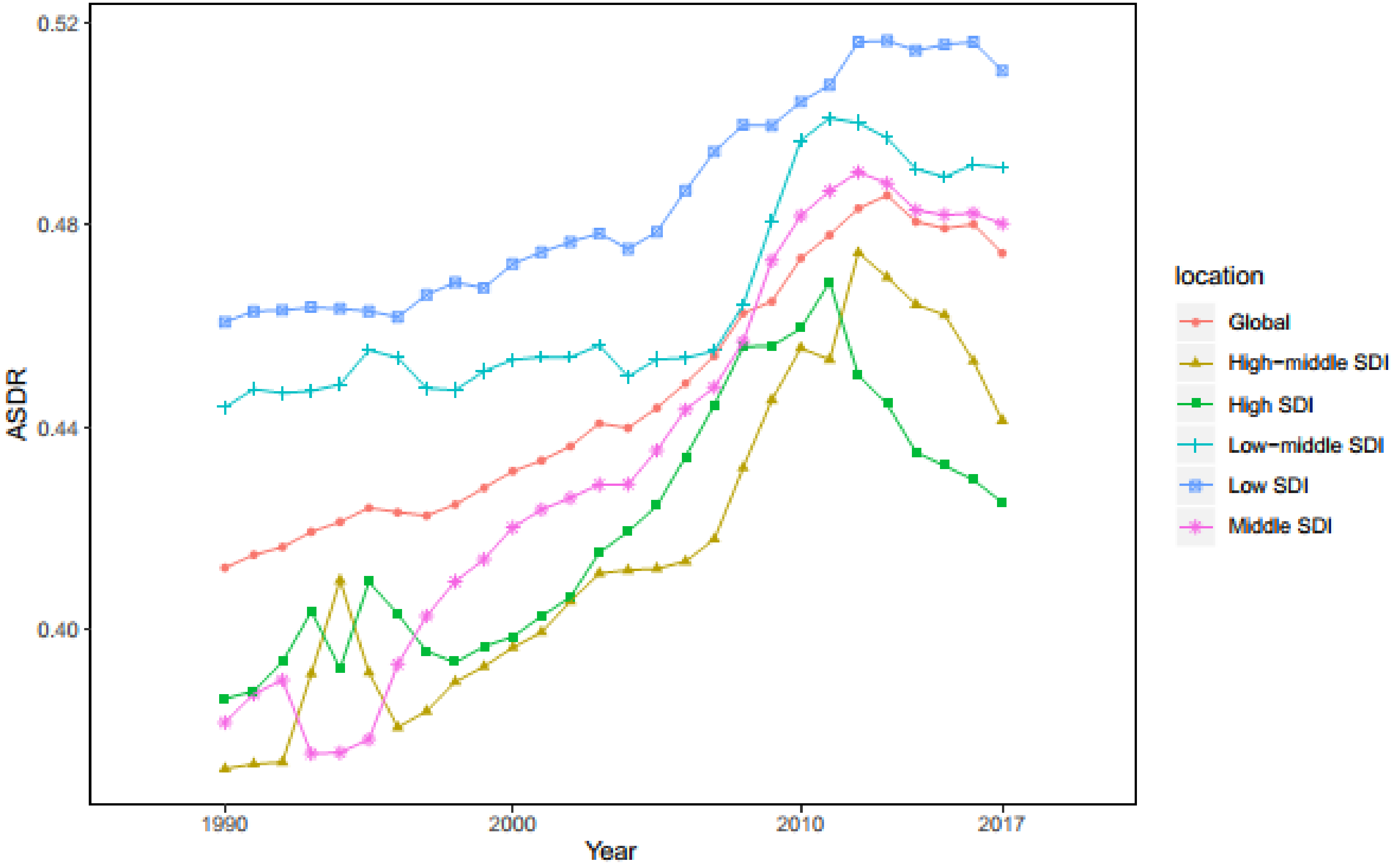

© 2020 Deng Y et al. *JAMA Network Open.*

eFigure 19. The proportion of different age subgroups in thyroid cancer incidence by years.

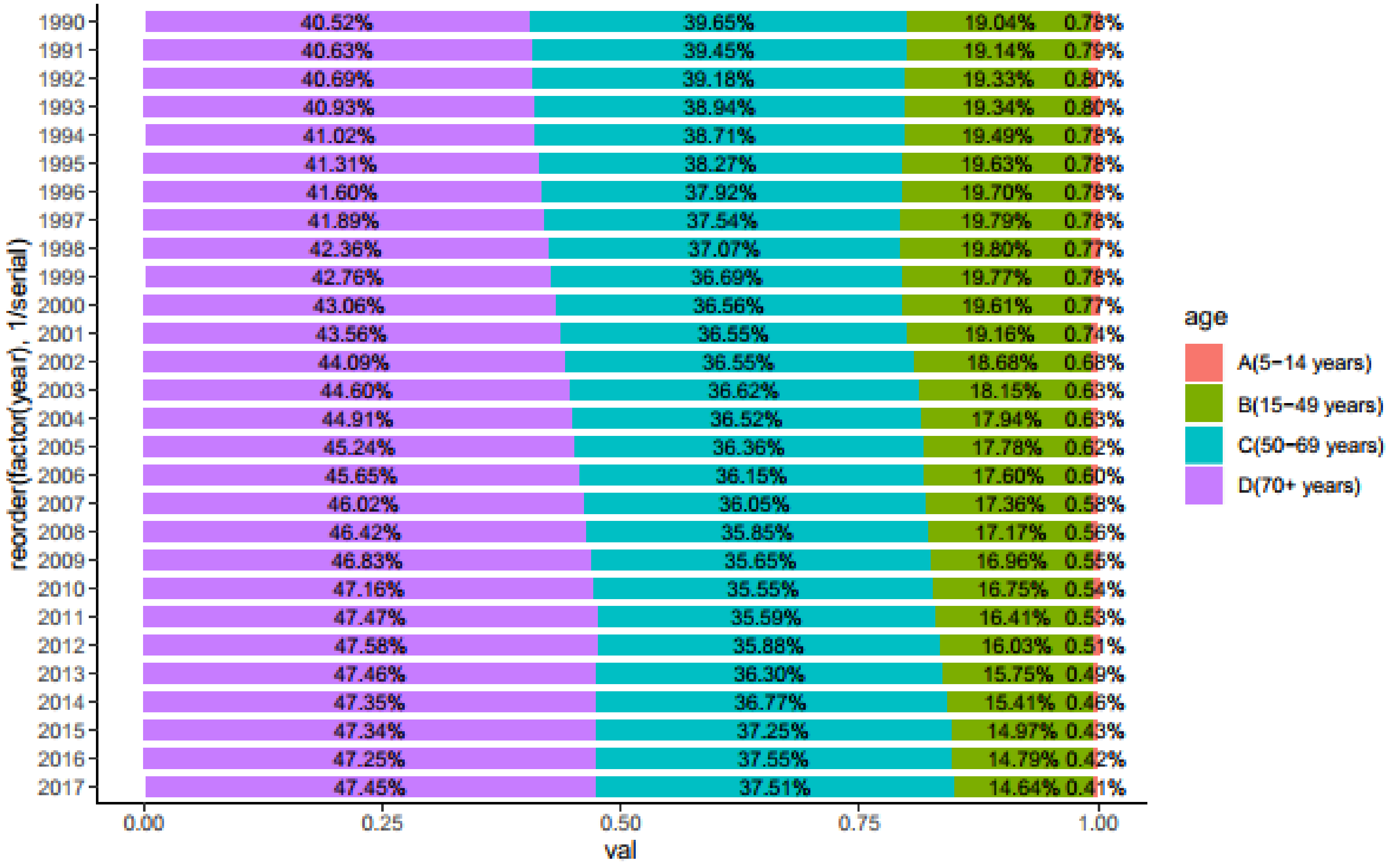

© 2020 Deng Y et al. *JAMA Network Open.*

eFigure 20. The correlation between EAPC of thyroid cancer DALYs and SDI in 2017.

Figure legends: The circles represent countries that were available on SDI data. The size of circle is increased with the cases of thyroid cancer. The ρ indices and P values presented in and were derived from Pearson correlation analysis. EAPC, estimated annual percentage change; SDI, socio-demographic index; DALY: disability adjusted life-year.

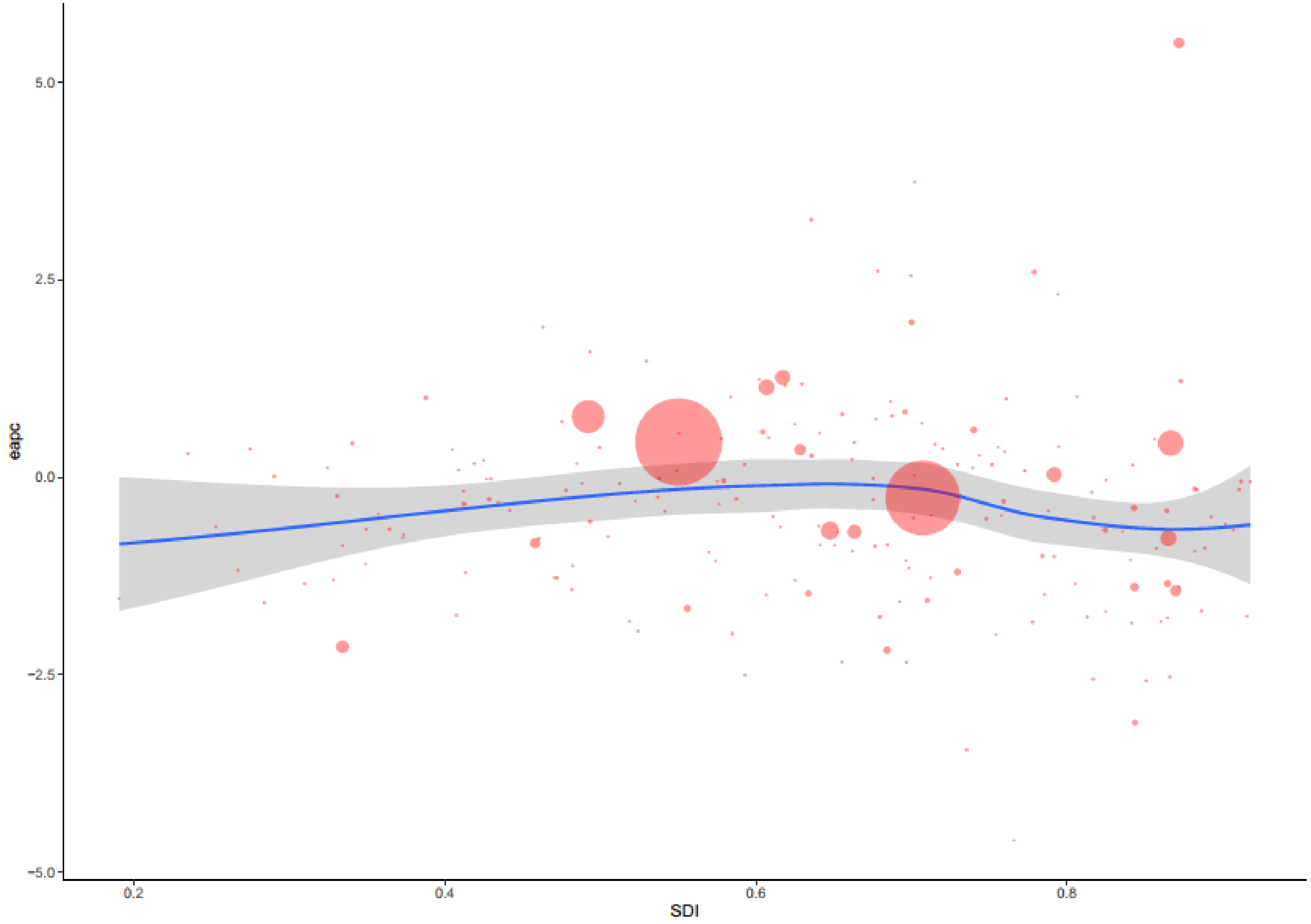

© 2020 Deng Y et al. *JAMA Network Open.*

eFigure 21. The change trends of thyroid cancer age-standardized DALY rate among both gender and SDI quintiles.

Figure legends: DALY: disability adjusted life-year; SDI, socio-demographic index.

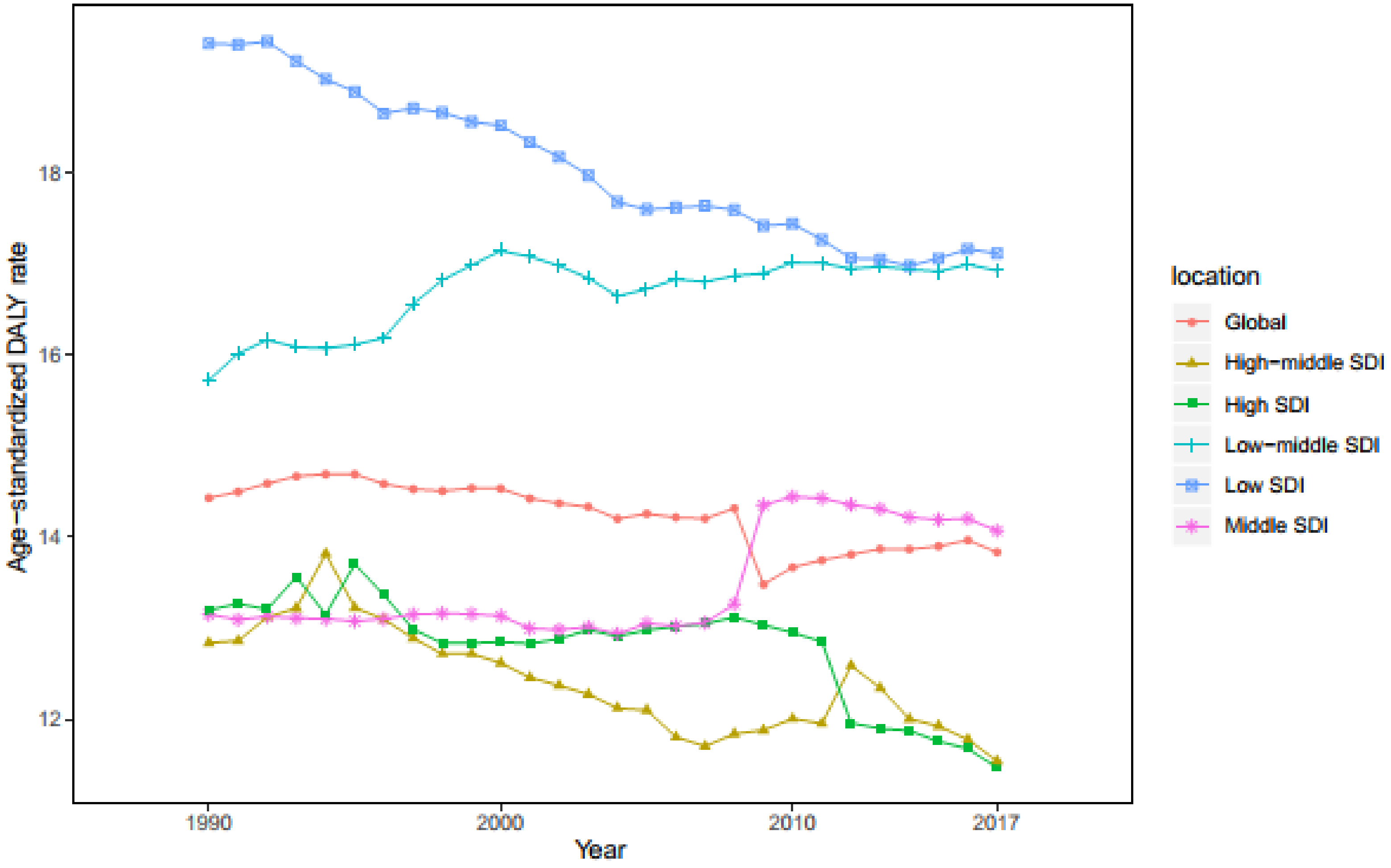

© 2020 Deng Y et al. *JAMA Network Open.*

eFigure 22. The change trends of thyroid cancer age-standardized DALY rate among females and SDI quintiles.

Figure legends: DALY: disability adjusted life-year; SDI, socio-demographic index.

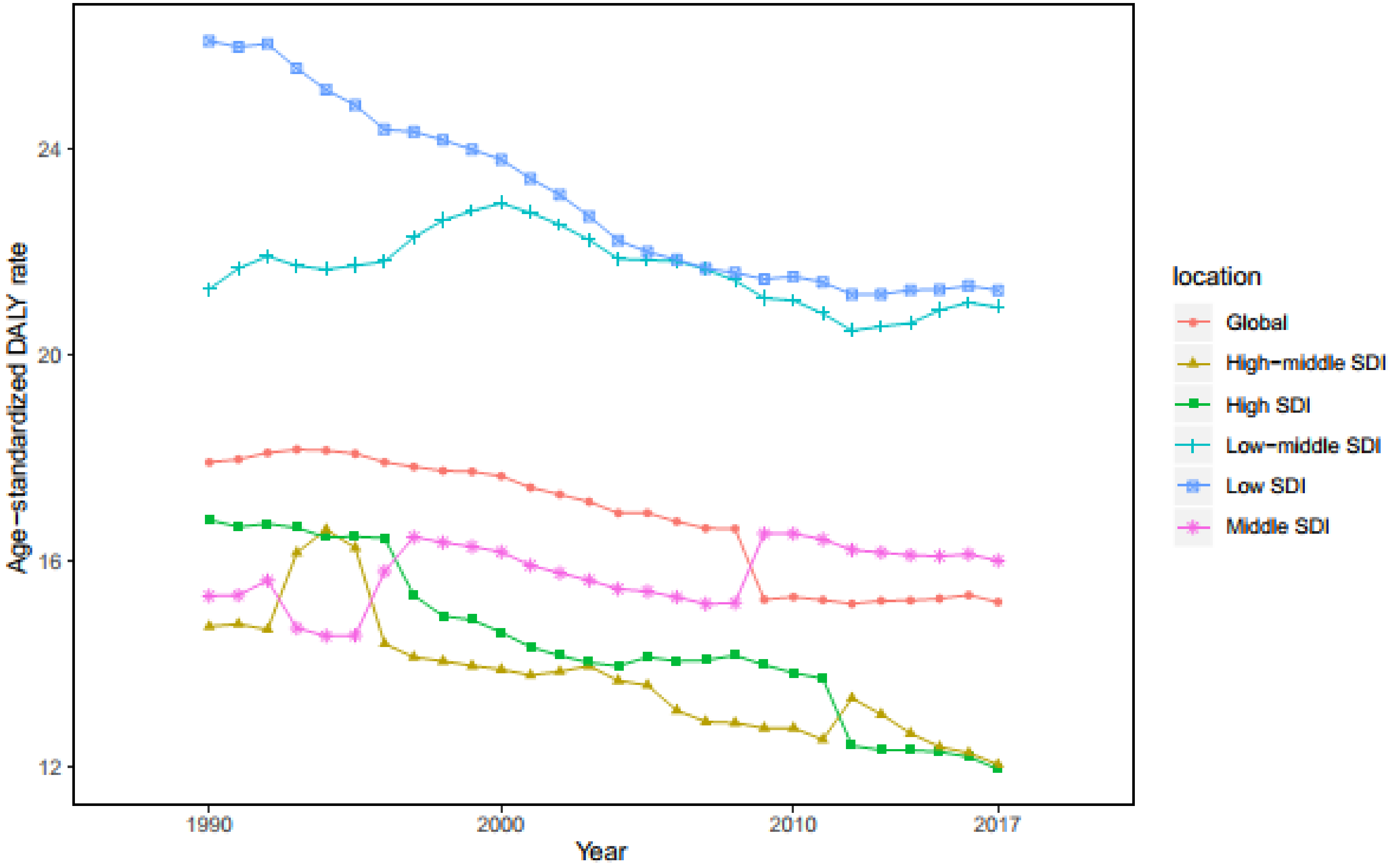

© 2020 Deng Y et al. *JAMA Network Open.*

eFigure 23. The change trends of thyroid cancer age-standardized DALY rate among males and SDI quintiles.

Figure legends: DALY: disability adjusted life-year; SDI, socio-demographic index.

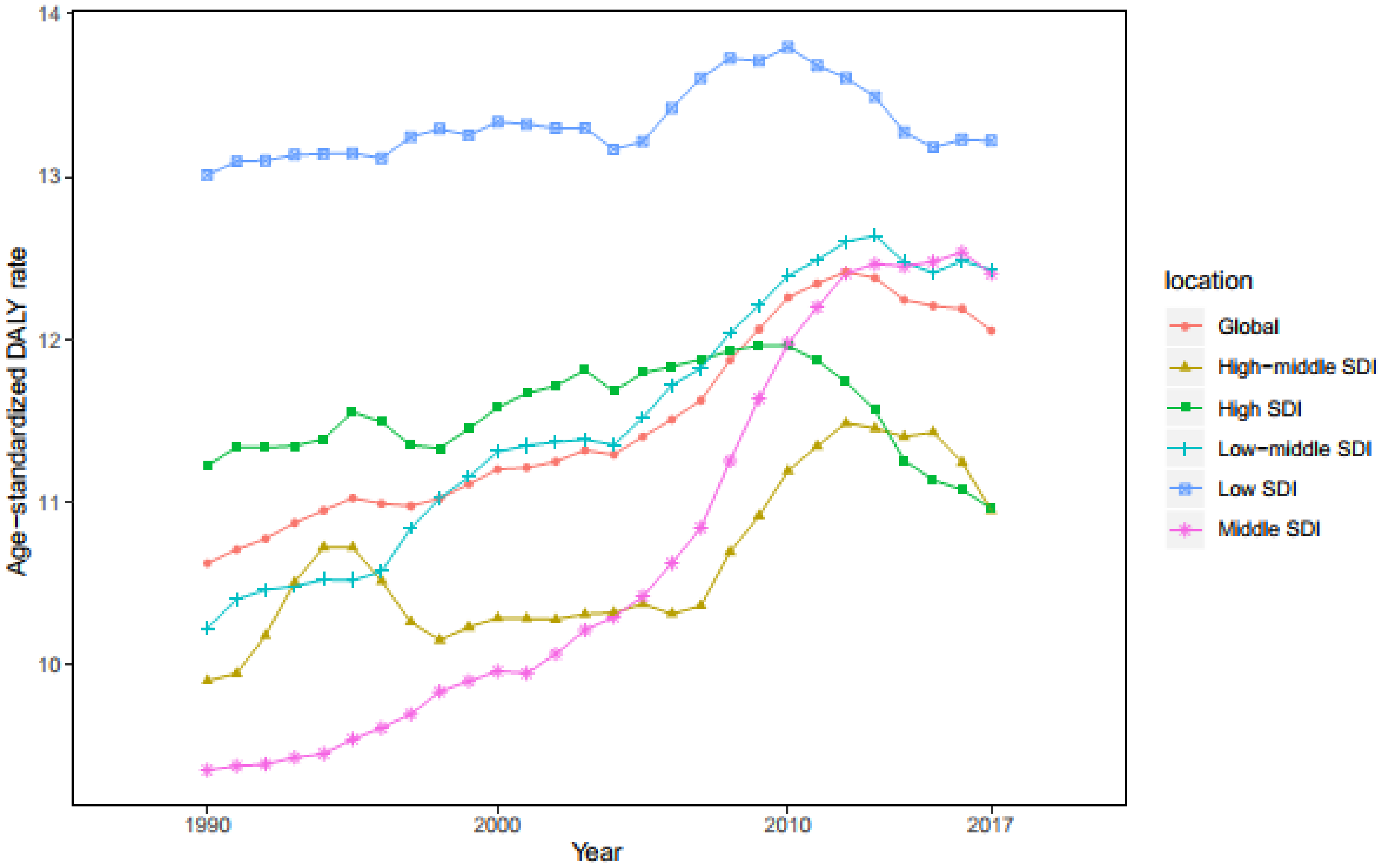

© 2020 Deng Y et al. *JAMA Network Open.*

eFigure 24. The age subgroups of thyroid cancer DALYs over 28 years worldwide.

Figure legends: DALY, disability adjusted life-year; SDI, socio-demographic index.

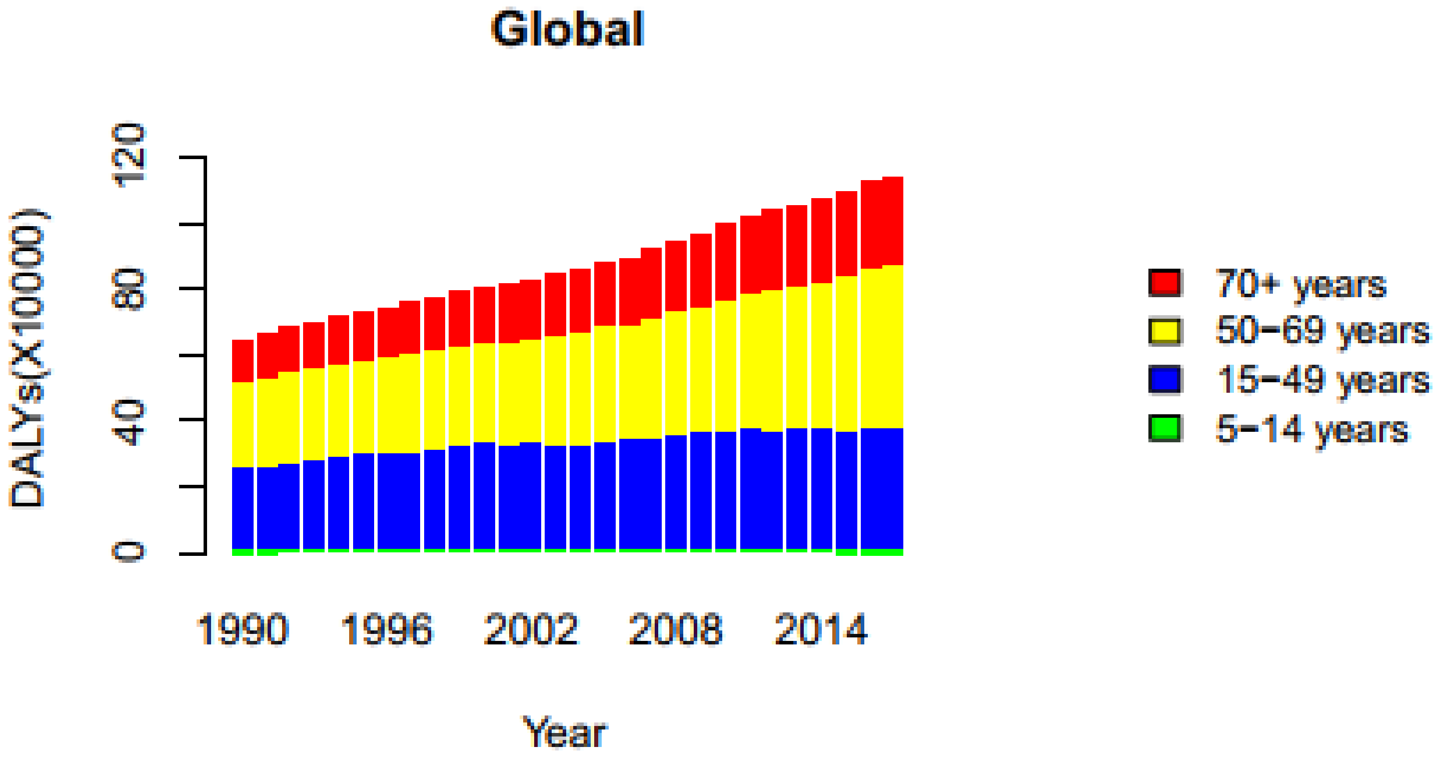

© 2020 Deng Y et al. *JAMA Network Open.*

eFigure 25.The age subgroups of thyroid cancer DALYs among high SDI quintiles over 28 years.

Figure legends: DALY, disability adjusted life-year; SDI, socio-demographic index.

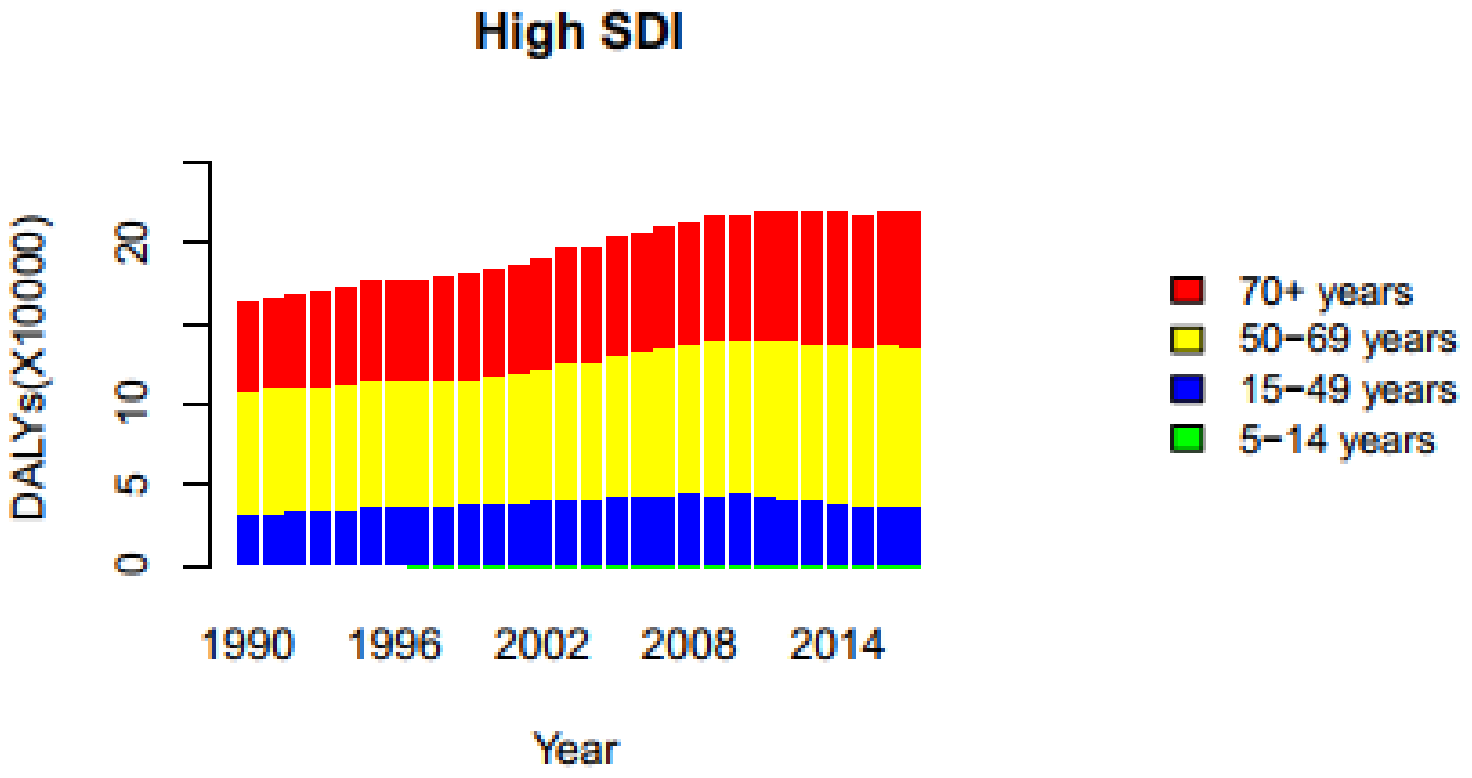

© 2020 Deng Y et al. *JAMA Network Open.*

eFigure 26.The age subgroups of thyroid cancer DALYs among high-middle SDI quintiles over 28 years.

Figure legends: DALY, disability adjusted life-year; SDI, socio-demographic index.

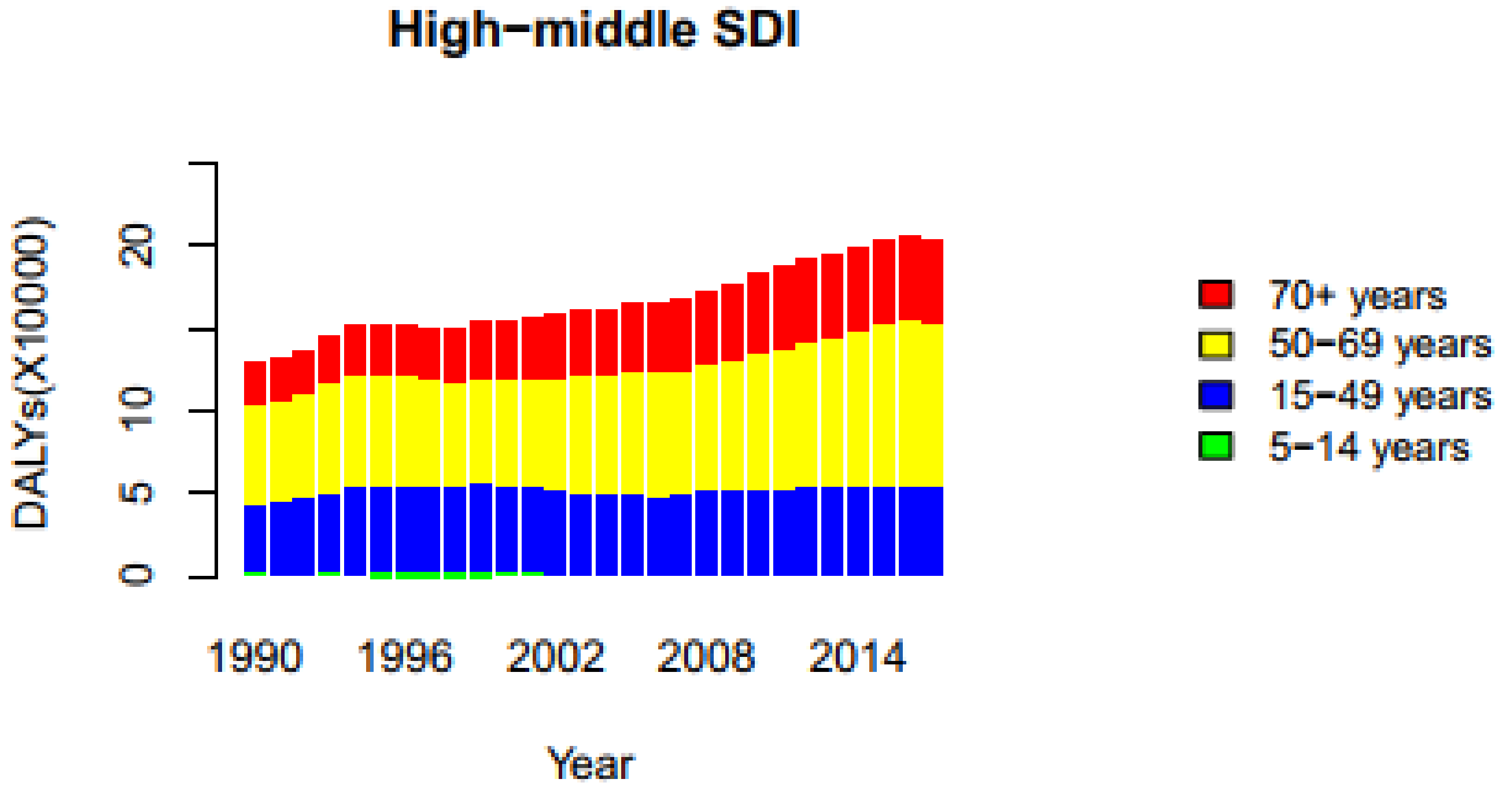

© 2020 Deng Y et al. *JAMA Network Open.*

eFigure 27.The age subgroups of thyroid cancer DALYs among middle SDI quintiles over 28 years.

Figure legends: DALY, disability adjusted life-year; SDI, socio-demographic index.

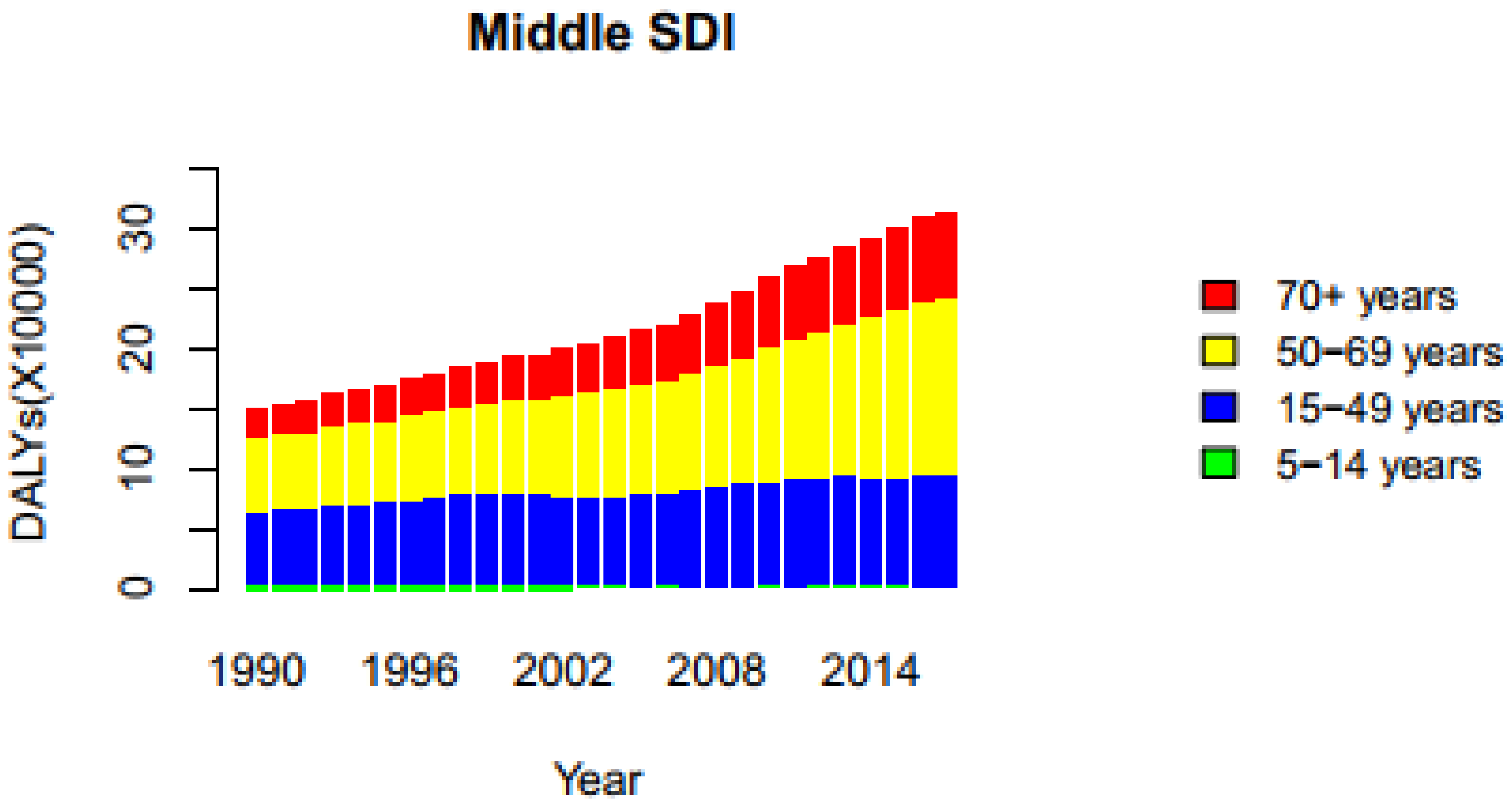

© 2020 Deng Y et al. *JAMA Network Open.*

eFigure 28.The age subgroups of thyroid cancer DALYs among low-middle SDI quintiles over 28 years.

Figure legends: DALY, disability adjusted life-year; SDI, socio-demographic index.

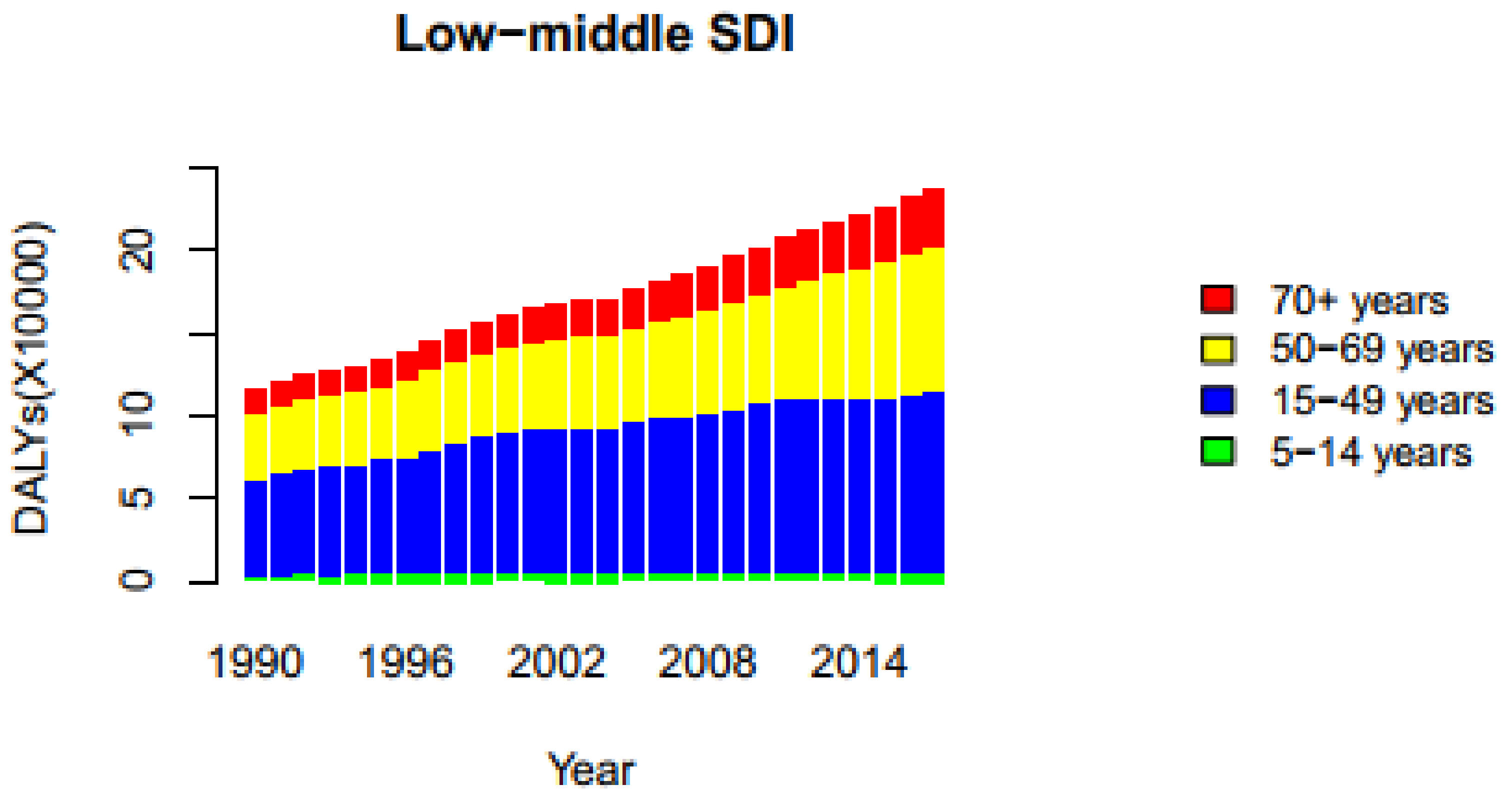

© 2020 Deng Y et al. *JAMA Network Open.*

eFigure 29.The age subgroups of thyroid cancer DALYs among low SDI quintiles over 28 years.

Figure legends: DALY, disability adjusted life-year; SDI, socio-demographic index.

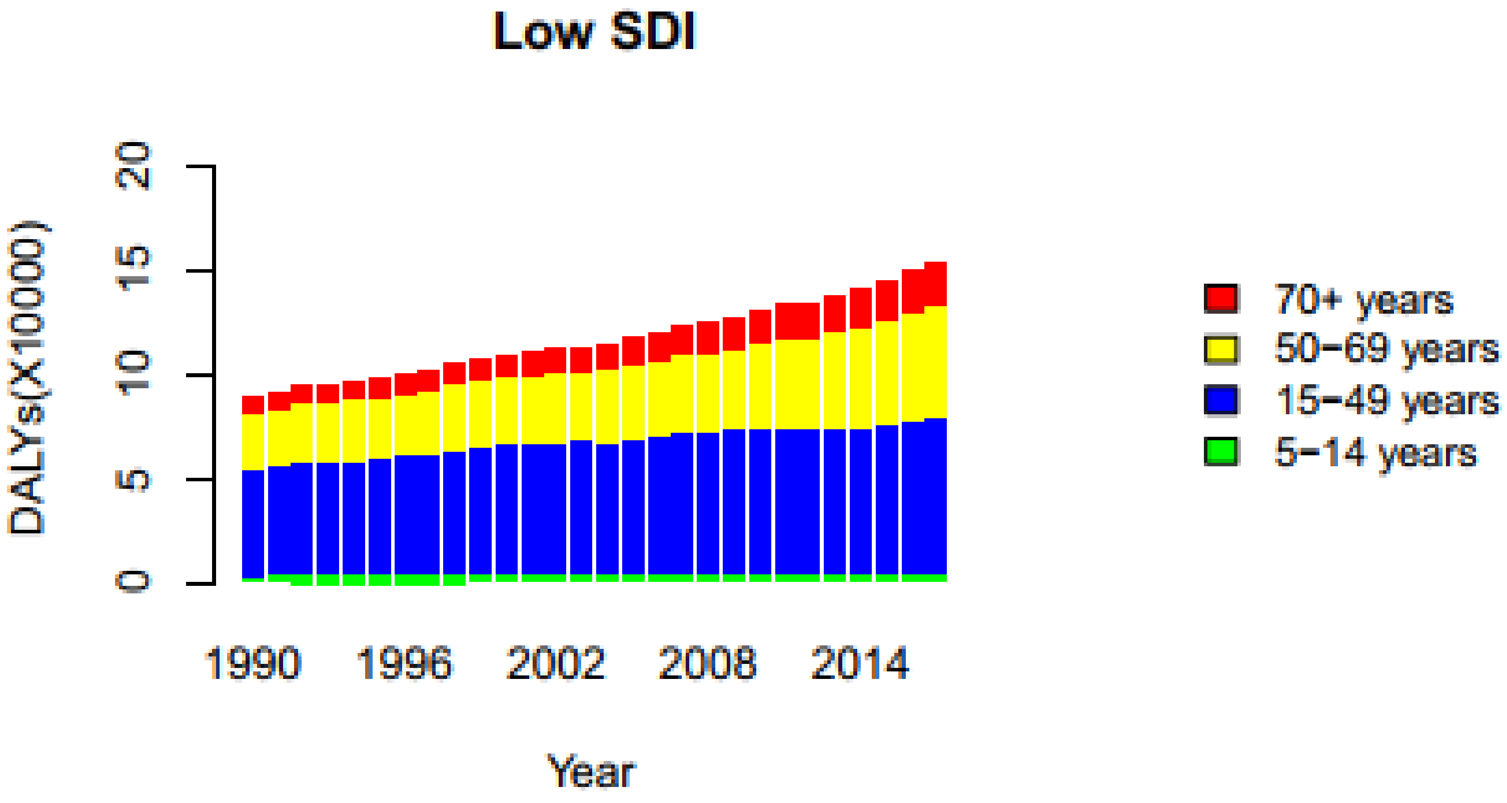

© 2020 Deng Y et al. *JAMA Network Open.*

eFigure 30. The proportion of different age subgroups in thyroid cancer DALYs by years.

Figure legends: DALY: disability adjusted life-year.

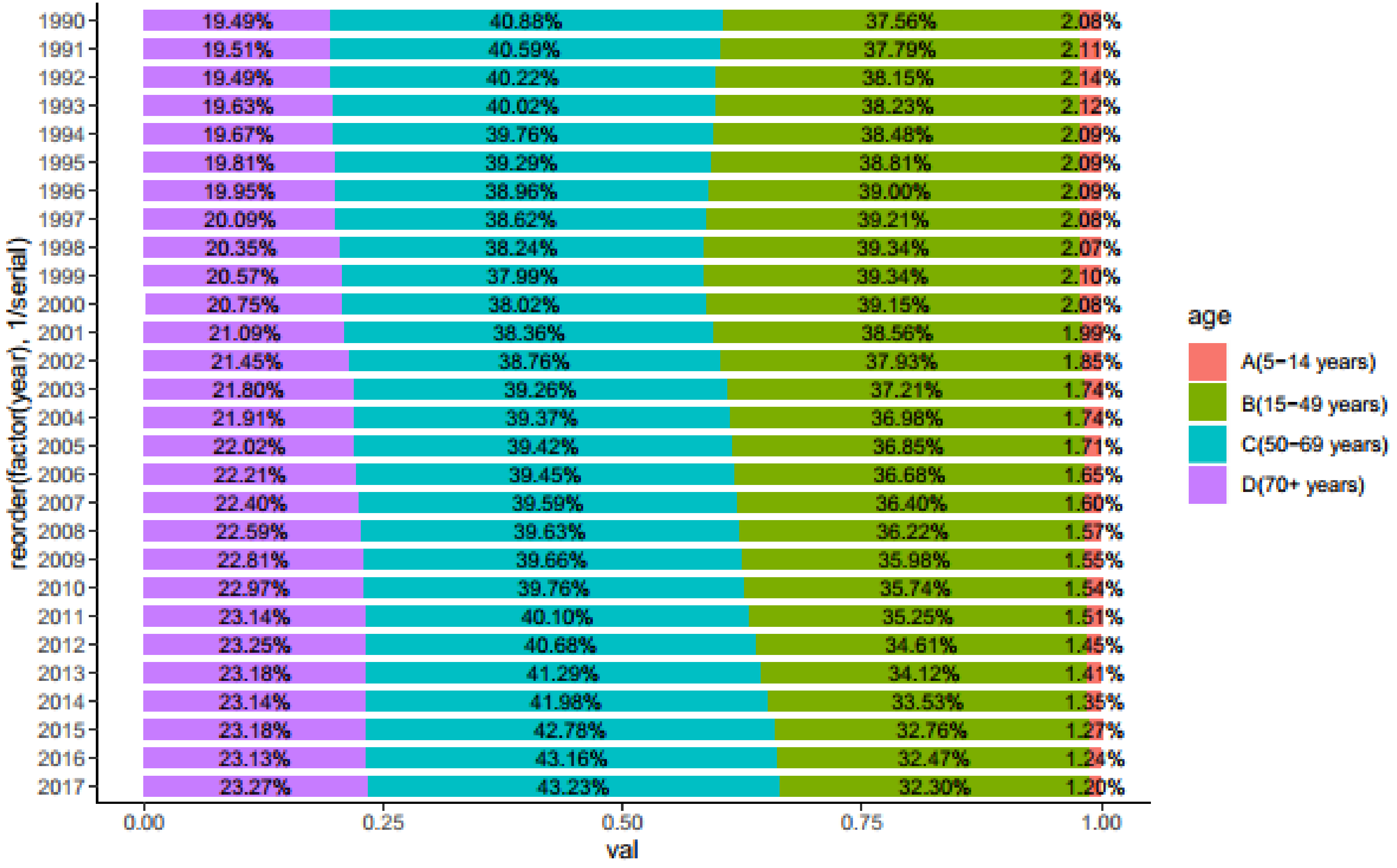

© 2020 Deng Y et al. *JAMA Network Open.*

eTable 1. The deaths from thyroid cancer and its temporal trends from 1990 to 2017.

Characteristics	1990				2017				1990-2017
	Death cases		ASDR (per 100,000)		Death cases		ASDR (per 100,000)		EAPC
	No. ×10³ (95% UI)	Female /male ratio	No. (95% UI)	Female /male ratio	No. ×10³ (95% UI)	Female/ male ratio	No. (95% UI)	Female /male ratio	No. (95% CI)
Global	22.07 (20.81 -24.22)	1.42	0.55 (0.52 - 0.60)	1.60	41.24 (39.91 - 44.14)	2.93	0.52 (0.51 - 0.56)	1.20	-0.15 (-0.19 - -0.12)
**Sex**									
Male	7.56 (7.22 - 8.15)	NA	0.41 (0.40 - 0.44)	NA	17.16 (16.41 - 17.77)	NA	0.47 (0.45 - 0.49)	NA	0.70 (0.61 - 0.78)
Female	14.51 (13.46 - 16.77)	NA	0.66 (0.61 - 0.76)	NA	24.08 (23.06 - 26.83)	NA	0.57 (0.54 - 0.63)	NA	-0.63 (-0.66 - -0.59)
**Socio-demographic index**									
High SDI	6.90 (6.81 - 6.99)	1.25	0.53 (0.52 - 0.54)	1.32	10.02 (9.71 - 10.38)	2.48	0.44 (0.42 - 0.45)	1.03	-0.68 (-0.74 - -0.62)
High-middle SDI	4.51 (4.29 - 4.72)	1.20	0.5 (0.47 - 0.52)	1.48	7.66 (7.34 - 8.05)	3.42	0.44 (0.42 - 0.46)	1.00	-0.48 (-0.58 - -0.39)
Low SDI	2.40 (1.81 – 3.00)	1.69	0.65 (0.52 - 0.80)	1.85	4.53 (4.06 - 5.01)	3.32	0.62 (0.55 - 0.68)	1.50	-0.23 (-0.28 - -0.17)
Low-middle SDI	3.33 (2.96 - 3.95)	1.89	0.56 (0.51 - 0.67)	1.94	7.27 (6.77 - 8.22)	3.74	0.61 (0.57 - 0.69)	1.52	0.28 (0.23 - 0.33)
Middle SDI	4.85 (4.57 - 5.73)	1.30	0.52 (0.49 - 0.62)	1.74	11.61 (11.04 - 12.89)	3.23	0.55 (0.52 - 0.61)	1.15	0.33 (0.25 - 0.41)
**Region**									
Andean Latin America	0.16 (0.15 - 0.18)	2.20	0.78 (0.70 - 0.86)	2.29	0.53 (0.46 - 0.58)	3.78	0.99 (0.86 - 1.09)	2.18	1.08 (0.85 - 1.31)
Australasia	0.09 (0.09 - 0.09)	1.23	0.37 (0.36 - 0.39)	1.22	0.19 (0.17 - 0.21)	1.90	0.40 (0.36 - 0.44)	1.05	0.71 (0.51 - 0.91)
Caribbean	0.14 (0.13 - 0.15)	1.47	0.52 (0.48 - 0.57)	1.71	0.27 (0.25 - 0.29)	3.31	0.53 (0.48 - 0.58)	1.29	0.10 (-0.10 - 0.30)
Central Asia	0.21 (0.20 - 0.24)	1.60	0.44 (0.41 - 0.50)	1.29	0.26 (0.25 - 0.28)	3.38	0.37 (0.35 - 0.39)	1.21	-1.11 (-1.40 - -0.83)
Central Europe	1.07 (1.04 - 1.09)	1.62	0.71 (0.69 - 0.73)	1.33	0.92 (0.88 - 0.96)	3.25	0.43 (0.41 - 0.45)	1.27	-2.15 (-2.35 - -1.96)
Central Latin America	0.61 (0.59 - 0.63)	2.00	0.73 (0.71 - 0.75)	2.01	1.56 (1.50 - 1.64)	3.68	0.69 (0.66 - 0.72)	1.77	-0.35 (-0.51 - -0.20)
Central Sub-	0.1 (0.08 - 0.13)	1.64	0.44 (0.37 - 0.55)	1.79	0.19 (0.15 - 0.25)	3.13	0.39 (0.30 - 0.51)	1.50	-0.56 (-0.63 - -0.49)

© 2020 Deng Y et al. *JAMA Network Open.*

Characteristics	1990				2017				1990-2017
	Death cases		ASDR (per 100,000)		Death cases		ASDR (per 100,000)		EAPC
	No. ×10^3 (95% UI)	Female /male ratio	No. (95% UI)	Female /male ratio	No. ×10^3 (95% UI)	Female/ male ratio	No. (95% UI)	Female /male ratio	No. (95% CI)
Saharan Africa									
East Asia	3.32 (3.10 - 3.87)	0.79	0.38 (0.36 - 0.45)	1.47	7.25 (6.83 - 7.92)	3.18	0.38 (0.35 - 0.41)	0.66	0.23 (-0.04 - 0.50)
Eastern Europe	1.34 (1.29 - 1.45)	1.84	0.48 (0.46 - 0.52)	1.30	1.66 (1.61 - 1.73)	3.43	0.49 (0.47 - 0.51)	1.28	-0.23 (-0.63 - 0.17)
Eastern Sub-Saharan Africa	0.88 (0.63 - 1.16)	1.37	0.98 (0.73 - 1.25)	1.75	1.41 (1.21 - 1.64)	3.18	0.80 (0.69 - 0.92)	1.11	-0.95 (-1.05 - -0.86)
High-income Asia Pacific	1.14 (1.12 - 1.16)	1.53	0.58 (0.57 - 0.59)	1.47	2.77 (2.65 - 2.94)	3.67	0.57 (0.55 - 0.61)	1.19	0.29 (0.01 - 0.56)
High-income North America	1.30 (1.27 - 1.32)	1.09	0.36 (0.35 - 0.37)	1.02	2.38 (2.30 - 2.45)	1.77	0.39 (0.37 - 0.40)	0.90	0.29 (0.20 - 0.38)
North Africa and Middle East	0.86 (0.72 - 1.07)	1.37	0.49 (0.42 - 0.63)	1.75	1.92 (1.80 - 2.28)	3.77	0.46 (0.43 - 0.55)	1.30	-0.15 (-0.23 - -0.08)
Oceania	0.02 (0.02 - 0.03)	1.46	0.85 (0.71 - 1.01)	2.19	0.05 (0.04 - 0.06)	2.62	0.83 (0.69 - 0.99)	1.99	-0.02 (-0.05 - 0.02)
South Asia	3.63 (3.14 - 4.39)	1.90	0.56 (0.50 - 0.69)	1.93	8.93 (8.18 - 9.84)	3.66	0.65 (0.59 - 0.71)	1.63	0.56 (0.48 - 0.64)
Southeast Asia	2.33 (2.01 - 2.74)	1.79	0.91 (0.79 - 1.09)	2.13	4.82 (4.46 - 5.70)	3.72	0.86 (0.79 - 1.02)	1.58	-0.16 (-0.24 - -0.08)
Southern Latin America	0.32 (0.31 - 0.33)	1.54	0.68 (0.65 - 0.71)	1.43	0.43 (0.40 - 0.47)	2.94	0.52 (0.48 - 0.57)	1.28	-1.12 (-1.30 - -0.93)
Southern Sub-Saharan Africa	0.11 (0.10 - 0.13)	1.65	0.40 (0.35 - 0.46)	1.63	0.22 (0.20 - 0.24)	3.77	0.40 (0.37 - 0.45)	1.37	0.09 (-0.43 - 0.60)
Tropical Latin America	0.53 (0.52 - 0.55)	1.71	0.60 (0.58 - 0.62)	1.52	1.13 (1.09 - 1.16)	1.37	0.50 (0.48 - 0.51)	1.09	-0.68 (-0.75 - -0.62)
Western Europe	3.62 (3.56 - 3.69)	1.14	0.60 (0.59 - 0.61)	1.36	3.86 (3.68 - 4.05)	2.60	0.41 (0.40 - 0.44)	0.95	-1.45 (-1.49 - -1.41)
Western Sub-Saharan Africa	0.27 (0.21 - 0.32)	1.36	0.31 (0.24 - 0.37)	1.57	0.48 (0.41 - 0.55)	2.51	0.28 (0.23 - 0.32)	1.17	-0.55 (-0.62 - -0.48)

Abbreviations: ASDR, age standardized death rate; EAPC, estimated annual percentage change; CI, confidence interval; UI, uncertainty interval; SDI, socio-demographic index

© 2020 Deng Y et al. *JAMA Network Open.*

eTable 2. The disability adjusted life-years of thyroid cancer and its temporal trends from 1990 to 2017.

Characteristics	1990			2017			1990-2017
	DALY	Age standardized DALY Rate (per 100,000)		DALY	Age standardized DALY Rate (per 100,000)		EAPC
	No. ×10^3 (95% UI)	No. (95% UI)	Female/male ratio	No. ×10^3 (95% UI)	No. (95% UI)	Female/male ratio	No. (95% CI)
Global	648.24 (595.58 - 713.24)	14.44 (13.35 - 15.85)	1.69	1,133.17 (1,073.44 - 1,227.49)	14.08 (13.34 - 15.27)	1.33	-0.11 (-0.15 - -0.08)
**Sex**							
Male	226.93 (213.37 - 247.06)	10.63 (10.05 - 11.51)	NA	468.25 (444.42 - 492.88)	12.06 (11.45 - 12.69)	NA	0.59 (0.52 - 0.66)
Female	421.31 (375.69 - 489.01)	17.94 (16.11 - 20.85)	NA	664.93 (618.50 - 746.73)	16.03 (14.89 - 17.98)	NA	-0.53 (-0.57 - -0.48)
**Socio-demographic index**							
High SDI	162.78 (155.31 - 171.62)	13.21 (12.57 - 13.97)	1.31	220.44 (205.12 - 239.51)	11.55 (10.66 - 12.69)	1.10	-0.36 (-0.48 - -0.24)
High-middle SDI	129.18 (119.94 - 136.17)	12.85 (11.99 - 13.52)	1.55	204.21 (190.73 - 220.65)	11.48 (10.71 - 12.41)	1.09	-0.56 (-0.68 - -0.43)
Low SDI	89.84 (63.65 - 114.09)	19.42 (14.41 - 24.36)	2.01	153.32 (136.63 - 169.90)	17.12 (15.29 - 18.87)	1.58	-0.54 (-0.58 - -0.50)
Low-middle SDI	115.39 (100.21 - 134.86)	15.73 (13.88 - 18.52)	2.08	236.77 (214.78 - 270.13)	16.93 (15.58 - 19.25)	1.71	0.23 (0.15 - 0.3)
Middle SDI	148.92 (139.53 - 172.28)	13.16 (12.37 - 15.41)	1.80	314.77 (294.66 - 346.94)	13.84 (12.97 - 15.26)	1.23	0.25 (0.16 - 0.33)
**Region**							
Andean Latin America	4.49 (4.03 - 5.07)	18.87 (16.94 - 21.14)	2.22	12.82 (11.19 - 14.27)	23.25 (20.24 - 25.86)	2.11	0.95 (0.71 - 1.20)
Australasia	2.28 (2.14 - 2.45)	9.77 (9.13 - 10.51)	1.25	4.77 (4.17 - 5.44)	11.47 (10.01 - 13.17)	1.14	1.03 (0.85 - 1.21)
Caribbean	3.8 (3.41 - 4.23)	13.51 (12.18 - 14.99)	1.75	7.07 (6.36 - 7.87)	13.94 (12.55 - 15.53)	1.35	0.17 (-0.04 - 0.38)
Central Asia	6.63 (6.17 - 7.50)	12.55 (11.68 - 14.27)	1.33	7.63 (7.09 - 8.23)	9.47 (8.83 - 10.15)	1.32	-1.59 (-1.88 - -1.30)
Central Europe	27.87 (26.83 - 29.07)	18.55 (17.84 - 19.35)	1.31	22.23 (20.81 - 23.89)	11.69 (10.91 - 12.62)	1.35	-2.03 (-2.24 - -1.83)
Central Latin America	16.48 (15.91 - 17.09)	17.14 (16.55 - 17.74)	1.97	39.54 (37.35 - 42.02)	16.53 (15.62 - 17.55)	1.77	-0.27 (-0.43 - -0.11)
Central Sub-	3.14 (2.43 - 4.07)	11.04 (8.92 - 14.06)	1.80	5.74 (4.64 - 7.68)	9.34 (7.39 - 12.49)	1.49	-0.71 (-0.79 - -0.64)

© 2020 Deng Y et al. *JAMA Network Open.*

Characteristics	1990			2017			1990-2017
	DALY	Age standardized DALY Rate (per 100,000)		DALY	Age standardized DALY Rate (per 100,000)		EAPC
	No. ×10^3 (95% UI)	No. (95% UI)	Female/male ratio	No. ×10^3 (95% UI)	No. (95% UI)	Female/male ratio	No. (95% CI)
Saharan Africa							
East Asia	104.01 (96.07 - 117.48)	9.90 (9.19 - 11.27)	1.65	184.14 (170.57 - 203.49)	9.15 (8.46 - 10.14)	0.76	-0.26 (-0.57 - 0.05)
Eastern Europe	35.79 (33.65 - 39.61)	12.76 (11.98 - 14.12)	1.27	44.72 (41.69 - 48.39)	14.36 (13.32 - 15.61)	1.30	0.14 (-0.25 - 0.54)
Eastern Sub-Saharan Africa	34.05 (22.78 - 45.82)	29.67 (21.03 - 39.14)	1.82	52.65 (44.75 - 61.71)	22.39 (19.25 - 25.97)	1.18	-1.30 (-1.42 - -1.17)
High-income Asia Pacific	25.92 (24.64 - 27.5)	12.55 (11.93 - 13.32)	1.52	52.27 (47.68 - 57.63)	14.71 (13.12 - 16.70)	1.34	1.45 (0.99 - 1.91)
High-income North America	33.04 (30.97 - 35.48)	9.82 (9.19 - 10.58)	1.04	60.84 (56.16 - 66.36)	11.06 (10.15 - 12.16)	0.98	0.37 (0.25 - 0.50)
North Africa and Middle East	27.73 (22.7 - 33.94)	12.99 (10.72 - 16.11)	1.79	63.08 (57.86 - 72.30)	12.8 (11.8 - 14.83)	1.43	0.06 (-0.01 - 0.13)
Oceania	0.77 (0.64 - 0.96)	21.01 (17.65 - 25.63)	1.96	1.67 (1.36 - 2.13)	20.67 (16.98 - 25.45)	1.75	-0.02 (-0.09 - 0.05)
South Asia	135.98 (114.47 - 164.22)	16.56 (14.26 - 19.95)	2.12	297.71 (269.76 - 332.09)	18.77 (17.12 - 20.83)	1.82	0.46 (0.37 - 0.55)
Southeast Asia	71.02 (60.63 - 81.06)	23.02 (19.76 - 26.81)	2.15	134.52 (122.89 - 156.20)	21.28 (19.48 - 24.79)	1.61	-0.27 (-0.33 - -0.21)
Southern Latin America	8.00 (7.63 - 8.36)	16.70 (15.91 - 17.46)	1.39	10.04 (9.12 - 11.10)	12.78 (11.61 - 14.15)	1.29	-1.13 (-1.34 - -0.91)
Southern Sub-Saharan Africa	3.50 (3.08 - 3.84)	10.46 (9.27 - 11.61)	1.67	6.32 (5.74 - 7.01)	10.16 (9.25 - 11.27)	1.35	-0.10 (-0.71 - 0.51)
Tropical Latin America	14.83 (14.29 - 15.48)	14.26 (13.76 - 14.84)	1.46	28.57 (27.18 - 30.04)	12.05 (11.48 - 12.65)	1.07	-0.64 (-0.73 - -0.55)
Western Europe	80.85 (77.15 - 84.69)	14.80 (14.09 - 15.60)	1.32	82.73 (76.83 - 90.09)	10.97 (10.09 - 12.09)	0.99	-1.12 (-1.21 - -1.04)

© 2020 Deng Y et al. *JAMA Network Open.*

Characteristics	1990			2017			1990-2017
	DALY	Age standardized DALY Rate (per 100,000)		DALY	Age standardized DALY Rate (per 100,000)		EAPC
	No. ×10^3 (95% UI)	No. (95% UI)	Female/male ratio	No. ×10^3 (95% UI)	No. (95% UI)	Female/male ratio	No. (95% CI)
Western Sub-Saharan Africa	8.05 (6.31 - 9.21)	7.45 (5.84 - 8.65)	1.64	14.10 (12.27 - 16.32)	6.41 (5.54 - 7.45)	1.20	-0.7 (-0.79 - -0.62)

Abbreviations: DALY, disability adjusted life-year; EAPC, estimated annual percentage change; CI, confidence interval; UI, uncertainty interval; SDI, socio-demographic index

© 2020 Deng Y et al. *JAMA Network Open.*

eTable3. The incidence of thyroid cancer and its temporal trends from 1990 to 2017 among 195 countries.

Sex	Location	Incident cases						Age-standardized incidence rate (per 100,000)						EAPC		
		1990(95%UI)			2017(95%UI)			1990(95%UI)			2017(95%UI)			1990-2017(95%CI)		
		Cases (No.)	UL	LL	Cases (No.)	UL	LL	95%UI	UL	LL	95%UI	UL	LL	EAPC	LL	UL
Both	Afghanistan	109.40	190.52	42.53	313.22	507.53	154.28	1.53	2.69	0.61	1.88	2.87	1.05	0.97	0.85	1.08
Both	Albania	49.25	56.75	38.18	106.45	134.51	83.59	1.89	2.17	1.47	3.00	3.80	2.35	1.64	1.44	1.85
Both	Algeria	349.86	421.46	290.84	1815.29	2215.22	1461.44	1.98	2.46	1.66	4.40	5.30	3.60	3.39	3.19	3.59
Both	American Samoa	0.67	0.83	0.55	1.92	2.39	1.60	2.46	3.03	1.96	3.91	4.79	3.30	2.03	1.42	2.64
Both	Andorra	1.97	2.65	1.49	4.92	6.84	3.79	3.16	4.19	2.40	4.03	5.60	3.11	0.77	0.55	1.00
Both	Angola	39.32	57.20	24.59	123.75	181.75	94.09	0.75	1.04	0.52	0.85	1.24	0.67	0.37	0.23	0.52
Both	Antigua	1.20	1.38	1.04	4.04	4.73	3.43	2.30	2.64	1.99	3.79	4.39	3.24	1.81	1.47	2.14
Both	Argentina	789.95	862.22	720.57	1292.59	1496.95	1104.67	2.42	2.65	2.21	2.63	3.06	2.24	-0.03	-0.32	0.26
Both	Armenia	21.48	24.95	18.32	110.18	123.71	97.15	0.71	0.81	0.61	2.78	3.12	2.47	5.69	4.60	6.80
Both	Australia	510.62	557.11	469.16	1743.14	2030.71	1493.41	2.68	2.92	2.45	5.38	6.25	4.58	3.16	2.95	3.37
Both	Austria	558.58	611.73	510.02	637.85	713.18	560.76	5.44	6.00	4.92	4.56	5.11	4.01	-1.11	-1.34	-0.89
Both	Azerbaijan	85.72	103.09	73.23	282.38	351.76	225.86	1.43	1.75	1.23	2.54	3.15	2.05	1.88	1.50	2.25
Both	Bahamas	5.10	5.79	4.48	15.92	18.54	13.55	2.52	2.83	2.23	3.75	4.35	3.20	1.80	1.63	1.97
Both	Bahrain	3.98	5.12	3.40	27.22	32.91	22.78	1.54	1.82	1.33	2.07	2.47	1.65	1.18	0.69	1.67
Both	Bangladesh	781.09	1168.71	558.74	2731.31	3886.38	2040.38	1.09	1.61	0.81	1.81	2.53	1.38	2.31	2.09	2.53
Both	Barbados	6.52	7.23	5.90	15.60	17.99	13.46	2.51	2.79	2.25	3.86	4.48	3.32	1.64	1.48	1.79
Both	Barbuda	1.20	1.38	1.04	4.04	4.73	3.43	2.30	2.64	1.99	3.79	4.39	3.24	1.81	1.47	2.14
Both	Belarus	360.12	404.09	323.28	632.92	747.73	548.51	3.01	3.38	2.70	4.51	5.33	3.93	1.46	1.15	1.76
Both	Belgium	633.62	694.81	569.12	744.10	862.58	649.55	4.67	5.16	4.19	4.39	5.11	3.82	0.00	-0.28	0.27

© 2020 Deng Y et al. *JAMA Network Open.*

Sex	Location	Incident cases						Age-standardized incidence rate (per 100,000)						EAPC		
		1990(95%UI)			2017(95%UI)			1990(95%UI)			2017(95%UI)			1990-2017(95%CI)		
		Cases (No.)	UL	LL	Cases (No.)	UL	LL	95%UI	UL	LL	95%UI	UL	LL	EAPC	LL	UL
Both	Belize	0.90	1.02	0.80	5.11	5.90	4.45	0.83	0.94	0.73	1.55	1.79	1.35	2.45	2.33	2.57
Both	Benin	14.10	17.84	9.99	34.02	42.86	25.60	0.55	0.67	0.41	0.57	0.72	0.43	0.05	-0.01	0.11
Both	Bermuda	2.43	2.72	2.17	4.66	5.36	4.01	3.62	4.05	3.24	4.47	5.16	3.85	0.73	0.61	0.86
Both	Bhutan	4.64	7.13	3.13	16.62	25.35	10.93	1.22	1.89	0.86	1.88	2.75	1.33	1.59	1.48	1.70
Both	Bolivia	91.65	124.38	57.68	360.46	473.41	263.49	2.35	3.13	1.52	3.79	4.95	2.79	1.74	1.59	1.88
Both	Bosnia and Herzegovina	74.43	87.21	56.15	151.03	179.90	111.36	1.58	1.83	1.22	2.95	3.56	2.13	2.59	2.40	2.79
Both	Botswana	6.06	8.58	4.37	22.30	29.67	17.60	0.85	1.18	0.63	1.27	1.69	1.02	2.28	1.60	2.96
Both	Brazil	1788.63	1870.16	1713.37	5375.98	5598.59	5117.00	1.66	1.73	1.59	2.27	2.36	2.16	1.16	0.97	1.34
Both	Brunei	5.44	6.89	4.19	26.34	31.45	21.37	3.62	4.48	2.88	6.20	7.28	5.09	2.75	2.44	3.05
Both	Bulgaria	280.82	309.23	250.15	323.34	368.08	284.18	2.45	2.70	2.18	3.06	3.50	2.67	0.64	0.50	0.79
Both	Burkina Faso	36.09	48.16	23.58	68.21	84.52	54.71	0.67	0.90	0.45	0.60	0.74	0.49	-0.57	-0.70	-0.44
Both	Burundi	44.94	58.97	30.62	82.41	106.35	63.57	1.38	1.78	0.97	1.28	1.62	1.01	-0.32	-0.41	-0.22
Both	Cambodia	108.04	148.93	64.81	282.00	367.04	218.63	1.94	2.61	1.22	2.18	2.82	1.71	0.30	0.16	0.43
Both	Cameroon	45.71	56.40	31.26	102.57	133.18	77.60	0.78	0.95	0.56	0.69	0.88	0.53	-0.54	-0.64	-0.45
Both	Canada	1147.00	1259.18	1038.88	2385.58	2714.81	2095.95	3.63	3.99	3.29	4.72	5.37	4.10	0.92	0.60	1.23
Both	Cape Verde	1.12	1.46	0.72	4.34	5.43	2.75	0.51	0.66	0.31	0.86	1.07	0.55	2.17	1.95	2.39
Both	Central African Republic	11.76	16.57	7.75	17.44	26.22	11.19	0.82	1.11	0.59	0.67	0.99	0.46	-0.71	-0.78	-0.64
Both	Chad	17.07	21.82	11.46	32.90	40.48	26.11	0.50	0.64	0.35	0.49	0.59	0.39	-0.20	-0.27	-0.13
Both	Chile	262.49	290.72	235.90	892.29	1040.73	764.03	2.32	2.55	2.09	4.06	4.74	3.48	2.19	2.10	2.29
Both	China	11015.78	12064.06	9503.28	41510.73	47065.27	38331.90	1.07	1.18	0.95	2.17	2.46	2.01	2.70	2.31	3.10
Both	Colombia	592.32	654.54	542.29	1913.61	2286.38	1625.01	2.65	2.89	2.44	3.57	4.25	3.02	1.02	0.74	1.29

© 2020 Deng Y et al. *JAMA Network Open.*

Sex	Location	Incident cases						Age-standardized incidence rate (per 100,000)						EAPC		
		1990(95%UI)			2017(95%UI)			1990(95%UI)			2017(95%UI)			1990-2017(95%CI)		
		Cases (No.)	UL	LL	Cases (No.)	UL	LL	95%UI	UL	LL	95%UI	UL	LL	EAPC	LL	UL
Both	Comoros	3.60	5.01	2.57	8.76	11.35	6.87	1.21	1.67	0.88	1.49	1.93	1.19	1.08	0.88	1.27
Both	Costa Rica	72.63	80.79	65.03	213.56	246.01	183.94	3.16	3.50	2.85	4.19	4.82	3.62	0.66	0.33	0.98
Both	Croatia	287.69	321.26	253.04	259.82	294.02	225.40	4.45	4.95	3.95	3.67	4.14	3.20	0.15	-0.22	0.53
Both	Cuba	243.57	273.77	219.21	630.06	738.63	536.51	2.28	2.57	2.05	3.86	4.52	3.29	2.02	1.73	2.31
Both	Cyprus	24.14	28.52	16.81	63.59	77.76	53.54	2.90	3.43	2.01	3.70	4.55	3.10	0.82	0.40	1.25
Both	Czech Republic	602.46	659.50	543.74	669.96	766.42	591.07	4.69	5.13	4.25	4.07	4.69	3.59	-0.56	-0.77	-0.36
Both	Democratic Republic of the Congo	131.70	178.97	97.68	284.20	413.26	205.85	0.67	0.88	0.52	0.67	0.96	0.48	-0.15	-0.33	0.03
Both	Denmark	176.29	196.30	159.31	318.85	364.48	278.54	2.71	3.03	2.44	3.84	4.41	3.34	1.34	1.10	1.57
Both	Djibouti	3.18	4.61	2.09	13.35	19.85	8.87	1.12	1.60	0.77	1.52	2.22	1.03	1.19	1.02	1.37
Both	Dominica	1.02	1.13	0.93	2.19	2.46	1.94	1.58	1.77	1.42	2.70	3.04	2.38	2.09	1.92	2.25
Both	Dominican Republic	51.54	60.11	41.61	226.44	286.05	175.69	1.10	1.28	0.88	2.24	2.80	1.74	2.75	2.26	3.24
Both	Ecuador	83.30	91.44	75.73	821.56	969.20	694.52	1.27	1.38	1.16	5.29	6.23	4.48	5.92	5.20	6.64
Both	Egypt	348.42	538.72	277.90	1698.09	2548.36	1238.74	0.88	1.41	0.71	2.03	3.12	1.49	3.31	3.09	3.53
Both	El Salvador	87.76	104.11	46.75	158.27	222.39	118.30	2.61	3.08	1.41	2.77	3.88	2.07	0.27	-0.48	1.02
Both	Equatorial Guinea	1.89	2.81	1.17	9.04	15.18	5.25	0.82	1.19	0.55	1.32	2.16	0.79	2.15	1.88	2.42
Both	Eritrea	17.61	24.70	11.12	66.29	88.93	47.37	1.22	1.65	0.84	1.78	2.35	1.30	1.04	0.81	1.27
Both	Estonia	71.71	79.74	64.87	107.33	130.61	87.73	3.87	4.32	3.47	5.38	6.55	4.35	1.29	1.02	1.57
Both	Ethiopia	1099.57	1626.93	598.75	2127.67	2662.39	1659.67	3.71	5.39	2.22	3.32	4.05	2.64	-0.67	-0.84	-0.50
Both	Fiji	17.07	21.60	10.23	34.21	43.90	19.68	3.58	4.56	2.13	4.04	5.14	2.36	0.25	0.10	0.40
Both	Finland	210.12	229.39	190.84	394.86	449.44	344.47	3.14	3.43	2.86	4.24	4.85	3.70	1.45	1.27	1.64

© 2020 Deng Y et al. *JAMA Network Open.*

Sex	Location	Incident cases						Age-standardized incidence rate (per 100,000)						EAPC		
		1990(95%UI)			2017(95%UI)			1990(95%UI)			2017(95%UI)			1990-2017(95%CI)		
		Cases (No.)	UL	LL	Cases (No.)	UL	LL	95%UI	UL	LL	95%UI	UL	LL	EAPC	LL	UL
Both	France	2646.19	2868.61	2435.11	3748.19	4259.97	3323.18	3.69	4.02	3.40	3.87	4.44	3.41	0.44	-0.42	1.30
Both	Gabon	5.58	7.73	4.06	11.73	18.02	8.76	0.89	1.22	0.66	0.94	1.42	0.71	0.20	0.11	0.30
Both	Gambia	2.83	3.49	2.26	8.80	11.74	6.95	0.55	0.67	0.45	0.71	0.93	0.56	1.12	1.02	1.22
Both	Georgia	96.56	110.92	79.45	168.02	190.50	146.62	1.55	1.78	1.29	3.41	3.87	2.96	3.23	2.17	4.31
Both	Germany	4496.84	4824.89	4146.97	6795.67	7837.01	5854.98	4.13	4.47	3.79	5.20	6.02	4.46	0.76	0.52	1.01
Both	Ghana	19.67	24.12	15.76	60.18	71.26	50.42	0.26	0.33	0.21	0.33	0.39	0.28	0.73	0.60	0.86
Both	Greece	328.20	362.40	295.90	568.04	639.56	496.90	2.39	2.63	2.15	3.38	3.83	2.95	1.24	0.99	1.50
Both	Greenland	0.59	0.70	0.51	1.43	1.79	0.89	1.40	1.62	1.16	2.04	2.50	1.28	1.70	1.16	2.24
Both	Grenada	2.36	2.62	2.10	4.17	4.72	3.69	3.54	3.93	3.15	3.23	3.67	2.85	0.43	-0.07	0.93
Both	Grenadines	2.09	2.39	1.84	5.08	5.87	4.40	2.72	3.10	2.39	3.89	4.51	3.37	1.49	1.19	1.80
Both	Guam	1.59	2.14	1.32	6.56	7.68	5.53	1.60	2.08	1.35	3.59	4.17	3.05	3.23	2.14	4.34
Both	Guatemala	53.65	59.54	48.74	242.62	284.83	204.31	1.19	1.31	1.09	1.95	2.28	1.65	0.32	-0.29	0.93
Both	Guinea	27.03	31.24	22.94	57.81	71.02	47.58	0.67	0.76	0.58	0.81	1.00	0.67	0.83	0.76	0.89
Both	Guinea-Bissau	3.88	4.97	2.61	6.02	8.28	4.09	0.72	0.91	0.51	0.65	0.86	0.46	-0.52	-0.64	-0.39
Both	Guyana	5.84	6.45	5.25	14.17	16.62	12.07	1.17	1.28	1.06	2.01	2.35	1.72	2.14	1.87	2.41
Both	Haiti	52.21	81.60	29.95	131.01	198.03	88.63	1.34	2.06	0.81	1.58	2.34	1.10	0.62	0.53	0.71
Both	Honduras	31.86	37.14	27.15	121.25	195.32	86.82	1.31	1.51	1.12	1.83	2.88	1.32	1.25	1.06	1.44
Both	Hungary	558.44	619.69	507.96	524.83	595.00	462.51	4.05	4.50	3.67	3.42	3.89	3.02	-0.61	-0.81	-0.40
Both	Iceland	30.18	33.49	27.26	51.49	58.26	45.27	11.30	12.58	10.17	11.57	13.03	10.18	0.36	0.19	0.52
Both	India	7368.53	9201.24	5769.57	25675.36	28164.28	22566.69	1.10	1.36	0.89	1.99	2.18	1.76	2.21	2.03	2.40
Both	Indonesia	2074.87	2475.55	1525.04	5034.27	5988.43	4051.21	1.63	1.92	1.25	2.02	2.39	1.66	0.70	0.64	0.76
Both	Iran	470.79	574.81	382.58	2672.60	3014.57	2016.09	1.34	1.63	1.09	3.16	3.55	2.36	4.01	3.64	4.38
Both	Iraq	222.13	303.95	145.72	506.52	621.07	396.93	2.12	2.87	1.45	1.66	2.00	1.28	-1.06	-1.31	-0.81

© 2020 Deng Y et al. *JAMA Network Open.*

Sex	Location	Incident cases						Age-standardized incidence rate (per 100,000)						EAPC		
		1990(95%UI)			2017(95%UI)			1990(95%UI)			2017(95%UI)			1990-2017(95%CI)		
		Cases (No.)	UL	LL	Cases (No.)	UL	LL	95%UI	UL	LL	95%UI	UL	LL	EAPC	LL	UL
Both	Ireland	111.75	123.65	101.14	252.71	293.26	217.15	2.94	3.26	2.64	3.99	4.63	3.44	1.71	1.46	1.96
Both	Israel	127.88	141.04	116.73	451.43	514.85	394.67	2.70	2.99	2.46	4.52	5.14	3.95	1.73	1.25	2.21
Both	Italy	5399.21	5939.56	4884.52	6736.96	7638.54	5843.99	7.13	7.87	6.41	6.79	7.79	5.87	-0.08	-0.41	0.25
Both	Ivory Coast	40.65	51.03	31.23	107.24	145.34	79.39	0.60	0.73	0.50	0.68	0.88	0.52	0.51	0.43	0.59
Both	Jamaica	22.60	25.45	20.03	106.17	133.20	85.77	1.22	1.38	1.08	3.61	4.55	2.92	4.20	3.76	4.63
Both	Japan	6029.94	6356.45	5750.41	11356.11	12338.18	10537.76	3.63	3.83	3.46	4.72	5.16	4.37	1.16	0.96	1.35
Both	Jordan	64.52	82.86	46.42	225.50	307.14	174.51	2.86	3.61	2.11	2.81	3.77	2.21	-0.69	-1.27	-0.11
Both	Kazakhstan	628.42	768.52	543.59	680.53	800.00	588.18	4.17	5.04	3.61	3.58	4.18	3.10	-1.41	-1.86	-0.96
Both	Kenya	62.84	79.05	51.84	231.19	285.27	192.47	0.50	0.61	0.42	0.70	0.85	0.59	1.34	1.24	1.44
Both	Kiribati	0.25	0.29	0.22	0.51	0.63	0.42	0.61	0.69	0.53	0.68	0.82	0.56	0.54	0.44	0.64
Both	Kuwait	43.82	50.62	37.91	112.11	135.01	94.93	3.36	3.74	2.97	2.78	3.26	2.41	-0.51	-1.01	0.00
Both	Kyrgyzstan	75.73	87.37	65.26	95.47	112.53	83.00	2.17	2.49	1.87	1.78	2.07	1.55	-0.30	-0.60	0.00
Both	Laos	47.27	69.39	26.39	98.70	134.06	70.08	1.90	2.73	1.12	1.91	2.53	1.39	-0.22	-0.39	-0.06
Both	Latvia	111.60	123.78	99.84	152.30	184.96	125.43	3.46	3.85	3.08	4.87	5.92	4.00	1.36	1.02	1.71
Both	Lebanon	112.71	146.39	77.95	784.54	1017.88	613.82	3.69	4.79	2.66	9.80	12.41	7.79	3.96	3.85	4.06
Both	Lesotho	9.03	11.59	7.14	15.13	21.71	10.41	0.82	1.05	0.65	1.12	1.60	0.78	1.76	1.49	2.03
Both	Liberia	8.10	10.21	5.57	14.20	18.42	10.70	0.62	0.77	0.43	0.54	0.69	0.42	-0.56	-0.79	-0.34
Both	Libya	61.85	80.54	46.78	381.86	503.41	290.44	2.18	2.86	1.70	5.42	7.12	4.19	4.14	3.77	4.51
Both	Lithuania	170.58	188.60	152.98	201.60	232.36	174.94	4.00	4.43	3.57	4.66	5.37	4.04	0.36	-0.03	0.74
Both	Luxembourg	31.11	34.56	27.86	56.86	68.26	47.79	6.16	6.85	5.48	6.69	8.01	5.64	0.02	-0.24	0.28
Both	Macedonia	42.85	51.34	29.16	79.03	95.73	60.73	2.05	2.44	1.41	2.63	3.17	2.04	0.44	0.13	0.74
Both	Madagascar	87.48	117.93	65.25	205.65	271.30	157.63	1.10	1.46	0.84	1.23	1.58	0.96	0.49	0.34	0.64
Both	Malawi	70.03	97.87	30.61	171.68	244.07	116.57	1.13	1.52	0.60	1.47	2.00	1.06	0.60	0.40	0.81

© 2020 Deng Y et al. *JAMA Network Open.*

Sex	Location	Incident cases						Age-standardized incidence rate (per 100,000)						EAPC		
		1990(95%UI)			2017(95%UI)			1990(95%UI)			2017(95%UI)			1990-2017(95%CI)		
		Cases (No.)	UL	LL	Cases (No.)	UL	LL	95%UI	UL	LL	95%UI	UL	LL	EAPC	LL	UL
Both	Malaysia	308.34	360.91	251.69	1480.53	1881.04	1176.85	2.51	2.91	2.12	4.98	6.37	4.01	2.53	2.42	2.64
Both	Maldives	3.29	4.84	1.88	15.15	18.01	12.92	3.14	4.53	1.97	4.35	5.19	3.68	0.93	0.78	1.07
Both	Mali	61.09	72.73	49.72	157.91	228.75	114.62	1.11	1.31	0.95	1.29	1.78	0.95	0.42	0.33	0.51
Both	Malta	15.65	17.40	14.05	34.92	39.64	30.93	3.64	4.05	3.27	5.15	5.85	4.57	1.43	1.16	1.70
Both	Marshall Islands	0.38	0.48	0.27	1.28	1.80	0.81	1.81	2.28	1.30	2.93	4.00	1.93	1.80	1.60	2.00
Both	Mauritania	8.28	10.24	5.51	16.99	21.79	13.18	0.67	0.83	0.45	0.71	0.91	0.54	0.06	-0.09	0.22
Both	Mauritius	13.32	14.86	11.90	42.77	49.53	36.29	1.52	1.68	1.36	2.53	2.93	2.17	2.27	1.81	2.72
Both	Mexico	962.73	994.74	931.58	4670.01	4872.89	4455.41	1.83	1.89	1.78	3.78	3.94	3.61	2.46	2.29	2.64
Both	Micronesia	0.83	1.07	0.63	1.74	2.43	1.16	1.48	1.88	1.16	2.12	2.86	1.49	1.34	1.20	1.48
Both	Moldova	108.79	123.47	95.82	149.30	169.93	130.29	2.29	2.59	2.02	2.85	3.24	2.49	1.12	0.72	1.53
Both	Mongolia	10.81	12.29	8.55	40.42	49.65	29.13	0.92	1.05	0.69	1.52	1.89	0.98	2.31	1.99	2.63
Both	Montenegro	21.52	25.75	17.82	39.94	47.95	33.23	3.26	3.88	2.71	4.54	5.45	3.78	1.35	1.26	1.43
Both	Morocco	426.97	524.21	313.55	1500.75	1995.13	1122.38	2.22	2.66	1.75	4.13	5.46	3.12	2.39	2.34	2.44
Both	Mozambique	98.17	136.22	72.28	283.87	381.38	192.19	1.08	1.50	0.82	1.58	2.10	1.11	1.51	1.37	1.65
Both	Myanmar	631.65	905.87	368.23	1303.98	1747.97	942.29	2.28	3.21	1.39	2.58	3.44	1.89	0.31	0.22	0.40
Both	Namibia	7.78	10.28	5.47	21.60	27.51	16.67	0.86	1.19	0.62	1.19	1.52	0.93	0.93	0.48	1.39
Both	Nepal	152.67	235.30	105.81	441.74	638.42	319.98	1.14	1.74	0.82	1.71	2.44	1.26	1.60	1.22	1.98
Both	Netherlands	587.10	644.13	527.68	1247.93	1417.31	1089.63	3.19	3.51	2.87	4.74	5.41	4.16	1.67	1.49	1.84
Both	New Zealand	133.42	149.17	119.77	282.34	317.69	250.69	3.53	3.96	3.17	4.83	5.45	4.28	1.45	1.27	1.63
Both	Nicaragua	21.71	29.40	18.38	124.72	151.95	100.83	1.04	1.42	0.89	2.36	2.87	1.92	3.75	3.29	4.22
Both	Niger	19.55	24.67	12.68	41.16	52.17	31.86	0.50	0.61	0.34	0.45	0.56	0.35	-0.63	-0.76	-0.50
Both	Nigeria	180.94	251.35	113.00	493.98	718.82	332.19	0.37	0.52	0.23	0.47	0.67	0.33	0.89	0.75	1.03
Both	North Korea	348.34	455.29	269.93	629.13	870.96	470.64	1.78	2.32	1.39	1.99	2.75	1.50	0.33	0.18	0.49

© 2020 Deng Y et al. *JAMA Network Open.*

Sex	Location	Incident cases						Age-standardized incidence rate (per 100,000)						EAPC		
		1990(95%UI)			2017(95%UI)			1990(95%UI)			2017(95%UI)			1990-2017(95%CI)		
		Cases (No.)	UL	LL	Cases (No.)	UL	LL	95%UI	UL	LL	95%UI	UL	LL	EAPC	LL	UL
Both	Northern Mariana Islands	0.66	0.83	0.52	1.71	2.10	1.42	2.06	2.51	1.70	2.97	3.60	2.50	0.89	0.23	1.55
Both	Norway	200.67	215.72	187.55	380.32	418.41	347.22	3.75	4.05	3.50	5.18	5.71	4.73	1.39	1.13	1.65
Both	Oman	23.24	30.51	16.18	145.10	182.53	114.13	1.84	2.38	1.36	3.56	4.35	2.86	2.66	2.50	2.81
Both	Pakistan	2234.90	2990.62	1758.19	9104.45	13714.52	6159.55	2.76	3.57	2.24	4.91	7.06	3.46	2.21	2.07	2.34
Both	Palestine	18.14	23.63	13.37	52.95	64.90	44.99	1.69	2.19	1.23	1.74	2.05	1.47	0.15	-0.03	0.34
Both	Panama	36.23	40.29	32.41	138.72	158.94	120.75	1.88	2.08	1.70	3.46	3.95	3.01	2.36	2.22	2.51
Both	Papua New Guinea	32.66	45.23	23.89	110.23	154.03	78.38	1.36	1.84	1.04	1.78	2.40	1.33	0.99	0.81	1.16
Both	Paraguay	36.45	42.43	31.36	160.69	206.78	121.49	1.41	1.64	1.21	2.74	3.51	2.05	2.46	2.18	2.75
Both	Peru	244.16	291.24	210.15	1165.60	1445.10	912.06	1.67	1.97	1.43	3.67	4.53	2.87	3.17	2.80	3.54
Both	Philippines	1128.82	1314.36	991.83	4868.24	5976.49	3985.03	2.86	3.29	2.54	5.66	6.89	4.67	2.79	2.62	2.96
Both	Poland	1683.61	1825.23	1547.59	2420.01	2747.72	2121.20	3.81	4.14	3.50	4.25	4.87	3.69	0.09	-0.24	0.42
Both	Portugal	491.02	540.71	443.84	769.65	883.83	661.26	3.79	4.18	3.44	4.23	4.88	3.62	0.09	-0.17	0.34
Both	Puerto Rico	115.77	129.44	102.69	155.44	177.10	135.15	3.18	3.56	2.82	3.08	3.52	2.67	-0.04	-0.41	0.34
Both	Qatar	8.68	12.17	3.37	43.65	56.47	34.32	3.61	4.69	1.48	2.18	2.73	1.75	-2.50	-2.96	-2.04
Both	Republic of Congo	12.32	17.99	7.92	32.92	52.23	22.44	0.94	1.35	0.66	0.99	1.53	0.72	0.15	-0.02	0.33
Both	Romania	648.62	713.74	585.36	1109.37	1261.97	965.26	2.36	2.59	2.14	3.85	4.37	3.33	1.49	1.22	1.76
Both	Russia	5467.84	6167.52	4972.23	10540.59	11412.06	9769.69	3.06	3.45	2.79	5.33	5.76	4.92	2.24	1.88	2.60
Both	Rwanda	59.69	83.95	36.10	124.37	174.52	93.56	1.36	1.89	0.84	1.44	1.98	1.12	0.04	-0.20	0.29
Both	Saint Lucia	2.77	3.11	2.48	8.28	9.63	7.05	2.91	3.27	2.59	3.89	4.50	3.34	0.93	0.55	1.30
Both	Saint Vincent	2.09	2.39	1.84	5.08	5.87	4.40	2.72	3.10	2.39	3.89	4.51	3.37	1.49	1.19	1.80

© 2020 Deng Y et al. *JAMA Network Open.*

Sex	Location	Incident cases						Age-standardized incidence rate (per 100,000)						EAPC		
		1990(95%UI)			2017(95%UI)			1990(95%UI)			2017(95%UI)			1990-2017(95%CI)		
		Cases (No.)	UL	LL	Cases (No.)	UL	LL	95%UI	UL	LL	95%UI	UL	LL	EAPC	LL	UL
Both	Samoa	2.56	3.17	2.01	4.99	6.64	3.58	2.31	2.88	1.84	3.14	4.14	2.31	1.04	0.88	1.20
Both	Sao Tome and Principe	0.43	0.64	0.30	1.03	1.32	0.80	0.55	0.80	0.39	0.76	0.94	0.60	0.92	0.74	1.10
Both	Saudi Arabia	198.87	256.63	155.75	2590.31	3391.63	1964.82	1.87	2.46	1.50	7.16	8.90	5.71	5.91	5.45	6.37
Both	Senegal	22.71	27.82	16.51	46.40	58.10	37.71	0.55	0.66	0.41	0.54	0.68	0.44	-0.25	-0.40	-0.09
Both	Serbia	280.92	341.20	222.69	432.84	503.22	349.27	2.44	2.95	1.96	3.33	3.89	2.70	1.59	1.43	1.75
Both	Seychelles	0.71	0.82	0.55	1.83	2.14	1.56	1.22	1.41	0.94	1.55	1.80	1.32	0.80	0.70	0.91
Both	Sierra Leone	13.10	17.32	9.09	26.32	34.32	20.14	0.54	0.71	0.39	0.60	0.77	0.46	0.35	0.26	0.44
Both	Singapore	83.72	93.89	74.56	266.86	314.64	229.12	2.77	3.10	2.50	3.76	4.42	3.23	1.68	1.45	1.91
Both	Slovakia	192.58	218.17	163.69	265.35	339.56	220.08	3.27	3.71	2.78	3.27	4.22	2.70	-0.16	-0.27	-0.05
Both	Slovenia	87.97	98.13	79.04	120.90	139.83	103.48	3.64	4.04	3.27	3.70	4.27	3.16	0.05	-0.13	0.23
Both	Solomon Islands	2.20	3.00	1.62	6.47	8.86	4.66	1.25	1.68	0.96	1.57	2.08	1.18	0.67	0.48	0.86
Both	Somalia	38.47	63.26	16.38	116.58	163.06	82.29	0.98	1.56	0.52	1.22	1.66	0.88	0.66	0.52	0.79
Both	South Africa	275.60	310.63	231.33	509.24	628.89	453.88	1.02	1.14	0.87	1.02	1.25	0.91	-0.55	-1.09	0.00
Both	South Korea	564.63	620.53	503.39	8968.26	10459.53	7669.50	1.49	1.61	1.34	12.87	15.23	11.04	10.70	8.85	12.59
Both	South Sudan	38.95	66.98	17.73	78.03	114.53	51.68	1.09	1.78	0.59	1.34	1.95	0.92	0.80	0.62	0.97
Both	Spain	1284.68	1393.03	1181.33	2631.48	2977.00	2305.73	2.64	2.87	2.42	3.59	4.09	3.13	1.29	1.07	1.51
Both	Sri Lanka	299.15	348.54	230.78	924.42	1214.44	686.26	2.30	2.65	1.78	3.63	4.75	2.73	1.21	0.78	1.64
Both	Sudan	124.16	191.94	75.14	388.26	545.24	264.41	1.03	1.55	0.67	1.53	2.11	1.06	1.52	1.39	1.65
Both	Suriname	4.40	5.01	3.88	14.79	17.36	12.53	1.48	1.68	1.31	2.41	2.82	2.05	1.91	1.63	2.20
Both	Swaziland	4.15	5.46	3.26	8.97	13.22	6.07	1.08	1.43	0.86	1.28	1.89	0.87	0.77	0.49	1.06
Both	Sweden	327.51	355.22	301.58	522.66	582.80	470.41	2.71	2.96	2.49	3.57	4.00	3.20	1.25	1.08	1.41
Both	Switzerland	427.36	472.66	383.70	574.13	657.39	497.96	4.88	5.41	4.37	4.34	5.00	3.79	-0.19	-0.46	0.09

© 2020 Deng Y et al. *JAMA Network Open.*

Sex	Location	Incident cases						Age-standardized incidence rate (per 100,000)						EAPC		
		1990(95%UI)			2017(95%UI)			1990(95%UI)			2017(95%UI)			1990-2017(95%CI)		
		Cases (No.)	UL	LL	Cases (No.)	UL	LL	95%UI	UL	LL	95%UI	UL	LL	EAPC	LL	UL
Both	Syria	40.41	52.24	27.59	166.32	209.34	129.06	0.61	0.79	0.41	1.05	1.31	0.83	1.83	1.64	2.01
Both	Tajikistan	11.72	13.84	10.21	33.94	40.20	29.24	0.37	0.44	0.32	0.51	0.60	0.45	0.92	0.54	1.29
Both	Tanzania	187.50	259.83	113.35	543.56	742.88	407.17	1.18	1.60	0.79	1.51	2.02	1.17	0.67	0.43	0.92
Both	Thailand	883.27	1280.44	746.31	2778.82	5675.53	2095.14	2.02	2.87	1.73	2.85	5.93	2.15	0.81	0.37	1.25
Both	Timor-Leste	5.63	7.78	3.59	16.75	21.66	11.50	1.40	1.88	0.96	1.93	2.49	1.37	1.31	1.05	1.57
Both	Tobago	18.63	20.82	16.69	45.54	58.27	34.65	1.92	2.14	1.73	2.61	3.32	1.99	0.66	0.23	1.09
Both	Togo	10.96	14.58	7.51	24.34	30.69	18.87	0.59	0.77	0.42	0.54	0.67	0.42	-0.49	-0.63	-0.35
Both	Tonga	0.74	0.90	0.61	1.68	2.10	1.32	1.24	1.50	1.03	1.99	2.48	1.57	1.84	1.67	2.01
Both	Trinidad	18.63	20.82	16.69	45.54	58.27	34.65	1.92	2.14	1.73	2.61	3.32	1.99	0.66	0.23	1.09
Both	Tunisia	120.09	144.39	100.18	478.41	639.16	349.11	1.82	2.20	1.54	3.75	4.99	2.75	2.54	2.26	2.83
Both	Turkey	724.81	1063.07	491.19	2964.33	4399.94	2402.34	1.73	2.48	1.19	3.28	4.89	2.66	2.81	2.54	3.07
Both	Turkmenistan	33.82	37.69	30.35	85.40	98.54	73.57	1.41	1.57	1.26	1.80	2.06	1.55	0.92	0.30	1.56
Both	Uganda	106.92	133.68	84.33	379.22	502.12	283.54	1.07	1.31	0.86	1.68	2.14	1.31	1.81	1.65	1.98
Both	UK	1768.66	1836.99	1695.44	3243.99	3368.01	3121.12	2.34	2.44	2.25	3.44	3.57	3.31	1.52	1.38	1.67
Both	Ukraine	1555.53	1826.81	1341.34	2711.21	3077.93	2396.26	2.30	2.69	1.99	4.34	4.98	3.83	2.36	2.00	2.71
Both	United Arab Emirates	19.24	26.47	14.19	313.24	440.57	217.58	1.71	2.48	1.19	3.09	4.52	2.05	2.53	2.21	2.86
Both	Uruguay	49.64	55.30	44.76	128.19	150.61	108.73	1.40	1.56	1.26	3.03	3.57	2.55	2.39	1.78	3.01
Both	USA	10832.65	11149.24	10554.57	25895.65	26874.13	24842.13	3.69	3.80	3.59	5.54	5.75	5.30	1.37	1.12	1.62
Both	Uzbekistan	57.38	74.75	49.91	247.69	292.06	206.47	0.42	0.57	0.37	0.89	1.04	0.75	2.99	2.84	3.14
Both	Vanuatu	1.22	1.82	0.77	3.75	5.89	2.33	1.46	2.15	0.94	1.93	2.95	1.20	1.10	1.03	1.18
Both	Venezuela	168.56	187.39	152.49	933.44	1137.25	750.18	1.39	1.52	1.26	3.02	3.67	2.45	2.83	2.53	3.13
Both	Vietnam	1324.09	1629.78	1097.60	7004.00	9025.46	5515.56	2.70	3.46	2.23	6.67	8.49	5.31	4.05	3.72	4.38

© 2020 Deng Y et al. *JAMA Network Open.*

Sex	Location	Incident cases						Age-standardized incidence rate (per 100,000)						EAPC		
		1990(95%UI)			2017(95%UI)			1990(95%UI)			2017(95%UI)			1990-2017(95%CI)		
		Cases (No.)	UL	LL	Cases (No.)	UL	LL	95%UI	UL	LL	95%UI	UL	LL	EAPC	LL	UL
Both	Virgin Islands	1.44	1.66	1.25	4.36	5.13	3.64	1.41	1.61	1.24	2.82	3.34	2.37	3.18	2.89	3.46
Both	Yemen	63.93	109.52	32.50	270.27	403.87	178.48	0.94	1.55	0.54	1.47	2.15	1.00	1.84	1.75	1.92
Both	Zambia	73.41	102.84	48.43	149.49	196.81	115.96	1.55	2.10	1.09	1.40	1.82	1.10	-0.69	-1.17	-0.20
Both	Zimbabwe	86.03	104.20	63.23	180.46	230.66	120.92	1.54	1.83	1.22	1.93	2.42	1.44	1.02	0.49	1.54
Female	Afghanistan	84.52	167.44	28.67	261.94	461.77	114.00	2.46	4.85	0.88	3.00	5.09	1.44	0.97	0.81	1.12
Female	Albania	33.98	40.99	22.29	69.64	96.95	49.42	2.68	3.24	1.72	3.83	5.33	2.73	1.27	1.09	1.45
Female	Algeria	279.01	351.92	222.84	1439.11	1830.40	1069.44	3.11	4.00	2.54	6.95	8.70	5.39	3.43	3.22	3.65
Female	American Samoa	0.56	0.72	0.44	1.64	2.09	1.32	4.20	5.33	3.22	6.50	8.16	5.34	1.97	1.36	2.59
Female	Andorra	1.34	1.97	0.91	3.23	5.04	2.24	4.56	6.66	3.09	5.46	8.47	3.84	0.38	0.17	0.60
Female	Angola	29.08	45.86	16.32	91.58	148.60	64.14	1.08	1.70	0.67	1.14	1.86	0.81	0.07	-0.08	0.21
Female	Antigua	1.08	1.26	0.92	3.63	4.32	3.02	3.86	4.51	3.30	6.46	7.63	5.44	1.84	1.41	2.26
Female	Argentina	584.77	651.84	519.25	924.47	1125.04	757.14	3.41	3.82	3.02	3.58	4.37	2.92	-0.19	-0.52	0.14
Female	Armenia	15.40	18.13	12.85	78.58	90.71	66.78	0.93	1.09	0.78	3.59	4.12	3.08	5.78	4.70	6.87
Female	Australia	330.16	366.10	298.30	1134.71	1366.94	910.15	3.35	3.72	3.01	6.85	8.32	5.46	3.22	2.98	3.47
Female	Austria	405.81	454.48	361.51	399.91	463.03	338.25	7.31	8.23	6.42	5.67	6.62	4.78	-1.29	-1.51	-1.06
Female	Azerbaijan	65.83	82.87	53.84	227.49	296.17	174.92	2.03	2.57	1.67	3.88	5.03	2.99	2.31	1.96	2.66
Female	Bahamas	3.82	4.50	3.25	11.41	13.87	9.14	3.52	4.10	3.00	5.09	6.16	4.09	1.61	1.40	1.82
Female	Bahrain	3.06	4.17	2.50	20.05	25.38	15.94	2.80	3.46	2.33	4.15	5.09	3.15	1.63	1.11	2.15
Female	Bangladesh	598.16	1002.29	385.13	2169.24	3372.86	1541.98	1.67	2.83	1.14	2.80	4.25	2.04	2.42	2.17	2.68
Female	Barbados	5.23	5.94	4.60	12.12	14.39	10.10	3.72	4.26	3.24	5.73	6.85	4.71	1.58	1.41	1.76
Female	Barbuda	1.08	1.26	0.92	3.63	4.32	3.02	3.86	4.51	3.30	6.46	7.63	5.44	1.84	1.41	2.26
Female	Belarus	280.98	323.45	247.21	471.84	574.18	390.65	4.28	4.90	3.76	5.98	7.33	4.96	1.15	0.82	1.48
Female	Belgium	452.64	507.74	401.40	406.72	481.23	343.91	6.22	7.08	5.47	4.71	5.62	3.95	-0.95	-1.29	-0.62

© 2020 Deng Y et al. *JAMA Network Open.*

Sex	Location	Incident cases						Age-standardized incidence rate (per 100,000)						EAPC		
		1990(95%UI)			2017(95%UI)			1990(95%UI)			2017(95%UI)			1990-2017(95%CI)		
		Cases (No.)	UL	LL	Cases (No.)	UL	LL	95%UI	UL	LL	95%UI	UL	LL	EAPC	LL	UL
Female	Belize	0.71	0.82	0.61	3.69	4.46	3.08	1.33	1.53	1.14	2.22	2.67	1.87	2.09	1.97	2.21
Female	Benin	10.14	13.59	6.39	23.45	31.18	16.26	0.72	0.96	0.47	0.71	0.94	0.50	-0.19	-0.27	-0.11
Female	Bermuda	1.71	1.96	1.48	2.69	3.29	2.19	4.79	5.47	4.12	4.94	6.05	4.00	-0.27	-0.45	-0.08
Female	Bhutan	3.64	6.21	2.14	12.34	21.02	7.25	1.89	3.27	1.16	2.88	4.80	1.82	1.48	1.34	1.61
Female	Bolivia	75.53	107.18	43.53	292.20	397.47	200.39	3.66	5.11	2.18	5.93	8.08	4.04	1.71	1.55	1.87
Female	Bosnia and Herzegovina	59.05	71.11	41.61	124.77	153.27	85.84	2.35	2.82	1.68	4.83	5.98	3.29	3.03	2.78	3.28
Female	Botswana	4.43	7.00	2.97	17.73	25.37	12.75	1.14	1.73	0.79	1.86	2.68	1.37	2.89	2.04	3.76
Female	Brazil	1315.84	1390.17	1249.19	3748.80	3952.83	3538.11	2.32	2.45	2.21	2.99	3.14	2.82	0.88	0.68	1.08
Female	Brunei	4.36	5.81	3.17	21.20	26.03	16.25	6.19	8.00	4.68	10.04	12.15	7.86	2.56	2.24	2.88
Female	Bulgaria	201.80	226.44	175.14	231.72	269.43	197.48	3.46	3.90	3.00	4.40	5.19	3.69	0.76	0.58	0.93
Female	Burkina Faso	28.82	40.18	17.33	51.26	67.00	38.41	0.97	1.34	0.59	0.81	1.03	0.60	-0.87	-0.99	-0.75
Female	Burundi	36.11	51.00	23.34	60.57	83.40	42.85	2.00	2.78	1.33	1.83	2.55	1.28	-0.41	-0.50	-0.32
Female	Cambodia	87.29	125.93	48.07	223.07	309.10	162.63	2.71	3.87	1.56	3.05	4.21	2.23	0.28	0.14	0.42
Female	Cameroon	35.28	45.50	20.73	71.99	101.40	50.64	1.12	1.44	0.69	0.89	1.25	0.61	-0.92	-1.02	-0.82
Female	Canada	858.38	963.04	759.21	1616.29	1886.28	1367.22	5.28	5.94	4.65	6.33	7.41	5.32	0.49	0.13	0.84
Female	Cape Verde	0.82	1.09	0.46	3.25	4.31	1.71	0.65	0.88	0.34	1.26	1.68	0.65	2.77	2.50	3.05
Female	Central African Republic	8.81	13.70	5.51	12.15	21.01	7.26	1.14	1.71	0.78	0.90	1.50	0.57	-0.83	-0.91	-0.74
Female	Chad	12.64	17.12	7.90	22.28	29.65	16.12	0.69	0.92	0.45	0.65	0.85	0.47	-0.29	-0.37	-0.21
Female	Chile	199.97	227.06	176.39	683.72	831.15	560.19	3.31	3.76	2.91	5.95	7.25	4.86	2.25	2.16	2.35
Female	China	8390.10	9412.36	6870.87	25186.34	30434.73	22437.97	1.62	1.83	1.36	2.63	3.18	2.34	1.51	1.13	1.88
Female	Colombia	464.15	524.17	416.45	1574.08	1948.99	1298.72	4.04	4.52	3.64	5.54	6.86	4.56	1.13	0.85	1.40

© 2020 Deng Y et al. *JAMA Network Open.*

Sex	Location	Incident cases						Age-standardized incidence rate (per 100,000)						EAPC		
		1990(95%UI)			2017(95%UI)			1990(95%UI)			2017(95%UI)			1990-2017(95%CI)		
		Cases (No.)	UL	LL	Cases (No.)	UL	LL	95%UI	UL	LL	95%UI	UL	LL	EAPC	LL	UL
Female	Comoros	2.81	4.20	1.83	6.58	9.10	4.71	1.78	2.67	1.18	2.12	2.95	1.53	0.95	0.77	1.14
Female	Costa Rica	57.67	65.53	50.31	162.28	191.09	136.18	4.90	5.51	4.30	6.04	7.10	5.07	0.29	-0.08	0.65
Female	Croatia	216.26	247.62	185.37	195.55	229.02	164.45	6.08	6.93	5.24	5.25	6.17	4.43	0.40	0.03	0.78
Female	Cuba	187.69	216.59	165.03	450.23	546.83	365.17	3.49	4.03	3.07	5.44	6.60	4.43	1.64	1.30	1.99
Female	Cyprus	17.47	21.90	10.38	34.89	48.16	27.32	4.09	5.12	2.40	3.93	5.58	3.09	-0.53	-1.08	0.02
Female	Czech Republic	453.51	502.31	402.50	493.86	580.62	420.96	6.54	7.30	5.78	5.87	6.95	4.96	-0.56	-0.80	-0.31
Female	Democratic Republic of the Congo	100.19	149.33	70.03	209.39	339.40	141.70	0.94	1.36	0.68	0.92	1.47	0.62	-0.26	-0.46	-0.06
Female	Denmark	136.31	154.60	121.27	225.03	264.64	190.16	4.16	4.73	3.65	5.46	6.48	4.59	1.00	0.81	1.18
Female	Djibouti	2.43	3.77	1.49	9.19	14.29	5.71	1.75	2.69	1.11	2.13	3.28	1.35	0.73	0.58	0.89
Female	Dominica	0.76	0.86	0.67	1.44	1.68	1.23	2.20	2.52	1.91	3.66	4.32	3.10	1.91	1.70	2.12
Female	Dominican Republic	39.10	47.30	29.01	166.19	218.41	121.18	1.62	1.96	1.18	3.26	4.27	2.37	3.08	2.47	3.69
Female	Ecuador	65.54	73.54	58.41	682.32	828.04	557.14	1.95	2.15	1.74	8.54	10.38	7.00	6.12	5.46	6.79
Female	Egypt	266.77	443.93	204.61	1281.79	2060.66	866.18	1.35	2.38	1.04	3.14	5.19	2.13	3.32	3.12	3.53
Female	El Salvador	75.70	92.19	34.85	133.88	195.90	95.86	4.21	5.09	1.94	4.10	6.01	2.94	-0.03	-0.83	0.78
Female	Equatorial Guinea	1.42	2.28	0.82	7.11	12.70	3.71	1.10	1.73	0.69	1.86	3.26	1.02	2.43	2.14	2.72
Female	Eritrea	11.94	17.95	7.62	43.65	63.38	28.34	1.49	2.20	1.00	2.13	3.02	1.44	0.91	0.78	1.04
Female	Estonia	57.95	65.51	51.07	85.52	106.65	66.34	5.66	6.48	4.97	7.91	10.07	6.09	1.41	1.11	1.72
Female	Ethiopia	802.47	1194.73	408.75	1500.71	1989.88	1115.70	5.12	7.48	2.80	4.39	5.74	3.30	-0.91	-1.07	-0.75
Female	Fiji	12.29	16.47	6.03	27.79	37.50	12.82	5.20	6.98	2.56	6.41	8.54	3.01	0.73	0.47	0.99
Female	Finland	149.46	166.77	133.07	244.20	284.08	205.80	3.95	4.42	3.52	4.81	5.61	4.06	0.95	0.76	1.14

© 2020 Deng Y et al. *JAMA Network Open.*

Sex	Location	Incident cases						Age-standardized incidence rate (per 100,000)						EAPC		
		1990(95%UI)			2017(95%UI)			1990(95%UI)			2017(95%UI)			1990-2017(95%CI)		
		Cases (No.)	UL	LL	Cases (No.)	UL	LL	95%UI	UL	LL	95%UI	UL	LL	EAPC	LL	UL
Female	France	1858.03	2037.79	1685.58	2102.80	2454.37	1786.60	4.85	5.35	4.38	4.13	4.87	3.50	-0.61	-1.01	-0.20
Female	Gabon	4.27	6.34	2.89	8.11	13.75	5.27	1.28	1.91	0.87	1.23	2.07	0.80	-0.11	-0.20	-0.02
Female	Gambia	2.15	2.76	1.62	6.66	9.30	5.00	0.81	1.04	0.63	0.99	1.38	0.73	0.91	0.79	1.03
Female	Georgia	78.81	92.77	63.62	131.24	153.12	110.31	2.32	2.72	1.86	5.05	5.89	4.21	3.28	2.19	4.38
Female	Germany	3336.61	3659.86	3025.25	4329.62	5201.49	3564.12	5.80	6.43	5.20	6.66	8.06	5.42	0.43	0.24	0.62
Female	Ghana	5.62	7.12	4.25	21.44	26.95	16.65	0.11	0.14	0.08	0.17	0.21	0.13	1.47	1.37	1.58
Female	Greece	231.30	259.98	204.62	361.26	416.89	307.73	3.23	3.63	2.85	4.17	4.85	3.55	0.89	0.65	1.13
Female	Greenland	0.43	0.54	0.35	1.10	1.44	0.56	2.15	2.56	1.70	3.37	4.30	1.78	2.05	1.42	2.69
Female	Grenada	1.72	1.96	1.49	2.69	3.12	2.29	4.80	5.50	4.09	4.28	5.03	3.61	0.24	-0.31	0.79
Female	Grenadines	1.82	2.13	1.58	4.05	4.77	3.41	4.54	5.32	3.90	6.40	7.56	5.41	1.40	1.05	1.75
Female	Guam	1.14	1.66	0.91	4.28	5.23	3.33	2.37	3.38	1.92	4.70	5.73	3.70	2.68	1.75	3.62
Female	Guatemala	43.06	48.53	38.34	197.09	237.85	159.39	1.86	2.08	1.67	2.90	3.49	2.36	0.13	-0.49	0.74
Female	Guinea	18.54	22.74	14.67	37.80	50.59	28.38	0.87	1.06	0.71	0.99	1.29	0.76	0.55	0.48	0.61
Female	Guinea-Bissau	2.90	3.93	1.57	4.29	6.20	2.83	0.98	1.30	0.56	0.81	1.17	0.55	-0.86	-1.02	-0.71
Female	Guyana	4.08	4.63	3.57	9.64	11.96	7.76	1.57	1.76	1.38	2.63	3.25	2.13	1.85	1.49	2.20
Female	Haiti	40.80	70.01	20.28	101.60	164.42	61.57	2.02	3.41	1.06	2.24	3.59	1.41	0.36	0.27	0.44
Female	Honduras	18.59	23.03	14.89	59.92	127.27	36.43	1.47	1.77	1.20	1.69	3.48	1.05	0.56	0.14	0.99
Female	Hungary	424.15	482.03	375.38	390.99	456.79	335.53	5.68	6.48	5.00	4.85	5.73	4.14	-0.51	-0.73	-0.29
Female	Iceland	21.08	23.86	18.29	24.12	28.01	20.61	15.64	17.76	13.49	10.87	12.70	9.27	-0.99	-1.31	-0.67
Female	India	5679.71	7529.92	4213.53	19152.28	21491.71	16040.84	1.69	2.25	1.29	2.95	3.30	2.48	1.96	1.74	2.17
Female	Indonesia	1673.18	2069.95	1141.81	3871.84	4879.65	2844.30	2.53	3.08	1.80	2.98	3.76	2.23	0.52	0.45	0.58
Female	Iran	381.47	484.48	298.02	2071.35	2398.72	1378.80	2.20	2.79	1.73	4.88	5.63	3.19	3.65	3.31	3.99
Female	Iraq	174.20	255.75	101.49	384.75	494.31	277.98	3.31	4.85	2.02	2.54	3.25	1.78	-1.16	-1.43	-0.88

© 2020 Deng Y et al. *JAMA Network Open.*

Sex	Location	Incident cases						Age-standardized incidence rate (per 100,000)						EAPC		
		1990(95%UI)			2017(95%UI)			1990(95%UI)			2017(95%UI)			1990-2017(95%CI)		
		Cases (No.)	UL	LL	Cases (No.)	UL	LL	95%UI	UL	LL	95%UI	UL	LL	EAPC	LL	UL
Female	Ireland	81.46	93.04	72.12	177.75	212.09	147.93	4.20	4.85	3.68	5.56	6.68	4.62	1.54	1.30	1.78
Female	Israel	92.16	103.65	81.85	304.06	360.46	256.27	3.68	4.14	3.25	5.86	7.00	4.92	1.55	1.07	2.03
Female	Italy	3935.47	4376.13	3524.88	4376.37	5133.55	3684.26	10.03	11.26	8.91	8.81	10.46	7.33	-0.41	-0.81	-0.01
Female	Ivory Coast	34.53	44.70	25.56	87.79	125.64	61.04	1.00	1.26	0.78	1.08	1.48	0.79	0.39	0.34	0.45
Female	Jamaica	17.96	20.59	15.53	83.10	109.37	63.66	1.86	2.15	1.60	5.53	7.29	4.24	4.21	3.70	4.72
Female	Japan	4761.47	5056.25	4494.70	8233.16	9050.75	7556.02	5.40	5.77	5.09	6.68	7.46	6.07	0.90	0.70	1.10
Female	Jordan	51.86	70.26	33.82	169.93	244.36	123.40	4.70	6.28	3.21	4.49	6.35	3.29	-0.86	-1.50	-0.20
Female	Kazakhstan	484.17	591.74	413.10	549.56	657.64	462.52	5.92	7.22	5.03	5.35	6.40	4.50	-1.21	-1.69	-0.74
Female	Kenya	44.97	62.94	34.28	153.68	202.22	122.76	0.68	0.91	0.53	0.86	1.12	0.70	0.88	0.81	0.95
Female	Kiribati	0.17	0.20	0.14	0.34	0.44	0.25	0.74	0.88	0.60	0.80	1.03	0.60	0.42	0.34	0.49
Female	Kuwait	35.31	41.53	29.43	77.18	97.54	62.07	7.14	8.22	6.13	4.30	5.29	3.58	-1.93	-2.73	-1.13
Female	Kyrgyzstan	63.92	74.74	53.64	77.80	93.65	66.05	3.38	3.95	2.85	2.70	3.23	2.31	-0.41	-0.69	-0.12
Female	Laos	37.78	60.65	18.89	76.62	111.73	49.55	2.87	4.50	1.51	2.83	4.04	1.87	-0.35	-0.52	-0.18
Female	Latvia	90.25	102.20	79.35	115.75	146.85	91.43	5.06	5.78	4.37	6.47	8.23	5.11	0.96	0.64	1.27
Female	Lebanon	94.75	128.16	60.13	609.54	829.14	450.63	5.86	7.88	3.89	14.92	19.83	11.23	3.71	3.62	3.81
Female	Lesotho	6.55	9.17	4.87	10.86	17.21	6.74	1.10	1.55	0.82	1.42	2.23	0.88	1.64	1.33	1.95
Female	Liberia	5.85	7.76	3.54	9.98	13.93	6.60	0.91	1.20	0.56	0.73	1.02	0.49	-0.95	-1.20	-0.69
Female	Libya	52.79	72.39	37.99	331.08	455.43	236.91	4.09	5.63	3.02	9.63	13.10	7.01	3.98	3.59	4.37
Female	Lithuania	137.37	154.27	120.97	158.99	187.87	132.38	5.90	6.67	5.14	6.61	7.84	5.51	0.37	0.02	0.73
Female	Luxembourg	19.68	22.54	17.26	31.69	40.35	25.23	7.39	8.50	6.45	7.50	9.52	5.97	-0.22	-0.44	0.00
Female	Macedonia	32.29	40.28	19.02	60.62	76.17	42.67	3.01	3.73	1.80	4.13	5.20	2.91	0.85	0.62	1.09
Female	Madagascar	67.94	96.11	47.33	153.37	220.29	105.37	1.69	2.37	1.22	1.74	2.44	1.21	0.12	-0.04	0.29
Female	Malawi	64.65	91.18	28.26	154.75	226.00	99.70	1.96	2.68	1.02	2.44	3.43	1.65	0.35	0.11	0.59

© 2020 Deng Y et al. *JAMA Network Open.*

Sex	Location	Incident cases						Age-standardized incidence rate (per 100,000)						EAPC		
		1990(95%UI)			2017(95%UI)			1990(95%UI)			2017(95%UI)			1990-2017(95%CI)		
		Cases (No.)	UL	LL	Cases (No.)	UL	LL	95%UI	UL	LL	95%UI	UL	LL	EAPC	LL	UL
Female	Malaysia	236.87	289.07	187.09	1120.51	1485.92	845.82	3.76	4.52	3.09	7.68	10.25	5.84	2.71	2.59	2.84
Female	Maldives	1.60	2.61	0.60	6.51	8.35	5.03	3.14	5.06	1.21	3.99	5.06	3.04	0.47	0.31	0.63
Female	Mali	50.11	61.22	38.71	127.56	194.40	81.99	1.73	2.09	1.42	2.01	2.94	1.33	0.35	0.26	0.45
Female	Malta	12.33	13.96	10.83	24.13	28.44	20.77	5.38	6.09	4.72	7.03	8.26	6.03	1.08	0.77	1.38
Female	Marshall Islands	0.27	0.37	0.18	0.94	1.46	0.53	2.55	3.51	1.76	4.27	6.38	2.56	1.92	1.67	2.16
Female	Mauritania	6.09	7.95	3.47	11.83	16.39	8.16	0.93	1.22	0.54	0.93	1.29	0.64	-0.20	-0.35	-0.06
Female	Mauritius	9.91	11.48	8.58	31.69	37.99	26.11	2.16	2.48	1.88	3.65	4.37	3.03	2.33	1.77	2.90
Female	Mexico	755.62	785.47	725.69	3712.39	3889.08	3538.48	2.76	2.86	2.65	5.68	5.94	5.42	2.42	2.21	2.63
Female	Micronesia	0.59	0.83	0.42	1.26	1.90	0.77	2.14	2.93	1.57	2.98	4.35	1.91	1.22	1.11	1.33
Female	Moldova	82.17	95.16	70.02	104.58	124.44	88.14	3.13	3.63	2.68	3.69	4.37	3.10	0.90	0.55	1.24
Female	Mongolia	7.15	8.50	5.07	26.16	34.10	15.38	1.16	1.39	0.76	1.87	2.45	0.96	2.21	1.88	2.55
Female	Montenegro	16.48	20.63	13.10	30.87	38.66	24.42	4.76	5.92	3.81	6.88	8.61	5.46	1.50	1.38	1.62
Female	Morocco	351.63	449.07	245.53	1214.17	1699.19	847.33	3.56	4.45	2.71	6.60	9.12	4.63	2.37	2.29	2.45
Female	Mozambique	74.85	113.09	52.67	196.37	283.42	123.92	1.49	2.30	1.07	1.95	2.79	1.29	0.94	0.81	1.08
Female	Myanmar	518.98	795.86	274.33	1051.15	1486.51	706.92	3.55	5.36	1.98	3.77	5.32	2.57	0.03	-0.06	0.13
Female	Namibia	5.83	8.23	3.76	15.39	21.09	10.79	1.22	1.81	0.80	1.54	2.15	1.09	0.50	-0.05	1.06
Female	Nepal	119.84	204.24	75.57	347.42	558.61	231.98	1.74	3.00	1.13	2.45	3.92	1.67	1.35	0.97	1.73
Female	Netherlands	423.08	469.44	375.29	837.64	991.20	702.17	4.42	4.94	3.89	6.25	7.33	5.28	1.48	1.28	1.68
Female	New Zealand	91.80	106.66	79.37	179.53	210.69	151.88	4.68	5.45	4.03	5.75	6.71	4.91	1.17	0.95	1.40
Female	Nicaragua	17.48	25.11	14.23	99.62	125.35	75.07	1.59	2.34	1.31	3.56	4.47	2.68	3.66	3.17	4.15
Female	Niger	14.12	18.75	8.47	28.43	38.77	20.20	0.69	0.88	0.44	0.57	0.76	0.41	-0.95	-1.08	-0.83
Female	Nigeria	119.28	175.72	65.48	350.73	528.29	221.63	0.51	0.76	0.27	0.62	0.92	0.40	0.74	0.66	0.81
Female	North Korea	273.11	380.17	193.99	458.35	696.90	312.62	2.54	3.56	1.81	2.87	4.37	1.96	0.37	0.19	0.54

© 2020 Deng Y et al. *JAMA Network Open.*

Sex	Location	Incident cases						Age-standardized incidence rate (per 100,000)						EAPC		
		1990(95%UI)			2017(95%UI)			1990(95%UI)			2017(95%UI)			1990-2017(95%CI)		
		Cases (No.)	UL	LL	Cases (No.)	UL	LL	95%UI	UL	LL	95%UI	UL	LL	EAPC	LL	UL
Female	Northern Mariana Islands	0.39	0.54	0.27	1.10	1.46	0.84	2.96	3.89	2.21	3.57	4.60	2.82	0.49	-0.10	1.09
Female	Norway	142.22	154.59	130.65	252.98	283.25	225.76	5.21	5.73	4.75	6.94	7.79	6.18	1.16	0.85	1.46
Female	Oman	18.16	25.23	11.52	98.53	130.42	69.79	3.70	5.04	2.47	7.26	9.58	5.38	2.56	2.33	2.78
Female	Pakistan	1879.50	2627.58	1425.17	7948.92	12615.31	5059.13	4.85	6.53	3.80	8.47	12.86	5.45	2.10	1.95	2.25
Female	Palestine	15.34	21.03	10.79	41.85	53.51	34.06	2.65	3.59	1.87	2.72	3.32	2.21	0.15	-0.02	0.32
Female	Panama	29.18	33.08	25.46	107.69	127.52	90.27	3.03	3.41	2.68	5.33	6.30	4.47	2.09	1.94	2.24
Female	Papua New Guinea	23.80	36.57	16.22	80.57	122.44	52.40	2.09	3.11	1.46	2.68	3.93	1.87	0.93	0.75	1.11
Female	Paraguay	27.43	33.14	22.94	116.02	159.60	76.58	2.06	2.47	1.69	3.89	5.33	2.54	2.36	2.12	2.60
Female	Peru	190.44	237.12	158.03	896.74	1168.12	641.36	2.54	3.12	2.11	5.54	7.19	3.95	3.10	2.69	3.50
Female	Philippines	871.14	1045.42	739.24	3914.13	4967.91	3065.18	4.30	5.08	3.71	8.79	11.12	6.92	2.95	2.76	3.13
Female	Poland	1302.91	1436.88	1174.05	1933.16	2244.72	1635.39	5.54	6.14	4.97	6.61	7.80	5.57	0.38	0.12	0.64
Female	Portugal	398.91	446.57	352.10	556.28	659.93	464.47	5.60	6.29	4.96	5.64	6.77	4.71	-0.48	-0.79	-0.17
Female	Puerto Rico	86.57	99.68	75.06	94.80	113.30	79.72	4.46	5.14	3.86	3.60	4.34	3.00	-0.88	-1.28	-0.48
Female	Qatar	7.36	10.84	2.10	22.51	31.93	16.17	9.59	13.35	2.91	4.67	6.55	3.38	-3.34	-4.01	-2.67
Female	Republic of Congo	9.72	15.38	5.67	25.86	45.71	15.18	1.36	2.09	0.86	1.50	2.57	0.95	0.35	0.16	0.53
Female	Romania	497.33	558.25	438.63	856.28	997.44	719.74	3.50	3.92	3.08	5.86	6.83	4.94	1.66	1.42	1.91
Female	Russia	4251.69	4848.00	3807.59	8336.47	9092.32	7643.64	4.13	4.73	3.69	7.66	8.35	6.95	2.54	2.15	2.94
Female	Rwanda	48.41	71.52	27.11	95.47	144.38	68.33	2.01	2.98	1.15	1.96	2.93	1.44	-0.34	-0.65	-0.02
Female	Saint Lucia	2.47	2.80	2.18	7.08	8.42	5.90	4.86	5.53	4.23	6.55	7.74	5.49	0.93	0.49	1.37
Female	Saint Vincent	1.82	2.13	1.58	4.05	4.77	3.41	4.54	5.32	3.90	6.40	7.56	5.41	1.40	1.05	1.75

© 2020 Deng Y et al. *JAMA Network Open.*

Sex	Location	Incident cases						Age-standardized incidence rate (per 100,000)						EAPC		
		1990(95%UI)			2017(95%UI)			1990(95%UI)			2017(95%UI)			1990-2017(95%CI)		
		Cases (No.)	UL	LL	Cases (No.)	UL	LL	95%UI	UL	LL	95%UI	UL	LL	EAPC	LL	UL
Female	Samoa	1.95	2.53	1.43	4.02	5.56	2.71	3.52	4.60	2.60	5.10	7.04	3.46	1.30	1.12	1.48
Female	Sao Tome and Principe	0.34	0.54	0.21	0.75	1.03	0.55	0.79	1.24	0.50	1.05	1.40	0.76	0.63	0.40	0.86
Female	Saudi Arabia	145.80	201.13	106.06	1827.39	2602.65	1207.27	3.32	4.59	2.43	12.12	16.37	8.81	5.66	5.23	6.08
Female	Senegal	15.80	20.57	9.87	30.43	40.05	22.84	0.71	0.91	0.46	0.66	0.86	0.49	-0.62	-0.78	-0.46
Female	Serbia	219.16	274.17	165.94	321.21	384.75	240.73	3.75	4.65	2.88	4.86	5.87	3.70	1.30	1.15	1.46
Female	Seychelles	0.46	0.55	0.30	1.26	1.53	1.01	1.52	1.83	0.97	2.18	2.65	1.75	1.49	1.35	1.63
Female	Sierra Leone	9.85	13.88	6.13	19.96	27.52	13.99	0.79	1.10	0.50	0.87	1.19	0.60	0.39	0.29	0.49
Female	Singapore	65.82	75.69	57.03	206.75	252.76	169.65	4.31	4.92	3.77	5.76	7.06	4.73	1.61	1.34	1.88
Female	Slovakia	151.43	176.56	123.32	204.63	275.99	163.23	4.82	5.61	3.90	4.83	6.63	3.83	-0.19	-0.34	-0.04
Female	Slovenia	70.42	79.69	61.94	83.56	100.16	69.14	5.41	6.12	4.74	5.17	6.23	4.25	-0.22	-0.44	-0.01
Female	Solomon Islands	1.54	2.33	1.04	4.67	6.94	3.09	1.92	2.80	1.39	2.28	3.30	1.61	0.47	0.29	0.65
Female	Somalia	28.87	51.02	12.36	80.19	117.54	55.37	1.44	2.41	0.75	1.61	2.33	1.10	0.23	0.06	0.39
Female	South Africa	221.21	251.58	179.44	382.04	504.17	331.97	1.51	1.71	1.26	1.39	1.82	1.21	-0.88	-1.47	-0.29
Female	South Korea	421.88	476.50	366.04	6573.02	7953.50	5505.40	2.06	2.31	1.80	19.85	24.29	16.41	11.05	9.06	13.08
Female	South Sudan	29.01	52.77	12.77	53.67	82.79	33.67	1.69	2.92	0.86	1.79	2.76	1.16	0.16	-0.02	0.33
Female	Spain	870.74	962.83	790.42	1567.67	1827.27	1344.91	3.36	3.74	3.02	4.16	4.89	3.56	0.91	0.66	1.16
Female	Sri Lanka	219.90	267.11	155.12	700.77	988.18	472.05	3.35	4.04	2.36	5.20	7.30	3.54	1.15	0.76	1.54
Female	Sudan	97.07	169.43	51.11	303.41	458.57	183.84	1.60	2.75	0.92	2.37	3.50	1.46	1.50	1.40	1.61
Female	Suriname	3.79	4.38	3.28	12.18	14.68	10.03	2.50	2.88	2.17	3.84	4.61	3.16	1.67	1.35	2.00
Female	Swaziland	3.15	4.50	2.28	6.01	9.67	3.64	1.49	2.12	1.08	1.55	2.54	0.93	0.25	-0.04	0.55
Female	Sweden	213.08	235.21	192.53	313.63	362.22	272.82	3.34	3.73	3.00	4.27	4.91	3.67	0.95	0.71	1.19
Female	Switzerland	321.22	362.55	278.63	371.97	447.91	313.99	7.25	8.23	6.22	5.63	6.83	4.74	-0.83	-1.14	-0.51

© 2020 Deng Y et al. *JAMA Network Open.*

Sex	Location	Incident cases						Age-standardized incidence rate (per 100,000)						EAPC		
		1990(95%UI)			2017(95%UI)			1990(95%UI)			2017(95%UI)			1990-2017(95%CI)		
		Cases (No.)	UL	LL	Cases (No.)	UL	LL	95%UI	UL	LL	95%UI	UL	LL	EAPC	LL	UL
Female	Syria	28.71	40.58	17.26	112.38	154.86	76.82	0.88	1.24	0.52	1.45	1.97	0.99	1.64	1.46	1.82
Female	Tajikistan	7.78	9.90	6.46	22.55	28.07	18.40	0.45	0.57	0.37	0.65	0.79	0.53	0.97	0.62	1.33
Female	Tanzania	151.90	220.72	91.10	415.21	606.25	288.72	1.78	2.60	1.18	2.13	3.06	1.52	0.32	0.04	0.60
Female	Thailand	663.09	1073.40	531.59	2105.37	4859.93	1471.29	2.86	4.52	2.32	4.10	9.60	2.84	0.96	0.47	1.44
Female	Timor-Leste	4.41	6.49	2.52	12.22	16.70	8.12	2.21	3.13	1.43	2.82	3.85	1.94	1.00	0.74	1.27
Female	Tobago	14.66	16.73	12.75	34.30	47.01	24.12	2.95	3.34	2.57	3.92	5.43	2.73	0.45	-0.03	0.93
Female	Togo	8.57	12.24	5.24	17.61	23.40	12.74	0.82	1.15	0.52	0.67	0.89	0.49	-0.93	-1.15	-0.72
Female	Tonga	0.52	0.67	0.41	1.10	1.47	0.79	1.68	2.14	1.32	2.52	3.37	1.81	1.54	1.35	1.73
Female	Trinidad	14.66	16.73	12.75	34.30	47.01	24.12	2.95	3.34	2.57	3.92	5.43	2.73	0.45	-0.03	0.93
Female	Tunisia	96.89	119.81	78.24	381.37	536.40	260.12	2.92	3.66	2.39	5.85	8.22	4.02	2.44	2.15	2.74
Female	Turkey	563.67	892.39	358.90	2187.05	3547.43	1690.42	2.62	4.04	1.69	4.74	7.69	3.66	2.58	2.30	2.86
Female	Turkmenistan	25.38	28.94	22.10	61.83	73.50	51.05	1.97	2.24	1.71	2.52	3.00	2.10	0.94	0.23	1.66
Female	Uganda	89.42	116.58	68.79	264.36	376.34	181.21	1.71	2.21	1.34	2.06	2.83	1.45	0.67	0.54	0.81
Female	UK	1238.02	1292.88	1178.63	2194.36	2307.33	2093.25	3.13	3.27	2.98	4.58	4.82	4.37	1.41	1.26	1.56
Female	Ukraine	1174.79	1398.12	995.29	1924.50	2242.56	1646.88	3.02	3.56	2.56	5.54	6.52	4.71	2.32	1.95	2.69
Female	United Arab Emirates	12.20	18.83	7.42	149.15	227.93	92.47	3.69	5.92	2.29	6.45	9.99	3.88	2.27	1.94	2.60
Female	Uruguay	37.39	42.78	32.95	89.83	110.43	71.82	2.00	2.31	1.75	4.02	4.99	3.18	2.04	1.39	2.70
Female	USA	6796.91	7037.91	6569.50	15491.78	16328.45	14684.06	4.32	4.48	4.16	6.47	6.84	6.10	1.49	1.22	1.76
Female	Uzbekistan	39.94	55.08	33.42	178.56	219.41	141.87	0.54	0.75	0.45	1.18	1.45	0.94	3.20	3.01	3.39
Female	Vanuatu	0.81	1.30	0.51	2.43	4.28	1.51	2.10	3.35	1.40	2.57	4.42	1.64	0.78	0.72	0.84
Female	Venezuela	132.64	151.81	117.52	709.69	899.01	534.07	2.09	2.35	1.86	4.44	5.63	3.34	2.78	2.47	3.10
Female	Vietnam	1056.30	1351.67	835.79	5310.83	7306.41	3868.66	3.89	5.21	3.05	9.65	13.26	7.03	4.07	3.74	4.40

© 2020 Deng Y et al. *JAMA Network Open.*

Sex	Location	Incident cases						Age-standardized incidence rate (per 100,000)						EAPC		
		1990(95%UI)			2017(95%UI)			1990(95%UI)			2017(95%UI)			1990-2017(95%CI)		
		Cases (No.)	UL	LL	Cases (No.)	UL	LL	95%UI	UL	LL	95%UI	UL	LL	EAPC	LL	UL
Female	Virgin Islands	0.95	1.13	0.79	2.45	3.01	1.96	1.73	2.04	1.47	3.07	3.82	2.44	2.58	2.33	2.83
Female	Yemen	49.85	94.84	24.34	212.41	337.04	130.12	1.43	2.65	0.77	2.22	3.45	1.40	1.82	1.74	1.90
Female	Zambia	60.05	88.37	35.94	109.46	155.81	77.55	2.44	3.49	1.56	1.93	2.72	1.38	-1.29	-1.95	-0.62
Female	Zimbabwe	65.93	83.70	43.33	133.61	180.53	74.53	2.28	2.85	1.65	2.58	3.43	1.68	0.82	0.33	1.32
Male	Afghanistan	24.89	44.87	9.91	51.28	82.63	32.81	0.64	1.16	0.26	0.74	1.13	0.50	0.70	0.62	0.77
Male	Albania	15.27	17.86	13.02	36.81	48.56	27.13	1.21	1.40	1.04	2.14	2.82	1.57	2.02	1.73	2.31
Male	Algeria	70.85	86.07	56.12	376.18	477.37	280.40	0.83	0.99	0.68	1.88	2.33	1.43	3.32	3.17	3.46
Male	American Samoa	0.12	0.14	0.09	0.28	0.35	0.23	0.78	0.95	0.63	1.17	1.42	0.94	1.67	0.95	2.39
Male	Andorra	0.63	0.84	0.46	1.69	2.36	1.20	1.93	2.55	1.42	2.68	3.71	1.92	1.33	1.03	1.63
Male	Angola	10.24	14.95	6.60	32.17	42.30	24.04	0.42	0.58	0.30	0.52	0.68	0.39	0.69	0.51	0.88
Male	Antigua	0.12	0.14	0.10	0.41	0.50	0.34	0.52	0.60	0.44	0.84	0.99	0.69	1.97	1.85	2.09
Male	Argentina	205.18	231.23	181.89	368.12	460.26	296.96	1.35	1.52	1.20	1.60	2.00	1.28	0.43	0.20	0.65
Male	Armenia	6.07	8.01	4.68	31.60	36.50	26.69	0.45	0.59	0.35	1.80	2.07	1.53	5.35	4.21	6.50
Male	Australia	180.45	208.37	154.15	608.43	752.76	482.74	1.96	2.26	1.69	3.84	4.76	3.04	3.05	2.88	3.22
Male	Austria	152.76	176.51	130.20	237.94	290.42	191.39	3.36	3.87	2.88	3.44	4.14	2.77	-0.66	-1.00	-0.33
Male	Azerbaijan	19.89	23.99	16.43	54.89	69.51	42.49	0.75	0.90	0.63	1.12	1.37	0.89	0.65	0.10	1.20
Male	Bahamas	1.27	1.51	1.07	4.51	5.60	3.60	1.41	1.67	1.18	2.26	2.77	1.83	2.30	2.09	2.52
Male	Bahrain	0.93	1.12	0.76	7.17	9.49	5.34	0.63	0.76	0.52	0.79	0.98	0.62	0.33	-0.01	0.66
Male	Bangladesh	182.93	232.33	139.40	562.07	786.51	387.17	0.56	0.72	0.44	0.79	1.08	0.55	1.38	1.27	1.50
Male	Barbados	1.29	1.49	1.11	3.48	4.22	2.88	1.08	1.25	0.93	1.80	2.19	1.48	2.05	1.74	2.37
Male	Barbuda	0.12	0.14	0.10	0.41	0.50	0.34	0.52	0.60	0.44	0.84	0.99	0.69	1.97	1.85	2.09
Male	Belarus	79.14	96.38	65.53	161.08	214.28	128.29	1.50	1.81	1.25	2.67	3.48	2.14	2.17	1.87	2.47
Male	Belgium	180.99	214.29	153.93	337.38	428.60	267.36	2.90	3.43	2.47	4.06	5.12	3.23	1.73	1.48	1.99

© 2020 Deng Y et al. *JAMA Network Open.*

Sex	Location	Incident cases						Age-standardized incidence rate (per 100,000)						EAPC		
		1990(95%UI)			2017(95%UI)			1990(95%UI)			2017(95%UI)			1990-2017(95%CI)		
		Cases (No.)	UL	LL	Cases (No.)	UL	LL	95%UI	UL	LL	95%UI	UL	LL	EAPC	LL	UL
Male	Belize	0.19	0.23	0.16	1.43	1.69	1.21	0.35	0.42	0.30	0.88	1.03	0.75	3.31	2.93	3.70
Male	Benin	3.96	4.84	3.23	10.58	14.76	7.31	0.36	0.44	0.30	0.42	0.58	0.29	0.60	0.54	0.67
Male	Bermuda	0.71	0.84	0.61	1.96	2.38	1.62	2.34	2.75	2.02	4.01	4.87	3.31	2.67	2.41	2.94
Male	Bhutan	1.00	1.29	0.75	4.28	6.33	2.64	0.59	0.75	0.45	1.02	1.49	0.65	2.33	2.23	2.42
Male	Bolivia	16.12	22.37	12.03	68.26	93.22	48.29	0.92	1.26	0.69	1.54	2.10	1.10	2.08	1.92	2.24
Male	Bosnia and Herzegovina	15.38	18.35	13.04	26.26	32.33	21.21	0.77	0.89	0.66	1.01	1.23	0.83	0.76	0.60	0.93
Male	Botswana	1.62	2.27	1.11	4.57	7.01	2.93	0.51	0.70	0.36	0.61	0.90	0.40	0.55	0.28	0.82
Male	Brazil	472.80	500.57	444.13	1627.18	1742.34	1509.36	0.94	0.99	0.89	1.48	1.58	1.38	1.83	1.66	2.00
Male	Brunei	1.08	1.37	0.85	5.14	6.32	4.13	1.40	1.77	1.13	2.51	3.01	2.07	2.82	2.56	3.08
Male	Bulgaria	79.02	91.47	68.34	91.61	111.71	74.96	1.39	1.61	1.20	1.72	2.10	1.41	0.49	0.35	0.63
Male	Burkina Faso	7.28	9.69	5.17	16.95	21.14	13.47	0.32	0.42	0.23	0.36	0.45	0.29	0.38	0.25	0.52
Male	Burundi	8.83	13.06	5.66	21.85	29.39	16.23	0.66	0.94	0.44	0.74	0.96	0.56	0.65	0.50	0.79
Male	Cambodia	20.75	30.14	14.57	58.93	76.83	45.25	0.92	1.32	0.66	1.12	1.43	0.88	0.73	0.56	0.90
Male	Cameroon	10.44	12.52	8.46	30.59	42.07	21.62	0.41	0.50	0.34	0.48	0.65	0.33	0.44	0.32	0.56
Male	Canada	288.62	333.70	250.17	769.29	928.44	629.28	1.92	2.22	1.67	3.04	3.69	2.50	1.98	1.72	2.24
Male	Cape Verde	0.29	0.39	0.21	1.09	1.48	0.71	0.30	0.41	0.22	0.48	0.65	0.32	1.75	1.66	1.85
Male	Central African Republic	2.95	4.59	1.81	5.29	8.25	3.38	0.45	0.68	0.30	0.43	0.62	0.30	-0.30	-0.35	-0.25
Male	Chad	4.42	5.65	3.26	10.63	13.50	8.16	0.29	0.37	0.22	0.33	0.41	0.25	0.38	0.26	0.50
Male	Chile	62.51	72.55	53.56	208.57	257.88	167.56	1.20	1.38	1.04	2.00	2.47	1.62	2.15	2.03	2.27
Male	China	2625.68	2845.14	2430.35	16324.40	17776.50	14919.65	0.54	0.58	0.50	1.72	1.87	1.57	5.17	4.69	5.65
Male	Colombia	128.17	155.79	112.11	339.53	423.71	271.07	1.20	1.39	1.07	1.35	1.69	1.08	0.19	-0.13	0.50

© 2020 Deng Y et al. *JAMA Network Open.*

Sex	Location	Incident cases						Age-standardized incidence rate (per 100,000)						EAPC		
		1990(95%UI)			2017(95%UI)			1990(95%UI)			2017(95%UI)			1990-2017(95%CI)		
		Cases (No.)	UL	LL	Cases (No.)	UL	LL	95%UI	UL	LL	95%UI	UL	LL	EAPC	LL	UL
Male	Comoros	0.79	1.00	0.60	2.19	3.19	1.55	0.60	0.76	0.46	0.84	1.20	0.60	1.53	1.28	1.79
Male	Costa Rica	14.96	17.28	13.00	51.28	62.06	42.16	1.37	1.56	1.21	2.14	2.57	1.76	1.64	1.24	2.04
Male	Croatia	71.43	83.77	60.05	64.27	78.27	52.97	2.49	2.88	2.13	1.97	2.38	1.63	-0.23	-0.64	0.17
Male	Cuba	55.88	65.77	48.07	179.83	224.78	142.34	1.06	1.25	0.91	2.23	2.79	1.76	3.04	2.80	3.28
Male	Cyprus	6.66	8.31	5.53	28.70	37.07	21.98	1.68	2.10	1.40	3.48	4.54	2.67	3.27	2.91	3.62
Male	Czech Republic	148.95	174.07	127.41	176.10	217.27	144.78	2.55	2.95	2.20	2.21	2.70	1.82	-0.21	-0.40	-0.01
Male	Democratic Republic of the Congo	31.51	41.85	22.76	74.81	101.86	52.07	0.37	0.48	0.27	0.40	0.54	0.27	0.14	-0.04	0.32
Male	Denmark	39.97	47.18	33.68	93.82	116.92	75.88	1.24	1.47	1.05	2.22	2.73	1.80	2.30	1.89	2.72
Male	Djibouti	0.74	1.09	0.47	4.16	6.42	2.60	0.55	0.78	0.39	0.98	1.44	0.63	2.27	2.13	2.41
Male	Dominica	0.27	0.30	0.23	0.75	0.89	0.63	0.91	1.04	0.79	1.79	2.12	1.50	2.77	2.66	2.87
Male	Dominican Republic	12.44	14.73	10.42	60.25	81.23	43.97	0.56	0.66	0.48	1.23	1.64	0.90	2.22	1.55	2.89
Male	Ecuador	17.76	19.98	15.70	139.25	170.24	114.22	0.57	0.63	0.51	1.85	2.23	1.52	4.94	3.98	5.91
Male	Egypt	81.64	96.85	66.02	416.31	567.92	300.29	0.43	0.50	0.36	1.00	1.34	0.74	3.38	3.13	3.63
Male	El Salvador	12.06	13.94	10.57	24.39	32.32	18.12	0.80	0.92	0.70	0.99	1.31	0.73	0.72	0.25	1.19
Male	Equatorial Guinea	0.48	0.76	0.29	1.92	2.84	1.18	0.48	0.72	0.31	0.68	0.97	0.41	1.36	0.99	1.74
Male	Eritrea	5.67	8.42	2.96	22.64	33.69	15.50	0.86	1.22	0.54	1.34	1.91	0.93	1.33	0.84	1.82
Male	Estonia	13.76	16.23	11.63	21.81	28.73	16.76	1.69	2.00	1.44	2.49	3.28	1.92	1.12	0.69	1.55
Male	Ethiopia	297.10	507.25	160.58	626.96	782.73	493.39	2.26	3.72	1.33	2.23	2.80	1.74	-0.13	-0.32	0.06
Male	Fiji	4.79	6.12	3.68	6.42	8.04	5.07	1.92	2.45	1.45	1.59	1.95	1.28	-1.28	-1.74	-0.81
Male	Finland	60.66	71.16	52.60	150.66	185.94	122.05	2.06	2.40	1.80	3.59	4.45	2.93	2.67	2.43	2.90

© 2020 Deng Y et al. *JAMA Network Open.*

Sex	Location	Incident cases						Age-standardized incidence rate (per 100,000)						EAPC		
		1990(95%UI)			2017(95%UI)			1990(95%UI)			2017(95%UI)			1990-2017(95%CI)		
		Cases (No.)	UL	LL	Cases (No.)	UL	LL	95%UI	UL	LL	95%UI	UL	LL	EAPC	LL	UL
Male	France	788.16	909.64	682.65	1645.39	2017.02	1340.05	2.39	2.76	2.09	3.60	4.45	2.91	2.16	0.63	3.71
Male	Gabon	1.31	1.70	1.04	3.62	4.73	2.71	0.46	0.58	0.38	0.63	0.80	0.48	1.16	1.05	1.28
Male	Gambia	0.68	0.90	0.50	2.14	2.85	1.55	0.31	0.40	0.23	0.41	0.54	0.31	1.30	1.21	1.40
Male	Georgia	17.75	21.80	13.96	36.79	43.66	30.31	0.66	0.80	0.53	1.60	1.89	1.33	3.29	2.17	4.42
Male	Germany	1160.23	1319.60	1021.51	2466.05	2985.45	1995.67	2.34	2.66	2.08	3.73	4.54	3.01	1.58	1.16	2.01
Male	Ghana	14.06	18.36	10.64	38.74	48.19	30.42	0.43	0.55	0.32	0.55	0.68	0.43	0.84	0.68	1.01
Male	Greece	96.90	112.94	81.99	206.78	252.92	170.23	1.47	1.71	1.24	2.54	3.10	2.08	1.97	1.58	2.38
Male	Greenland	0.16	0.20	0.12	0.33	0.40	0.27	0.69	0.84	0.54	0.87	1.03	0.71	0.99	0.74	1.24
Male	Grenada	0.65	0.74	0.56	1.48	1.77	1.24	2.16	2.47	1.87	2.23	2.67	1.86	1.13	0.58	1.67
Male	Grenadines	0.27	0.31	0.23	1.03	1.23	0.85	0.76	0.87	0.66	1.51	1.79	1.25	2.89	2.75	3.03
Male	Guam	0.45	0.57	0.35	2.28	2.84	1.80	0.90	1.12	0.73	2.54	3.15	2.01	4.32	2.86	5.80
Male	Guatemala	10.59	11.80	9.47	45.53	55.02	37.55	0.50	0.55	0.45	0.80	0.96	0.66	0.22	-0.41	0.86
Male	Guinea	8.49	10.08	7.09	20.01	27.30	14.85	0.45	0.54	0.38	0.61	0.84	0.46	1.32	1.25	1.40
Male	Guinea-Bissau	0.98	1.43	0.61	1.73	2.60	1.07	0.43	0.62	0.28	0.45	0.66	0.29	0.21	0.14	0.28
Male	Guyana	1.75	2.02	1.52	4.53	5.59	3.62	0.76	0.87	0.66	1.35	1.65	1.09	2.75	2.49	3.02
Male	Haiti	11.41	18.36	8.46	29.42	42.71	20.31	0.64	1.01	0.49	0.83	1.16	0.58	1.09	0.99	1.18
Male	Honduras	13.26	16.42	10.61	61.33	86.05	37.88	1.14	1.40	0.93	1.98	2.76	1.23	1.99	1.84	2.14
Male	Hungary	134.29	155.31	116.38	133.84	162.02	111.03	2.16	2.49	1.88	1.87	2.26	1.57	-0.68	-0.89	-0.47
Male	Iceland	9.10	10.56	7.77	27.36	33.08	22.61	6.97	8.11	5.93	12.31	14.80	10.23	2.49	2.34	2.64
Male	India	1688.82	2005.77	1469.66	6523.09	6947.88	6058.76	0.54	0.64	0.48	1.06	1.13	0.98	2.74	2.57	2.92
Male	Indonesia	401.70	466.45	352.92	1162.44	1367.25	1024.80	0.70	0.81	0.61	1.04	1.21	0.92	1.42	1.38	1.46
Male	Iran	89.32	113.00	75.15	601.25	704.39	509.85	0.52	0.65	0.44	1.44	1.68	1.24	4.99	4.52	5.45
Male	Iraq	47.93	65.39	35.03	121.77	148.42	99.52	0.98	1.34	0.72	0.82	0.99	0.69	-0.66	-0.86	-0.46

© 2020 Deng Y et al. *JAMA Network Open.*

Sex	Location	Incident cases						Age-standardized incidence rate (per 100,000)						EAPC		
		1990(95%UI)			2017(95%UI)			1990(95%UI)			2017(95%UI)			1990-2017(95%CI)		
		Cases (No.)	UL	LL	Cases (No.)	UL	LL	95%UI	UL	LL	95%UI	UL	LL	EAPC	LL	UL
Male	Ireland	30.29	35.29	25.75	74.96	96.60	59.45	1.64	1.90	1.40	2.36	3.02	1.88	2.16	1.82	2.50
Male	Israel	35.72	42.37	30.84	147.37	181.91	119.57	1.61	1.91	1.38	3.09	3.82	2.50	2.25	1.77	2.74
Male	Italy	1463.73	1728.60	1242.80	2360.59	2904.71	1910.58	4.02	4.74	3.42	4.73	5.93	3.83	0.77	0.55	0.99
Male	Ivory Coast	6.12	7.53	4.92	19.46	25.83	13.50	0.25	0.30	0.20	0.32	0.42	0.22	0.91	0.75	1.08
Male	Jamaica	4.64	5.35	4.00	23.07	30.82	16.46	0.54	0.62	0.46	1.61	2.14	1.15	4.29	3.84	4.75
Male	Japan	1268.47	1371.36	1185.37	3122.95	3581.98	2727.94	1.68	1.82	1.58	2.69	3.11	2.35	2.13	1.90	2.36
Male	Jordan	12.66	18.36	8.83	55.57	70.78	42.88	1.18	1.70	0.84	1.33	1.70	1.04	0.03	-0.17	0.24
Male	Kazakhstan	144.25	185.45	115.17	130.96	164.54	105.97	2.17	2.76	1.78	1.53	1.89	1.26	-2.25	-2.66	-1.83
Male	Kenya	17.87	21.65	13.68	77.51	95.65	62.15	0.32	0.38	0.25	0.53	0.65	0.43	2.18	1.99	2.36
Male	Kiribati	0.09	0.10	0.07	0.17	0.22	0.13	0.45	0.53	0.39	0.52	0.65	0.41	0.69	0.52	0.87
Male	Kuwait	8.51	10.30	7.01	34.93	44.31	27.60	1.18	1.36	1.01	1.67	2.08	1.33	2.74	2.04	3.45
Male	Kyrgyzstan	11.81	14.14	9.89	17.67	22.43	14.52	0.76	0.91	0.64	0.72	0.89	0.60	0.22	-0.18	0.61
Male	Laos	9.50	14.30	6.48	22.08	28.89	15.81	0.82	1.22	0.57	0.96	1.23	0.72	0.49	0.33	0.64
Male	Latvia	21.35	25.46	17.99	36.55	46.85	27.62	1.51	1.79	1.28	2.82	3.62	2.16	2.55	2.05	3.06
Male	Lebanon	17.95	23.24	14.23	175.01	227.80	125.16	1.33	1.70	1.05	4.51	5.84	3.32	5.29	5.02	5.57
Male	Lesotho	2.48	3.30	1.87	4.27	5.98	2.94	0.50	0.66	0.38	0.71	0.97	0.51	1.64	1.47	1.81
Male	Liberia	2.25	2.83	1.69	4.22	5.74	2.95	0.34	0.43	0.26	0.36	0.49	0.26	0.20	0.04	0.37
Male	Libya	9.06	12.06	6.77	50.78	68.47	36.26	0.63	0.82	0.47	1.46	1.93	1.08	3.96	3.57	4.35
Male	Lithuania	33.21	39.40	28.03	42.61	53.40	34.53	1.74	2.06	1.47	2.28	2.85	1.86	0.28	-0.27	0.84
Male	Luxembourg	11.43	13.66	9.48	25.17	32.75	19.19	4.84	5.74	4.04	5.89	7.59	4.55	0.44	0.06	0.81
Male	Macedonia	10.56	12.63	8.81	18.41	23.25	14.34	1.07	1.26	0.90	1.20	1.50	0.94	-0.53	-1.11	0.06
Male	Madagascar	19.54	24.37	15.11	52.28	69.59	38.76	0.52	0.65	0.41	0.70	0.91	0.53	1.33	1.20	1.47
Male	Malawi	5.38	7.45	2.55	16.93	21.94	12.75	0.24	0.31	0.14	0.37	0.47	0.29	1.66	1.48	1.85

© 2020 Deng Y et al. *JAMA Network Open.*

Sex	Location	Incident cases						Age-standardized incidence rate (per 100,000)						EAPC		
		1990(95%UI)			2017(95%UI)			1990(95%UI)			2017(95%UI)			1990-2017(95%CI)		
		Cases (No.)	UL	LL	Cases (No.)	UL	LL	95%UI	UL	LL	95%UI	UL	LL	EAPC	LL	UL
Male	Malaysia	71.47	87.79	58.58	360.02	467.90	273.24	1.23	1.49	1.02	2.42	3.13	1.86	2.32	2.14	2.49
Male	Maldives	1.70	2.84	1.08	8.65	10.86	6.80	3.00	4.84	2.00	4.78	5.96	3.76	1.55	1.39	1.71
Male	Mali	10.98	13.35	8.99	30.35	40.86	20.53	0.45	0.55	0.38	0.56	0.75	0.37	0.97	0.78	1.15
Male	Malta	3.32	3.86	2.86	10.79	12.93	9.01	1.68	1.95	1.45	3.27	3.92	2.75	2.71	2.43	2.99
Male	Marshall Islands	0.11	0.17	0.08	0.34	0.50	0.21	1.07	1.55	0.74	1.63	2.34	1.09	1.54	1.42	1.67
Male	Mauritania	2.18	2.69	1.76	5.17	7.63	3.03	0.39	0.48	0.32	0.47	0.69	0.27	0.67	0.51	0.82
Male	Mauritius	3.41	3.97	2.90	11.09	13.39	9.03	0.81	0.94	0.70	1.37	1.65	1.13	2.29	1.92	2.66
Male	Mexico	207.11	218.22	198.28	957.61	1026.12	880.74	0.83	0.87	0.80	1.66	1.78	1.53	2.47	2.35	2.58
Male	Micronesia	0.23	0.31	0.17	0.48	0.68	0.30	0.80	1.07	0.61	1.22	1.68	0.83	1.59	1.42	1.77
Male	Moldova	26.63	32.64	21.90	44.72	54.81	36.74	1.27	1.54	1.06	1.88	2.27	1.56	1.88	1.15	2.61
Male	Mongolia	3.66	4.39	3.01	14.26	18.30	10.82	0.64	0.76	0.53	1.08	1.36	0.84	2.29	1.99	2.59
Male	Montenegro	5.04	6.19	4.14	9.06	11.54	6.92	1.63	1.98	1.35	2.08	2.63	1.60	0.92	0.84	1.01
Male	Morocco	75.34	92.52	60.68	286.58	387.60	203.85	0.83	1.00	0.68	1.64	2.21	1.17	2.53	2.47	2.60
Male	Mozambique	23.33	31.51	16.90	87.50	125.01	56.89	0.62	0.81	0.46	1.15	1.60	0.77	2.81	2.59	3.04
Male	Myanmar	112.67	176.10	70.37	252.82	320.53	199.13	0.89	1.36	0.58	1.17	1.47	0.94	0.99	0.85	1.13
Male	Namibia	1.95	2.42	1.53	6.21	8.20	4.73	0.47	0.57	0.38	0.78	0.99	0.62	1.96	1.81	2.12
Male	Nepal	32.83	45.88	22.94	94.32	129.51	67.22	0.54	0.74	0.38	0.84	1.14	0.60	1.78	1.50	2.06
Male	Netherlands	164.01	190.67	139.72	410.30	490.59	332.04	1.90	2.20	1.62	3.20	3.83	2.61	2.14	1.86	2.43
Male	New Zealand	41.63	50.17	34.59	102.81	122.16	86.74	2.31	2.77	1.92	3.81	4.53	3.19	1.94	1.78	2.10
Male	Nicaragua	4.23	4.97	3.60	25.10	32.22	19.52	0.44	0.51	0.37	1.02	1.31	0.80	3.85	3.48	4.23
Male	Niger	5.44	7.16	3.70	12.73	17.13	9.08	0.31	0.40	0.21	0.31	0.41	0.22	-0.16	-0.29	-0.02
Male	Nigeria	61.66	87.91	41.53	143.25	203.07	102.10	0.24	0.35	0.17	0.31	0.44	0.22	0.81	0.64	0.98
Male	North Korea	75.23	104.77	52.96	170.78	243.82	123.05	0.95	1.27	0.69	1.20	1.64	0.90	0.84	0.74	0.94

© 2020 Deng Y et al. *JAMA Network Open.*

Sex	Location	Incident cases						Age-standardized incidence rate (per 100,000)						EAPC		
		1990(95%UI)			2017(95%UI)			1990(95%UI)			2017(95%UI)			1990-2017(95%CI)		
		Cases (No.)	UL	LL	Cases (No.)	UL	LL	95%UI	UL	LL	95%UI	UL	LL	EAPC	LL	UL
Male	Northern Mariana Islands	0.27	0.36	0.20	0.62	0.80	0.47	1.44	1.86	1.12	2.20	2.77	1.74	1.09	0.32	1.87
Male	Norway	58.45	66.46	51.52	127.34	149.22	108.48	2.26	2.57	1.98	3.46	4.05	2.95	2.01	1.78	2.24
Male	Oman	5.08	7.27	3.60	46.57	64.74	32.14	0.75	1.03	0.55	1.82	2.45	1.31	3.63	3.36	3.89
Male	Pakistan	355.40	442.24	282.59	1155.53	1577.41	808.54	0.92	1.14	0.75	1.46	1.97	1.03	1.85	1.77	1.94
Male	Palestine	2.80	4.01	2.03	11.09	13.52	8.98	0.58	0.85	0.42	0.73	0.88	0.60	0.81	0.52	1.10
Male	Panama	7.05	8.06	6.16	31.04	37.11	26.00	0.76	0.86	0.67	1.58	1.88	1.32	3.19	2.88	3.50
Male	Papua New Guinea	8.85	16.70	6.10	29.66	55.55	19.97	0.70	1.28	0.51	0.94	1.68	0.67	1.11	0.94	1.28
Male	Paraguay	9.02	10.91	7.34	44.67	58.67	33.08	0.76	0.90	0.62	1.58	2.07	1.17	2.79	2.29	3.29
Male	Peru	53.72	63.49	44.83	268.87	351.54	199.21	0.76	0.89	0.64	1.73	2.25	1.31	3.45	3.19	3.71
Male	Philippines	257.68	303.31	219.26	954.11	1202.97	754.85	1.38	1.61	1.19	2.42	3.02	1.93	2.23	2.05	2.42
Male	Poland	380.71	429.94	334.93	486.85	583.24	406.94	1.90	2.13	1.69	1.76	2.11	1.48	-0.75	-1.37	-0.12
Male	Portugal	92.11	106.52	79.27	213.37	262.37	169.90	1.60	1.84	1.38	2.60	3.22	2.06	2.07	1.80	2.33
Male	Puerto Rico	29.19	33.72	25.01	60.64	73.49	50.22	1.72	2.00	1.47	2.51	3.07	2.08	1.81	1.49	2.13
Male	Qatar	1.32	1.85	0.97	21.13	28.29	15.04	0.92	1.20	0.71	1.41	1.83	1.04	1.80	1.22	2.38
Male	Republic of Congo	2.60	3.60	1.94	7.07	9.89	4.91	0.46	0.61	0.36	0.48	0.67	0.34	0.18	0.01	0.36
Male	Romania	151.30	173.12	129.69	253.08	302.04	211.12	1.15	1.31	1.00	1.78	2.12	1.49	1.05	0.65	1.45
Male	Russia	1216.16	1439.74	1033.66	2204.11	2503.48	1959.30	1.63	1.91	1.40	2.54	2.88	2.26	1.54	1.22	1.86
Male	Rwanda	11.28	16.19	7.56	28.89	41.01	20.81	0.63	0.89	0.43	0.85	1.17	0.62	1.14	1.01	1.27
Male	Saint Lucia	0.31	0.35	0.26	1.20	1.43	1.00	0.70	0.81	0.61	1.16	1.37	0.97	1.95	1.82	2.08
Male	Saint Vincent	0.27	0.31	0.23	1.03	1.23	0.85	0.76	0.87	0.66	1.51	1.79	1.25	2.89	2.75	3.03

© 2020 Deng Y et al. *JAMA Network Open.*

Sex	Location	Incident cases						Age-standardized incidence rate (per 100,000)						EAPC		
		1990(95%UI)			2017(95%UI)			1990(95%UI)			2017(95%UI)			1990-2017(95%CI)		
		Cases (No.)	UL	LL	Cases (No.)	UL	LL	95%UI	UL	LL	95%UI	UL	LL	EAPC	LL	UL
Male	Samoa	0.61	0.79	0.46	0.97	1.42	0.62	1.15	1.47	0.88	1.27	1.77	0.85	0.23	0.09	0.37
Male	Sao Tome and Principe	0.09	0.12	0.07	0.28	0.39	0.19	0.28	0.36	0.21	0.46	0.62	0.32	2.00	1.91	2.09
Male	Saudi Arabia	53.07	70.98	39.86	762.92	1061.69	516.61	0.95	1.25	0.72	3.93	5.14	2.86	6.35	5.76	6.95
Male	Senegal	6.91	8.33	5.64	15.97	21.08	12.21	0.38	0.45	0.31	0.42	0.56	0.33	0.43	0.27	0.58
Male	Serbia	61.76	77.06	48.57	111.64	138.48	87.51	1.10	1.36	0.88	1.73	2.17	1.35	2.48	2.20	2.76
Male	Seychelles	0.25	0.30	0.20	0.56	0.69	0.46	0.93	1.13	0.77	0.98	1.18	0.80	-0.27	-0.48	-0.06
Male	Sierra Leone	3.25	4.24	2.20	6.37	8.29	4.85	0.29	0.38	0.20	0.32	0.42	0.25	0.33	0.29	0.37
Male	Singapore	17.90	20.90	15.37	60.12	73.96	48.59	1.26	1.45	1.11	1.76	2.15	1.44	1.86	1.67	2.06
Male	Slovakia	41.15	49.01	34.28	60.71	76.18	48.01	1.53	1.82	1.28	1.59	1.96	1.27	0.14	0.04	0.24
Male	Slovenia	17.55	20.47	15.12	37.34	45.91	30.17	1.64	1.89	1.42	2.28	2.78	1.86	1.41	1.20	1.61
Male	Solomon Islands	0.67	1.07	0.47	1.81	2.67	1.28	0.70	1.11	0.50	0.88	1.28	0.64	0.73	0.53	0.92
Male	Somalia	9.60	17.24	3.83	36.39	52.47	24.30	0.55	0.91	0.28	0.84	1.17	0.59	1.54	1.42	1.65
Male	South Africa	54.39	62.44	47.55	127.20	145.42	109.46	0.47	0.55	0.40	0.58	0.66	0.50	0.37	-0.02	0.77
Male	South Korea	142.75	164.14	121.86	2395.24	3071.88	1889.66	0.87	0.98	0.77	6.27	7.98	4.95	10.26	8.87	11.67
Male	South Sudan	9.94	17.84	4.55	24.36	37.61	16.33	0.58	0.99	0.30	0.91	1.39	0.62	1.71	1.56	1.87
Male	Spain	413.94	478.62	356.52	1063.81	1305.84	856.16	1.84	2.12	1.57	3.00	3.70	2.42	2.07	1.86	2.28
Male	Sri Lanka	79.25	95.18	66.04	223.65	301.93	156.96	1.27	1.50	1.06	1.90	2.55	1.35	1.02	0.45	1.59
Male	Sudan	27.09	44.39	17.08	84.85	113.97	59.81	0.48	0.77	0.31	0.72	0.96	0.51	1.68	1.53	1.83
Male	Suriname	0.61	0.71	0.52	2.61	3.27	2.08	0.43	0.50	0.37	0.88	1.10	0.71	2.90	2.73	3.06
Male	Swaziland	0.99	1.26	0.78	2.96	4.25	1.96	0.60	0.74	0.48	0.95	1.34	0.64	1.97	1.74	2.20
Male	Sweden	114.43	132.31	99.49	209.03	247.32	175.95	2.03	2.34	1.76	2.88	3.38	2.41	1.82	1.58	2.06
Male	Switzerland	106.14	125.66	90.31	202.16	253.50	161.98	2.51	2.96	2.13	3.07	3.83	2.47	1.36	1.08	1.63

© 2020 Deng Y et al. *JAMA Network Open.*

Sex	Location	Incident cases						Age-standardized incidence rate (per 100,000)						EAPC		
		1990(95%UI)			2017(95%UI)			1990(95%UI)			2017(95%UI)			1990-2017(95%CI)		
		Cases (No.)	UL	LL	Cases (No.)	UL	LL	95%UI	UL	LL	95%UI	UL	LL	EAPC	LL	UL
Male	Syria	11.70	15.93	8.97	53.95	71.35	40.42	0.35	0.48	0.27	0.69	0.90	0.52	2.30	2.11	2.49
Male	Tajikistan	3.94	4.66	3.33	11.39	14.15	9.28	0.28	0.32	0.23	0.38	0.46	0.31	0.87	0.46	1.28
Male	Tanzania	35.61	50.57	20.54	128.36	177.96	93.32	0.53	0.72	0.34	0.84	1.15	0.61	1.74	1.60	1.87
Male	Thailand	220.19	258.12	184.37	673.45	838.49	537.91	1.09	1.27	0.93	1.49	1.82	1.19	0.43	0.07	0.79
Male	Timor-Leste	1.22	1.61	0.82	4.53	6.70	2.76	0.64	0.84	0.42	1.07	1.56	0.66	2.14	1.88	2.40
Male	Tobago	3.97	4.57	3.47	11.24	15.13	8.27	0.87	0.99	0.77	1.31	1.76	0.97	1.53	1.11	1.95
Male	Togo	2.40	3.06	1.81	6.73	8.73	4.98	0.33	0.41	0.25	0.38	0.48	0.28	0.46	0.40	0.53
Male	Tonga	0.22	0.28	0.18	0.58	0.79	0.41	0.75	0.94	0.60	1.41	1.92	0.99	2.54	2.35	2.73
Male	Trinidad	3.97	4.57	3.47	11.24	15.13	8.27	0.87	0.99	0.77	1.31	1.76	0.97	1.53	1.11	1.95
Male	Tunisia	23.20	28.57	18.42	97.03	136.84	67.99	0.74	0.89	0.59	1.57	2.19	1.10	2.62	2.39	2.85
Male	Turkey	161.14	240.84	112.43	777.28	954.16	627.37	0.80	1.18	0.57	1.79	2.20	1.45	3.67	3.31	4.02
Male	Turkmenistan	8.44	9.90	7.05	23.57	29.22	19.24	0.76	0.87	0.64	1.03	1.26	0.85	1.12	0.68	1.57
Male	Uganda	17.49	23.41	12.58	114.87	152.27	86.41	0.42	0.54	0.31	1.25	1.63	0.96	4.63	4.28	4.97
Male	UK	530.64	570.31	494.47	1049.63	1109.67	992.65	1.50	1.61	1.40	2.26	2.39	2.14	1.87	1.66	2.07
Male	Ukraine	380.74	518.42	297.51	786.70	954.45	655.01	1.34	1.81	1.06	2.88	3.50	2.38	2.57	2.14	3.00
Male	United Arab Emirates	7.04	9.96	4.91	164.08	259.38	95.88	0.83	1.13	0.57	1.98	3.20	1.18	3.52	3.26	3.78
Male	Uruguay	12.25	14.32	10.62	38.37	47.04	30.56	0.74	0.86	0.64	1.93	2.36	1.53	3.36	2.82	3.90
Male	USA	4035.74	4219.26	3865.62	10403.87	11038.52	9810.87	3.01	3.15	2.88	4.57	4.84	4.30	1.20	0.94	1.46
Male	Uzbekistan	17.45	22.99	13.99	69.12	86.01	55.91	0.30	0.41	0.24	0.60	0.72	0.49	2.70	2.55	2.85
Male	Vanuatu	0.41	0.68	0.23	1.32	2.17	0.73	0.92	1.47	0.51	1.32	2.15	0.74	1.48	1.40	1.56
Male	Venezuela	35.92	41.51	31.52	223.75	290.68	167.06	0.64	0.73	0.56	1.52	1.96	1.14	3.00	2.71	3.29
Male	Vietnam	267.79	331.14	214.84	1693.17	2199.80	1268.64	1.27	1.56	1.03	3.54	4.54	2.75	4.58	4.23	4.93

© 2020 Deng Y et al. *JAMA Network Open.*

Sex	Location	Incident cases						Age-standardized incidence rate (per 100,000)						EAPC		
		1990(95%UI)			2017(95%UI)			1990(95%UI)			2017(95%UI)			1990-2017(95%CI)		
		Cases (No.)	UL	LL	Cases (No.)	UL	LL	95%UI	UL	LL	95%UI	UL	LL	EAPC	LL	UL
Male	Virgin Islands	0.49	0.60	0.40	1.91	2.46	1.43	1.06	1.26	0.89	2.57	3.26	1.95	4.13	3.76	4.50
Male	Yemen	14.09	27.91	7.16	57.86	84.36	41.35	0.45	0.84	0.25	0.70	1.00	0.51	1.85	1.77	1.92
Male	Zambia	13.35	18.16	8.78	40.03	53.06	30.70	0.66	0.89	0.47	0.86	1.12	0.67	0.81	0.74	0.89
Male	Zimbabwe	20.10	24.54	16.02	46.85	60.11	36.27	0.79	0.96	0.63	1.12	1.43	0.90	1.07	0.42	1.72

Abbreviations: CI, confidence interval; EAPC, estimated annual percentage change; UI, uncertainty interval; LL, lower limit; UL, upper limit.

© 2020 Deng Y et al. *JAMA Network Open.*

eTable 4. The deaths from thyroid cancer and its temporal trends from 1990 to 2017 among 195 countries.

Sex	Location	Deaths						Age-standardized death rate (per 100,000)						EAPC		
		1990(95%UI)			2017(95%UI)			1990(95%UI)			2017(95%UI)			1990-2017(95%CI)		
		Cases (No.)	UL	LL	Cases (No.)	UL	LL	95%UI	UL	LL	95%UI	UL	LL	EAPC	LL	UL
Both	Afghanistan	55.24	85.92	27.33	92.59	129.96	57.34	0.79	1.21	0.41	0.79	1.07	0.53	0.12	0.02	0.22
Both	Albania	11.36	12.55	9.99	16.73	20.52	13.61	0.51	0.56	0.45	0.41	0.51	0.34	-1.02	-1.29	-0.75
Both	Algeria	73.41	94.78	63.07	194.89	235.07	169.32	0.53	0.75	0.45	0.58	0.72	0.50	0.70	0.52	0.88
Both	American Samoa	0.19	0.23	0.15	0.33	0.37	0.28	1.01	1.24	0.76	0.92	1.05	0.79	-0.25	-0.70	0.21
Both	Andorra	0.24	0.31	0.19	0.48	0.63	0.38	0.42	0.55	0.33	0.35	0.46	0.27	-0.81	-0.96	-0.67
Both	Angola	18.84	25.72	13.17	41.92	57.16	33.43	0.47	0.61	0.36	0.41	0.56	0.32	-0.60	-0.64	-0.55
Both	Antigua	0.26	0.29	0.24	0.58	0.64	0.52	0.50	0.55	0.45	0.58	0.64	0.52	0.53	0.31	0.76
Both	Argentina	230.48	241.56	219.18	257.81	287.96	229.72	0.70	0.74	0.67	0.48	0.54	0.43	-1.54	-1.80	-1.28
Both	Armenia	5.77	6.60	5.04	26.27	28.72	23.98	0.22	0.26	0.20	0.64	0.69	0.58	4.13	3.12	5.15
Both	Australia	72.19	76.17	68.45	165.01	184.95	145.39	0.36	0.38	0.34	0.41	0.46	0.36	0.88	0.69	1.08
Both	Austria	98.61	104.54	93.37	78.04	85.58	70.53	0.79	0.83	0.75	0.42	0.46	0.38	-2.76	-2.96	-2.57
Both	Azerbaijan	20.43	25.97	17.35	35.63	46.33	29.68	0.39	0.52	0.33	0.39	0.53	0.33	-0.62	-0.85	-0.39
Both	Bahamas	0.95	1.03	0.88	2.25	2.52	2.01	0.59	0.64	0.55	0.60	0.68	0.54	0.26	0.07	0.45
Both	Bahrain	0.95	1.09	0.82	3.09	3.62	2.34	0.60	0.68	0.48	0.45	0.55	0.30	-1.10	-1.52	-0.68
Both	Bangladesh	327.20	468.59	252.24	547.05	677.93	450.06	0.58	0.82	0.47	0.43	0.53	0.36	-0.94	-1.06	-0.83
Both	Barbados	1.79	1.96	1.64	2.75	3.05	2.45	0.59	0.64	0.54	0.57	0.63	0.51	-0.05	-0.18	0.07
Both	Barbuda	0.26	0.29	0.24	0.58	0.64	0.52	0.50	0.55	0.45	0.58	0.64	0.52	0.53	0.31	0.76
Both	Belarus	58.65	64.13	54.33	86.05	97.90	77.19	0.45	0.49	0.42	0.54	0.61	0.48	0.48	0.05	0.92
Both	Belgium	141.40	150.26	132.84	98.90	109.86	89.26	0.88	0.93	0.83	0.43	0.47	0.39	-2.50	-3.02	-1.97

© 2020 Deng Y et al. *JAMA Network Open.*

Sex	Location	Deaths						Age-standardized death rate (per 100,000)						EAPC		
		1990(95%UI)			2017(95%UI)			1990(95%UI)			2017(95%UI)			1990-2017(95%CI)		
		Cases (No.)	UL	LL	Cases (No.)	UL	LL	95%UI	UL	LL	95%UI	UL	LL	EAPC	LL	UL
Both	Belize	0.28	0.31	0.26	0.96	1.06	0.88	0.30	0.33	0.27	0.36	0.40	0.33	0.83	0.63	1.04
Both	Benin	7.21	8.58	5.56	14.01	17.63	10.92	0.34	0.40	0.26	0.31	0.38	0.24	-0.41	-0.47	-0.34
Both	Bermuda	0.55	0.60	0.50	0.76	0.85	0.68	0.87	0.95	0.80	0.60	0.67	0.54	-1.32	-1.48	-1.17
Both	Bhutan	1.85	2.74	1.39	3.50	4.70	2.65	0.64	0.95	0.49	0.55	0.72	0.42	-0.62	-0.66	-0.58
Both	Bolivia	42.95	55.46	29.22	110.80	139.90	84.00	1.34	1.72	0.93	1.33	1.68	1.01	-0.05	-0.12	0.02
Both	Bosnia and Herzegovina	19.82	22.11	16.77	27.46	31.31	22.19	0.49	0.54	0.42	0.46	0.52	0.37	-0.45	-0.59	-0.31
Both	Botswana	2.33	3.02	1.80	5.21	6.66	4.31	0.41	0.53	0.32	0.41	0.53	0.34	0.59	0.17	1.02
Both	Brazil	521.18	537.57	506.01	1090.48	1121.79	1046.98	0.60	0.62	0.58	0.49	0.51	0.47	-0.74	-0.80	-0.67
Both	Brunei	0.93	1.13	0.78	2.49	2.83	2.19	0.99	1.20	0.85	0.91	1.03	0.80	0.14	-0.13	0.40
Both	Bulgaria	61.07	65.43	56.84	53.87	59.53	48.77	0.49	0.52	0.45	0.38	0.42	0.35	-1.15	-1.34	-0.95
Both	Burkina Faso	17.77	23.52	12.50	25.86	30.64	21.33	0.39	0.52	0.28	0.30	0.36	0.25	-1.23	-1.42	-1.05
Both	Burundi	20.33	26.15	14.25	29.03	35.95	23.05	0.80	1.03	0.59	0.64	0.79	0.50	-1.05	-1.15	-0.94
Both	Cambodia	51.53	67.53	33.64	93.32	116.19	76.47	1.11	1.43	0.75	0.87	1.09	0.71	-1.08	-1.15	-1.00
Both	Cameroon	19.74	23.63	15.09	39.41	49.63	30.97	0.43	0.51	0.33	0.36	0.45	0.28	-0.74	-0.87	-0.61
Both	Canada	127.52	133.58	121.56	244.61	268.61	222.21	0.39	0.40	0.37	0.36	0.40	0.33	-0.21	-0.41	-0.01
Both	Cape Verde	0.39	0.52	0.27	0.83	1.01	0.62	0.17	0.22	0.12	0.19	0.23	0.14	0.22	0.13	0.31
Both	Central African Republic	6.19	8.22	4.46	8.99	12.78	6.28	0.53	0.69	0.42	0.44	0.62	0.33	-0.76	-0.80	-0.71
Both	Chad	9.28	11.54	6.50	15.34	18.37	12.43	0.31	0.39	0.22	0.29	0.34	0.23	-0.35	-0.45	-0.24
Both	Chile	72.53	78.27	67.24	145.73	165.17	128.13	0.73	0.79	0.68	0.63	0.71	0.55	-0.58	-0.68	-0.48
Both	China	3108.79	3635.50	2889.65	6800.91	7432.81	6381.41	0.38	0.45	0.35	0.37	0.41	0.35	0.25	-0.04	0.54
Both	Colombia	166.13	174.94	157.20	356.53	405.21	311.61	0.95	1.00	0.90	0.66	0.75	0.58	-1.47	-1.75	-1.18

© 2020 Deng Y et al. *JAMA Network Open.*

Sex	Location	Deaths						Age-standardized death rate (per 100,000)						EAPC		
		1990(95%UI)			2017(95%UI)			1990(95%UI)			2017(95%UI)			1990-2017(95%CI)		
		Cases (No.)	UL	LL	Cases (No.)	UL	LL	95%UI	UL	LL	95%UI	UL	LL	EAPC	LL	UL
Both	Comoros	1.46	1.94	1.09	2.82	3.55	2.26	0.62	0.81	0.47	0.60	0.75	0.48	-0.09	-0.20	0.03
Both	Costa Rica	12.39	13.27	11.62	28.79	31.91	25.87	0.68	0.74	0.64	0.59	0.66	0.53	-0.99	-1.28	-0.69
Both	Croatia	49.20	53.12	45.51	37.24	40.97	33.82	0.77	0.83	0.71	0.42	0.46	0.38	-1.67	-1.96	-1.38
Both	Cuba	44.82	47.98	41.76	97.62	109.75	85.60	0.43	0.46	0.40	0.52	0.59	0.46	0.83	0.66	1.00
Both	Cyprus	5.65	6.57	4.43	7.73	8.87	6.69	0.66	0.76	0.52	0.41	0.47	0.35	-2.04	-2.37	-1.72
Both	Czech Republic	116.69	122.80	110.24	81.57	89.94	74.02	0.83	0.88	0.79	0.39	0.43	0.36	-2.74	-2.86	-2.62
Both	Democratic Republic of the Congo	64.12	82.02	50.49	118.01	161.84	84.79	0.41	0.52	0.33	0.37	0.51	0.26	-0.50	-0.60	-0.40
Both	Denmark	26.45	28.44	24.47	38.86	43.23	34.56	0.32	0.35	0.30	0.34	0.38	0.31	0.05	-0.22	0.32
Both	Djibouti	1.09	1.52	0.74	3.81	5.48	2.58	0.58	0.80	0.41	0.63	0.90	0.44	0.27	0.23	0.32
Both	Dominica	0.34	0.37	0.31	0.53	0.58	0.48	0.47	0.51	0.43	0.57	0.63	0.52	0.81	0.55	1.08
Both	Dominican Republic	17.44	19.65	14.44	40.57	48.46	33.07	0.45	0.52	0.37	0.44	0.53	0.36	-0.02	-0.55	0.51
Both	Ecuador	31.74	33.62	29.60	183.28	206.13	161.12	0.59	0.62	0.55	1.27	1.43	1.12	3.47	2.95	4.00
Both	Egypt	90.24	144.73	75.66	193.93	310.24	148.05	0.31	0.53	0.26	0.33	0.57	0.25	0.40	0.29	0.51
Both	El Salvador	31.98	36.69	19.77	30.11	43.95	23.83	1.07	1.23	0.66	0.53	0.77	0.42	-2.56	-3.42	-1.69
Both	Equatorial Guinea	1.08	1.52	0.73	1.90	2.99	1.18	0.55	0.76	0.41	0.42	0.66	0.27	-1.09	-1.28	-0.89
Both	Eritrea	8.75	11.85	5.92	22.51	28.93	16.72	0.80	1.04	0.59	0.87	1.12	0.65	0.22	0.06	0.38
Both	Estonia	11.22	12.14	10.36	14.76	17.50	12.34	0.54	0.58	0.50	0.52	0.62	0.44	-0.46	-0.81	-0.11
Both	Ethiopia	521.93	771.16	314.68	660.06	796.70	526.76	2.24	3.21	1.46	1.45	1.73	1.16	-1.87	-1.97	-1.77
Both	Fiji	5.11	6.36	3.23	8.43	10.35	5.34	1.48	1.85	0.92	1.30	1.61	0.81	-0.68	-0.85	-0.51
Both	Finland	44.63	47.49	41.63	58.11	64.18	52.48	0.61	0.64	0.57	0.47	0.52	0.43	-0.84	-0.98	-0.69
Both	France	572.28	594.38	548.62	534.22	585.96	490.44	0.66	0.68	0.63	0.38	0.42	0.35	-1.88	-2.42	-1.33
Both	Gabon	2.80	3.73	2.16	4.01	5.65	3.16	0.50	0.67	0.39	0.40	0.57	0.31	-0.84	-0.94	-0.73

© 2020 Deng Y et al. *JAMA Network Open.*

Sex	Location	Deaths						Age-standardized death rate (per 100,000)						EAPC		
		1990(95%UI)			2017(95%UI)			1990(95%UI)			2017(95%UI)			1990-2017(95%CI)		
		Cases (No.)	UL	LL	Cases (No.)	UL	LL	95%UI	UL	LL	95%UI	UL	LL	EAPC	LL	UL
Both	Gambia	1.13	1.37	0.95	3.12	3.94	2.54	0.31	0.37	0.26	0.33	0.42	0.27	0.40	0.33	0.47
Both	Georgia	18.78	20.85	16.32	30.96	34.09	27.63	0.30	0.34	0.27	0.53	0.58	0.47	2.52	1.67	3.37
Both	Germany	1000.85	1039.96	958.96	953.49	1059.31	849.50	0.75	0.78	0.72	0.50	0.56	0.45	-1.93	-2.29	-1.56
Both	Ghana	11.58	14.52	9.13	27.42	33.06	22.34	0.19	0.24	0.15	0.20	0.24	0.16	0.10	0.03	0.18
Both	Greece	69.04	73.93	64.22	92.75	102.11	83.31	0.45	0.48	0.42	0.36	0.40	0.33	-1.04	-1.33	-0.76
Both	Greenland	0.16	0.19	0.12	0.35	0.42	0.18	0.56	0.65	0.37	0.59	0.73	0.29	0.75	0.12	1.38
Both	Grenada	0.83	0.89	0.76	1.24	1.37	1.12	1.16	1.25	1.07	0.80	0.88	0.72	-0.67	-1.12	-0.23
Both	Grenadines	0.56	0.62	0.52	1.08	1.19	0.97	0.76	0.83	0.69	0.79	0.87	0.71	0.27	-0.06	0.59
Both	Guam	0.28	0.35	0.25	1.06	1.19	0.91	0.41	0.51	0.36	0.62	0.70	0.53	1.87	1.20	2.53
Both	Guatemala	22.05	23.68	20.62	57.51	65.29	50.49	0.61	0.65	0.57	0.54	0.61	0.47	-2.06	-2.69	-1.42
Both	Guinea	14.23	16.43	12.50	24.16	30.14	20.24	0.40	0.47	0.35	0.43	0.54	0.35	0.25	0.20	0.30
Both	Guinea-Bissau	1.94	2.45	1.41	2.57	3.38	1.83	0.46	0.57	0.34	0.38	0.49	0.28	-0.74	-0.80	-0.69
Both	Guyana	1.97	2.11	1.83	3.52	4.00	3.09	0.50	0.53	0.46	0.57	0.65	0.51	0.81	0.61	1.01
Both	Haiti	27.36	40.33	17.35	50.25	69.73	36.51	0.84	1.22	0.56	0.77	1.05	0.57	-0.22	-0.31	-0.14
Both	Honduras	16.41	19.03	14.03	46.41	63.46	34.67	0.79	0.92	0.66	0.80	1.08	0.60	0.07	-0.08	0.22
Both	Hungary	129.91	137.65	121.74	84.04	91.83	76.51	0.87	0.92	0.82	0.43	0.48	0.40	-2.67	-2.96	-2.39
Both	Iceland	3.69	3.96	3.44	5.47	5.99	4.99	1.26	1.34	1.17	1.02	1.11	0.93	-0.71	-0.81	-0.62
Both	India	2548.89	3108.41	2108.93	6511.07	7021.42	5851.91	0.50	0.61	0.42	0.60	0.65	0.54	0.73	0.63	0.83
Both	Indonesia	738.30	844.39	588.23	1363.16	1568.05	1185.72	0.74	0.84	0.60	0.69	0.80	0.61	-0.25	-0.32	-0.17
Both	Iran	79.50	90.24	69.78	274.11	296.40	217.11	0.32	0.36	0.28	0.41	0.44	0.32	1.70	1.21	2.19
Both	Iraq	52.49	67.34	40.82	96.86	111.07	80.26	0.64	0.83	0.51	0.42	0.47	0.35	-1.70	-1.94	-1.45
Both	Ireland	23.05	24.82	21.26	24.49	27.62	21.59	0.55	0.59	0.51	0.34	0.38	0.30	-1.40	-1.73	-1.07
Both	Israel	32.30	34.96	29.88	75.19	84.54	66.85	0.65	0.71	0.61	0.65	0.73	0.58	-0.28	-0.59	0.03

© 2020 Deng Y et al. *JAMA Network Open.*

Sex	Location	Deaths						Age-standardized death rate (per 100,000)						EAPC		
		1990(95%UI)			2017(95%UI)			1990(95%UI)			2017(95%UI)			1990-2017(95%CI)		
		Cases (No.)	UL	LL	Cases (No.)	UL	LL	95%UI	UL	LL	95%UI	UL	LL	EAPC	LL	UL
Both	Italy	590.17	613.88	567.89	630.72	689.49	569.35	0.65	0.67	0.63	0.42	0.46	0.38	-1.53	-1.60	-1.45
Both	Ivory Coast	15.15	18.17	12.62	35.35	44.27	27.62	0.33	0.39	0.29	0.33	0.42	0.26	-0.03	-0.19	0.13
Both	Jamaica	6.93	7.66	6.34	19.11	22.72	15.97	0.37	0.41	0.34	0.66	0.78	0.55	2.17	1.87	2.46
Both	Japan	1015.48	1036.21	995.05	2039.50	2143.40	1962.20	0.60	0.62	0.59	0.48	0.51	0.46	-0.90	-0.99	-0.80
Both	Jordan	10.27	12.53	8.14	25.73	32.27	21.33	0.67	0.83	0.54	0.48	0.60	0.40	-1.59	-1.91	-1.27
Both	Kazakhstan	116.33	137.52	105.82	78.90	87.81	71.51	0.86	1.01	0.78	0.47	0.52	0.42	-3.15	-3.52	-2.78
Both	Kenya	21.73	25.33	18.42	56.54	68.48	47.11	0.25	0.28	0.20	0.26	0.31	0.21	0.13	0.02	0.24
Both	Kiribati	0.14	0.16	0.13	0.24	0.29	0.20	0.42	0.47	0.36	0.42	0.50	0.34	0.11	0.02	0.19
Both	Kuwait	3.75	4.02	3.48	8.65	9.85	7.65	0.60	0.65	0.55	0.39	0.44	0.34	-1.44	-1.80	-1.09
Both	Kyrgyzstan	16.92	18.67	15.21	15.36	17.29	13.96	0.55	0.61	0.49	0.36	0.40	0.32	-1.37	-1.54	-1.20
Both	Laos	23.57	32.61	14.81	31.36	39.35	24.46	1.11	1.50	0.73	0.78	0.97	0.62	-1.51	-1.60	-1.43
Both	Latvia	17.64	19.12	16.30	23.73	27.72	20.10	0.49	0.53	0.45	0.57	0.67	0.49	0.37	-0.03	0.78
Both	Lebanon	15.75	19.79	12.78	37.66	47.47	31.26	0.68	0.89	0.55	0.61	0.80	0.51	-0.37	-0.50	-0.23
Both	Lesotho	4.36	5.48	3.52	6.59	9.13	4.77	0.46	0.59	0.37	0.59	0.81	0.44	1.59	1.26	1.91
Both	Liberia	4.04	4.98	2.99	5.41	6.76	4.31	0.35	0.43	0.26	0.29	0.36	0.23	-0.88	-1.07	-0.70
Both	Libya	9.42	12.25	7.79	23.89	31.39	19.51	0.46	0.63	0.38	0.50	0.69	0.40	0.49	0.32	0.65
Both	Lithuania	22.39	24.20	20.77	27.37	30.43	24.61	0.49	0.52	0.45	0.47	0.52	0.42	-0.90	-1.39	-0.40
Both	Luxembourg	5.37	5.82	4.93	5.99	6.99	5.22	0.96	1.04	0.88	0.61	0.71	0.53	-1.92	-2.02	-1.82
Both	Macedonia	8.80	9.86	6.96	10.39	11.96	8.90	0.46	0.51	0.37	0.31	0.36	0.27	-2.30	-2.73	-1.88
Both	Madagascar	33.18	42.55	26.06	64.03	79.45	51.30	0.53	0.67	0.42	0.54	0.68	0.43	0.17	0.07	0.28
Both	Malawi	27.22	35.19	15.74	47.31	62.15	35.60	0.57	0.73	0.38	0.55	0.70	0.43	-0.51	-0.74	-0.27
Both	Malaysia	84.37	96.13	73.44	202.71	255.03	170.63	0.94	1.08	0.81	0.85	1.05	0.72	-0.36	-0.49	-0.22
Both	Maldives	1.38	2.01	0.92	2.67	3.13	2.29	1.69	2.38	1.20	1.01	1.18	0.86	-2.37	-2.58	-2.15

© 2020 Deng Y et al. *JAMA Network Open.*

Sex	Location	Deaths						Age-standardized death rate (per 100,000)						EAPC		
		1990(95%UI)			2017(95%UI)			1990(95%UI)			2017(95%UI)			1990-2017(95%CI)		
		Cases (No.)	UL	LL	Cases (No.)	UL	LL	95%UI	UL	LL	95%UI	UL	LL	EAPC	LL	UL
Both	Mali	26.07	30.75	22.74	42.65	56.10	33.16	0.59	0.74	0.51	0.47	0.62	0.37	-0.93	-1.04	-0.81
Both	Malta	3.14	3.39	2.89	4.86	5.40	4.37	0.73	0.79	0.68	0.55	0.60	0.50	-1.03	-1.18	-0.88
Both	Marshall Islands	0.14	0.18	0.10	0.30	0.39	0.21	0.90	1.11	0.67	1.00	1.26	0.73	0.39	0.20	0.57
Both	Mauritania	4.02	4.90	2.91	5.88	7.46	4.60	0.38	0.47	0.28	0.31	0.39	0.24	-0.89	-0.98	-0.80
Both	Mauritius	3.08	3.35	2.84	6.46	7.10	5.78	0.44	0.47	0.40	0.40	0.44	0.36	0.16	-0.28	0.60
Both	Mexico	302.65	311.36	295.52	845.29	872.90	811.96	0.70	0.72	0.69	0.76	0.78	0.73	0.21	0.10	0.31
Both	Micronesia	0.39	0.48	0.31	0.51	0.64	0.41	0.83	1.02	0.67	0.84	1.02	0.69	0.01	-0.06	0.08
Both	Moldova	20.72	22.77	18.90	21.90	23.93	19.95	0.45	0.49	0.41	0.39	0.42	0.35	-0.50	-0.86	-0.15
Both	Mongolia	5.08	5.85	3.59	10.48	12.82	6.61	0.48	0.55	0.33	0.50	0.62	0.29	0.17	-0.13	0.46
Both	Montenegro	3.74	4.33	3.18	5.48	6.26	4.73	0.60	0.69	0.50	0.55	0.63	0.47	-0.51	-0.67	-0.34
Both	Morocco	103.92	121.62	90.06	203.13	256.62	166.63	0.66	0.79	0.57	0.64	0.81	0.53	-0.09	-0.14	-0.04
Both	Mozambique	44.65	60.42	34.43	90.37	115.72	64.91	0.63	0.84	0.49	0.71	0.90	0.52	0.61	0.46	0.76
Both	Myanmar	298.11	409.39	191.89	397.41	508.18	318.60	1.26	1.71	0.84	0.92	1.17	0.75	-1.29	-1.43	-1.15
Both	Namibia	3.25	4.64	2.43	5.72	7.63	4.66	0.43	0.66	0.32	0.39	0.55	0.31	-0.52	-0.96	-0.07
Both	Nepal	60.69	88.64	46.17	126.62	166.48	102.27	0.58	0.85	0.45	0.58	0.77	0.47	0.05	-0.18	0.28
Both	Netherlands	93.59	98.72	88.33	143.03	156.02	130.39	0.45	0.48	0.43	0.42	0.46	0.38	-0.26	-0.45	-0.07
Both	New Zealand	16.85	18.03	15.73	27.36	29.78	24.85	0.42	0.45	0.39	0.37	0.40	0.34	-0.18	-0.41	0.06
Both	Nicaragua	6.43	8.67	5.65	23.16	26.91	19.89	0.40	0.55	0.35	0.52	0.60	0.44	1.36	0.97	1.75
Both	Niger	9.63	11.81	6.82	17.74	22.05	13.94	0.32	0.39	0.23	0.25	0.31	0.20	-1.13	-1.24	-1.01
Both	Nigeria	110.05	154.51	70.04	177.27	247.00	127.40	0.26	0.37	0.16	0.23	0.32	0.17	-0.64	-0.75	-0.53
Both	North Korea	67.60	87.03	54.52	129.61	167.41	104.00	0.42	0.55	0.34	0.42	0.55	0.34	0.06	-0.06	0.19
Both	Northern Mariana Islands	0.09	0.10	0.07	0.21	0.24	0.18	0.56	0.66	0.47	0.47	0.54	0.41	-0.73	-1.05	-0.41

© 2020 Deng Y et al. *JAMA Network Open.*

Sex	Location	Deaths						Age-standardized death rate (per 100,000)						EAPC		
		1990(95%UI)			2017(95%UI)			1990(95%UI)			2017(95%UI)			1990-2017(95%CI)		
		Cases (No.)	UL	LL	Cases (No.)	UL	LL	95%UI	UL	LL	95%UI	UL	LL	EAPC	LL	UL
Both	Norway	34.71	35.56	33.84	41.43	43.63	39.29	0.49	0.50	0.48	0.43	0.46	0.41	-0.36	-0.47	-0.25
Both	Oman	3.37	4.21	2.67	9.08	10.86	7.42	0.45	0.56	0.36	0.44	0.53	0.36	0.21	-0.10	0.52
Both	Pakistan	688.90	854.42	581.08	1743.68	2363.06	1301.09	1.05	1.26	0.89	1.34	1.77	1.03	0.85	0.63	1.08
Both	Palestine	4.73	5.79	3.41	10.50	12.12	8.61	0.54	0.66	0.38	0.48	0.56	0.37	-0.21	-0.38	-0.05
Both	Panama	7.57	8.11	7.06	20.18	21.97	18.24	0.49	0.52	0.45	0.51	0.56	0.46	0.60	0.41	0.79
Both	Papua New Guinea	12.98	17.49	10.12	31.46	41.62	24.25	0.71	0.94	0.56	0.75	0.96	0.58	0.28	0.19	0.37
Both	Paraguay	13.49	15.31	11.26	40.23	49.17	31.29	0.62	0.70	0.51	0.78	0.95	0.60	1.05	0.81	1.29
Both	Peru	87.21	100.78	76.55	233.87	279.62	184.51	0.71	0.83	0.63	0.77	0.91	0.60	0.27	0.04	0.50
Both	Philippines	337.15	373.38	307.01	1059.08	1250.75	897.61	1.12	1.24	1.02	1.55	1.82	1.33	1.38	1.19	1.58
Both	Poland	412.92	431.95	396.51	313.41	343.07	286.49	0.91	0.95	0.87	0.45	0.49	0.41	-3.26	-3.68	-2.85
Both	Portugal	108.83	116.09	101.61	106.07	116.71	95.47	0.80	0.85	0.75	0.43	0.47	0.38	-2.52	-2.89	-2.15
Both	Puerto Rico	22.54	24.21	20.99	23.26	25.80	21.12	0.60	0.65	0.56	0.34	0.37	0.31	-2.10	-2.50	-1.70
Both	Qatar	1.00	1.25	0.52	2.78	3.43	2.23	1.36	1.74	0.52	0.36	0.43	0.30	-5.95	-6.52	-5.37
Both	Republic of Congo	6.18	8.57	4.55	10.99	16.03	8.37	0.57	0.77	0.45	0.46	0.66	0.34	-0.90	-1.02	-0.78
Both	Romania	142.94	150.63	135.14	156.69	170.39	143.42	0.50	0.53	0.48	0.43	0.47	0.40	-0.97	-1.24	-0.70
Both	Russia	888.44	951.38	859.27	1116.80	1150.55	1082.68	0.50	0.53	0.48	0.48	0.50	0.47	-0.42	-0.87	0.04
Both	Rwanda	25.34	34.82	16.07	34.89	45.32	27.60	0.74	1.02	0.49	0.57	0.73	0.46	-1.29	-1.44	-1.14
Both	Saint Lucia	0.76	0.82	0.70	1.45	1.60	1.29	0.84	0.91	0.77	0.70	0.77	0.62	-0.79	-1.18	-0.40
Both	Saint Vincent	0.56	0.62	0.52	1.08	1.19	0.97	0.76	0.83	0.69	0.79	0.87	0.71	0.27	-0.06	0.59
Both	Samoa	0.68	0.97	0.54	1.01	1.51	0.78	0.77	1.25	0.60	0.76	1.24	0.58	-0.08	-0.14	-0.03
Both	Sao Tome and Principe	0.18	0.26	0.14	0.31	0.38	0.25	0.26	0.36	0.20	0.30	0.37	0.25	0.34	0.21	0.46
Both	Saudi Arabia	34.70	43.14	28.46	130.89	154.98	108.99	0.51	0.66	0.41	0.75	0.89	0.65	2.30	1.96	2.64

© 2020 Deng Y et al. *JAMA Network Open.*

Sex	Location	Deaths						Age-standardized death rate (per 100,000)						EAPC		
		1990(95%UI)			2017(95%UI)			1990(95%UI)			2017(95%UI)			1990-2017(95%CI)		
		Cases (No.)	UL	LL	Cases (No.)	UL	LL	95%UI	UL	LL	95%UI	UL	LL	EAPC	LL	UL
Both	Senegal	11.07	12.98	8.76	21.06	25.78	17.46	0.33	0.38	0.26	0.30	0.37	0.25	-0.43	-0.54	-0.33
Both	Serbia	54.32	64.72	45.27	76.76	86.78	66.17	0.48	0.57	0.41	0.49	0.55	0.42	0.34	0.21	0.46
Both	Seychelles	0.26	0.30	0.21	0.31	0.34	0.27	0.46	0.53	0.36	0.30	0.33	0.27	-1.58	-1.80	-1.35
Both	Sierra Leone	6.47	8.26	4.82	10.27	12.95	8.12	0.32	0.40	0.24	0.31	0.38	0.24	-0.12	-0.24	-0.01
Both	Singapore	11.74	12.55	10.97	19.34	21.59	17.45	0.54	0.58	0.50	0.29	0.32	0.26	-2.02	-2.17	-1.88
Both	Slovakia	39.27	42.97	34.79	38.39	44.74	33.38	0.65	0.71	0.58	0.43	0.50	0.37	-1.73	-1.84	-1.61
Both	Slovenia	15.14	16.31	14.03	16.48	18.37	14.69	0.61	0.66	0.57	0.38	0.42	0.34	-1.88	-1.98	-1.78
Both	Solomon Islands	0.90	1.15	0.71	2.13	2.68	1.67	0.65	0.82	0.51	0.70	0.86	0.55	0.38	0.27	0.49
Both	Somalia	17.84	27.89	9.12	50.35	66.86	36.65	0.62	0.91	0.38	0.71	0.93	0.53	0.40	0.32	0.49
Both	South Africa	74.26	88.36	63.22	135.65	153.99	123.10	0.34	0.42	0.29	0.32	0.36	0.29	-0.33	-0.88	0.21
Both	South Korea	113.69	118.44	104.67	712.70	786.02	644.72	0.42	0.44	0.38	0.87	0.96	0.79	3.70	2.48	4.93
Both	South Sudan	15.93	25.09	8.98	27.41	39.22	19.04	0.59	0.88	0.37	0.65	0.93	0.46	0.27	0.14	0.41
Both	Spain	263.12	275.62	251.74	374.69	408.83	342.33	0.47	0.49	0.45	0.38	0.41	0.35	-0.79	-0.85	-0.72
Both	Sri Lanka	83.06	92.50	67.38	128.80	160.18	101.55	0.78	0.86	0.64	0.53	0.66	0.42	-1.86	-2.32	-1.40
Both	Sudan	45.51	62.25	33.32	81.43	103.26	61.96	0.47	0.62	0.35	0.45	0.56	0.34	-0.07	-0.14	-0.01
Both	Suriname	1.37	1.51	1.24	3.35	3.78	2.93	0.53	0.59	0.48	0.59	0.66	0.51	0.55	0.37	0.74
Both	Swaziland	1.61	2.08	1.30	2.94	4.10	2.05	0.56	0.72	0.45	0.55	0.78	0.39	0.38	-0.04	0.80
Both	Sweden	75.32	79.69	70.86	82.43	89.71	75.78	0.47	0.49	0.44	0.38	0.41	0.35	-0.38	-0.56	-0.21
Both	Switzerland	54.05	58.26	50.30	73.90	82.82	66.20	0.50	0.53	0.46	0.41	0.46	0.37	-0.59	-0.79	-0.38
Both	Syria	11.07	13.79	8.24	24.10	28.65	19.72	0.21	0.26	0.15	0.19	0.23	0.16	-0.59	-0.76	-0.41
Both	Tajikistan	4.97	6.25	3.81	8.63	9.91	7.24	0.18	0.23	0.13	0.18	0.21	0.14	-0.36	-0.62	-0.11
Both	Tanzania	73.93	97.15	51.92	157.16	202.08	124.37	0.59	0.78	0.45	0.59	0.75	0.48	-0.17	-0.35	0.01
Both	Thailand	273.38	353.50	238.49	489.54	729.60	417.99	0.81	1.01	0.70	0.51	0.76	0.44	-2.00	-2.23	-1.76

© 2020 Deng Y et al. *JAMA Network Open.*

Sex	Location	Deaths						Age-standardized death rate (per 100,000)						EAPC		
		1990(95%UI)			2017(95%UI)			1990(95%UI)			2017(95%UI)			1990-2017(95%CI)		
		Cases (No.)	UL	LL	Cases (No.)	UL	LL	95%UI	UL	LL	95%UI	UL	LL	EAPC	LL	UL
Both	Timor-Leste	2.36	3.13	1.67	5.72	7.22	4.40	0.82	1.07	0.61	0.73	0.92	0.57	-0.46	-0.57	-0.34
Both	Tobago	4.89	5.25	4.53	8.40	10.32	6.92	0.56	0.61	0.52	0.47	0.58	0.39	-1.36	-1.85	-0.87
Both	Togo	4.49	5.66	3.37	9.00	10.89	7.31	0.33	0.41	0.26	0.28	0.33	0.22	-0.83	-0.90	-0.76
Both	Tonga	0.30	0.36	0.26	0.52	0.65	0.43	0.60	0.73	0.51	0.69	0.85	0.56	0.53	0.42	0.64
Both	Trinidad	4.89	5.25	4.53	8.40	10.32	6.92	0.56	0.61	0.52	0.47	0.58	0.39	-1.36	-1.85	-0.87
Both	Tunisia	22.59	27.98	19.72	51.12	69.04	39.57	0.45	0.58	0.39	0.44	0.59	0.34	-0.23	-0.34	-0.12
Both	Turkey	218.66	285.12	161.73	366.85	480.60	318.32	0.62	0.80	0.46	0.42	0.56	0.37	-1.53	-1.69	-1.37
Both	Turkmenistan	9.02	9.85	8.30	12.78	14.02	11.49	0.45	0.49	0.42	0.34	0.37	0.30	-1.08	-1.44	-0.72
Both	Uganda	42.68	51.87	35.08	116.96	143.78	94.27	0.56	0.68	0.46	0.74	0.89	0.60	1.20	1.06	1.35
Both	UK	371.38	385.12	357.71	426.21	438.38	413.99	0.40	0.41	0.38	0.33	0.34	0.32	-0.64	-0.74	-0.53
Both	Ukraine	322.98	375.64	286.09	374.34	410.95	343.35	0.44	0.51	0.39	0.50	0.55	0.46	0.16	-0.19	0.51
Both	United Arab Emirates	2.90	3.83	2.12	23.25	34.55	15.17	0.55	0.82	0.36	0.55	0.84	0.35	0.23	0.08	0.39
Both	Uruguay	15.36	16.87	14.11	25.85	29.17	22.62	0.38	0.42	0.35	0.48	0.54	0.42	0.38	-0.11	0.88
Both	USA	1167.86	1193.61	1145.65	2137.67	2203.80	2061.26	0.35	0.36	0.35	0.39	0.40	0.38	0.34	0.26	0.42
Both	Uzbekistan	16.29	23.91	14.04	40.75	46.14	35.59	0.14	0.21	0.12	0.21	0.24	0.18	1.42	1.19	1.66
Both	Vanuatu	0.50	0.73	0.33	1.46	2.16	0.91	0.76	1.07	0.51	0.92	1.34	0.58	0.82	0.71	0.93
Both	Venezuela	44.74	47.85	41.66	156.94	183.76	132.18	0.47	0.50	0.43	0.58	0.68	0.49	0.69	0.55	0.83
Both	Vietnam	425.80	605.08	352.97	1033.91	1320.31	861.59	1.00	1.50	0.82	1.15	1.49	0.96	0.96	0.73	1.20
Both	Virgin Islands	0.35	0.38	0.31	0.83	0.96	0.71	0.41	0.45	0.37	0.46	0.52	0.39	0.73	0.58	0.88
Both	Yemen	24.07	36.83	15.21	60.37	81.96	43.75	0.47	0.70	0.31	0.47	0.63	0.35	0.03	-0.01	0.07
Both	Zambia	26.38	34.65	19.18	47.48	59.56	38.41	0.75	0.96	0.58	0.64	0.81	0.52	-0.94	-1.19	-0.70
Both	Zimbabwe	27.37	31.16	23.35	61.08	73.77	48.45	0.64	0.73	0.55	0.85	1.03	0.70	1.60	0.94	2.27
Female	Afghanistan	84.52	167.44	28.67	261.94	461.77	114.00	2.46	4.85	0.88	3.00	5.09	1.44	0.97	0.81	1.12

© 2020 Deng Y et al. *JAMA Network Open.*

Sex	Location	Deaths						Age-standardized death rate (per 100,000)						EAPC		
		1990(95%UI)			2017(95%UI)			1990(95%UI)			2017(95%UI)			1990-2017(95%CI)		
		Cases (No.)	UL	LL	Cases (No.)	UL	LL	95%UI	UL	LL	95%UI	UL	LL	EAPC	LL	UL
Female	Albania	33.98	40.99	22.29	69.64	96.95	49.42	2.68	3.24	1.72	3.83	5.33	2.73	1.27	1.09	1.45
Female	Algeria	279.01	351.92	222.84	1439.11	1830.40	1069.44	3.11	4.00	2.54	6.95	8.70	5.39	3.43	3.22	3.65
Female	American Samoa	0.56	0.72	0.44	1.64	2.09	1.32	4.20	5.33	3.22	6.50	8.16	5.34	1.97	1.36	2.59
Female	Andorra	1.34	1.97	0.91	3.23	5.04	2.24	4.56	6.66	3.09	5.46	8.47	3.84	0.38	0.17	0.60
Female	Angola	29.08	45.86	16.32	91.58	148.60	64.14	1.08	1.70	0.67	1.14	1.86	0.81	0.07	-0.08	0.21
Female	Antigua	1.08	1.26	0.92	3.63	4.32	3.02	3.86	4.51	3.30	6.46	7.63	5.44	1.84	1.41	2.26
Female	Argentina	584.77	651.84	519.25	924.47	1125.04	757.14	3.41	3.82	3.02	3.58	4.37	2.92	-0.19	-0.52	0.14
Female	Armenia	15.40	18.13	12.85	78.58	90.71	66.78	0.93	1.09	0.78	3.59	4.12	3.08	5.78	4.70	6.87
Female	Australia	330.16	366.10	298.30	1134.71	1366.94	910.15	3.35	3.72	3.01	6.85	8.32	5.46	3.22	2.98	3.47
Female	Austria	405.81	454.48	361.51	399.91	463.03	338.25	7.31	8.23	6.42	5.67	6.62	4.78	-1.29	-1.51	-1.06
Female	Azerbaijan	65.83	82.87	53.84	227.49	296.17	174.92	2.03	2.57	1.67	3.88	5.03	2.99	2.31	1.96	2.66
Female	Bahamas	3.82	4.50	3.25	11.41	13.87	9.14	3.52	4.10	3.00	5.09	6.16	4.09	1.61	1.40	1.82
Female	Bahrain	3.06	4.17	2.50	20.05	25.38	15.94	2.80	3.46	2.33	4.15	5.09	3.15	1.63	1.11	2.15
Female	Bangladesh	598.16	1002.29	385.13	2169.24	3372.86	1541.98	1.67	2.83	1.14	2.80	4.25	2.04	2.42	2.17	2.68
Female	Barbados	5.23	5.94	4.60	12.12	14.39	10.10	3.72	4.26	3.24	5.73	6.85	4.71	1.58	1.41	1.76
Female	Barbuda	1.08	1.26	0.92	3.63	4.32	3.02	3.86	4.51	3.30	6.46	7.63	5.44	1.84	1.41	2.26
Female	Belarus	280.98	323.45	247.21	471.84	574.18	390.65	4.28	4.90	3.76	5.98	7.33	4.96	1.15	0.82	1.48
Female	Belgium	452.64	507.74	401.40	406.72	481.23	343.91	6.22	7.08	5.47	4.71	5.62	3.95	-0.95	-1.29	-0.62
Female	Belize	0.71	0.82	0.61	3.69	4.46	3.08	1.33	1.53	1.14	2.22	2.67	1.87	2.09	1.97	2.21
Female	Benin	10.14	13.59	6.39	23.45	31.18	16.26	0.72	0.96	0.47	0.71	0.94	0.50	-0.19	-0.27	-0.11
Female	Bermuda	1.71	1.96	1.48	2.69	3.29	2.19	4.79	5.47	4.12	4.94	6.05	4.00	-0.27	-0.45	-0.08
Female	Bhutan	3.64	6.21	2.14	12.34	21.02	7.25	1.89	3.27	1.16	2.88	4.80	1.82	1.48	1.34	1.61
Female	Bolivia	75.53	107.18	43.53	292.20	397.47	200.39	3.66	5.11	2.18	5.93	8.08	4.04	1.71	1.55	1.87

© 2020 Deng Y et al. *JAMA Network Open.*

Sex	Location	Deaths						Age-standardized death rate (per 100,000)						EAPC		
		1990(95%UI)			2017(95%UI)			1990(95%UI)			2017(95%UI)			1990-2017(95%CI)		
		Cases (No.)	UL	LL	Cases (No.)	UL	LL	95%UI	UL	LL	95%UI	UL	LL	EAPC	LL	UL
Female	Bosnia and Herzegovina	59.05	71.11	41.61	124.77	153.27	85.84	2.35	2.82	1.68	4.83	5.98	3.29	3.03	2.78	3.28
Female	Botswana	4.43	7.00	2.97	17.73	25.37	12.75	1.14	1.73	0.79	1.86	2.68	1.37	2.89	2.04	3.76
Female	Brazil	1315.84	1390.17	1249.19	3748.80	3952.83	3538.11	2.32	2.45	2.21	2.99	3.14	2.82	0.88	0.68	1.08
Female	Brunei	4.36	5.81	3.17	21.20	26.03	16.25	6.19	8.00	4.68	10.04	12.15	7.86	2.56	2.24	2.88
Female	Bulgaria	201.80	226.44	175.14	231.72	269.43	197.48	3.46	3.90	3.00	4.40	5.19	3.69	0.76	0.58	0.93
Female	Burkina Faso	28.82	40.18	17.33	51.26	67.00	38.41	0.97	1.34	0.59	0.81	1.03	0.60	-0.87	-0.99	-0.75
Female	Burundi	36.11	51.00	23.34	60.57	83.40	42.85	2.00	2.78	1.33	1.83	2.55	1.28	-0.41	-0.50	-0.32
Female	Cambodia	87.29	125.93	48.07	223.07	309.10	162.63	2.71	3.87	1.56	3.05	4.21	2.23	0.28	0.14	0.42
Female	Cameroon	35.28	45.50	20.73	71.99	101.40	50.64	1.12	1.44	0.69	0.89	1.25	0.61	-0.92	-1.02	-0.82
Female	Canada	858.38	963.04	759.21	1616.29	1886.28	1367.22	5.28	5.94	4.65	6.33	7.41	5.32	0.49	0.13	0.84
Female	Cape Verde	0.82	1.09	0.46	3.25	4.31	1.71	0.65	0.88	0.34	1.26	1.68	0.65	2.77	2.50	3.05
Female	Central African Republic	8.81	13.70	5.51	12.15	21.01	7.26	1.14	1.71	0.78	0.90	1.50	0.57	-0.83	-0.91	-0.74
Female	Chad	12.64	17.12	7.90	22.28	29.65	16.12	0.69	0.92	0.45	0.65	0.85	0.47	-0.29	-0.37	-0.21
Female	Chile	199.97	227.06	176.39	683.72	831.15	560.19	3.31	3.76	2.91	5.95	7.25	4.86	2.25	2.16	2.35
Female	China	8390.10	9412.36	6870.87	25186.34	30434.73	22437.97	1.62	1.83	1.36	2.63	3.18	2.34	1.51	1.13	1.88
Female	Colombia	464.15	524.17	416.45	1574.08	1948.99	1298.72	4.04	4.52	3.64	5.54	6.86	4.56	1.13	0.85	1.40
Female	Comoros	2.81	4.20	1.83	6.58	9.10	4.71	1.78	2.67	1.18	2.12	2.95	1.53	0.95	0.77	1.14
Female	Costa Rica	57.67	65.53	50.31	162.28	191.09	136.18	4.90	5.51	4.30	6.04	7.10	5.07	0.29	-0.08	0.65
Female	Croatia	216.26	247.62	185.37	195.55	229.02	164.45	6.08	6.93	5.24	5.25	6.17	4.43	0.40	0.03	0.78
Female	Cuba	187.69	216.59	165.03	450.23	546.83	365.17	3.49	4.03	3.07	5.44	6.60	4.43	1.64	1.30	1.99
Female	Cyprus	17.47	21.90	10.38	34.89	48.16	27.32	4.09	5.12	2.40	3.93	5.58	3.09	-0.53	-1.08	0.02

© 2020 Deng Y et al. *JAMA Network Open.*

Sex	Location	Deaths						Age-standardized death rate (per 100,000)						EAPC		
		1990(95%UI)			2017(95%UI)			1990(95%UI)			2017(95%UI)			1990-2017(95%CI)		
		Cases (No.)	UL	LL	Cases (No.)	UL	LL	95%UI	UL	LL	95%UI	UL	LL	EAPC	LL	UL
Female	Czech Republic	453.51	502.31	402.50	493.86	580.62	420.96	6.54	7.30	5.78	5.87	6.95	4.96	-0.56	-0.80	-0.31
Female	Democratic Republic of the Congo	100.19	149.33	70.03	209.39	339.40	141.70	0.94	1.36	0.68	0.92	1.47	0.62	-0.26	-0.46	-0.06
Female	Denmark	136.31	154.60	121.27	225.03	264.64	190.16	4.16	4.73	3.65	5.46	6.48	4.59	1.00	0.81	1.18
Female	Djibouti	2.43	3.77	1.49	9.19	14.29	5.71	1.75	2.69	1.11	2.13	3.28	1.35	0.73	0.58	0.89
Female	Dominica	0.76	0.86	0.67	1.44	1.68	1.23	2.20	2.52	1.91	3.66	4.32	3.10	1.91	1.70	2.12
Female	Dominican Republic	39.10	47.30	29.01	166.19	218.41	121.18	1.62	1.96	1.18	3.26	4.27	2.37	3.08	2.47	3.69
Female	Ecuador	65.54	73.54	58.41	682.32	828.04	557.14	1.95	2.15	1.74	8.54	10.38	7.00	6.12	5.46	6.79
Female	Egypt	266.77	443.93	204.61	1281.79	2060.66	866.18	1.35	2.38	1.04	3.14	5.19	2.13	3.32	3.12	3.53
Female	El Salvador	75.70	92.19	34.85	133.88	195.90	95.86	4.21	5.09	1.94	4.10	6.01	2.94	-0.03	-0.83	0.78
Female	Equatorial Guinea	1.42	2.28	0.82	7.11	12.70	3.71	1.10	1.73	0.69	1.86	3.26	1.02	2.43	2.14	2.72
Female	Eritrea	11.94	17.95	7.62	43.65	63.38	28.34	1.49	2.20	1.00	2.13	3.02	1.44	0.91	0.78	1.04
Female	Estonia	57.95	65.51	51.07	85.52	106.65	66.34	5.66	6.48	4.97	7.91	10.07	6.09	1.41	1.11	1.72
Female	Ethiopia	802.47	1194.73	408.75	1500.71	1989.88	1115.70	5.12	7.48	2.80	4.39	5.74	3.30	-0.91	-1.07	-0.75
Female	Fiji	12.29	16.47	6.03	27.79	37.50	12.82	5.20	6.98	2.56	6.41	8.54	3.01	0.73	0.47	0.99
Female	Finland	149.46	166.77	133.07	244.20	284.08	205.80	3.95	4.42	3.52	4.81	5.61	4.06	0.95	0.76	1.14
Female	France	1858.03	2037.79	1685.58	2102.80	2454.37	1786.60	4.85	5.35	4.38	4.13	4.87	3.50	-0.61	-1.01	-0.20
Female	Gabon	4.27	6.34	2.89	8.11	13.75	5.27	1.28	1.91	0.87	1.23	2.07	0.80	-0.11	-0.20	-0.02
Female	Gambia	2.15	2.76	1.62	6.66	9.30	5.00	0.81	1.04	0.63	0.99	1.38	0.73	0.91	0.79	1.03
Female	Georgia	78.81	92.77	63.62	131.24	153.12	110.31	2.32	2.72	1.86	5.05	5.89	4.21	3.28	2.19	4.38
Female	Germany	3336.61	3659.86	3025.25	4329.62	5201.49	3564.12	5.80	6.43	5.20	6.66	8.06	5.42	0.43	0.24	0.62
Female	Ghana	5.62	7.12	4.25	21.44	26.95	16.65	0.11	0.14	0.08	0.17	0.21	0.13	1.47	1.37	1.58
Female	Greece	231.30	259.98	204.62	361.26	416.89	307.73	3.23	3.63	2.85	4.17	4.85	3.55	0.89	0.65	1.13

© 2020 Deng Y et al. *JAMA Network Open.*

Sex	Location	Deaths						Age-standardized death rate (per 100,000)						EAPC		
		1990(95%UI)			2017(95%UI)			1990(95%UI)			2017(95%UI)			1990-2017(95%CI)		
		Cases (No.)	UL	LL	Cases (No.)	UL	LL	95%UI	UL	LL	95%UI	UL	LL	EAPC	LL	UL
Female	Greenland	0.43	0.54	0.35	1.10	1.44	0.56	2.15	2.56	1.70	3.37	4.30	1.78	2.05	1.42	2.69
Female	Grenada	1.72	1.96	1.49	2.69	3.12	2.29	4.80	5.50	4.09	4.28	5.03	3.61	0.24	-0.31	0.79
Female	Grenadines	1.82	2.13	1.58	4.05	4.77	3.41	4.54	5.32	3.90	6.40	7.56	5.41	1.40	1.05	1.75
Female	Guam	1.14	1.66	0.91	4.28	5.23	3.33	2.37	3.38	1.92	4.70	5.73	3.70	2.68	1.75	3.62
Female	Guatemala	43.06	48.53	38.34	197.09	237.85	159.39	1.86	2.08	1.67	2.90	3.49	2.36	0.13	-0.49	0.74
Female	Guinea	18.54	22.74	14.67	37.80	50.59	28.38	0.87	1.06	0.71	0.99	1.29	0.76	0.55	0.48	0.61
Female	Guinea-Bissau	2.90	3.93	1.57	4.29	6.20	2.83	0.98	1.30	0.56	0.81	1.17	0.55	-0.86	-1.02	-0.71
Female	Guyana	4.08	4.63	3.57	9.64	11.96	7.76	1.57	1.76	1.38	2.63	3.25	2.13	1.85	1.49	2.20
Female	Haiti	40.80	70.01	20.28	101.60	164.42	61.57	2.02	3.41	1.06	2.24	3.59	1.41	0.36	0.27	0.44
Female	Honduras	18.59	23.03	14.89	59.92	127.27	36.43	1.47	1.77	1.20	1.69	3.48	1.05	0.56	0.14	0.99
Female	Hungary	424.15	482.03	375.38	390.99	456.79	335.53	5.68	6.48	5.00	4.85	5.73	4.14	-0.51	-0.73	-0.29
Female	Iceland	21.08	23.86	18.29	24.12	28.01	20.61	15.64	17.76	13.49	10.87	12.70	9.27	-0.99	-1.31	-0.67
Female	India	5679.71	7529.92	4213.53	19152.28	21491.71	16040.84	1.69	2.25	1.29	2.95	3.30	2.48	1.96	1.74	2.17
Female	Indonesia	1673.18	2069.95	1141.81	3871.84	4879.65	2844.30	2.53	3.08	1.80	2.98	3.76	2.23	0.52	0.45	0.58
Female	Iran	381.47	484.48	298.02	2071.35	2398.72	1378.80	2.20	2.79	1.73	4.88	5.63	3.19	3.65	3.31	3.99
Female	Iraq	174.20	255.75	101.49	384.75	494.31	277.98	3.31	4.85	2.02	2.54	3.25	1.78	-1.16	-1.43	-0.88
Female	Ireland	81.46	93.04	72.12	177.75	212.09	147.93	4.20	4.85	3.68	5.56	6.68	4.62	1.54	1.30	1.78
Female	Israel	92.16	103.65	81.85	304.06	360.46	256.27	3.68	4.14	3.25	5.86	7.00	4.92	1.55	1.07	2.03
Female	Italy	3935.47	4376.13	3524.88	4376.37	5133.55	3684.26	10.03	11.26	8.91	8.81	10.46	7.33	-0.41	-0.81	-0.01
Female	Ivory Coast	34.53	44.70	25.56	87.79	125.64	61.04	1.00	1.26	0.78	1.08	1.48	0.79	0.39	0.34	0.45
Female	Jamaica	17.96	20.59	15.53	83.10	109.37	63.66	1.86	2.15	1.60	5.53	7.29	4.24	4.21	3.70	4.72
Female	Japan	4761.47	5056.25	4494.70	8233.16	9050.75	7556.02	5.40	5.77	5.09	6.68	7.46	6.07	0.90	0.70	1.10
Female	Jordan	51.86	70.26	33.82	169.93	244.36	123.40	4.70	6.28	3.21	4.49	6.35	3.29	-0.86	-1.50	-0.20

© 2020 Deng Y et al. *JAMA Network Open.*

Sex	Location	Deaths						Age-standardized death rate (per 100,000)						EAPC		
		1990(95%UI)			2017(95%UI)			1990(95%UI)			2017(95%UI)			1990-2017(95%CI)		
		Cases (No.)	UL	LL	Cases (No.)	UL	LL	95%UI	UL	LL	95%UI	UL	LL	EAPC	LL	UL
Female	Kazakhstan	484.17	591.74	413.10	549.56	657.64	462.52	5.92	7.22	5.03	5.35	6.40	4.50	-1.21	-1.69	-0.74
Female	Kenya	44.97	62.94	34.28	153.68	202.22	122.76	0.68	0.91	0.53	0.86	1.12	0.70	0.88	0.81	0.95
Female	Kiribati	0.17	0.20	0.14	0.34	0.44	0.25	0.74	0.88	0.60	0.80	1.03	0.60	0.42	0.34	0.49
Female	Kuwait	35.31	41.53	29.43	77.18	97.54	62.07	7.14	8.22	6.13	4.30	5.29	3.58	-1.93	-2.73	-1.13
Female	Kyrgyzstan	63.92	74.74	53.64	77.80	93.65	66.05	3.38	3.95	2.85	2.70	3.23	2.31	-0.41	-0.69	-0.12
Female	Laos	37.78	60.65	18.89	76.62	111.73	49.55	2.87	4.50	1.51	2.83	4.04	1.87	-0.35	-0.52	-0.18
Female	Latvia	90.25	102.20	79.35	115.75	146.85	91.43	5.06	5.78	4.37	6.47	8.23	5.11	0.96	0.64	1.27
Female	Lebanon	94.75	128.16	60.13	609.54	829.14	450.63	5.86	7.88	3.89	14.92	19.83	11.23	3.71	3.62	3.81
Female	Lesotho	6.55	9.17	4.87	10.86	17.21	6.74	1.10	1.55	0.82	1.42	2.23	0.88	1.64	1.33	1.95
Female	Liberia	5.85	7.76	3.54	9.98	13.93	6.60	0.91	1.20	0.56	0.73	1.02	0.49	-0.95	-1.20	-0.69
Female	Libya	52.79	72.39	37.99	331.08	455.43	236.91	4.09	5.63	3.02	9.63	13.10	7.01	3.98	3.59	4.37
Female	Lithuania	137.37	154.27	120.97	158.99	187.87	132.38	5.90	6.67	5.14	6.61	7.84	5.51	0.37	0.02	0.73
Female	Luxembourg	19.68	22.54	17.26	31.69	40.35	25.23	7.39	8.50	6.45	7.50	9.52	5.97	-0.22	-0.44	0.00
Female	Macedonia	32.29	40.28	19.02	60.62	76.17	42.67	3.01	3.73	1.80	4.13	5.20	2.91	0.85	0.62	1.09
Female	Madagascar	67.94	96.11	47.33	153.37	220.29	105.37	1.69	2.37	1.22	1.74	2.44	1.21	0.12	-0.04	0.29
Female	Malawi	64.65	91.18	28.26	154.75	226.00	99.70	1.96	2.68	1.02	2.44	3.43	1.65	0.35	0.11	0.59
Female	Malaysia	236.87	289.07	187.09	1120.51	1485.92	845.82	3.76	4.52	3.09	7.68	10.25	5.84	2.71	2.59	2.84
Female	Maldives	1.60	2.61	0.60	6.51	8.35	5.03	3.14	5.06	1.21	3.99	5.06	3.04	0.47	0.31	0.63
Female	Mali	50.11	61.22	38.71	127.56	194.40	81.99	1.73	2.09	1.42	2.01	2.94	1.33	0.35	0.26	0.45
Female	Malta	12.33	13.96	10.83	24.13	28.44	20.77	5.38	6.09	4.72	7.03	8.26	6.03	1.08	0.77	1.38
Female	Marshall Islands	0.27	0.37	0.18	0.94	1.46	0.53	2.55	3.51	1.76	4.27	6.38	2.56	1.92	1.67	2.16
Female	Mauritania	6.09	7.95	3.47	11.83	16.39	8.16	0.93	1.22	0.54	0.93	1.29	0.64	-0.20	-0.35	-0.06
Female	Mauritius	9.91	11.48	8.58	31.69	37.99	26.11	2.16	2.48	1.88	3.65	4.37	3.03	2.33	1.77	2.90

© 2020 Deng Y et al. *JAMA Network Open.*

Sex	Location	Deaths						Age-standardized death rate (per 100,000)						EAPC		
		1990(95%UI)			2017(95%UI)			1990(95%UI)			2017(95%UI)			1990-2017(95%CI)		
		Cases (No.)	UL	LL	Cases (No.)	UL	LL	95%UI	UL	LL	95%UI	UL	LL	EAPC	LL	UL
Female	Mexico	755.62	785.47	725.69	3712.39	3889.08	3538.48	2.76	2.86	2.65	5.68	5.94	5.42	2.42	2.21	2.63
Female	Micronesia	0.59	0.83	0.42	1.26	1.90	0.77	2.14	2.93	1.57	2.98	4.35	1.91	1.22	1.11	1.33
Female	Moldova	82.17	95.16	70.02	104.58	124.44	88.14	3.13	3.63	2.68	3.69	4.37	3.10	0.90	0.55	1.24
Female	Mongolia	7.15	8.50	5.07	26.16	34.10	15.38	1.16	1.39	0.76	1.87	2.45	0.96	2.21	1.88	2.55
Female	Montenegro	16.48	20.63	13.10	30.87	38.66	24.42	4.76	5.92	3.81	6.88	8.61	5.46	1.50	1.38	1.62
Female	Morocco	351.63	449.07	245.53	1214.17	1699.19	847.33	3.56	4.45	2.71	6.60	9.12	4.63	2.37	2.29	2.45
Female	Mozambique	74.85	113.09	52.67	196.37	283.42	123.92	1.49	2.30	1.07	1.95	2.79	1.29	0.94	0.81	1.08
Female	Myanmar	518.98	795.86	274.33	1051.15	1486.51	706.92	3.55	5.36	1.98	3.77	5.32	2.57	0.03	-0.06	0.13
Female	Namibia	5.83	8.23	3.76	15.39	21.09	10.79	1.22	1.81	0.80	1.54	2.15	1.09	0.50	-0.05	1.06
Female	Nepal	119.84	204.24	75.57	347.42	558.61	231.98	1.74	3.00	1.13	2.45	3.92	1.67	1.35	0.97	1.73
Female	Netherlands	423.08	469.44	375.29	837.64	991.20	702.17	4.42	4.94	3.89	6.25	7.33	5.28	1.48	1.28	1.68
Female	New Zealand	91.80	106.66	79.37	179.53	210.69	151.88	4.68	5.45	4.03	5.75	6.71	4.91	1.17	0.95	1.40
Female	Nicaragua	17.48	25.11	14.23	99.62	125.35	75.07	1.59	2.34	1.31	3.56	4.47	2.68	3.66	3.17	4.15
Female	Niger	14.12	18.75	8.47	28.43	38.77	20.20	0.69	0.88	0.44	0.57	0.76	0.41	-0.95	-1.08	-0.83
Female	Nigeria	119.28	175.72	65.48	350.73	528.29	221.63	0.51	0.76	0.27	0.62	0.92	0.40	0.74	0.66	0.81
Female	North Korea	273.11	380.17	193.99	458.35	696.90	312.62	2.54	3.56	1.81	2.87	4.37	1.96	0.37	0.19	0.54
Female	Northern Mariana Islands	0.39	0.54	0.27	1.10	1.46	0.84	2.96	3.89	2.21	3.57	4.60	2.82	0.49	-0.10	1.09
Female	Norway	142.22	154.59	130.65	252.98	283.25	225.76	5.21	5.73	4.75	6.94	7.79	6.18	1.16	0.85	1.46
Female	Oman	18.16	25.23	11.52	98.53	130.42	69.79	3.70	5.04	2.47	7.26	9.58	5.38	2.56	2.33	2.78
Female	Pakistan	1879.50	2627.58	1425.17	7948.92	12615.31	5059.13	4.85	6.53	3.80	8.47	12.86	5.45	2.10	1.95	2.25
Female	Palestine	15.34	21.03	10.79	41.85	53.51	34.06	2.65	3.59	1.87	2.72	3.32	2.21	0.15	-0.02	0.32
Female	Panama	29.18	33.08	25.46	107.69	127.52	90.27	3.03	3.41	2.68	5.33	6.30	4.47	2.09	1.94	2.24

© 2020 Deng Y et al. *JAMA Network Open.*

Sex	Location	Deaths						Age-standardized death rate (per 100,000)						EAPC		
		1990(95%UI)			2017(95%UI)			1990(95%UI)			2017(95%UI)			1990-2017(95%CI)		
		Cases (No.)	UL	LL	Cases (No.)	UL	LL	95%UI	UL	LL	95%UI	UL	LL	EAPC	LL	UL
Female	Papua New Guinea	23.80	36.57	16.22	80.57	122.44	52.40	2.09	3.11	1.46	2.68	3.93	1.87	0.93	0.75	1.11
Female	Paraguay	27.43	33.14	22.94	116.02	159.60	76.58	2.06	2.47	1.69	3.89	5.33	2.54	2.36	2.12	2.60
Female	Peru	190.44	237.12	158.03	896.74	1168.12	641.36	2.54	3.12	2.11	5.54	7.19	3.95	3.10	2.69	3.50
Female	Philippines	871.14	1045.42	739.24	3914.13	4967.91	3065.18	4.30	5.08	3.71	8.79	11.12	6.92	2.95	2.76	3.13
Female	Poland	1302.91	1436.88	1174.05	1933.16	2244.72	1635.39	5.54	6.14	4.97	6.61	7.80	5.57	0.38	0.12	0.64
Female	Portugal	398.91	446.57	352.10	556.28	659.93	464.47	5.60	6.29	4.96	5.64	6.77	4.71	-0.48	-0.79	-0.17
Female	Puerto Rico	86.57	99.68	75.06	94.80	113.30	79.72	4.46	5.14	3.86	3.60	4.34	3.00	-0.88	-1.28	-0.48
Female	Qatar	7.36	10.84	2.10	22.51	31.93	16.17	9.59	13.35	2.91	4.67	6.55	3.38	-3.34	-4.01	-2.67
Female	Republic of Congo	9.72	15.38	5.67	25.86	45.71	15.18	1.36	2.09	0.86	1.50	2.57	0.95	0.35	0.16	0.53
Female	Romania	497.33	558.25	438.63	856.28	997.44	719.74	3.50	3.92	3.08	5.86	6.83	4.94	1.66	1.42	1.91
Female	Russia	4251.69	4848.00	3807.59	8336.47	9092.32	7643.64	4.13	4.73	3.69	7.66	8.35	6.95	2.54	2.15	2.94
Female	Rwanda	48.41	71.52	27.11	95.47	144.38	68.33	2.01	2.98	1.15	1.96	2.93	1.44	-0.34	-0.65	-0.02
Female	Saint Lucia	2.47	2.80	2.18	7.08	8.42	5.90	4.86	5.53	4.23	6.55	7.74	5.49	0.93	0.49	1.37
Female	Saint Vincent	1.82	2.13	1.58	4.05	4.77	3.41	4.54	5.32	3.90	6.40	7.56	5.41	1.40	1.05	1.75
Female	Samoa	1.95	2.53	1.43	4.02	5.56	2.71	3.52	4.60	2.60	5.10	7.04	3.46	1.30	1.12	1.48
Female	Sao Tome and Principe	0.34	0.54	0.21	0.75	1.03	0.55	0.79	1.24	0.50	1.05	1.40	0.76	0.63	0.40	0.86
Female	Saudi Arabia	145.80	201.13	106.06	1827.39	2602.65	1207.27	3.32	4.59	2.43	12.12	16.37	8.81	5.66	5.23	6.08
Female	Senegal	15.80	20.57	9.87	30.43	40.05	22.84	0.71	0.91	0.46	0.66	0.86	0.49	-0.62	-0.78	-0.46
Female	Serbia	219.16	274.17	165.94	321.21	384.75	240.73	3.75	4.65	2.88	4.86	5.87	3.70	1.30	1.15	1.46
Female	Seychelles	0.46	0.55	0.30	1.26	1.53	1.01	1.52	1.83	0.97	2.18	2.65	1.75	1.49	1.35	1.63
Female	Sierra Leone	9.85	13.88	6.13	19.96	27.52	13.99	0.79	1.10	0.50	0.87	1.19	0.60	0.39	0.29	0.49
Female	Singapore	65.82	75.69	57.03	206.75	252.76	169.65	4.31	4.92	3.77	5.76	7.06	4.73	1.61	1.34	1.88

© 2020 Deng Y et al. *JAMA Network Open.*

Sex	Location	Deaths						Age-standardized death rate (per 100,000)						EAPC		
		1990(95%UI)			2017(95%UI)			1990(95%UI)			2017(95%UI)			1990-2017(95%CI)		
		Cases (No.)	UL	LL	Cases (No.)	UL	LL	95%UI	UL	LL	95%UI	UL	LL	EAPC	LL	UL
Female	Slovakia	151.43	176.56	123.32	204.63	275.99	163.23	4.82	5.61	3.90	4.83	6.63	3.83	-0.19	-0.34	-0.04
Female	Slovenia	70.42	79.69	61.94	83.56	100.16	69.14	5.41	6.12	4.74	5.17	6.23	4.25	-0.22	-0.44	-0.01
Female	Solomon Islands	1.54	2.33	1.04	4.67	6.94	3.09	1.92	2.80	1.39	2.28	3.30	1.61	0.47	0.29	0.65
Female	Somalia	28.87	51.02	12.36	80.19	117.54	55.37	1.44	2.41	0.75	1.61	2.33	1.10	0.23	0.06	0.39
Female	South Africa	221.21	251.58	179.44	382.04	504.17	331.97	1.51	1.71	1.26	1.39	1.82	1.21	-0.88	-1.47	-0.29
Female	South Korea	421.88	476.50	366.04	6573.02	7953.50	5505.40	2.06	2.31	1.80	19.85	24.29	16.41	11.05	9.06	13.08
Female	South Sudan	29.01	52.77	12.77	53.67	82.79	33.67	1.69	2.92	0.86	1.79	2.76	1.16	0.16	-0.02	0.33
Female	Spain	870.74	962.83	790.42	1567.67	1827.27	1344.91	3.36	3.74	3.02	4.16	4.89	3.56	0.91	0.66	1.16
Female	Sri Lanka	219.90	267.11	155.12	700.77	988.18	472.05	3.35	4.04	2.36	5.20	7.30	3.54	1.15	0.76	1.54
Female	Sudan	97.07	169.43	51.11	303.41	458.57	183.84	1.60	2.75	0.92	2.37	3.50	1.46	1.50	1.40	1.61
Female	Suriname	3.79	4.38	3.28	12.18	14.68	10.03	2.50	2.88	2.17	3.84	4.61	3.16	1.67	1.35	2.00
Female	Swaziland	3.15	4.50	2.28	6.01	9.67	3.64	1.49	2.12	1.08	1.55	2.54	0.93	0.25	-0.04	0.55
Female	Sweden	213.08	235.21	192.53	313.63	362.22	272.82	3.34	3.73	3.00	4.27	4.91	3.67	0.95	0.71	1.19
Female	Switzerland	321.22	362.55	278.63	371.97	447.91	313.99	7.25	8.23	6.22	5.63	6.83	4.74	-0.83	-1.14	-0.51
Female	Syria	28.71	40.58	17.26	112.38	154.86	76.82	0.88	1.24	0.52	1.45	1.97	0.99	1.64	1.46	1.82
Female	Tajikistan	7.78	9.90	6.46	22.55	28.07	18.40	0.45	0.57	0.37	0.65	0.79	0.53	0.97	0.62	1.33
Female	Tanzania	151.90	220.72	91.10	415.21	606.25	288.72	1.78	2.60	1.18	2.13	3.06	1.52	0.32	0.04	0.60
Female	Thailand	663.09	1073.40	531.59	2105.37	4859.93	1471.29	2.86	4.52	2.32	4.10	9.60	2.84	0.96	0.47	1.44
Female	Timor-Leste	4.41	6.49	2.52	12.22	16.70	8.12	2.21	3.13	1.43	2.82	3.85	1.94	1.00	0.74	1.27
Female	Tobago	14.66	16.73	12.75	34.30	47.01	24.12	2.95	3.34	2.57	3.92	5.43	2.73	0.45	-0.03	0.93
Female	Togo	8.57	12.24	5.24	17.61	23.40	12.74	0.82	1.15	0.52	0.67	0.89	0.49	-0.93	-1.15	-0.72
Female	Tonga	0.52	0.67	0.41	1.10	1.47	0.79	1.68	2.14	1.32	2.52	3.37	1.81	1.54	1.35	1.73
Female	Trinidad	14.66	16.73	12.75	34.30	47.01	24.12	2.95	3.34	2.57	3.92	5.43	2.73	0.45	-0.03	0.93

© 2020 Deng Y et al. *JAMA Network Open.*

Sex	Location	Deaths						Age-standardized death rate (per 100,000)						EAPC		
		1990(95%UI)			2017(95%UI)			1990(95%UI)			2017(95%UI)			1990-2017(95%CI)		
		Cases (No.)	UL	LL	Cases (No.)	UL	LL	95%UI	UL	LL	95%UI	UL	LL	EAPC	LL	UL
Female	Tunisia	96.89	119.81	78.24	381.37	536.40	260.12	2.92	3.66	2.39	5.85	8.22	4.02	2.44	2.15	2.74
Female	Turkey	563.67	892.39	358.90	2187.05	3547.43	1690.42	2.62	4.04	1.69	4.74	7.69	3.66	2.58	2.30	2.86
Female	Turkmenistan	25.38	28.94	22.10	61.83	73.50	51.05	1.97	2.24	1.71	2.52	3.00	2.10	0.94	0.23	1.66
Female	Uganda	89.42	116.58	68.79	264.36	376.34	181.21	1.71	2.21	1.34	2.06	2.83	1.45	0.67	0.54	0.81
Female	UK	1238.02	1292.88	1178.63	2194.36	2307.33	2093.25	3.13	3.27	2.98	4.58	4.82	4.37	1.41	1.26	1.56
Female	Ukraine	1174.79	1398.12	995.29	1924.50	2242.56	1646.88	3.02	3.56	2.56	5.54	6.52	4.71	2.32	1.95	2.69
Female	United Arab Emirates	12.20	18.83	7.42	149.15	227.93	92.47	3.69	5.92	2.29	6.45	9.99	3.88	2.27	1.94	2.60
Female	Uruguay	37.39	42.78	32.95	89.83	110.43	71.82	2.00	2.31	1.75	4.02	4.99	3.18	2.04	1.39	2.70
Female	USA	6796.91	7037.91	6569.50	15491.78	16328.45	14684.06	4.32	4.48	4.16	6.47	6.84	6.10	1.49	1.22	1.76
Female	Uzbekistan	39.94	55.08	33.42	178.56	219.41	141.87	0.54	0.75	0.45	1.18	1.45	0.94	3.20	3.01	3.39
Female	Vanuatu	0.81	1.30	0.51	2.43	4.28	1.51	2.10	3.35	1.40	2.57	4.42	1.64	0.78	0.72	0.84
Female	Venezuela	132.64	151.81	117.52	709.69	899.01	534.07	2.09	2.35	1.86	4.44	5.63	3.34	2.78	2.47	3.10
Female	Vietnam	1056.30	1351.67	835.79	5310.83	7306.41	3868.66	3.89	5.21	3.05	9.65	13.26	7.03	4.07	3.74	4.40
Female	Virgin Islands	0.95	1.13	0.79	2.45	3.01	1.96	1.73	2.04	1.47	3.07	3.82	2.44	2.58	2.33	2.83
Female	Yemen	49.85	94.84	24.34	212.41	337.04	130.12	1.43	2.65	0.77	2.22	3.45	1.40	1.82	1.74	1.90
Female	Zambia	60.05	88.37	35.94	109.46	155.81	77.55	2.44	3.49	1.56	1.93	2.72	1.38	-1.29	-1.95	-0.62
Female	Zimbabwe	65.93	83.70	43.33	133.61	180.53	74.53	2.28	2.85	1.65	2.58	3.43	1.68	0.82	0.33	1.32
Male	Afghanistan	19.45	34.28	8.69	27.75	42.68	18.67	0.50	0.87	0.23	0.50	0.74	0.35	0.07	-0.06	0.20
Male	Albania	5.68	6.45	4.99	9.36	12.01	7.14	0.56	0.63	0.48	0.51	0.65	0.40	-0.56	-0.86	-0.27
Male	Algeria	27.49	32.71	22.79	84.42	101.17	68.40	0.41	0.49	0.34	0.50	0.59	0.40	1.00	0.84	1.16
Male	American Samoa	0.04	0.05	0.03	0.07	0.09	0.06	0.36	0.45	0.29	0.37	0.45	0.30	0.08	-0.37	0.55
Male	Andorra	0.10	0.13	0.08	0.21	0.28	0.15	0.35	0.45	0.27	0.32	0.43	0.24	-0.30	-0.35	-0.24
Male	Angola	6.83	9.65	4.58	16.31	20.73	12.09	0.34	0.46	0.24	0.34	0.44	0.26	-0.02	-0.11	0.07

© 2020 Deng Y et al. *JAMA Network Open.*

Sex	Location	Deaths						Age-standardized death rate (per 100,000)						EAPC		
		1990(95%UI)			2017(95%UI)			1990(95%UI)			2017(95%UI)			1990-2017(95%CI)		
		Cases (No.)	UL	LL	Cases (No.)	UL	LL	95%UI	UL	LL	95%UI	UL	LL	EAPC	LL	UL
Male	Antigua	0.05	0.05	0.04	0.10	0.11	0.09	0.21	0.23	0.18	0.21	0.24	0.19	0.20	0.09	0.31
Male	Argentina	84.22	90.40	77.82	100.91	117.45	85.04	0.58	0.63	0.54	0.44	0.52	0.37	-1.10	-1.33	-0.88
Male	Armenia	2.29	3.11	1.80	10.25	11.59	8.95	0.21	0.28	0.17	0.59	0.67	0.52	3.84	2.83	4.85
Male	Australia	28.99	31.48	26.72	73.66	86.64	62.02	0.33	0.36	0.30	0.39	0.46	0.33	1.09	0.91	1.26
Male	Austria	31.34	34.22	28.54	34.83	40.04	30.23	0.69	0.75	0.63	0.45	0.52	0.39	-2.35	-2.64	-2.05
Male	Azerbaijan	7.31	8.52	6.19	13.22	15.83	10.80	0.33	0.39	0.28	0.35	0.42	0.29	-0.92	-1.34	-0.50
Male	Bahamas	0.38	0.43	0.33	0.99	1.16	0.84	0.52	0.59	0.46	0.57	0.66	0.49	0.56	0.44	0.67
Male	Bahrain	0.35	0.41	0.29	1.18	1.43	0.95	0.37	0.45	0.31	0.24	0.29	0.20	-2.41	-2.83	-1.98
Male	Bangladesh	120.40	154.11	94.41	212.72	265.30	160.78	0.43	0.55	0.33	0.33	0.41	0.25	-1.04	-1.24	-0.85
Male	Barbados	0.51	0.57	0.45	0.94	1.09	0.80	0.41	0.46	0.37	0.44	0.51	0.38	0.41	0.16	0.66
Male	Barbuda	0.05	0.05	0.04	0.10	0.11	0.09	0.21	0.23	0.18	0.21	0.24	0.19	0.20	0.09	0.31
Male	Belarus	16.98	19.35	15.05	27.76	34.12	23.44	0.35	0.40	0.31	0.47	0.57	0.40	1.04	0.60	1.49
Male	Belgium	40.06	44.11	36.43	47.98	55.78	41.03	0.63	0.69	0.58	0.49	0.57	0.42	-0.57	-0.88	-0.25
Male	Belize	0.09	0.11	0.08	0.42	0.47	0.37	0.19	0.22	0.16	0.30	0.34	0.27	1.62	1.16	2.09
Male	Benin	2.89	3.53	2.38	6.29	8.67	4.40	0.29	0.35	0.24	0.30	0.41	0.21	0.12	0.04	0.19
Male	Bermuda	0.22	0.25	0.19	0.40	0.45	0.35	0.83	0.93	0.72	0.75	0.85	0.66	0.08	-0.15	0.31
Male	Bhutan	0.61	0.77	0.47	1.56	2.25	1.02	0.43	0.56	0.33	0.49	0.70	0.32	0.61	0.47	0.74
Male	Bolivia	10.54	14.45	7.94	32.14	43.12	23.91	0.69	0.93	0.53	0.82	1.10	0.61	0.77	0.61	0.94
Male	Bosnia and Herzegovina	7.47	8.58	6.50	9.09	10.76	7.64	0.43	0.49	0.38	0.34	0.40	0.29	-1.44	-1.71	-1.17
Male	Botswana	0.86	1.17	0.62	1.66	2.40	1.13	0.33	0.43	0.24	0.30	0.42	0.21	-0.31	-0.42	-0.19
Male	Brazil	192.11	200.41	184.50	461.22	485.23	434.56	0.47	0.49	0.45	0.47	0.49	0.44	0.11	0.00	0.22
Male	Brunei	0.30	0.37	0.24	0.83	0.99	0.72	0.61	0.76	0.50	0.63	0.75	0.53	0.44	0.28	0.59

© 2020 Deng Y et al. *JAMA Network Open.*

Sex	Location	Deaths						Age-standardized death rate (per 100,000)						EAPC		
		1990(95%UI)			2017(95%UI)			1990(95%UI)			2017(95%UI)			1990-2017(95%CI)		
		Cases (No.)	UL	LL	Cases (No.)	UL	LL	95%UI	UL	LL	95%UI	UL	LL	EAPC	LL	UL
Male	Bulgaria	25.97	28.63	23.46	21.94	25.33	19.12	0.44	0.49	0.40	0.36	0.42	0.32	-1.04	-1.23	-0.85
Male	Burkina Faso	5.32	7.14	3.86	9.69	11.93	7.79	0.26	0.34	0.19	0.25	0.30	0.20	-0.31	-0.46	-0.15
Male	Burundi	5.72	8.31	3.75	11.64	15.02	8.83	0.51	0.72	0.35	0.50	0.63	0.39	-0.04	-0.12	0.05
Male	Cambodia	13.47	19.23	9.73	28.84	36.10	23.01	0.68	0.96	0.50	0.65	0.80	0.53	-0.17	-0.22	-0.12
Male	Cameroon	6.90	8.29	5.66	17.82	24.25	12.60	0.32	0.38	0.26	0.33	0.45	0.24	0.13	-0.02	0.27
Male	Canada	49.24	52.77	45.72	107.26	122.27	93.87	0.34	0.37	0.32	0.36	0.40	0.31	0.30	0.12	0.48
Male	Cape Verde	0.16	0.23	0.12	0.36	0.47	0.25	0.17	0.24	0.12	0.19	0.24	0.13	0.06	-0.12	0.23
Male	Central African Republic	2.06	3.14	1.31	3.42	5.14	2.29	0.37	0.54	0.25	0.33	0.47	0.24	-0.47	-0.52	-0.42
Male	Chad	3.34	4.27	2.44	7.09	8.94	5.54	0.24	0.30	0.17	0.25	0.31	0.19	0.11	-0.06	0.28
Male	Chile	23.45	26.17	21.00	46.38	54.31	39.27	0.52	0.58	0.46	0.45	0.53	0.38	-0.38	-0.49	-0.27
Male	China	1157.97	1243.80	1082.81	3935.72	4216.74	3643.32	0.30	0.32	0.28	0.46	0.49	0.43	2.39	2.00	2.78
Male	Colombia	53.33	57.70	49.30	102.99	124.10	85.32	0.61	0.66	0.56	0.42	0.51	0.35	-1.59	-1.87	-1.31
Male	Comoros	0.48	0.61	0.37	1.10	1.56	0.80	0.43	0.54	0.33	0.50	0.71	0.37	0.74	0.54	0.94
Male	Costa Rica	4.25	4.69	3.90	10.59	12.15	9.13	0.48	0.53	0.44	0.47	0.54	0.40	-0.26	-0.60	0.09
Male	Croatia	18.09	20.14	16.25	13.18	15.14	11.61	0.69	0.76	0.62	0.36	0.42	0.32	-1.92	-2.24	-1.60
Male	Cuba	16.69	18.40	15.13	41.18	48.68	34.46	0.33	0.36	0.30	0.47	0.56	0.40	1.58	1.40	1.77
Male	Cyprus	2.12	2.60	1.77	4.06	4.87	3.30	0.55	0.67	0.46	0.47	0.57	0.39	-0.49	-0.77	-0.21
Male	Czech Republic	39.14	42.70	35.55	30.46	35.21	26.21	0.69	0.75	0.63	0.35	0.40	0.30	-2.18	-2.41	-1.95
Male	Democratic Republic of the Congo	21.43	28.29	15.76	41.60	55.81	29.09	0.30	0.38	0.22	0.28	0.38	0.19	-0.30	-0.43	-0.18
Male	Denmark	8.55	9.64	7.65	14.52	16.73	12.36	0.25	0.27	0.22	0.29	0.33	0.25	0.43	0.02	0.84
Male	Djibouti	0.38	0.55	0.26	1.84	2.72	1.18	0.38	0.53	0.28	0.56	0.80	0.38	1.51	1.44	1.59

© 2020 Deng Y et al. *JAMA Network Open.*

Sex	Location	Deaths						Age-standardized death rate (per 100,000)						EAPC		
		1990(95%UI)			2017(95%UI)			1990(95%UI)			2017(95%UI)			1990-2017(95%CI)		
		Cases (No.)	UL	LL	Cases (No.)	UL	LL	95%UI	UL	LL	95%UI	UL	LL	EAPC	LL	UL
Male	Dominica	0.12	0.13	0.11	0.24	0.28	0.21	0.40	0.44	0.36	0.55	0.63	0.48	1.28	1.18	1.37
Male	Dominican Republic	6.30	7.33	5.46	17.27	22.10	13.19	0.33	0.38	0.28	0.39	0.49	0.30	0.07	-0.54	0.68
Male	Ecuador	9.60	10.40	8.74	45.81	53.23	38.95	0.36	0.39	0.33	0.66	0.77	0.56	2.77	1.97	3.58
Male	Egypt	36.70	42.29	31.49	90.88	120.01	67.39	0.24	0.27	0.21	0.29	0.37	0.21	0.81	0.60	1.02
Male	El Salvador	7.13	8.09	6.30	8.18	10.53	6.34	0.52	0.59	0.46	0.34	0.44	0.26	-1.67	-2.23	-1.12
Male	Equatorial Guinea	0.35	0.54	0.22	0.67	0.95	0.41	0.39	0.58	0.27	0.34	0.47	0.21	-0.75	-1.04	-0.46
Male	Eritrea	3.48	5.10	1.95	10.04	14.40	6.85	0.65	0.91	0.45	0.81	1.13	0.56	0.57	0.18	0.97
Male	Estonia	2.80	3.13	2.52	3.85	4.87	3.10	0.37	0.41	0.34	0.40	0.50	0.32	-0.35	-0.95	0.26
Male	Ethiopia	196.97	331.42	110.76	313.67	392.54	244.68	1.77	2.85	1.08	1.39	1.76	1.08	-1.02	-1.14	-0.90
Male	Fiji	1.80	2.25	1.40	2.23	2.71	1.83	0.95	1.22	0.72	0.69	0.83	0.57	-1.68	-2.19	-1.16
Male	Finland	11.80	12.95	10.72	22.35	25.78	19.04	0.43	0.47	0.39	0.43	0.50	0.37	0.30	0.17	0.43
Male	France	177.72	189.07	167.08	243.82	278.88	213.67	0.53	0.56	0.49	0.43	0.49	0.38	-0.39	-1.53	0.76
Male	Gabon	0.84	1.06	0.69	1.75	2.21	1.35	0.34	0.41	0.28	0.36	0.45	0.28	0.31	0.25	0.37
Male	Gambia	0.43	0.57	0.32	1.22	1.57	0.91	0.23	0.29	0.18	0.28	0.35	0.21	0.82	0.71	0.92
Male	Georgia	5.80	6.76	4.75	11.58	13.31	9.97	0.24	0.27	0.20	0.48	0.55	0.42	2.85	1.84	3.86
Male	Germany	312.42	328.01	295.23	438.05	506.23	373.24	0.64	0.67	0.60	0.53	0.62	0.46	-1.10	-1.56	-0.64
Male	Ghana	9.85	12.81	7.42	23.24	28.76	18.21	0.34	0.44	0.26	0.40	0.50	0.31	0.56	0.46	0.65
Male	Greece	24.00	26.34	21.73	37.90	43.39	32.96	0.35	0.38	0.31	0.35	0.40	0.30	-0.39	-0.69	-0.09
Male	Greenland	0.05	0.06	0.04	0.09	0.11	0.08	0.32	0.40	0.26	0.29	0.33	0.24	-0.33	-0.57	-0.10
Male	Grenada	0.31	0.35	0.28	0.54	0.62	0.47	1.07	1.19	0.95	0.73	0.84	0.64	-0.47	-0.96	0.02
Male	Grenadines	0.12	0.13	0.10	0.35	0.40	0.30	0.35	0.40	0.31	0.51	0.58	0.44	1.44	1.30	1.58
Male	Guam	0.11	0.13	0.09	0.45	0.53	0.37	0.30	0.36	0.25	0.53	0.63	0.45	2.49	1.46	3.54
Male	Guatemala	6.45	7.14	5.84	17.15	20.09	14.43	0.35	0.39	0.32	0.35	0.41	0.29	-1.71	-2.37	-1.04

© 2020 Deng Y et al. *JAMA Network Open.*

Sex	Location	Deaths						Age-standardized death rate (per 100,000)						EAPC		
		1990(95%UI)			2017(95%UI)			1990(95%UI)			2017(95%UI)			1990-2017(95%CI)		
		Cases (No.)	UL	LL	Cases (No.)	UL	LL	95%UI	UL	LL	95%UI	UL	LL	EAPC	LL	UL
Male	Guinea	5.96	7.08	4.99	11.62	15.78	8.72	0.34	0.40	0.29	0.40	0.55	0.30	0.78	0.71	0.84
Male	Guinea-Bissau	0.72	1.03	0.47	1.07	1.58	0.68	0.35	0.50	0.24	0.34	0.48	0.22	-0.14	-0.20	-0.09
Male	Guyana	0.84	0.94	0.76	1.64	1.96	1.35	0.43	0.48	0.39	0.55	0.66	0.46	1.53	1.26	1.79
Male	Haiti	8.22	13.00	6.19	17.16	23.94	11.97	0.52	0.79	0.40	0.57	0.78	0.40	0.49	0.40	0.57
Male	Honduras	8.63	10.57	7.03	29.72	39.81	19.29	0.84	1.02	0.68	1.05	1.40	0.68	0.79	0.68	0.91
Male	Hungary	44.71	48.80	40.88	30.26	34.40	26.59	0.72	0.79	0.66	0.40	0.45	0.35	-2.31	-2.55	-2.07
Male	Iceland	1.51	1.66	1.36	3.40	3.83	2.99	1.13	1.25	1.03	1.38	1.54	1.22	0.77	0.57	0.97
Male	India	920.14	1097.81	802.99	2546.21	2705.72	2362.67	0.36	0.42	0.31	0.48	0.51	0.44	1.35	1.22	1.48
Male	Indonesia	209.33	242.39	184.32	493.26	573.66	433.40	0.44	0.50	0.38	0.53	0.61	0.47	0.75	0.65	0.84
Male	Iran	31.30	38.45	27.30	109.87	118.03	100.69	0.23	0.28	0.20	0.32	0.34	0.29	2.18	1.72	2.64
Male	Iraq	21.23	28.45	15.82	42.75	49.61	37.00	0.53	0.70	0.40	0.36	0.42	0.31	-1.50	-1.72	-1.29
Male	Ireland	8.65	9.68	7.72	10.52	12.66	8.95	0.47	0.52	0.42	0.32	0.38	0.27	-0.99	-1.30	-0.68
Male	Israel	11.72	13.20	10.53	29.62	34.93	25.26	0.52	0.59	0.47	0.58	0.69	0.50	0.14	-0.18	0.45
Male	Italy	205.39	217.26	193.27	268.96	306.41	235.25	0.54	0.57	0.51	0.43	0.49	0.37	-0.72	-0.80	-0.63
Male	Ivory Coast	4.14	5.03	3.35	11.81	15.69	8.13	0.20	0.25	0.16	0.24	0.31	0.17	0.49	0.29	0.68
Male	Jamaica	2.17	2.50	1.91	5.90	7.50	4.28	0.26	0.30	0.23	0.43	0.54	0.31	1.96	1.59	2.33
Male	Japan	323.25	333.64	313.32	684.53	739.45	645.61	0.46	0.47	0.45	0.42	0.46	0.40	-0.35	-0.51	-0.20
Male	Jordan	4.06	5.78	2.97	11.01	13.59	8.83	0.52	0.73	0.38	0.38	0.47	0.30	-1.44	-1.57	-1.30
Male	Kazakhstan	42.70	53.08	36.40	25.40	30.11	21.88	0.78	0.96	0.67	0.36	0.42	0.31	-3.96	-4.33	-3.58
Male	Kenya	9.14	10.77	6.94	28.55	35.15	22.33	0.21	0.24	0.16	0.27	0.33	0.21	1.05	0.84	1.26
Male	Kiribati	0.05	0.06	0.05	0.09	0.12	0.07	0.34	0.40	0.29	0.36	0.45	0.29	0.39	0.21	0.57
Male	Kuwait	1.51	1.68	1.36	5.13	6.04	4.28	0.38	0.42	0.34	0.38	0.44	0.31	1.37	0.65	2.10
Male	Kyrgyzstan	4.08	4.67	3.53	4.59	5.54	3.93	0.32	0.37	0.28	0.24	0.28	0.21	-0.75	-1.05	-0.45

© 2020 Deng Y et al. *JAMA Network Open.*

Sex	Location	Deaths						Age-standardized death rate (per 100,000)						EAPC		
		1990(95%UI)			2017(95%UI)			1990(95%UI)			2017(95%UI)			1990-2017(95%CI)		
		Cases (No.)	UL	LL	Cases (No.)	UL	LL	95%UI	UL	LL	95%UI	UL	LL	EAPC	LL	UL
Male	Laos	6.26	9.39	4.36	11.13	13.98	8.37	0.61	0.89	0.43	0.57	0.72	0.44	-0.27	-0.32	-0.23
Male	Latvia	4.45	4.96	4.00	6.84	8.25	5.46	0.33	0.37	0.30	0.48	0.57	0.38	1.36	0.86	1.85
Male	Lebanon	5.43	6.87	4.33	15.09	18.41	11.29	0.52	0.65	0.42	0.54	0.66	0.40	0.32	0.14	0.49
Male	Lesotho	1.49	1.98	1.15	2.18	2.97	1.57	0.35	0.47	0.27	0.45	0.59	0.34	1.31	1.10	1.51
Male	Liberia	1.68	2.11	1.28	2.43	3.25	1.74	0.27	0.34	0.21	0.25	0.34	0.19	-0.37	-0.53	-0.21
Male	Libya	2.77	3.53	2.11	7.26	9.19	5.54	0.25	0.32	0.19	0.29	0.36	0.23	0.77	0.61	0.93
Male	Lithuania	6.49	7.30	5.83	7.73	8.81	6.69	0.36	0.40	0.32	0.37	0.42	0.32	-0.98	-1.65	-0.30
Male	Luxembourg	2.19	2.44	1.95	3.02	3.63	2.49	0.97	1.08	0.86	0.69	0.83	0.57	-1.54	-1.75	-1.33
Male	Macedonia	3.92	4.53	3.41	4.75	5.68	3.85	0.44	0.50	0.38	0.31	0.37	0.26	-2.33	-2.93	-1.73
Male	Madagascar	11.28	13.86	9.00	25.46	32.79	19.08	0.35	0.43	0.28	0.44	0.55	0.33	1.06	0.94	1.18
Male	Malawi	3.83	5.07	2.17	8.30	10.19	6.36	0.19	0.25	0.12	0.23	0.28	0.18	0.49	0.29	0.68
Male	Malaysia	27.15	32.07	23.02	73.85	94.41	59.86	0.59	0.71	0.50	0.60	0.75	0.49	-0.06	-0.14	0.02
Male	Maldives	0.93	1.48	0.63	1.97	2.37	1.63	1.93	2.92	1.38	1.39	1.69	1.15	-1.55	-1.79	-1.32
Male	Mali	7.29	8.84	6.02	14.62	19.07	9.86	0.34	0.41	0.29	0.32	0.42	0.22	-0.05	-0.21	0.11
Male	Malta	0.89	0.99	0.79	1.79	2.05	1.56	0.47	0.53	0.43	0.46	0.52	0.41	-0.15	-0.43	0.13
Male	Marshall Islands	0.06	0.08	0.04	0.13	0.18	0.09	0.65	0.90	0.46	0.79	1.07	0.56	0.77	0.56	0.98
Male	Mauritania	1.42	1.74	1.14	2.82	4.02	1.63	0.29	0.36	0.23	0.30	0.42	0.17	0.11	0.02	0.20
Male	Mauritius	1.05	1.18	0.93	2.42	2.80	2.07	0.31	0.34	0.27	0.32	0.37	0.28	0.63	0.23	1.02
Male	Mexico	95.31	98.82	92.14	271.62	286.74	251.45	0.46	0.47	0.44	0.52	0.55	0.48	0.49	0.35	0.63
Male	Micronesia	0.13	0.17	0.10	0.19	0.26	0.13	0.53	0.67	0.42	0.62	0.79	0.46	0.65	0.52	0.79
Male	Moldova	7.63	8.84	6.55	9.42	10.73	8.22	0.39	0.45	0.34	0.40	0.45	0.35	0.21	-0.38	0.81
Male	Mongolia	2.06	2.42	1.71	4.59	5.72	3.65	0.41	0.47	0.34	0.44	0.54	0.35	0.22	-0.03	0.47
Male	Montenegro	1.45	1.71	1.24	2.08	2.50	1.70	0.52	0.61	0.45	0.46	0.56	0.38	-0.62	-0.73	-0.52

© 2020 Deng Y et al. *JAMA Network Open.*

Sex	Location	Deaths						Age-standardized death rate (per 100,000)						EAPC		
		1990(95%UI)			2017(95%UI)			1990(95%UI)			2017(95%UI)			1990-2017(95%CI)		
		Cases (No.)	UL	LL	Cases (No.)	UL	LL	95%UI	UL	LL	95%UI	UL	LL	EAPC	LL	UL
Male	Morocco	35.95	43.15	29.43	84.23	109.28	63.67	0.46	0.56	0.38	0.54	0.70	0.41	0.58	0.45	0.72
Male	Mozambique	15.62	20.50	11.60	39.25	54.31	26.63	0.49	0.63	0.37	0.68	0.91	0.48	1.63	1.46	1.80
Male	Myanmar	72.36	109.05	46.38	119.11	146.53	97.32	0.64	0.95	0.43	0.63	0.77	0.52	-0.07	-0.12	-0.02
Male	Namibia	1.13	1.39	0.91	2.35	2.86	1.93	0.31	0.38	0.26	0.36	0.44	0.31	0.61	0.42	0.80
Male	Nepal	20.11	27.28	14.11	48.51	64.01	35.84	0.39	0.52	0.27	0.48	0.63	0.36	0.93	0.77	1.10
Male	Netherlands	31.26	33.83	28.84	58.39	66.56	51.37	0.37	0.40	0.34	0.39	0.45	0.35	0.07	-0.21	0.36
Male	New Zealand	5.82	6.49	5.23	12.34	14.01	10.72	0.33	0.37	0.30	0.37	0.41	0.32	0.43	0.23	0.63
Male	Nicaragua	1.94	2.21	1.67	7.19	8.87	5.86	0.25	0.29	0.21	0.34	0.42	0.28	1.58	1.19	1.98
Male	Niger	3.86	4.98	2.62	7.86	10.46	5.71	0.26	0.33	0.18	0.23	0.30	0.17	-0.65	-0.77	-0.53
Male	Nigeria	43.81	62.75	29.71	75.29	104.77	55.35	0.20	0.28	0.14	0.20	0.27	0.15	-0.17	-0.29	-0.05
Male	North Korea	22.33	29.03	16.25	52.26	68.81	40.65	0.38	0.48	0.28	0.45	0.57	0.36	0.79	0.55	1.03
Male	Northern Mariana Islands	0.05	0.06	0.04	0.10	0.13	0.08	0.43	0.55	0.35	0.46	0.55	0.38	0.08	-0.39	0.55
Male	Norway	11.93	12.41	11.51	15.95	17.49	14.64	0.41	0.42	0.40	0.38	0.41	0.35	-0.04	-0.19	0.12
Male	Oman	1.40	1.92	1.03	4.93	6.45	3.54	0.34	0.45	0.25	0.39	0.51	0.28	0.98	0.63	1.33
Male	Pakistan	197.17	238.32	161.39	476.71	626.69	341.62	0.57	0.69	0.47	0.75	0.98	0.54	1.09	0.97	1.21
Male	Palestine	1.28	1.77	0.95	3.33	3.94	2.78	0.30	0.42	0.22	0.28	0.34	0.24	-0.36	-0.57	-0.14
Male	Panama	2.56	2.83	2.32	7.71	8.74	6.73	0.33	0.36	0.30	0.41	0.46	0.35	1.46	1.17	1.75
Male	Papua New Guinea	4.56	8.33	3.29	12.69	22.44	9.11	0.44	0.76	0.33	0.52	0.86	0.37	0.71	0.58	0.84
Male	Paraguay	4.83	5.62	4.02	16.11	20.42	12.26	0.46	0.54	0.39	0.65	0.82	0.50	1.36	0.92	1.81
Male	Peru	27.53	31.86	23.24	80.27	100.24	62.10	0.46	0.53	0.38	0.54	0.67	0.42	0.80	0.62	0.99
Male	Philippines	107.14	121.49	95.10	329.18	400.87	267.91	0.71	0.80	0.63	1.01	1.22	0.83	1.48	1.22	1.74
Male	Poland	146.50	156.32	137.31	102.32	116.72	89.75	0.78	0.83	0.73	0.36	0.41	0.32	-3.58	-4.24	-2.92

© 2020 Deng Y et al. *JAMA Network Open.*

Sex	Location	Deaths						Age-standardized death rate (per 100,000)						EAPC		
		1990(95%UI)			2017(95%UI)			1990(95%UI)			2017(95%UI)			1990-2017(95%CI)		
		Cases (No.)	UL	LL	Cases (No.)	UL	LL	95%UI	UL	LL	95%UI	UL	LL	EAPC	LL	UL
Male	Portugal	29.94	32.67	27.15	41.66	47.94	35.84	0.52	0.56	0.47	0.42	0.48	0.36	-0.76	-0.98	-0.55
Male	Puerto Rico	8.69	9.59	7.87	12.42	14.21	10.84	0.51	0.56	0.46	0.42	0.47	0.37	-0.52	-0.85	-0.20
Male	Qatar	0.31	0.41	0.24	1.96	2.55	1.48	0.47	0.60	0.37	0.30	0.39	0.23	-2.03	-2.75	-1.31
Male	Republic of Congo	1.73	2.35	1.33	3.69	5.08	2.60	0.35	0.46	0.28	0.32	0.44	0.23	-0.44	-0.55	-0.33
Male	Romania	54.80	59.75	50.06	58.03	65.50	51.51	0.42	0.46	0.39	0.38	0.42	0.34	-1.00	-1.38	-0.62
Male	Russia	253.75	276.91	239.56	326.53	339.68	312.29	0.41	0.44	0.39	0.38	0.40	0.36	-0.74	-1.21	-0.27
Male	Rwanda	7.22	10.31	4.94	13.08	17.94	9.54	0.48	0.68	0.34	0.51	0.69	0.37	0.10	0.03	0.18
Male	Saint Lucia	0.13	0.15	0.12	0.33	0.37	0.29	0.34	0.37	0.30	0.33	0.37	0.29	-0.03	-0.20	0.14
Male	Saint Vincent	0.12	0.13	0.10	0.35	0.40	0.30	0.35	0.40	0.31	0.51	0.58	0.44	1.44	1.30	1.58
Male	Samoa	0.25	0.31	0.19	0.34	0.44	0.24	0.56	0.69	0.44	0.53	0.65	0.39	-0.29	-0.38	-0.20
Male	Sao Tome and Principe	0.06	0.08	0.05	0.13	0.17	0.09	0.21	0.26	0.16	0.27	0.35	0.20	1.05	1.00	1.09
Male	Saudi Arabia	17.33	22.43	13.25	77.92	98.94	58.69	0.45	0.58	0.34	0.79	0.95	0.63	3.14	2.70	3.59
Male	Senegal	4.83	5.80	3.98	10.36	13.43	7.96	0.29	0.35	0.24	0.31	0.40	0.24	0.21	0.10	0.33
Male	Serbia	20.36	24.58	16.49	28.54	33.55	24.09	0.39	0.47	0.32	0.41	0.47	0.34	0.62	0.47	0.76
Male	Seychelles	0.11	0.13	0.09	0.13	0.15	0.11	0.46	0.53	0.39	0.28	0.32	0.24	-2.02	-2.30	-1.74
Male	Sierra Leone	2.38	3.07	1.63	3.86	4.93	2.99	0.23	0.30	0.16	0.23	0.29	0.18	-0.07	-0.13	-0.01
Male	Singapore	4.27	4.72	3.90	8.14	9.58	6.96	0.41	0.46	0.38	0.27	0.32	0.23	-1.25	-1.45	-1.05
Male	Slovakia	12.42	14.03	10.88	12.47	14.76	10.30	0.48	0.54	0.43	0.34	0.40	0.28	-1.44	-1.58	-1.29
Male	Slovenia	4.58	5.08	4.15	7.10	8.18	6.12	0.47	0.52	0.43	0.40	0.46	0.35	-0.59	-0.73	-0.45
Male	Solomon Islands	0.36	0.56	0.26	0.84	1.18	0.63	0.44	0.67	0.31	0.50	0.70	0.38	0.60	0.46	0.75
Male	Somalia	6.39	11.09	3.00	21.73	30.17	14.78	0.44	0.71	0.25	0.62	0.83	0.44	1.20	1.10	1.30
Male	South Africa	23.09	27.65	19.41	46.84	52.54	40.40	0.25	0.30	0.20	0.27	0.30	0.23	0.14	-0.31	0.58

© 2020 Deng Y et al. *JAMA Network Open.*

Sex	Location	Deaths						Age-standardized death rate (per 100,000)						EAPC		
		1990(95%UI)			2017(95%UI)			1990(95%UI)			2017(95%UI)			1990-2017(95%CI)		
		Cases (No.)	UL	LL	Cases (No.)	UL	LL	95%UI	UL	LL	95%UI	UL	LL	EAPC	LL	UL
Male	South Korea	42.45	46.49	38.09	284.03	337.07	241.95	0.38	0.42	0.34	0.77	0.90	0.66	4.10	3.21	4.99
Male	South Sudan	6.27	10.78	3.31	12.97	19.79	8.84	0.43	0.69	0.26	0.59	0.87	0.41	1.14	1.02	1.26
Male	Spain	94.72	100.84	88.62	167.24	189.94	146.38	0.40	0.43	0.38	0.40	0.45	0.35	0.09	0.03	0.16
Male	Sri Lanka	32.26	37.74	27.75	50.16	65.97	37.33	0.60	0.69	0.51	0.47	0.61	0.35	-1.37	-1.99	-0.75
Male	Sudan	16.79	26.39	11.03	35.78	48.27	25.64	0.34	0.51	0.22	0.37	0.50	0.26	0.53	0.43	0.62
Male	Suriname	0.29	0.33	0.26	0.92	1.11	0.77	0.24	0.27	0.21	0.35	0.41	0.29	1.56	1.34	1.78
Male	Swaziland	0.54	0.67	0.43	1.24	1.73	0.85	0.41	0.51	0.33	0.51	0.71	0.36	1.27	0.95	1.59
Male	Sweden	24.63	26.83	22.66	32.02	35.81	28.50	0.37	0.40	0.34	0.34	0.38	0.30	0.11	-0.12	0.33
Male	Switzerland	17.92	20.21	15.85	31.75	37.96	26.91	0.41	0.45	0.36	0.41	0.49	0.35	0.51	0.32	0.69
Male	Syria	5.38	7.22	4.25	13.68	17.68	10.42	0.19	0.26	0.15	0.22	0.28	0.16	0.06	-0.11	0.23
Male	Tajikistan	2.15	2.53	1.80	4.42	5.34	3.70	0.18	0.21	0.15	0.19	0.23	0.16	-0.07	-0.37	0.24
Male	Tanzania	22.22	30.15	14.62	60.72	82.08	44.53	0.38	0.50	0.27	0.49	0.66	0.36	0.90	0.78	1.01
Male	Thailand	88.74	103.19	76.60	169.13	202.12	141.75	0.55	0.66	0.47	0.40	0.48	0.34	-1.74	-1.94	-1.54
Male	Timor-Leste	0.69	0.91	0.45	2.27	3.31	1.47	0.46	0.61	0.31	0.59	0.85	0.39	1.04	0.91	1.17
Male	Tobago	1.66	1.83	1.50	3.32	4.19	2.55	0.42	0.46	0.38	0.40	0.50	0.31	-0.47	-0.92	-0.02
Male	Togo	1.56	1.96	1.20	3.73	4.78	2.81	0.25	0.32	0.20	0.26	0.33	0.20	0.05	-0.06	0.16
Male	Tonga	0.11	0.14	0.09	0.21	0.28	0.16	0.43	0.53	0.35	0.58	0.76	0.43	1.16	1.01	1.32
Male	Trinidad	1.66	1.83	1.50	3.32	4.19	2.55	0.42	0.46	0.38	0.40	0.50	0.31	-0.47	-0.92	-0.02
Male	Tunisia	8.96	10.68	7.20	20.85	27.52	15.46	0.34	0.41	0.27	0.37	0.48	0.27	0.10	-0.02	0.22
Male	Turkey	74.77	109.23	54.97	162.42	190.10	136.74	0.43	0.62	0.32	0.42	0.49	0.35	0.08	-0.21	0.37
Male	Turkmenistan	3.22	3.65	2.82	5.39	6.32	4.57	0.36	0.40	0.31	0.30	0.34	0.25	-0.74	-1.03	-0.46
Male	Uganda	11.62	15.27	8.57	54.07	69.02	41.27	0.32	0.42	0.25	0.77	0.98	0.59	3.72	3.42	4.02
Male	UK	130.14	139.70	121.63	170.83	178.36	163.69	0.35	0.37	0.33	0.30	0.32	0.29	-0.35	-0.47	-0.23

© 2020 Deng Y et al. *JAMA Network Open.*

Sex	Location	Deaths						Age-standardized death rate (per 100,000)						EAPC		
		1990(95%UI)			2017(95%UI)			1990(95%UI)			2017(95%UI)			1990-2017(95%CI)		
		Cases (No.)	UL	LL	Cases (No.)	UL	LL	95%UI	UL	LL	95%UI	UL	LL	EAPC	LL	UL
Male	Ukraine	102.03	134.12	83.13	140.42	159.76	124.61	0.38	0.50	0.32	0.49	0.56	0.44	0.49	0.12	0.87
Male	United Arab Emirates	1.58	2.12	1.14	17.18	27.74	10.26	0.38	0.53	0.25	0.46	0.78	0.25	0.85	0.72	0.97
Male	Uruguay	5.11	5.81	4.54	10.48	12.31	8.84	0.30	0.34	0.27	0.48	0.56	0.40	1.43	0.95	1.91
Male	USA	472.33	488.07	459.27	1005.64	1050.23	955.34	0.35	0.36	0.34	0.41	0.43	0.39	0.49	0.37	0.61
Male	Uzbekistan	7.01	9.67	5.71	19.50	22.86	16.40	0.15	0.21	0.12	0.25	0.29	0.21	2.00	1.90	2.11
Male	Vanuatu	0.22	0.35	0.13	0.69	1.12	0.38	0.57	0.87	0.34	0.78	1.26	0.44	1.32	1.18	1.45
Male	Venezuela	14.69	16.34	13.32	55.49	68.54	43.42	0.32	0.36	0.29	0.44	0.54	0.34	0.81	0.56	1.05
Male	Vietnam	122.71	148.54	100.72	393.30	483.72	320.82	0.68	0.81	0.56	1.00	1.22	0.83	2.16	1.83	2.49
Male	Virgin Islands	0.17	0.20	0.15	0.48	0.58	0.37	0.44	0.50	0.38	0.60	0.71	0.47	1.62	1.47	1.76
Male	Yemen	8.35	15.75	4.70	24.36	33.78	17.63	0.32	0.57	0.20	0.38	0.52	0.28	0.72	0.66	0.78
Male	Zambia	8.09	10.77	5.74	19.25	24.82	15.28	0.47	0.62	0.36	0.54	0.70	0.43	0.33	0.19	0.47
Male	Zimbabwe	9.81	11.83	7.95	21.47	26.31	17.36	0.48	0.57	0.40	0.66	0.80	0.54	1.16	0.50	1.83

Abbreviations: CI, confidence interval; EAPC, estimated annual percentage change; UI, uncertainty interval; LL, lower limit; UL, upper limit.

© 2020 Deng Y et al. *JAMA Network Open.*

eTable5. The DALYs of thyroid cancer and its temporal trends from 1990 to 2017 among 195 countries.

Sex	Location	Disability adjusted life-years						Age-standardized DALY rate (per 100,000)						EAPC		
		1990(95%UI)			2017(95%UI)			1990(95%UI)			2017(95%UI)			1990-2017(95%CI)		
		DALYs (No.)	UL	LL	DALYs (No.)	UL	LL	95%UI	UL	LL	95%UI	UL	LL	EAPC	LL	UL
Both	Afghanistan	1680.36	2772.52	706.60	3340.83	4941.95	1849.81	22.37	36.95	9.56	21.99	30.96	13.37	0.01	-0.11	0.14
Both	Albania	347.71	383.41	298.89	444.32	544.28	353.87	13.93	15.43	12.01	11.71	14.41	9.32	-0.85	-1.10	-0.61
Both	Algeria	2626.80	3061.55	2259.13	6644.42	7688.35	5728.44	15.34	19.31	13.26	17.52	20.32	15.18	0.83	0.66	0.99
Both	American Samoa	5.02	6.02	4.06	9.01	10.45	7.85	20.13	24.26	15.79	19.83	22.79	17.27	0.02	-0.41	0.46
Both	Andorra	6.39	8.28	4.93	11.60	15.22	9.03	10.50	13.63	8.16	9.22	12.11	7.18	-0.59	-0.66	-0.52
Both	Angola	631.72	874.67	414.80	1353.09	1841.77	1068.80	12.15	16.45	8.58	10.08	13.76	7.99	-0.77	-0.83	-0.71
Both	Antigua	6.61	7.35	5.94	15.14	16.98	13.43	12.80	14.24	11.55	14.61	16.31	13.02	0.42	0.13	0.70
Both	Argentina	5855.30	6168.44	5537.33	6172.35	7004.42	5430.54	17.64	18.57	16.68	12.12	13.77	10.65	-1.56	-1.85	-1.27
Both	Armenia	153.13	177.15	132.92	553.38	608.80	502.25	5.26	6.05	4.57	13.53	14.80	12.27	3.74	2.76	4.73
Both	Australia	1846.22	1990.32	1710.93	4068.21	4706.80	3495.32	9.47	10.23	8.77	11.57	13.56	9.93	1.22	1.03	1.40
Both	Austria	2088.86	2253.72	1938.65	1661.12	1862.29	1464.77	18.43	19.98	17.05	10.61	11.99	9.32	-2.53	-2.73	-2.32
Both	Azerbaijan	639.46	751.59	558.91	1147.87	1410.48	961.21	11.06	13.39	9.56	11.03	13.87	9.28	-0.51	-0.69	-0.33
Both	Bahamas	29.41	31.97	26.85	68.71	78.42	60.63	16.09	17.55	14.69	16.82	19.14	14.85	0.38	0.18	0.58
Both	Bahrain	27.31	31.95	24.13	92.83	108.28	77.88	12.71	14.55	10.75	9.29	10.97	6.83	-1.27	-1.66	-0.88
Both	Bangladesh	12496.30	18227.90	9174.81	18891.36	24957.53	14749.06	17.49	25.19	13.32	13.10	16.82	10.45	-0.83	-0.96	-0.71
Both	Barbados	40.20	43.60	37.08	65.67	73.84	57.97	14.62	15.92	13.48	14.82	16.63	13.06	0.12	0.01	0.24
Both	Barbuda	6.61	7.35	5.94	15.14	16.98	13.43	12.80	14.24	11.55	14.61	16.31	13.02	0.42	0.13	0.70
Both	Belarus	1628.69	1789.78	1499.60	2098.53	2442.82	1838.65	13.08	14.40	12.03	14.03	16.37	12.32	0.08	-0.37	0.54
Both	Belgium	2816.47	3027.56	2614.86	2157.11	2442.85	1904.54	18.94	20.42	17.50	11.29	13.04	9.92	-1.69	-2.07	-1.32
Both	Belize	7.44	8.24	6.75	29.77	33.05	26.87	7.16	7.93	6.48	9.81	10.87	8.89	1.24	1.02	1.46
Both	Benin	216.67	263.38	160.22	406.34	510.00	312.88	8.63	10.37	6.60	7.29	9.21	5.63	-0.73	-0.81	-0.65
Both	Bermuda	13.98	15.21	12.79	16.92	19.06	14.91	21.38	23.25	19.57	14.80	16.80	13.10	-1.35	-1.53	-1.16

© 2020 Deng Y et al. *JAMA Network Open.*

Sex	Location	Disability adjusted life-years						Age-standardized DALY rate (per 100,000)						EAPC		
		1990(95%UI)			2017(95%UI)			1990(95%UI)			2017(95%UI)			1990-2017(95%CI)		
		DALYs (No.)	UL	LL	DALYs (No.)	UL	LL	95%UI	UL	LL	95%UI	UL	LL	EAPC	LL	UL
Both	Bhutan	69.67	102.87	50.05	115.66	163.40	82.88	18.50	27.54	13.78	14.80	20.10	11.03	-0.95	-1.00	-0.90
Both	Bolivia	1212.18	1591.64	800.13	2766.96	3528.11	2101.22	32.14	41.93	21.56	30.40	38.53	23.04	-0.27	-0.36	-0.19
Both	Bosnia and Herzegovina	549.98	620.99	454.00	646.84	738.88	512.86	12.16	13.63	10.20	11.44	13.07	9.06	-0.48	-0.63	-0.34
Both	Botswana	68.80	91.40	52.00	144.99	185.31	116.58	9.93	12.92	7.64	9.34	11.92	7.59	0.44	-0.01	0.90
Both	Brazil	14488.46	15112.46	13955.80	27529.24	28911.12	26187.71	14.27	14.84	13.76	11.90	12.48	11.33	-0.69	-0.78	-0.60
Both	Brunei	26.12	31.63	21.64	76.14	87.99	65.54	22.07	26.73	18.32	21.81	25.04	18.83	0.48	0.21	0.76
Both	Bulgaria	1698.24	1828.39	1564.94	1375.78	1536.29	1240.06	13.90	14.99	12.85	11.50	12.86	10.37	-1.00	-1.17	-0.84
Both	Burkina Faso	559.63	736.08	380.10	772.55	921.49	638.62	10.39	13.69	7.20	7.31	8.69	6.04	-1.59	-1.80	-1.37
Both	Burundi	784.11	1013.68	534.50	1097.59	1374.27	867.89	23.80	30.62	16.69	17.61	21.76	13.96	-1.35	-1.48	-1.22
Both	Cambodia	1579.91	2107.58	986.29	2538.54	3189.74	2074.80	28.73	38.14	18.59	20.61	25.70	16.93	-1.42	-1.51	-1.33
Both	Cameroon	637.70	763.93	468.21	1177.10	1496.26	920.91	11.14	13.30	8.42	8.50	10.78	6.64	-1.12	-1.25	-0.99
Both	Canada	3342.97	3637.87	3105.85	5818.99	6578.40	5140.71	10.40	11.31	9.66	10.11	11.47	8.92	-0.15	-0.36	0.06
Both	Cape Verde	10.84	13.75	7.80	23.42	28.31	17.46	4.74	6.08	3.28	5.00	6.06	3.71	0.08	0.00	0.16
Both	Central African Republic	202.11	273.18	137.25	294.12	421.49	195.95	13.89	18.40	10.09	11.16	15.86	7.81	-0.86	-0.93	-0.80
Both	Chad	271.59	338.69	187.91	450.98	542.54	366.63	8.01	10.00	5.60	6.88	8.24	5.56	-0.62	-0.72	-0.52
Both	Chile	1795.10	1944.62	1651.16	3277.36	3757.68	2841.39	16.65	18.00	15.29	14.44	16.57	12.48	-0.53	-0.65	-0.40
Both	China	97404.96	110236.17	89652.29	172611.84	190843.93	159229.50	9.78	11.15	9.04	9.02	9.98	8.31	-0.26	-0.59	0.07
Both	Colombia	4616.40	4924.64	4353.13	8443.43	9782.30	7282.35	22.63	24.09	21.36	15.67	18.15	13.52	-1.47	-1.77	-1.17
Both	Comoros	57.19	76.36	42.69	96.03	120.88	76.83	18.91	25.10	14.22	17.03	21.36	13.68	-0.32	-0.45	-0.19
Both	Costa Rica	361.17	389.77	336.12	769.12	868.54	684.45	17.45	18.88	16.30	15.38	17.38	13.67	-0.93	-1.23	-0.64
Both	Croatia	1274.48	1389.24	1169.82	832.27	930.04	744.31	19.58	21.27	18.04	10.67	12.01	9.55	-1.70	-1.98	-1.41
Both	Cuba	1210.65	1303.46	1124.49	2404.51	2742.31	2089.52	11.39	12.27	10.57	13.77	15.69	11.99	0.78	0.59	0.97

© 2020 Deng Y et al. *JAMA Network Open.*

Sex	Location	Disability adjusted life-years						Age-standardized DALY rate (per 100,000)						EAPC		
		1990(95%UI)			2017(95%UI)			1990(95%UI)			2017(95%UI)			1990-2017(95%CI)		
		DALYs (No.)	UL	LL	DALYs (No.)	UL	LL	95%UI	UL	LL	95%UI	UL	LL	EAPC	LL	UL
Both	Cyprus	136.54	159.23	103.22	191.76	225.91	162.81	16.07	18.80	12.18	10.70	12.57	9.06	-1.77	-2.08	-1.47
Both	Czech Republic	2790.17	2975.95	2617.36	1868.98	2115.52	1656.87	20.66	22.01	19.35	10.16	11.55	8.99	-2.57	-2.71	-2.44
Both	Democratic Republic of the Congo	2001.17	2610.01	1531.17	3576.03	4894.63	2634.01	10.21	13.10	7.97	8.81	12.17	6.30	-0.66	-0.76	-0.55
Both	Denmark	641.11	699.68	589.17	873.76	979.12	774.32	8.92	9.77	8.17	9.15	10.39	8.11	-0.06	-0.30	0.18
Both	Djibouti	46.02	64.96	30.56	140.25	203.40	95.27	16.59	23.00	11.53	17.57	25.27	11.97	0.17	0.13	0.22
Both	Dominica	7.98	8.65	7.37	12.80	14.11	11.54	11.68	12.68	10.73	14.87	16.38	13.44	0.96	0.69	1.23
Both	Dominican Republic	496.73	558.07	420.93	1132.56	1369.60	917.22	11.25	12.67	9.40	11.69	14.23	9.43	0.17	-0.40	0.73
Both	Ecuador	844.86	899.05	783.79	4273.38	4851.81	3735.14	13.66	14.46	12.68	28.36	32.13	24.80	3.26	2.71	3.81
Both	Egypt	3234.66	4910.25	2713.15	7265.39	10969.45	5569.75	8.68	13.71	7.25	9.63	15.35	7.35	0.58	0.44	0.71
Both	El Salvador	851.97	985.68	494.96	753.85	1053.10	592.19	26.32	30.42	15.40	13.30	18.58	10.44	-2.50	-3.37	-1.63
Both	Equatorial Guinea	33.73	49.02	21.26	60.64	97.22	37.23	14.31	20.27	9.53	10.31	16.36	6.36	-1.30	-1.54	-1.06
Both	Eritrea	350.76	489.81	209.89	897.90	1179.50	652.98	23.10	30.86	15.90	24.85	31.93	18.47	0.09	-0.13	0.31
Both	Estonia	298.33	325.74	274.91	310.08	368.27	256.68	15.24	16.63	14.05	13.34	15.97	11.02	-0.90	-1.21	-0.59
Both	Ethiopia	20394.19	30802.26	11387.83	24537.55	29920.01	19315.68	67.28	99.04	40.33	40.69	49.44	32.31	-2.14	-2.26	-2.02
Both	Fiji	158.73	196.23	102.51	238.96	297.22	153.06	35.36	44.35	22.28	29.94	36.52	19.18	-0.85	-0.99	-0.72
Both	Finland	983.44	1052.14	915.54	1187.35	1329.55	1057.30	14.14	15.14	13.15	11.62	13.05	10.29	-0.50	-0.67	-0.34
Both	France	12048.81	12758.77	11414.45	11096.22	12550.93	9831.51	15.53	16.49	14.68	10.12	11.57	8.93	-1.34	-2.11	-0.56
Both	Gabon	77.66	103.47	57.81	114.81	164.29	90.50	12.38	16.40	9.41	9.72	13.89	7.67	-0.86	-0.97	-0.74
Both	Gambia	36.71	44.84	30.05	92.71	118.54	74.66	7.60	9.17	6.32	8.00	10.22	6.48	0.35	0.28	0.42
Both	Georgia	523.09	585.72	443.86	785.18	870.50	697.55	8.25	9.24	7.07	14.58	16.16	12.98	2.55	1.63	3.48
Both	Germany	21754.35	22963.69	20560.10	20538.92	23351.35	18037.19	18.04	19.08	16.97	13.34	15.20	11.66	-1.44	-1.74	-1.13
Both	Ghana	324.81	408.14	257.50	717.57	847.65	602.23	4.38	5.49	3.46	4.24	5.07	3.50	-0.25	-0.38	-0.12

© 2020 Deng Y et al. *JAMA Network Open.*

Sex	Location	Disability adjusted life-years						Age-standardized DALY rate (per 100,000)						EAPC		
		1990(95%UI)			2017(95%UI)			1990(95%UI)			2017(95%UI)			1990-2017(95%CI)		
		DALYs (No.)	UL	LL	DALYs (No.)	UL	LL	95%UI	UL	LL	95%UI	UL	LL	EAPC	LL	UL
Both	Greece	1462.31	1572.95	1347.29	1798.18	2008.71	1606.90	9.85	10.61	9.04	9.11	10.26	8.10	-0.51	-0.68	-0.33
Both	Greenland	4.40	5.03	3.68	7.83	9.55	4.71	11.68	13.49	8.90	11.49	13.97	6.75	0.32	-0.18	0.82
Both	Grenada	20.30	21.89	18.69	28.31	31.30	25.70	29.78	32.15	27.42	20.51	22.58	18.53	-0.61	-1.07	-0.16
Both	Grenadines	14.33	15.76	13.08	28.76	32.17	25.87	18.85	20.79	17.18	21.52	24.03	19.38	0.50	0.17	0.83
Both	Guam	8.13	9.99	7.01	28.30	32.03	24.49	9.23	11.38	8.00	15.54	17.51	13.53	2.32	1.57	3.07
Both	Guatemala	641.24	690.73	595.89	1582.31	1807.25	1374.79	14.71	15.83	13.70	13.45	15.37	11.67	-1.94	-2.59	-1.29
Both	Guinea	451.07	513.18	390.76	784.90	949.40	654.35	11.04	12.64	9.65	11.25	14.06	9.40	0.12	0.05	0.19
Both	Guinea-Bissau	65.19	83.00	45.99	81.69	108.77	56.03	12.20	15.44	8.78	9.25	12.20	6.56	-1.10	-1.18	-1.02
Both	Guyana	59.59	64.39	55.20	106.16	121.54	92.58	12.90	13.92	11.92	15.53	17.73	13.62	1.02	0.80	1.23
Both	Haiti	837.11	1248.84	510.08	1473.63	2112.11	1046.45	21.60	32.01	13.59	18.90	26.76	13.68	-0.42	-0.51	-0.33
Both	Honduras	443.75	510.31	381.25	1153.18	1625.36	833.60	18.80	21.76	16.09	18.33	25.66	13.36	-0.08	-0.22	0.07
Both	Hungary	3142.69	3357.00	2931.63	1908.15	2133.69	1717.10	21.62	23.11	20.16	11.15	12.52	10.03	-2.55	-2.78	-2.32
Both	Iceland	93.35	102.28	85.63	130.10	146.80	116.11	33.74	36.95	30.85	27.25	30.81	24.20	-0.67	-0.75	-0.59
Both	India	94721.50	116164.43	74683.76	202323.54	220531.59	181444.20	14.50	17.64	11.81	16.38	17.78	14.71	0.45	0.34	0.55
Both	Indonesia	23314.12	27101.50	18026.72	37168.90	42682.17	31656.30	19.21	22.10	15.22	16.15	18.45	13.87	-0.67	-0.75	-0.60
Both	Iran	2517.21	2911.10	2182.78	8086.96	8945.04	6499.33	7.90	9.00	6.87	10.61	11.67	8.43	1.96	1.50	2.42
Both	Iraq	1720.64	2207.42	1269.86	3012.69	3517.34	2500.49	17.62	22.85	13.23	10.90	12.63	8.98	-1.98	-2.27	-1.68
Both	Ireland	523.46	566.93	482.81	601.50	696.49	523.31	12.92	13.96	11.90	8.96	10.40	7.78	-0.93	-1.21	-0.66
Both	Israel	709.94	767.77	655.29	1560.76	1782.06	1372.75	14.49	15.67	13.39	14.80	16.88	13.02	-0.19	-0.52	0.15
Both	Italy	14997.53	16329.79	13847.41	13978.31	15834.15	12268.63	18.06	19.75	16.58	12.11	14.01	10.49	-1.39	-1.53	-1.25
Both	Ivory Coast	577.72	714.41	450.30	1246.32	1616.48	963.89	8.90	10.58	7.47	8.62	10.86	6.72	-0.17	-0.33	-0.02
Both	Jamaica	167.75	185.86	153.26	516.18	618.87	429.11	9.12	10.12	8.31	17.75	21.30	14.76	2.61	2.29	2.93
Both	Japan	22384.74	23712.95	21255.54	32067.88	34786.77	29872.14	13.01	13.81	12.34	10.57	11.72	9.70	-0.77	-0.87	-0.67
Both	Jordan	379.37	466.85	292.77	780.73	981.89	635.73	18.78	23.10	14.73	11.42	14.36	9.34	-2.34	-2.77	-1.92

© 2020 Deng Y et al. *JAMA Network Open.*

Sex	Location	Disability adjusted life-years						Age-standardized DALY rate (per 100,000)						EAPC		
		1990(95%UI)			2017(95%UI)			1990(95%UI)			2017(95%UI)			1990-2017(95%CI)		
		DALYs (No.)	UL	LL	DALYs (No.)	UL	LL	95%UI	UL	LL	95%UI	UL	LL	EAPC	LL	UL
Both	Kazakhstan	3791.69	4542.10	3427.33	2487.14	2845.52	2224.00	26.00	31.05	23.57	13.57	15.46	12.18	-3.45	-3.86	-3.04
Both	Kenya	730.70	871.61	622.03	2010.69	2439.17	1683.14	6.21	7.33	5.30	6.77	8.23	5.66	0.38	0.25	0.51
Both	Kiribati	4.07	4.58	3.57	6.95	8.24	5.71	9.65	10.88	8.37	9.27	11.05	7.66	-0.02	-0.13	0.08
Both	Kuwait	139.46	153.14	126.16	275.41	322.66	238.69	14.70	16.00	13.47	9.16	10.58	8.04	-1.48	-1.89	-1.07
Both	Kyrgyzstan	511.91	564.73	457.85	470.32	535.48	422.40	15.16	16.77	13.61	9.54	10.74	8.57	-1.49	-1.65	-1.33
Both	Laos	731.30	1029.31	432.41	905.55	1155.62	686.68	29.14	40.47	17.92	18.90	23.97	14.64	-1.82	-1.92	-1.72
Both	Latvia	474.99	519.51	435.99	519.85	616.49	438.19	13.91	15.28	12.76	14.74	17.50	12.42	-0.03	-0.43	0.36
Both	Lebanon	559.17	689.19	430.92	1480.16	1871.52	1190.82	19.64	24.07	15.44	20.25	25.69	16.36	0.16	0.00	0.33
Both	Lesotho	122.33	152.06	98.92	185.57	259.49	131.33	11.06	13.74	8.94	14.02	19.52	9.91	1.59	1.23	1.95
Both	Liberia	117.77	145.74	84.20	162.01	204.41	128.33	9.12	11.28	6.58	6.82	8.56	5.42	-1.30	-1.53	-1.06
Both	Libya	334.12	409.42	269.35	959.45	1217.14	757.56	12.92	16.38	10.60	15.89	20.20	12.62	0.99	0.79	1.19
Both	Lithuania	636.12	698.33	582.98	638.18	716.31	569.99	14.32	15.71	13.10	12.89	14.57	11.44	-1.05	-1.54	-0.54
Both	Luxembourg	126.80	138.12	116.45	142.32	170.66	120.97	23.81	25.99	21.86	15.94	19.13	13.55	-1.76	-1.87	-1.64
Both	Macedonia	258.31	294.75	196.34	293.09	344.54	243.89	12.63	14.26	9.70	9.22	10.84	7.70	-1.99	-2.41	-1.57
Both	Madagascar	1350.46	1745.21	1035.94	2498.28	3151.29	1964.92	16.87	21.61	13.23	15.66	19.59	12.55	-0.24	-0.39	-0.08
Both	Malawi	1012.31	1405.90	461.68	1724.63	2364.91	1234.90	16.59	21.84	9.03	15.79	20.98	11.70	-0.66	-0.93	-0.38
Both	Malaysia	2539.02	2894.14	2150.93	5755.95	7262.76	4770.95	22.54	25.66	19.56	20.86	26.24	17.43	-0.30	-0.36	-0.24
Both	Maldives	39.18	58.96	24.26	69.31	82.04	59.28	37.97	55.19	25.04	22.81	27.00	19.34	-2.34	-2.50	-2.17
Both	Mali	963.61	1116.63	812.10	1542.63	2106.50	1166.05	17.44	20.17	15.17	13.29	17.62	10.26	-1.18	-1.31	-1.05
Both	Malta	72.69	79.18	66.75	106.70	119.94	95.20	16.71	18.20	15.37	13.75	15.42	12.26	-0.69	-0.85	-0.52
Both	Marshall Islands	4.27	5.33	3.13	9.72	12.91	6.59	21.22	26.68	15.43	24.68	32.27	17.31	0.56	0.32	0.79
Both	Mauritania	116.00	140.36	82.52	158.15	200.76	123.30	9.65	11.71	6.97	7.04	8.96	5.46	-1.27	-1.39	-1.15
Both	Mauritius	85.45	92.45	79.03	172.07	191.61	152.53	10.42	11.29	9.63	10.10	11.25	9.00	0.36	-0.08	0.80
Both	Mexico	7941.67	8218.20	7695.11	21520.06	22680.78	20397.51	16.22	16.79	15.72	18.18	19.13	17.25	0.35	0.24	0.46

© 2020 Deng Y et al. *JAMA Network Open.*

Sex	Location	Disability adjusted life-years						Age-standardized DALY rate (per 100,000)						EAPC		
		1990(95%UI)			2017(95%UI)			1990(95%UI)			2017(95%UI)			1990-2017(95%CI)		
		DALYs (No.)	UL	LL	DALYs (No.)	UL	LL	95%UI	UL	LL	95%UI	UL	LL	EAPC	LL	UL
Both	Micronesia	10.74	13.29	8.57	15.01	19.53	11.20	19.41	23.78	15.67	19.26	24.37	15.07	-0.05	-0.13	0.02
Both	Moldova	635.10	704.90	575.77	652.35	726.99	582.97	13.37	14.81	12.17	11.95	13.32	10.75	-0.28	-0.67	0.11
Both	Mongolia	137.25	157.21	105.40	310.09	375.29	220.83	11.76	13.56	8.71	12.57	15.47	8.00	0.23	-0.08	0.53
Both	Montenegro	101.28	117.79	85.79	137.01	159.08	117.10	15.48	18.01	13.13	14.47	16.86	12.39	-0.43	-0.61	-0.24
Both	Morocco	3621.27	4235.71	2939.79	6694.63	8353.65	5430.71	19.48	22.57	16.52	19.23	23.94	15.70	-0.04	-0.08	-0.01
Both	Mozambique	1740.88	2340.58	1293.26	3557.01	4623.66	2477.90	18.76	25.35	14.38	20.45	26.36	14.55	0.43	0.26	0.60
Both	Myanmar	9205.25	12839.02	5646.37	10733.68	13750.44	8302.07	33.34	46.06	21.04	22.31	28.48	17.47	-1.66	-1.79	-1.52
Both	Namibia	105.79	135.37	76.45	180.34	223.74	146.52	11.73	16.14	8.69	10.52	13.38	8.54	-0.63	-1.12	-0.13
Both	Nepal	2244.06	3276.11	1624.76	3913.14	5290.95	3028.83	17.02	24.78	12.72	15.69	20.97	12.35	-0.28	-0.54	-0.01
Both	Netherlands	2136.38	2293.30	1972.00	3183.70	3561.39	2804.96	11.01	11.88	10.14	10.78	12.15	9.46	-0.05	-0.23	0.13
Both	New Zealand	437.63	478.40	401.90	701.30	787.57	630.42	11.28	12.35	10.34	10.98	12.32	9.80	0.16	-0.05	0.36
Both	Nicaragua	183.26	243.15	159.85	615.61	715.98	524.33	9.58	12.93	8.34	12.62	14.66	10.73	1.47	1.09	1.85
Both	Niger	328.87	411.14	222.22	533.89	667.33	423.49	8.32	10.23	5.85	5.98	7.44	4.71	-1.54	-1.69	-1.38
Both	Nigeria	2672.84	3706.15	1745.76	4783.98	6765.37	3419.17	5.48	7.70	3.55	4.99	7.03	3.58	-0.56	-0.67	-0.45
Both	North Korea	2205.87	2772.83	1764.90	3678.15	4747.23	2897.19	11.74	14.91	9.41	11.62	15.02	9.19	-0.01	-0.09	0.07
Both	Northern Mariana Islands	2.99	3.58	2.46	6.14	7.13	5.22	12.28	14.49	10.36	11.45	13.15	9.90	-0.48	-0.84	-0.12
Both	Norway	746.75	789.72	710.00	923.23	1018.15	847.17	12.30	13.12	11.62	11.32	12.62	10.28	-0.15	-0.26	-0.05
Both	Oman	123.36	155.04	95.15	373.88	459.54	301.33	11.94	15.06	9.36	12.11	14.71	9.90	0.28	-0.01	0.56
Both	Pakistan	26448.27	33822.08	21646.12	72465.53	105335.50	51635.83	33.48	41.77	28.02	42.46	58.20	31.33	0.77	0.51	1.02
Both	Palestine	124.97	155.34	94.56	282.23	324.90	247.00	12.36	15.37	9.21	10.47	12.10	8.95	-0.43	-0.58	-0.28
Both	Panama	227.46	245.33	211.58	549.07	607.22	491.15	12.70	13.69	11.80	13.83	15.30	12.36	0.73	0.51	0.95
Both	Papua New Guinea	448.18	610.66	346.15	1106.51	1529.74	843.08	19.12	25.55	14.94	19.50	25.61	15.10	0.17	0.07	0.27
Both	Paraguay	344.46	392.85	292.04	1035.86	1275.36	803.62	14.11	16.13	11.83	18.58	22.88	14.37	1.16	0.87	1.45

© 2020 Deng Y et al. *JAMA Network Open.*

Sex	Location	Disability adjusted life-years						Age-standardized DALY rate (per 100,000)						EAPC		
		1990(95%UI)			2017(95%UI)			1990(95%UI)			2017(95%UI)			1990-2017(95%CI)		
		DALYs (No.)	UL	LL	DALYs (No.)	UL	LL	95%UI	UL	LL	95%UI	UL	LL	EAPC	LL	UL
Both	Peru	2435.60	2819.34	2133.04	5780.22	6949.04	4633.98	17.58	20.28	15.29	18.71	22.50	14.95	0.27	0.04	0.50
Both	Philippines	10437.79	11749.16	9424.29	30154.84	35550.75	25309.44	28.34	31.59	25.74	37.95	44.77	32.03	1.26	1.08	1.45
Both	Poland	10724.94	11288.19	10174.33	7602.20	8519.76	6824.01	23.80	25.04	22.60	12.18	13.78	10.93	-3.10	-3.53	-2.67
Both	Portugal	2296.30	2471.19	2138.94	2188.79	2445.67	1936.40	16.91	18.17	15.74	10.79	12.14	9.49	-1.83	-2.12	-1.55
Both	Puerto Rico	558.57	603.41	515.81	539.66	607.68	481.99	15.10	16.29	13.94	9.28	10.48	8.22	-1.77	-2.18	-1.36
Both	Qatar	35.17	45.36	18.62	116.16	145.41	90.90	23.11	29.09	11.37	8.21	10.11	6.65	-4.59	-5.02	-4.16
Both	Republic of Congo	189.65	266.34	130.95	344.11	513.67	256.72	14.54	20.21	10.49	11.42	16.72	8.68	-1.06	-1.21	-0.91
Both	Romania	4069.90	4338.55	3821.82	4009.20	4447.66	3621.75	14.37	15.30	13.50	12.53	13.97	11.30	-1.00	-1.30	-0.69
Both	Russia	23645.64	25990.69	22304.64	30060.84	32288.50	28112.59	12.96	14.26	12.21	14.12	15.27	13.16	0.04	-0.41	0.48
Both	Rwanda	972.80	1348.24	601.05	1244.21	1669.40	970.11	22.09	30.43	13.92	15.54	20.37	12.20	-1.74	-1.95	-1.53
Both	Saint Lucia	19.72	21.44	18.04	37.95	42.31	33.37	21.02	22.93	19.21	17.96	20.05	15.82	-0.70	-1.10	-0.29
Both	Saint Vincent	14.33	15.76	13.08	28.76	32.17	25.87	18.85	20.79	17.18	21.52	24.03	19.38	0.50	0.17	0.83
Both	Samoa	23.53	28.36	19.35	31.13	39.61	24.37	21.86	28.66	17.68	20.46	26.93	16.03	-0.34	-0.38	-0.30
Both	Sao Tome and Principe	5.66	8.30	4.08	9.32	11.61	7.35	7.15	10.21	5.20	7.49	9.25	5.96	-0.08	-0.23	0.08
Both	Saudi Arabia	1204.67	1470.72	983.80	5931.34	7492.21	4731.62	13.32	16.64	10.91	21.61	25.95	17.97	2.60	2.28	2.92
Both	Senegal	339.32	404.92	259.37	580.08	712.57	486.36	8.29	9.76	6.52	7.04	8.69	5.87	-0.77	-0.90	-0.64
Both	Serbia	1526.71	1845.55	1241.73	1830.65	2092.60	1529.66	13.03	15.65	10.71	12.66	14.52	10.59	0.16	0.03	0.30
Both	Seychelles	6.51	7.45	5.22	8.20	9.25	7.23	11.18	12.81	8.94	7.34	8.26	6.49	-1.57	-1.80	-1.34
Both	Sierra Leone	194.72	249.93	142.37	300.16	382.51	237.40	8.23	10.55	6.11	7.29	9.35	5.67	-0.46	-0.57	-0.36
Both	Singapore	331.67	360.42	305.46	550.30	638.73	474.99	12.74	13.76	11.80	7.90	9.15	6.85	-1.39	-1.53	-1.25
Both	Slovakia	1010.78	1113.47	894.38	918.30	1104.96	788.34	16.94	18.62	14.97	10.74	12.99	9.23	-1.84	-1.94	-1.74
Both	Slovenia	375.22	406.41	347.47	358.28	406.79	313.41	15.20	16.44	14.11	9.72	11.00	8.50	-1.82	-1.93	-1.71
Both	Solomon Islands	29.14	38.00	22.37	67.05	86.55	51.26	16.72	21.53	13.23	17.35	21.93	13.58	0.21	0.09	0.34

© 2020 Deng Y et al. *JAMA Network Open.*

Sex	Location	Disability adjusted life-years						Age-standardized DALY rate (per 100,000)						EAPC		
		1990(95%UI)			2017(95%UI)			1990(95%UI)			2017(95%UI)			1990-2017(95%CI)		
		DALYs (No.)	UL	LL	DALYs (No.)	UL	LL	95%UI	UL	LL	95%UI	UL	LL	EAPC	LL	UL
Both	Somalia	681.49	1104.54	284.87	1896.01	2559.44	1356.89	17.50	27.24	9.10	20.01	26.82	14.55	0.30	0.18	0.42
Both	South Africa	2284.55	2567.91	1996.43	3622.46	4194.58	3262.69	9.08	10.44	7.86	7.65	8.74	6.90	-0.87	-1.52	-0.23
Both	South Korea	3178.79	3365.31	2912.84	19579.69	22646.29	16933.31	9.43	9.96	8.64	25.38	29.80	21.78	5.49	4.18	6.82
Both	South Sudan	578.60	970.25	272.27	1038.93	1492.90	701.31	16.49	26.28	9.10	18.65	26.75	12.89	0.36	0.18	0.53
Both	Spain	6244.38	6624.60	5900.12	7979.65	8907.02	7103.30	12.00	12.74	11.30	9.88	11.11	8.73	-0.67	-0.75	-0.59
Both	Sri Lanka	2338.60	2633.95	1870.78	3489.44	4463.95	2722.96	19.19	21.57	15.45	13.66	17.38	10.65	-1.77	-2.24	-1.29
Both	Sudan	1408.69	1997.90	927.78	2544.76	3316.07	1883.66	12.14	16.82	8.45	11.33	14.65	8.54	-0.17	-0.22	-0.11
Both	Suriname	38.47	42.54	34.58	90.76	104.01	78.67	13.41	14.82	12.05	15.00	17.14	13.07	0.56	0.40	0.72
Both	Swaziland	49.55	62.44	40.31	90.30	126.10	64.07	13.52	17.41	10.97	13.57	18.99	9.56	0.49	0.02	0.97
Both	Sweden	1496.51	1591.19	1397.97	1634.28	1801.57	1480.29	10.88	11.62	10.15	9.48	10.54	8.51	-0.16	-0.33	0.02
Both	Switzerland	1305.81	1422.78	1198.94	1509.77	1733.08	1329.89	13.73	15.02	12.53	10.18	11.74	8.94	-0.90	-1.07	-0.72
Both	Syria	325.54	404.17	242.57	716.08	868.56	584.89	5.20	6.51	3.83	4.93	5.92	4.05	-0.50	-0.67	-0.32
Both	Tajikistan	127.36	148.38	110.90	251.06	286.06	219.16	4.06	4.86	3.42	4.19	4.76	3.65	-0.30	-0.56	-0.04
Both	Tanzania	2674.04	3582.73	1633.45	5586.35	7360.59	4315.63	17.02	22.52	11.56	16.67	21.60	13.09	-0.34	-0.54	-0.13
Both	Thailand	7523.15	10210.47	6585.66	11431.88	19379.23	9331.78	18.43	24.58	16.11	11.73	19.88	9.61	-2.19	-2.48	-1.89
Both	Timor-Leste	79.96	108.28	53.75	148.86	191.85	108.09	20.60	27.35	14.77	17.27	22.26	12.76	-0.75	-0.89	-0.61
Both	Tobago	133.27	143.84	123.53	227.76	281.98	183.82	14.46	15.60	13.39	12.71	15.70	10.27	-1.15	-1.63	-0.67
Both	Togo	154.99	198.13	111.72	274.61	338.35	221.69	8.66	10.91	6.44	6.55	7.97	5.31	-1.21	-1.31	-1.10
Both	Tonga	7.79	9.39	6.56	12.73	15.51	10.42	13.04	15.60	10.98	15.36	18.77	12.56	0.67	0.54	0.81
Both	Trinidad	133.27	143.84	123.53	227.76	281.98	183.82	14.46	15.60	13.39	12.71	15.70	10.27	-1.15	-1.63	-0.67
Both	Tunisia	689.23	796.01	601.61	1452.71	1890.60	1118.55	11.27	13.39	9.87	11.73	15.25	9.04	-0.01	-0.15	0.12
Both	Turkey	6065.21	8181.36	4338.73	9992.93	13290.41	8491.43	15.28	20.18	11.03	11.27	15.02	9.59	-1.20	-1.36	-1.04
Both	Turkmenistan	287.28	313.95	263.23	414.73	462.77	368.32	12.45	13.66	11.42	9.39	10.42	8.38	-1.05	-1.47	-0.64
Both	Uganda	1601.10	1976.25	1287.21	4480.45	5649.90	3534.29	16.09	19.61	13.11	20.86	25.65	16.71	1.01	0.86	1.16

© 2020 Deng Y et al. *JAMA Network Open.*

Sex	Location	Disability adjusted life-years						Age-standardized DALY rate (per 100,000)						EAPC		
		1990(95%UI)			2017(95%UI)			1990(95%UI)			2017(95%UI)			1990-2017(95%CI)		
		DALYs (No.)	UL	LL	DALYs (No.)	UL	LL	95%UI	UL	LL	95%UI	UL	LL	EAPC	LL	UL
Both	UK	8086.62	8509.45	7650.32	9193.72	9853.44	8611.02	9.64	10.20	9.12	8.62	9.31	8.01	-0.39	-0.48	-0.29
Both	Ukraine	8471.51	9923.98	7430.35	10444.53	11739.65	9438.05	11.94	13.94	10.52	15.50	17.55	13.95	0.60	0.21	0.99
Both	United Arab Emirates	120.21	153.27	92.53	1048.89	1530.55	704.42	14.04	20.12	9.53	14.71	22.20	9.54	0.39	0.25	0.52
Both	Uruguay	353.58	386.32	326.35	587.96	670.74	508.95	9.29	10.12	8.56	12.56	14.33	10.84	0.68	0.16	1.21
Both	USA	29690.08	31905.39	27782.15	55014.13	60244.76	50565.28	9.76	10.53	9.12	11.18	12.31	10.22	0.43	0.31	0.55
Both	Uzbekistan	456.18	607.63	401.14	1209.66	1382.10	1044.20	3.55	4.87	3.08	4.94	5.60	4.31	1.18	0.96	1.40
Both	Vanuatu	15.68	23.18	9.93	43.11	64.49	26.38	19.24	28.26	12.54	22.63	33.72	14.05	0.70	0.59	0.82
Both	Venezuela	1211.62	1302.87	1126.35	4158.05	4914.21	3466.89	11.01	11.83	10.23	14.17	16.71	11.80	0.80	0.64	0.95
Both	Vietnam	13044.01	16202.48	11135.69	31769.73	38693.02	26269.96	27.24	36.54	22.94	32.22	39.09	26.82	1.14	0.86	1.42
Both	Virgin Islands	9.63	10.78	8.61	20.86	24.25	17.58	10.10	11.21	9.06	12.01	13.95	10.20	1.02	0.88	1.16
Both	Yemen	778.47	1226.24	429.00	1927.91	2691.64	1333.78	11.97	18.36	7.31	11.90	16.38	8.62	-0.02	-0.05	0.01
Both	Zambia	1055.11	1435.89	701.33	1810.28	2305.76	1455.18	22.50	29.60	16.35	17.99	22.76	14.52	-1.27	-1.54	-1.01
Both	Zimbabwe	873.84	1009.91	711.62	2100.03	2574.88	1578.84	16.56	19.01	13.89	23.39	28.31	18.26	1.90	1.10	2.70
Female	Afghanistan	1143.17	2177.59	410.77	2399.65	4077.16	1111.33	32.36	61.73	12.28	30.51	49.80	15.66	-0.16	-0.25	-0.06
Female	Albania	168.76	196.14	115.71	199.13	270.40	140.05	13.88	16.21	9.43	9.97	13.57	6.98	-1.41	-1.63	-1.19
Female	Algeria	1675.49	2082.28	1401.71	3980.98	4883.24	3220.50	19.42	26.78	16.13	21.05	25.96	17.31	0.72	0.54	0.90
Female	American Samoa	3.77	4.70	2.94	6.79	8.19	5.72	31.61	39.23	24.06	29.29	34.86	24.89	-0.16	-0.60	0.29
Female	Andorra	3.56	5.20	2.41	6.32	9.86	4.40	12.24	17.85	8.32	10.17	15.85	7.05	-0.96	-1.15	-0.77
Female	Angola	404.27	654.50	233.84	821.74	1334.78	577.22	15.69	24.64	9.99	11.40	18.58	8.04	-1.25	-1.33	-1.17
Female	Antigua	5.39	6.09	4.73	12.43	14.22	10.83	19.25	21.84	16.92	22.72	25.84	19.82	0.50	0.11	0.89
Female	Argentina	3581.76	3837.74	3322.07	3602.21	4289.44	3002.23	19.91	21.35	18.45	12.97	15.49	10.79	-1.78	-2.13	-1.44
Female	Armenia	88.09	101.94	75.46	322.24	365.02	283.46	5.46	6.32	4.72	13.80	15.63	12.16	3.87	2.90	4.84
Female	Australia	1065.83	1169.49	972.76	2253.12	2713.73	1809.46	10.33	11.40	9.43	12.41	15.01	9.79	1.17	0.96	1.37

© 2020 Deng Y et al. *JAMA Network Open.*

Sex	Location	Disability adjusted life-years						Age-standardized DALY rate (per 100,000)						EAPC		
		1990(95%UI)			2017(95%UI)			1990(95%UI)			2017(95%UI)			1990-2017(95%CI)		
		DALYs (No.)	UL	LL	DALYs (No.)	UL	LL	95%UI	UL	LL	95%UI	UL	LL	EAPC	LL	UL
Female	Austria	1346.00	1474.14	1229.73	870.94	1017.95	743.41	20.08	22.22	18.13	10.39	12.28	8.84	-2.74	-2.93	-2.55
Female	Azerbaijan	395.24	504.16	327.09	743.22	980.59	579.59	12.40	16.18	10.18	13.27	17.75	10.38	-0.04	-0.20	0.13
Female	Bahamas	17.45	19.44	15.55	37.93	44.94	31.93	17.71	19.69	15.76	17.56	20.79	14.80	0.14	-0.14	0.43
Female	Bahrain	16.77	20.86	14.12	53.85	65.23	40.10	18.67	22.55	14.85	14.60	18.06	9.22	-0.81	-1.31	-0.32
Female	Bangladesh	8466.76	14351.53	5510.37	12284.42	18476.05	9019.04	23.92	41.34	16.46	16.81	24.73	12.61	-0.91	-1.08	-0.75
Female	Barbados	28.10	31.04	25.20	42.65	49.08	36.08	18.14	20.11	16.19	17.86	20.68	15.08	-0.03	-0.28	0.21
Female	Barbuda	5.39	6.09	4.73	12.43	14.22	10.83	19.25	21.84	16.92	22.72	25.84	19.82	0.50	0.11	0.89
Female	Belarus	1088.76	1226.21	975.82	1320.87	1569.93	1112.06	14.99	16.92	13.39	14.78	17.85	12.44	-0.28	-0.72	0.17
Female	Belgium	1825.36	1983.05	1659.37	991.56	1164.87	853.63	21.09	23.06	19.19	9.50	11.33	8.07	-2.89	-3.39	-2.38
Female	Belize	4.98	5.60	4.38	16.77	19.69	14.47	9.83	11.07	8.63	11.34	13.11	9.82	0.73	0.61	0.86
Female	Benin	135.57	179.37	85.49	225.38	294.85	161.86	9.99	13.15	6.57	7.52	9.97	5.32	-1.22	-1.34	-1.10
Female	Bermuda	7.95	8.90	7.09	7.51	9.07	6.21	22.17	24.82	19.80	11.88	14.38	9.80	-2.67	-2.89	-2.46
Female	Bhutan	48.33	81.21	29.25	67.05	112.48	41.81	25.57	43.66	16.15	17.53	29.16	11.51	-1.66	-1.78	-1.55
Female	Bolivia	903.20	1258.30	533.06	1939.64	2618.06	1355.23	45.82	63.77	27.52	41.00	55.37	28.63	-0.54	-0.62	-0.46
Female	Bosnia and Herzegovina	347.46	404.16	257.78	437.28	520.49	309.24	14.04	16.20	10.61	14.63	17.40	10.32	0.05	-0.11	0.22
Female	Botswana	41.23	62.30	28.38	96.92	138.91	72.41	11.19	16.61	7.80	11.40	16.24	8.56	1.16	0.47	1.84
Female	Brazil	8781.61	9239.62	8370.97	15240.51	16171.99	14314.95	16.70	17.52	15.94	12.22	12.96	11.48	-1.23	-1.33	-1.13
Female	Brunei	17.52	22.27	13.56	50.71	61.09	40.74	32.06	41.33	25.25	28.81	34.16	23.53	0.17	-0.17	0.51
Female	Bulgaria	962.41	1063.82	863.62	790.10	911.10	681.48	15.09	16.66	13.62	12.41	14.40	10.67	-0.99	-1.16	-0.81
Female	Burkina Faso	405.79	561.81	241.65	488.34	619.10	375.10	13.85	19.24	8.52	8.46	10.74	6.36	-2.17	-2.39	-1.95
Female	Burundi	565.33	793.40	364.85	668.42	917.13	471.44	31.37	43.77	21.00	21.42	29.51	14.80	-1.74	-1.90	-1.59
Female	Cambodia	1171.00	1694.41	658.90	1736.20	2407.41	1310.62	37.40	53.33	22.14	24.68	34.09	18.65	-1.79	-1.90	-1.67
Female	Cameroon	427.93	545.27	249.55	655.95	919.64	456.19	14.23	18.19	8.66	9.08	12.87	6.10	-1.76	-1.89	-1.64

© 2020 Deng Y et al. *JAMA Network Open.*

Sex	Location	Disability adjusted life-years						Age-standardized DALY rate (per 100,000)						EAPC		
		1990(95%UI)			2017(95%UI)			1990(95%UI)			2017(95%UI)			1990-2017(95%CI)		
		DALYs (No.)	UL	LL	DALYs (No.)	UL	LL	95%UI	UL	LL	95%UI	UL	LL	EAPC	LL	UL
Female	Canada	2012.05	2239.07	1827.99	3188.19	3711.87	2729.83	11.68	13.07	10.54	10.63	12.47	8.97	-0.53	-0.80	-0.26
Female	Cape Verde	6.29	8.05	3.74	13.18	16.54	7.45	4.85	6.26	2.71	5.30	6.64	2.93	0.30	0.20	0.41
Female	Central African Republic	133.53	202.99	86.28	176.38	302.77	107.87	17.68	26.46	12.22	13.30	22.42	8.49	-1.04	-1.11	-0.98
Female	Chad	179.36	237.42	113.20	250.19	326.85	183.31	9.97	13.29	6.39	7.98	10.44	5.85	-0.90	-0.97	-0.83
Female	Chile	1157.97	1278.07	1044.21	2149.87	2560.65	1786.80	19.91	21.99	17.96	17.56	20.95	14.54	-0.52	-0.65	-0.39
Female	China	61296.23	74076.43	54142.30	75144.44	91247.13	66648.98	12.23	14.87	10.91	7.75	9.54	6.89	-2.11	-2.45	-1.77
Female	Colombia	3057.79	3321.31	2844.95	5958.73	7238.99	4947.22	29.76	32.29	27.68	20.57	24.95	17.12	-1.41	-1.72	-1.09
Female	Comoros	39.17	58.90	26.45	59.50	82.41	44.29	24.83	37.03	16.89	19.92	27.57	14.91	-0.76	-0.85	-0.66
Female	Costa Rica	235.35	259.81	213.50	478.06	553.11	413.84	22.39	24.66	20.35	18.05	20.90	15.60	-1.41	-1.77	-1.04
Female	Croatia	765.15	854.83	685.05	512.95	596.07	439.70	20.47	22.77	18.41	11.73	13.68	10.02	-1.40	-1.68	-1.12
Female	Cuba	765.17	838.68	695.86	1381.98	1653.29	1139.60	14.23	15.66	12.96	15.24	18.27	12.51	0.24	-0.05	0.53
Female	Cyprus	84.15	103.39	53.95	85.71	108.01	68.74	18.76	23.05	11.83	9.08	11.56	7.29	-3.21	-3.63	-2.78
Female	Czech Republic	1754.22	1893.34	1617.71	1134.86	1327.88	979.22	22.72	24.73	20.86	11.40	13.48	9.72	-2.67	-2.82	-2.51
Female	Democratic Republic of the Congo	1338.79	1926.73	960.62	2235.61	3542.44	1521.37	12.90	18.50	9.48	10.45	16.51	6.97	-0.88	-1.01	-0.76
Female	Denmark	427.34	476.15	384.07	531.11	617.85	453.82	11.23	12.62	10.02	10.76	12.59	9.11	-0.33	-0.51	-0.15
Female	Djibouti	29.67	45.34	18.19	74.08	113.02	47.28	22.65	34.22	14.71	19.73	30.18	12.72	-0.61	-0.65	-0.57
Female	Dominica	5.01	5.57	4.46	6.53	7.45	5.60	13.03	14.54	11.56	15.24	17.52	13.07	0.60	0.20	1.00
Female	Dominican Republic	313.32	370.18	236.03	648.27	828.14	476.61	14.09	16.72	10.57	13.17	16.88	9.68	0.30	-0.44	1.06
Female	Ecuador	576.56	630.97	526.00	3147.13	3689.25	2688.55	18.52	20.19	16.89	40.63	47.59	34.74	3.51	3.07	3.95
Female	Egypt	1929.19	3441.83	1508.67	3980.16	6848.90	2760.53	10.66	20.51	8.28	11.00	20.11	7.61	0.29	0.18	0.40
Female	El Salvador	669.24	796.90	314.80	556.20	836.36	411.19	38.92	46.37	18.31	17.19	25.83	12.79	-2.95	-3.93	-1.95

© 2020 Deng Y et al. *JAMA Network Open.*

Sex	Location	Disability adjusted life-years						Age-standardized DALY rate (per 100,000)						EAPC		
		1990(95%UI)			2017(95%UI)			1990(95%UI)			2017(95%UI)			1990-2017(95%CI)		
		DALYs (No.)	UL	LL	DALYs (No.)	UL	LL	95%UI	UL	LL	95%UI	UL	LL	EAPC	LL	UL
Female	Equatorial Guinea	22.57	36.03	13.31	39.03	68.09	21.15	17.66	27.70	11.21	12.02	21.02	6.70	-1.43	-1.64	-1.21
Female	Eritrea	202.29	301.77	127.62	464.06	650.31	310.87	25.06	35.93	16.84	24.26	33.81	16.67	-0.16	-0.22	-0.10
Female	Estonia	211.96	237.58	190.35	216.93	270.49	172.71	18.34	20.56	16.38	15.85	20.25	12.33	-0.82	-1.07	-0.57
Female	Ethiopia	13079.94	19614.20	6699.47	13691.51	18377.31	9875.19	83.53	122.24	46.43	43.63	58.06	32.23	-2.80	-2.95	-2.65
Female	Fiji	96.52	127.63	47.16	168.92	222.52	78.01	45.41	60.43	22.36	41.07	53.85	19.32	-0.44	-0.76	-0.12
Female	Finland	670.41	725.30	613.84	679.16	779.17	578.54	16.40	17.77	15.00	12.10	13.91	10.32	-0.94	-1.14	-0.75
Female	France	7491.95	8028.07	7015.01	5287.60	6089.19	4567.17	16.80	18.18	15.56	8.55	10.04	7.27	-2.52	-2.67	-2.38
Female	Gabon	52.61	75.88	35.59	62.25	104.36	41.32	15.68	22.62	10.71	10.31	17.46	6.91	-1.49	-1.67	-1.32
Female	Gambia	23.69	29.45	17.92	58.06	78.62	44.06	9.78	12.35	7.56	9.41	13.04	7.06	0.00	-0.08	0.08
Female	Georgia	357.03	409.26	287.30	495.73	564.73	427.12	9.86	11.30	7.99	16.68	19.06	14.23	2.52	1.60	3.45
Female	Germany	13892.35	14828.26	12932.65	10551.77	12678.76	8772.69	19.65	21.12	18.18	12.91	15.64	10.60	-1.81	-2.01	-1.60
Female	Ghana	63.23	78.16	49.17	152.33	184.00	123.77	1.33	1.67	1.02	1.31	1.61	1.05	-0.06	-0.10	-0.02
Female	Greece	899.54	988.89	802.58	989.12	1147.33	847.89	11.18	12.39	9.97	9.12	10.66	7.72	-0.90	-1.11	-0.70
Female	Greenland	2.76	3.24	2.06	5.33	6.91	2.25	15.20	18.02	10.24	16.69	21.51	7.06	0.88	0.20	1.56
Female	Grenada	12.22	13.56	10.96	14.62	16.68	12.76	32.06	35.58	28.66	20.94	23.86	18.28	-0.96	-1.52	-0.38
Female	Grenadines	11.16	12.56	9.98	19.21	22.01	16.67	27.32	30.83	24.27	29.69	34.08	25.70	0.27	-0.16	0.70
Female	Guam	4.88	6.81	4.06	15.01	18.01	11.57	11.42	15.78	9.58	16.36	19.60	12.67	1.60	1.11	2.10
Female	Guatemala	450.26	494.91	409.64	1099.44	1300.68	903.61	20.63	22.62	18.78	17.38	20.52	14.36	-2.22	-2.88	-1.55
Female	Guinea	265.16	326.17	215.46	409.21	536.76	313.40	12.58	15.33	10.50	11.41	14.63	8.86	-0.38	-0.46	-0.31
Female	Guinea-Bissau	43.16	57.97	23.39	47.97	68.94	32.73	15.05	20.09	8.58	10.01	14.12	6.73	-1.66	-1.78	-1.55
Female	Guyana	33.33	36.86	29.81	55.62	67.35	45.63	14.14	15.65	12.59	15.82	18.99	13.07	0.44	0.16	0.73
Female	Haiti	590.49	995.93	300.38	983.47	1564.63	614.91	29.86	50.13	15.77	23.56	36.82	15.08	-0.84	-0.92	-0.76
Female	Honduras	208.56	251.60	171.21	409.13	803.74	262.54	17.55	21.13	14.16	12.51	23.92	8.17	-1.12	-1.61	-0.62
Female	Hungary	1939.97	2126.09	1779.16	1146.75	1314.97	1001.92	23.24	25.44	21.27	11.68	13.60	10.11	-2.58	-2.82	-2.33

© 2020 Deng Y et al. *JAMA Network Open.*

Sex	Location	Disability adjusted life-years						Age-standardized DALY rate (per 100,000)						EAPC		
		1990(95%UI)			2017(95%UI)			1990(95%UI)			2017(95%UI)			1990-2017(95%CI)		
		DALYs (No.)	UL	LL	DALYs (No.)	UL	LL	95%UI	UL	LL	95%UI	UL	LL	EAPC	LL	UL
Female	Iceland	54.10	60.62	47.98	47.21	55.34	40.41	37.44	41.97	32.95	19.19	22.66	16.35	-2.18	-2.35	-2.00
Female	India	63063.09	84777.72	46130.57	124747.31	141082.93	103233.53	19.41	26.25	14.70	19.96	22.47	16.56	-0.04	-0.18	0.09
Female	Indonesia	16747.80	20525.98	11730.02	23643.25	29410.48	17819.26	26.81	32.32	19.28	19.76	24.50	15.15	-1.17	-1.25	-1.09
Female	Iran	1543.40	1923.74	1263.26	4879.91	5609.55	3176.48	10.13	12.31	8.39	12.86	14.73	8.30	1.62	1.17	2.08
Female	Iraq	1050.46	1513.89	665.35	1729.37	2188.23	1231.57	21.38	31.52	14.20	12.80	16.05	8.82	-2.12	-2.44	-1.80
Female	Ireland	323.02	356.98	288.72	349.48	413.86	293.45	15.07	16.83	13.47	10.14	12.05	8.48	-1.09	-1.39	-0.80
Female	Israel	443.47	489.90	399.31	896.26	1057.38	761.24	16.74	18.41	15.14	15.74	18.66	13.40	-0.47	-0.78	-0.15
Female	Italy	9429.22	10375.27	8605.00	7763.38	9163.47	6577.72	20.63	22.98	18.54	12.63	15.44	10.59	-1.75	-1.95	-1.54
Female	Ivory Coast	451.41	581.30	335.83	891.95	1233.79	650.63	13.60	16.93	10.79	11.97	16.24	8.86	-0.50	-0.62	-0.37
Female	Jamaica	114.30	131.36	102.10	343.44	438.12	269.88	11.74	13.53	10.43	23.01	29.36	18.07	2.61	2.17	3.04
Female	Japan	14841.49	15912.89	13941.21	19945.92	22049.61	18380.83	15.50	16.70	14.49	11.94	13.61	10.66	-0.98	-1.07	-0.89
Female	Jordan	233.99	303.18	158.23	447.96	631.57	331.85	23.85	30.84	17.30	14.07	19.51	10.48	-2.47	-3.03	-1.91
Female	Kazakhstan	2333.95	2799.21	2056.20	1657.15	1949.71	1439.26	28.52	34.20	25.08	16.18	18.93	14.09	-3.12	-3.55	-2.68
Female	Kenya	432.17	579.54	333.95	997.62	1299.44	797.18	7.13	9.09	5.57	6.35	8.19	5.13	-0.50	-0.57	-0.43
Female	Kiribati	2.38	2.81	1.94	3.91	5.04	2.92	10.68	12.58	8.70	9.54	12.19	7.06	-0.31	-0.40	-0.23
Female	Kuwait	85.52	96.25	74.69	127.02	158.59	104.51	23.59	26.45	20.85	9.94	12.09	8.42	-3.42	-4.26	-2.57
Female	Kyrgyzstan	373.77	425.22	325.23	318.84	372.37	280.10	19.76	22.49	17.26	11.96	13.86	10.58	-1.68	-1.83	-1.53
Female	Laos	535.10	840.34	277.56	587.12	832.29	392.88	41.03	62.94	21.97	23.74	33.28	16.13	-2.30	-2.43	-2.17
Female	Latvia	336.60	375.56	303.48	343.66	421.93	277.83	16.73	18.67	15.06	15.82	19.76	12.74	-0.53	-0.90	-0.16
Female	Lebanon	380.14	498.47	263.28	917.77	1231.57	697.74	24.96	33.29	17.96	23.96	31.99	18.30	-0.21	-0.36	-0.06
Female	Lesotho	75.84	106.31	56.56	114.07	180.47	71.74	12.78	17.94	9.59	15.22	23.93	9.55	1.49	1.07	1.90
Female	Liberia	72.41	94.94	44.24	90.84	126.81	62.12	11.76	15.40	7.28	7.67	10.81	5.29	-1.86	-2.12	-1.60
Female	Libya	238.37	319.82	178.28	692.15	946.48	508.61	19.80	28.21	14.93	23.45	31.97	17.17	0.87	0.66	1.09
Female	Lithuania	436.54	487.05	392.27	436.09	504.64	373.01	17.13	19.23	15.29	14.84	17.36	12.56	-0.98	-1.39	-0.56

© 2020 Deng Y et al. *JAMA Network Open.*

Sex	Location	Disability adjusted life-years						Age-standardized DALY rate (per 100,000)						EAPC		
		1990(95%UI)			2017(95%UI)			1990(95%UI)			2017(95%UI)			1990-2017(95%CI)		
		DALYs (No.)	UL	LL	DALYs (No.)	UL	LL	95%UI	UL	LL	95%UI	UL	LL	EAPC	LL	UL
Female	Luxembourg	68.73	77.51	60.66	65.33	84.19	52.78	22.77	25.71	20.25	14.08	18.24	11.33	-1.97	-2.14	-1.81
Female	Macedonia	145.89	174.37	90.51	167.28	208.34	124.26	13.84	16.36	8.84	10.47	12.95	7.74	-1.61	-1.89	-1.32
Female	Madagascar	897.19	1255.63	632.13	1517.48	2126.56	1059.20	22.62	31.43	16.53	18.48	25.67	12.88	-0.77	-0.94	-0.60
Female	Malawi	896.98	1252.58	407.58	1435.70	2068.29	945.85	27.55	36.72	14.87	23.88	32.94	16.47	-1.11	-1.42	-0.79
Female	Malaysia	1667.33	2011.70	1361.26	3559.63	4717.40	2790.33	29.41	36.07	24.75	26.30	34.58	20.80	-0.37	-0.45	-0.28
Female	Maldives	14.10	22.55	5.35	19.17	23.77	14.74	30.54	48.31	11.40	13.70	17.11	10.31	-3.56	-3.79	-3.33
Female	Mali	714.94	858.56	554.68	1051.63	1542.90	697.72	24.92	29.94	20.80	17.88	26.07	12.45	-1.52	-1.65	-1.39
Female	Malta	50.92	56.76	45.49	64.73	75.19	55.69	21.32	23.77	19.13	15.64	18.21	13.40	-1.10	-1.27	-0.92
Female	Marshall Islands	2.48	3.36	1.73	5.57	8.13	3.36	25.83	35.28	17.85	29.01	41.60	18.18	0.42	0.16	0.68
Female	Mauritania	74.94	97.64	42.43	86.07	119.76	60.64	11.91	15.62	6.82	7.49	10.44	5.22	-1.89	-2.00	-1.77
Female	Mauritius	54.18	60.25	48.24	104.06	120.08	88.50	12.53	13.93	11.20	11.55	13.28	9.85	0.16	-0.38	0.71
Female	Mexico	5308.94	5552.36	5095.95	14487.57	15425.73	13668.06	21.19	22.13	20.39	23.11	24.56	21.85	0.21	0.10	0.32
Female	Micronesia	6.78	9.18	4.98	8.84	12.39	6.10	25.16	33.62	18.75	22.34	30.70	15.91	-0.51	-0.55	-0.48
Female	Moldova	394.16	443.48	348.28	360.68	419.00	314.59	14.66	16.47	12.98	11.80	13.71	10.28	-0.72	-1.05	-0.39
Female	Mongolia	75.39	90.29	46.05	158.64	209.96	74.13	12.39	14.97	7.17	12.63	16.95	5.16	0.04	-0.28	0.37
Female	Montenegro	60.22	74.75	47.05	83.84	102.02	66.54	17.05	21.00	13.35	16.66	20.48	13.25	-0.23	-0.47	0.01
Female	Morocco	2455.61	3027.99	1863.62	4171.92	5615.30	3052.26	26.06	32.32	20.81	23.70	31.84	17.50	-0.32	-0.37	-0.28
Female	Mozambique	1178.38	1769.40	843.07	2004.48	2830.48	1310.23	23.24	35.54	16.89	20.93	29.31	14.16	-0.46	-0.66	-0.27
Female	Myanmar	6965.03	10969.98	3774.48	7448.04	10294.35	5239.86	48.23	73.75	27.27	27.92	38.63	19.89	-2.24	-2.42	-2.06
Female	Namibia	67.27	96.13	44.45	100.46	137.88	73.80	14.22	22.43	9.47	10.70	15.46	7.83	-1.43	-2.07	-0.79
Female	Nepal	1547.42	2607.50	1020.32	2545.31	4189.15	1775.40	23.09	40.11	15.40	18.89	30.63	13.38	-0.77	-1.05	-0.49
Female	Netherlands	1348.21	1471.11	1223.46	1834.54	2116.54	1550.93	12.56	13.82	11.28	11.81	13.91	9.87	-0.14	-0.28	0.01
Female	New Zealand	273.09	306.16	243.32	379.01	440.23	324.24	13.21	14.81	11.75	11.08	12.98	9.47	-0.24	-0.49	0.01
Female	Nicaragua	125.59	185.02	104.03	413.69	503.79	326.36	12.82	19.48	10.57	16.03	19.56	12.65	1.25	0.85	1.66

© 2020 Deng Y et al. *JAMA Network Open.*

Sex	Location	Disability adjusted life-years						Age-standardized DALY rate (per 100,000)						EAPC		
		1990(95%UI)			2017(95%UI)			1990(95%UI)			2017(95%UI)			1990-2017(95%CI)		
		DALYs (No.)	UL	LL	DALYs (No.)	UL	LL	95%UI	UL	LL	95%UI	UL	LL	EAPC	LL	UL
Female	Niger	207.19	270.56	124.93	301.24	404.82	217.77	10.36	13.32	6.59	6.56	8.66	4.68	-2.07	-2.23	-1.90
Female	Nigeria	1508.29	2206.47	811.16	2735.20	3989.80	1781.05	6.60	9.83	3.24	5.57	8.17	3.47	-0.86	-0.96	-0.76
Female	North Korea	1472.99	2047.96	1055.74	2124.17	3186.19	1500.49	13.79	19.25	9.95	12.49	18.94	8.78	-0.38	-0.45	-0.30
Female	Northern Mariana Islands	1.31	1.71	0.97	2.98	3.80	2.39	14.04	17.85	10.82	10.80	13.30	8.77	-1.00	-1.27	-0.73
Female	Norway	462.16	493.96	434.51	537.58	598.40	485.75	13.87	15.01	12.90	12.69	14.27	11.30	-0.26	-0.37	-0.15
Female	Oman	72.81	99.09	50.04	175.94	228.74	130.20	16.92	23.19	11.82	15.85	20.41	11.77	-0.13	-0.46	0.20
Female	Pakistan	19549.50	26679.04	15269.99	55276.98	87452.81	35517.50	52.27	69.65	42.07	64.17	95.92	43.09	0.59	0.30	0.88
Female	Palestine	89.40	119.75	61.26	181.76	218.28	149.16	16.65	22.07	11.26	13.55	16.24	10.68	-0.49	-0.64	-0.35
Female	Panama	152.17	166.44	138.19	341.71	394.81	292.88	17.16	18.77	15.62	16.96	19.57	14.54	0.24	0.05	0.44
Female	Papua New Guinea	279.43	417.21	198.02	635.83	942.61	438.99	26.12	38.62	18.77	24.87	35.50	17.90	-0.08	-0.17	0.01
Female	Paraguay	216.43	254.82	179.13	602.28	805.07	381.21	17.20	20.27	13.97	21.22	28.29	13.27	0.95	0.73	1.17
Female	Peru	1616.32	1988.34	1361.29	3723.20	4764.47	2649.42	23.31	28.47	19.65	23.69	30.34	16.81	0.03	-0.23	0.29
Female	Philippines	6962.36	8075.97	6137.46	20455.90	25509.34	16380.36	37.36	43.03	33.07	49.07	61.07	39.40	1.19	1.01	1.37
Female	Poland	6665.72	7098.21	6222.15	4933.24	5703.54	4268.12	26.37	28.13	24.58	14.39	16.81	12.34	-2.84	-3.18	-2.50
Female	Portugal	1551.49	1704.62	1416.92	1237.55	1445.81	1064.65	19.94	21.85	18.14	10.66	12.62	9.06	-2.71	-3.11	-2.31
Female	Puerto Rico	338.27	375.02	302.62	247.88	296.25	211.21	17.01	18.87	15.20	7.83	9.33	6.63	-3.03	-3.50	-2.57
Female	Qatar	23.80	33.39	7.72	35.06	49.58	24.96	45.28	60.72	15.43	10.67	14.62	7.91	-6.36	-7.03	-5.68
Female	Republic of Congo	134.04	206.13	83.48	228.40	394.59	143.02	18.95	28.61	12.49	14.80	24.97	9.80	-1.06	-1.22	-0.91
Female	Romania	2448.02	2655.00	2247.34	2450.21	2817.30	2133.70	16.17	17.58	14.83	14.11	16.33	12.25	-0.88	-1.14	-0.62
Female	Russia	15732.21	17361.81	14724.11	20196.81	21994.33	18583.03	13.91	15.45	12.95	16.20	17.87	14.73	0.38	-0.05	0.82
Female	Rwanda	710.33	1052.70	396.98	783.86	1157.93	570.85	29.74	43.80	16.75	17.36	25.15	12.90	-2.52	-2.78	-2.27
Female	Saint Lucia	16.11	17.75	14.51	28.81	32.97	24.85	31.69	35.14	28.38	26.65	30.45	23.05	-0.80	-1.32	-0.28
Female	Saint Vincent	11.16	12.56	9.98	19.21	22.01	16.67	27.32	30.83	24.27	29.69	34.08	25.70	0.27	-0.16	0.70

© 2020 Deng Y et al. *JAMA Network Open.*

Sex	Location	Disability adjusted life-years						Age-standardized DALY rate (per 100,000)						EAPC		
		1990(95%UI)			2017(95%UI)			1990(95%UI)			2017(95%UI)			1990-2017(95%CI)		
		DALYs (No.)	UL	LL	DALYs (No.)	UL	LL	95%UI	UL	LL	95%UI	UL	LL	EAPC	LL	UL
Female	Samoa	15.05	19.52	11.58	20.50	28.22	14.80	28.12	40.67	21.08	26.85	39.08	19.07	-0.25	-0.30	-0.20
Female	Sao Tome and Principe	3.88	6.27	2.38	5.41	7.18	3.98	9.14	14.47	5.78	8.40	11.09	6.12	-0.72	-0.94	-0.50
Female	Saudi Arabia	632.10	849.50	470.66	2854.60	4014.43	1952.32	16.47	23.82	12.19	23.86	31.32	18.33	1.99	1.74	2.25
Female	Senegal	200.96	260.57	126.54	302.62	393.38	229.58	9.40	11.95	6.13	6.97	9.12	5.29	-1.37	-1.51	-1.24
Female	Serbia	946.19	1184.35	728.39	1101.43	1311.53	829.90	15.56	19.49	12.07	14.21	16.97	10.73	-0.21	-0.39	-0.04
Female	Seychelles	3.59	4.24	2.21	4.45	5.23	3.66	11.40	13.59	7.04	7.86	9.27	6.50	-1.18	-1.42	-0.94
Female	Sierra Leone	128.58	180.74	80.13	190.18	264.58	135.91	10.72	15.00	6.88	9.15	12.60	6.44	-0.56	-0.68	-0.43
Female	Singapore	208.70	233.49	187.52	332.51	405.14	273.81	15.60	17.32	14.07	9.18	11.17	7.56	-1.59	-1.79	-1.38
Female	Slovakia	663.02	747.26	542.72	594.55	757.84	483.60	20.01	22.67	16.38	12.58	16.36	10.19	-1.93	-2.04	-1.82
Female	Slovenia	248.18	275.99	224.49	191.48	225.36	160.54	17.34	19.42	15.73	9.62	11.57	7.96	-2.41	-2.58	-2.24
Female	Solomon Islands	17.23	25.14	12.28	38.77	55.43	27.58	22.84	32.43	16.98	21.37	29.51	15.52	-0.18	-0.27	-0.08
Female	Somalia	445.07	754.37	191.90	1079.36	1556.71	741.20	22.90	37.25	12.12	22.34	31.84	15.48	-0.33	-0.46	-0.20
Female	South Africa	1547.97	1780.27	1300.16	2263.40	2835.29	1985.52	11.22	13.22	9.38	8.54	10.65	7.49	-1.23	-1.91	-0.53
Female	South Korea	1896.40	2037.97	1687.73	11307.34	13539.93	9353.22	10.14	10.87	9.03	29.54	35.90	24.04	5.66	4.16	7.19
Female	South Sudan	365.27	655.22	167.08	573.38	860.21	360.51	22.32	37.65	11.91	20.70	31.46	13.23	-0.44	-0.64	-0.25
Female	Spain	3742.97	4029.88	3479.13	4155.82	4741.18	3619.72	12.96	14.02	11.99	9.55	11.02	8.25	-1.11	-1.21	-1.01
Female	Sri Lanka	1437.50	1697.99	1017.85	2154.84	2967.08	1465.18	23.85	27.96	17.07	15.58	21.27	10.67	-2.08	-2.49	-1.68
Female	Sudan	914.99	1554.42	522.77	1507.24	2201.22	948.35	16.01	26.46	9.86	13.86	19.89	9.04	-0.47	-0.51	-0.44
Female	Suriname	29.89	33.78	26.23	64.46	76.05	54.77	20.53	23.23	17.96	20.38	24.04	17.33	0.09	-0.12	0.30
Female	Swaziland	31.85	44.98	23.62	47.90	75.76	29.83	16.21	23.07	11.79	13.18	20.69	8.19	-0.30	-0.81	0.21
Female	Sweden	945.47	1021.92	871.12	935.11	1060.45	825.35	12.37	13.46	11.31	10.27	11.82	8.95	-0.46	-0.60	-0.31
Female	Switzerland	841.93	935.75	750.59	817.02	966.79	693.75	16.41	18.42	14.48	10.46	12.43	8.82	-1.63	-1.85	-1.42
Female	Syria	167.24	226.98	104.87	325.67	428.71	221.94	5.67	7.73	3.47	4.66	6.07	3.13	-0.97	-1.15	-0.78

© 2020 Deng Y et al. *JAMA Network Open.*

Sex	Location	Disability adjusted life-years						Age-standardized DALY rate (per 100,000)						EAPC		
		1990(95%UI)			2017(95%UI)			1990(95%UI)			2017(95%UI)			1990-2017(95%CI)		
		DALYs (No.)	UL	LL	DALYs (No.)	UL	LL	95%UI	UL	LL	95%UI	UL	LL	EAPC	LL	UL
Female	Tajikistan	67.72	87.55	55.89	120.96	144.19	100.18	4.00	5.25	3.23	3.95	4.70	3.16	-0.53	-0.77	-0.29
Female	Tanzania	1934.67	2768.85	1176.48	3526.20	5099.36	2560.48	23.23	33.02	15.65	19.55	27.87	14.44	-0.99	-1.24	-0.73
Female	Thailand	4880.12	7505.40	4044.22	7321.54	14830.92	5557.07	22.93	34.40	19.11	13.94	28.44	10.52	-2.24	-2.56	-1.92
Female	Timor-Leste	56.21	81.50	32.94	90.13	119.67	65.45	29.99	42.53	19.35	20.96	27.76	15.55	-1.52	-1.66	-1.38
Female	Tobago	88.11	97.49	79.52	138.93	188.32	102.82	18.60	20.62	16.74	15.15	20.57	11.14	-1.65	-2.20	-1.10
Female	Togo	105.20	146.12	62.76	159.79	210.25	117.07	10.79	14.80	6.74	6.79	8.95	4.94	-1.94	-2.11	-1.77
Female	Tonga	4.70	5.97	3.72	6.78	8.92	5.03	15.66	19.90	12.38	15.79	20.83	11.74	-0.01	-0.13	0.12
Female	Trinidad	88.11	97.49	79.52	138.93	188.32	102.82	18.60	20.62	16.74	15.15	20.57	11.14	-1.65	-2.20	-1.10
Female	Tunisia	434.15	547.54	360.90	895.35	1283.50	620.68	14.48	19.65	12.02	14.16	20.25	9.87	-0.20	-0.32	-0.08
Female	Turkey	3845.46	5779.54	2525.15	5594.31	8593.02	4451.30	18.99	27.78	12.56	12.08	18.57	9.63	-1.86	-2.02	-1.70
Female	Turkmenistan	173.93	194.67	154.94	229.80	268.20	195.59	13.98	15.64	12.43	10.00	11.59	8.59	-1.22	-1.73	-0.72
Female	Uganda	1216.00	1572.19	937.16	2446.47	3440.41	1665.80	23.54	29.76	18.51	20.40	27.67	14.55	-0.70	-0.82	-0.59
Female	UK	4945.03	5257.53	4642.32	5330.52	5761.89	4940.81	10.56	11.33	9.88	9.48	10.41	8.68	-0.46	-0.57	-0.36
Female	Ukraine	5436.15	6328.52	4680.71	6077.61	6973.90	5311.74	12.49	14.61	10.73	15.17	17.66	13.21	0.47	0.05	0.89
Female	United Arab Emirates	52.45	81.00	32.83	288.27	438.81	179.74	21.18	35.54	12.85	19.12	29.17	11.18	-0.21	-0.41	-0.01
Female	Uruguay	227.16	256.12	204.17	336.70	403.94	275.46	10.84	12.18	9.73	12.96	15.75	10.53	0.13	-0.43	0.69
Female	USA	16630.96	18055.16	15469.44	28488.44	31747.20	25809.97	9.79	10.72	9.05	10.98	12.37	9.87	0.49	0.35	0.63
Female	Uzbekistan	246.44	358.93	209.86	643.63	781.13	525.60	3.42	5.09	2.88	4.77	5.75	3.89	1.18	0.87	1.49
Female	Vanuatu	8.47	13.43	5.63	21.72	36.15	13.96	23.89	37.66	16.34	24.65	39.94	15.93	0.15	0.06	0.23
Female	Venezuela	797.35	874.67	726.64	2603.49	3228.37	2001.63	14.03	15.35	12.78	16.96	21.07	13.05	0.67	0.52	0.82
Female	Vietnam	9136.27	12384.31	7351.18	19138.21	25376.61	14539.06	34.27	50.26	27.14	36.13	47.48	27.39	0.63	0.37	0.88
Female	Virgin Islands	4.74	5.49	4.09	8.53	10.25	7.04	9.38	10.80	8.14	9.13	11.09	7.57	0.11	-0.08	0.29
Female	Yemen	504.22	932.36	259.17	1181.46	1849.40	758.89	15.44	27.62	8.81	14.30	21.93	9.48	-0.32	-0.35	-0.29

© 2020 Deng Y et al. *JAMA Network Open.*

Sex	Location	Disability adjusted life-years						Age-standardized DALY rate (per 100,000)						EAPC		
		1990(95%UI)			2017(95%UI)			1990(95%UI)			2017(95%UI)			1990-2017(95%CI)		
		DALYs (No.)	UL	LL	DALYs (No.)	UL	LL	95%UI	UL	LL	95%UI	UL	LL	EAPC	LL	UL
Female	Zambia	767.37	1115.38	474.82	1087.72	1526.10	779.66	31.86	45.35	20.92	20.63	28.76	15.08	-2.19	-2.65	-1.73
Female	Zimbabwe	556.89	677.42	399.32	1313.31	1736.84	792.87	20.95	25.14	16.16	26.93	34.86	18.23	1.84	1.06	2.63
Male	Afghanistan	537.19	992.04	205.41	941.18	1504.63	602.42	13.22	24.26	5.06	13.26	20.61	8.90	0.13	-0.03	0.30
Male	Albania	178.95	202.95	157.14	245.19	318.22	184.44	14.27	16.11	12.51	13.57	17.62	10.28	-0.42	-0.72	-0.12
Male	Algeria	951.31	1128.01	760.95	2663.44	3235.97	2107.29	11.20	13.22	9.22	14.05	16.88	11.18	1.05	0.89	1.20
Male	American Samoa	1.25	1.51	1.03	2.22	2.66	1.84	8.71	10.57	7.08	9.42	11.21	7.79	0.34	-0.23	0.90
Male	Andorra	2.82	3.68	2.12	5.27	7.16	3.78	8.86	11.39	6.68	8.29	11.19	6.02	-0.20	-0.27	-0.12
Male	Angola	227.44	338.90	144.44	531.35	686.87	396.56	8.68	12.20	5.89	8.50	10.83	6.29	-0.17	-0.26	-0.08
Male	Antigua	1.22	1.38	1.08	2.71	3.14	2.32	5.20	5.89	4.59	5.52	6.36	4.76	0.30	0.21	0.39
Male	Argentina	2273.54	2446.94	2092.04	2570.14	3014.07	2143.09	14.86	15.99	13.68	11.09	12.99	9.25	-1.21	-1.45	-0.97
Male	Armenia	65.04	85.80	51.14	231.14	260.72	205.57	4.96	6.55	3.95	13.00	14.60	11.56	3.52	2.52	4.53
Male	Australia	780.39	860.39	709.70	1815.09	2181.16	1504.52	8.52	9.38	7.76	10.68	12.80	8.81	1.30	1.13	1.47
Male	Austria	742.87	814.52	669.20	790.18	919.02	673.64	16.11	17.64	14.59	10.86	12.63	9.26	-2.22	-2.52	-1.92
Male	Azerbaijan	244.22	282.69	206.86	404.66	490.01	323.85	9.30	10.73	7.96	8.64	10.27	7.08	-1.19	-1.55	-0.83
Male	Bahamas	11.96	13.65	10.40	30.78	36.72	25.84	14.11	16.15	12.28	15.90	18.70	13.41	0.72	0.59	0.85
Male	Bahrain	10.53	12.24	8.98	38.98	47.69	31.00	8.01	9.57	6.70	5.31	6.42	4.33	-2.21	-2.58	-1.85
Male	Bangladesh	4029.54	5116.67	3097.86	6606.95	8892.84	4694.77	11.72	14.98	9.18	9.35	12.38	6.71	-0.86	-1.03	-0.69
Male	Barbados	12.10	13.53	10.82	23.03	26.98	19.39	10.17	11.47	9.14	11.29	13.22	9.54	0.53	0.30	0.75
Male	Barbuda	1.22	1.38	1.08	2.71	3.14	2.32	5.20	5.89	4.59	5.52	6.36	4.76	0.30	0.21	0.39
Male	Belarus	539.93	618.98	478.79	777.66	973.15	650.14	10.35	11.77	9.21	12.70	15.88	10.71	0.64	0.15	1.14
Male	Belgium	991.11	1102.82	896.62	1165.55	1397.23	979.56	15.44	17.06	14.02	13.11	15.80	11.02	-0.22	-0.49	0.05
Male	Belize	2.46	2.90	2.13	13.00	14.75	11.44	4.56	5.38	3.95	8.37	9.49	7.39	2.08	1.61	2.55
Male	Benin	81.10	98.82	66.02	180.96	250.92	124.40	7.10	8.70	5.81	7.00	9.72	4.86	-0.07	-0.15	0.00
Male	Bermuda	6.03	6.78	5.32	9.40	10.88	8.14	20.30	22.80	17.88	18.22	21.01	15.76	0.06	-0.19	0.32

© 2020 Deng Y et al. *JAMA Network Open.*

Sex	Location	Disability adjusted life-years						Age-standardized DALY rate (per 100,000)						EAPC		
		1990(95%UI)			2017(95%UI)			1990(95%UI)			2017(95%UI)			1990-2017(95%CI)		
		DALYs (No.)	UL	LL	DALYs (No.)	UL	LL	95%UI	UL	LL	95%UI	UL	LL	EAPC	LL	UL
Male	Bhutan	21.34	27.49	16.12	48.61	70.13	31.34	11.81	15.08	9.01	12.38	18.04	7.99	0.29	0.19	0.39
Male	Bolivia	308.98	429.56	230.20	827.32	1110.13	602.29	16.87	23.48	12.73	18.79	25.30	13.85	0.48	0.32	0.65
Male	Bosnia and Herzegovina	202.52	234.17	175.99	209.57	250.27	176.75	10.18	11.65	8.94	7.92	9.37	6.73	-1.54	-1.80	-1.27
Male	Botswana	27.57	38.60	19.24	48.06	70.55	31.61	8.31	11.48	5.87	6.78	9.88	4.54	-0.82	-0.97	-0.68
Male	Brazil	5706.85	5981.13	5453.55	12288.74	13102.99	11515.37	11.48	12.04	10.99	11.46	12.21	10.74	0.06	-0.04	0.17
Male	Brunei	8.60	10.71	7.05	25.43	29.98	21.77	13.04	16.31	10.52	14.51	17.02	12.47	0.83	0.64	1.02
Male	Bulgaria	735.83	810.29	664.13	585.68	679.59	507.89	12.57	13.81	11.44	10.53	12.11	9.10	-1.00	-1.18	-0.81
Male	Burkina Faso	153.83	203.54	111.51	284.21	353.56	225.27	6.37	8.53	4.59	5.95	7.38	4.77	-0.43	-0.61	-0.25
Male	Burundi	218.78	324.20	138.42	429.17	566.91	319.80	14.84	21.64	9.71	13.81	17.76	10.49	-0.26	-0.36	-0.15
Male	Cambodia	408.90	594.88	290.96	802.34	1040.02	621.42	17.23	24.65	12.41	15.22	19.25	12.04	-0.49	-0.55	-0.42
Male	Cameroon	209.77	249.71	171.66	521.15	708.56	376.99	7.86	9.42	6.48	7.87	10.81	5.55	-0.13	-0.29	0.02
Male	Canada	1330.93	1450.24	1217.60	2630.80	3052.45	2274.13	8.94	9.71	8.20	9.54	10.99	8.26	0.38	0.21	0.55
Male	Cape Verde	4.55	6.17	3.34	10.24	13.50	7.08	4.57	6.15	3.33	4.77	6.32	3.26	-0.07	-0.21	0.06
Male	Central African Republic	68.58	106.61	41.77	117.74	184.13	74.51	9.71	14.79	6.24	8.77	13.08	5.97	-0.53	-0.61	-0.46
Male	Chad	92.23	116.48	68.48	200.79	253.26	155.43	5.86	7.47	4.31	5.89	7.44	4.55	-0.01	-0.19	0.16
Male	Chile	637.13	715.04	565.89	1127.49	1343.57	941.41	12.58	14.11	11.19	10.72	12.72	8.98	-0.47	-0.60	-0.34
Male	China	36108.73	39017.73	33577.81	97467.41	105681.61	88921.60	7.38	7.96	6.89	10.39	11.26	9.48	1.84	1.48	2.20
Male	Colombia	1558.61	1693.69	1437.30	2484.70	3023.93	2046.99	15.08	16.34	13.87	9.96	12.12	8.22	-1.81	-2.11	-1.51
Male	Comoros	18.02	22.92	13.83	36.53	52.06	26.25	12.75	16.14	9.80	13.94	19.77	10.03	0.44	0.24	0.64
Male	Costa Rica	125.82	137.93	115.16	291.06	337.14	250.44	12.27	13.49	11.22	12.32	14.24	10.57	-0.16	-0.50	0.18
Male	Croatia	509.33	568.00	453.01	319.32	367.48	277.78	17.92	19.88	16.04	9.30	10.71	8.12	-2.04	-2.35	-1.72
Male	Cuba	445.48	495.09	402.34	1022.54	1216.93	844.25	8.48	9.44	7.66	12.19	14.49	10.08	1.54	1.37	1.70

© 2020 Deng Y et al. *JAMA Network Open.*

Sex	Location	Disability adjusted life-years						Age-standardized DALY rate (per 100,000)						EAPC		
		1990(95%UI)			2017(95%UI)			1990(95%UI)			2017(95%UI)			1990-2017(95%CI)		
		DALYs (No.)	UL	LL	DALYs (No.)	UL	LL	95%UI	UL	LL	95%UI	UL	LL	EAPC	LL	UL
Male	Cyprus	52.40	64.89	44.25	106.05	128.32	84.66	13.12	16.30	11.07	12.49	15.15	10.07	-0.13	-0.36	0.11
Male	Czech Republic	1035.95	1132.02	935.65	734.11	854.36	632.01	17.69	19.26	16.05	8.74	10.12	7.52	-2.27	-2.49	-2.05
Male	Democratic Republic of the Congo	662.37	885.20	472.88	1340.42	1797.50	950.54	7.23	9.50	5.30	6.81	9.13	4.74	-0.32	-0.44	-0.20
Male	Denmark	213.77	241.91	191.20	342.66	403.56	292.13	6.40	7.20	5.72	7.45	8.74	6.40	0.42	0.02	0.82
Male	Djibouti	16.35	23.68	9.91	66.17	99.86	41.90	11.01	15.65	7.60	15.62	22.98	10.10	1.32	1.26	1.38
Male	Dominica	2.97	3.30	2.66	6.26	7.19	5.42	10.07	11.28	9.02	14.43	16.55	12.52	1.41	1.29	1.53
Male	Dominican Republic	183.41	215.29	156.52	484.28	630.21	364.98	8.31	9.79	7.12	10.16	13.17	7.67	0.08	-0.58	0.73
Male	Ecuador	268.31	291.14	243.94	1126.25	1328.99	944.24	8.60	9.32	7.80	15.22	17.91	12.78	2.56	1.73	3.40
Male	Egypt	1305.47	1528.83	1097.24	3285.22	4370.11	2447.29	6.79	7.85	5.80	8.33	11.00	6.22	0.95	0.73	1.17
Male	El Salvador	182.73	206.82	162.32	197.65	256.65	151.15	12.03	13.65	10.62	8.06	10.46	6.14	-1.64	-2.18	-1.09
Male	Equatorial Guinea	11.16	17.99	6.71	21.61	31.24	13.19	10.23	15.89	6.51	8.13	11.58	4.86	-1.10	-1.44	-0.75
Male	Eritrea	148.47	223.50	73.29	433.83	636.95	294.03	20.21	29.11	12.12	24.88	35.73	17.18	0.41	-0.08	0.90
Male	Estonia	86.36	96.76	77.31	93.16	118.79	75.25	10.75	11.98	9.67	10.00	12.74	8.04	-0.94	-1.49	-0.39
Male	Ethiopia	7314.26	12673.44	3897.98	10846.04	13472.19	8539.40	50.66	85.49	28.53	37.70	47.16	29.43	-1.24	-1.35	-1.12
Male	Fiji	62.21	78.01	48.85	70.05	84.83	57.07	24.98	31.31	19.31	17.50	21.30	14.42	-1.82	-2.30	-1.34
Male	Finland	313.03	347.19	284.69	508.19	593.21	429.16	10.75	11.86	9.81	10.98	12.89	9.31	0.43	0.28	0.59
Male	France	4556.86	4901.86	4266.65	5808.62	6798.01	5008.63	13.54	14.51	12.67	11.78	13.90	10.09	-0.07	-1.40	1.28
Male	Gabon	25.04	32.20	19.73	52.56	67.48	40.20	8.46	10.71	6.81	9.03	11.40	7.03	0.27	0.21	0.34
Male	Gambia	13.02	17.42	9.42	34.64	45.65	25.05	5.52	7.28	4.17	6.47	8.39	4.79	0.76	0.66	0.86
Male	Georgia	166.06	198.28	132.25	289.46	332.38	244.88	6.17	7.15	5.03	12.23	13.93	10.42	2.72	1.71	3.74
Male	Germany	7862.00	8338.18	7368.38	9987.16	11786.93	8394.94	15.65	16.59	14.69	13.79	16.39	11.50	-0.87	-1.29	-0.46
Male	Ghana	261.58	340.72	196.63	565.24	691.81	449.42	7.59	9.86	5.69	8.00	9.81	6.29	0.06	-0.09	0.21

© 2020 Deng Y et al. *JAMA Network Open.*

Sex	Location	Disability adjusted life-years						Age-standardized DALY rate (per 100,000)						EAPC		
		1990(95%UI)			2017(95%UI)			1990(95%UI)			2017(95%UI)			1990-2017(95%CI)		
		DALYs (No.)	UL	LL	DALYs (No.)	UL	LL	95%UI	UL	LL	95%UI	UL	LL	EAPC	LL	UL
Male	Greece	562.77	620.92	505.68	809.06	928.23	697.78	8.21	9.06	7.40	9.02	10.41	7.74	0.02	-0.26	0.31
Male	Greenland	1.64	1.99	1.32	2.51	2.93	2.12	7.55	9.16	6.14	6.59	7.60	5.64	-0.38	-0.60	-0.15
Male	Grenada	8.09	8.97	7.15	13.69	15.87	11.96	27.04	30.20	23.97	19.95	23.04	17.39	-0.19	-0.67	0.29
Male	Grenadines	3.17	3.53	2.82	9.55	11.02	8.16	9.01	10.12	7.97	13.78	15.83	11.85	1.69	1.56	1.82
Male	Guam	3.25	3.98	2.67	13.29	15.78	10.93	6.98	8.36	5.76	14.53	17.15	12.04	3.27	2.14	4.40
Male	Guatemala	190.98	210.26	174.57	482.86	571.88	400.27	8.63	9.53	7.81	8.67	10.27	7.24	-1.66	-2.33	-1.00
Male	Guinea	185.91	222.34	155.00	375.69	504.20	281.77	9.42	11.26	7.84	11.07	15.00	8.22	0.75	0.67	0.82
Male	Guinea-Bissau	22.03	32.53	13.74	33.72	50.47	21.12	9.11	13.29	5.81	8.32	12.34	5.30	-0.28	-0.34	-0.23
Male	Guyana	26.26	29.26	23.54	50.54	61.58	41.18	11.55	12.88	10.31	15.17	18.33	12.46	1.71	1.42	2.00
Male	Haiti	246.63	398.34	181.82	490.16	707.48	338.15	13.07	20.86	9.77	13.59	19.07	9.48	0.30	0.21	0.39
Male	Honduras	235.19	288.71	189.84	744.05	1025.96	464.06	20.10	24.54	16.22	24.74	34.16	15.63	0.67	0.56	0.79
Male	Hungary	1202.72	1320.35	1095.93	761.40	869.62	664.08	19.09	20.96	17.47	10.37	11.82	9.09	-2.45	-2.67	-2.22
Male	Iceland	39.25	43.68	35.26	82.88	95.34	71.59	29.80	33.20	26.80	35.57	40.90	30.78	0.78	0.61	0.95
Male	India	31658.41	37799.03	27310.06	77576.23	82402.88	72699.31	9.87	11.79	8.58	12.78	13.57	11.95	1.18	1.04	1.31
Male	Indonesia	6566.32	7569.51	5832.64	13525.65	15797.92	11935.02	11.13	12.87	9.80	12.22	14.19	10.78	0.34	0.25	0.42
Male	Iran	973.81	1192.45	856.93	3207.05	3485.02	2907.45	5.75	7.05	5.05	8.33	9.04	7.59	2.43	1.96	2.89
Male	Iraq	670.19	896.05	493.25	1283.31	1510.51	1097.62	13.95	18.73	10.25	9.10	10.63	7.87	-1.74	-2.01	-1.47
Male	Ireland	200.44	222.57	179.57	252.02	313.62	209.83	10.57	11.73	9.51	7.71	9.59	6.44	-0.68	-0.96	-0.39
Male	Israel	266.47	302.74	238.15	664.50	798.68	562.66	11.82	13.33	10.61	13.63	16.32	11.62	0.23	-0.14	0.60
Male	Italy	5568.31	6019.34	5123.41	6214.93	7340.00	5320.10	14.94	16.16	13.75	11.58	13.83	9.84	-0.82	-0.92	-0.71
Male	Ivory Coast	126.31	154.44	102.00	354.37	467.92	246.54	4.66	5.67	3.78	5.52	7.36	3.80	0.51	0.29	0.73
Male	Jamaica	53.44	61.32	47.09	172.74	221.86	123.37	6.21	7.14	5.44	12.13	15.54	8.66	2.69	2.30	3.08
Male	Japan	7543.25	7898.64	7225.96	12121.96	13494.31	11212.54	9.85	10.33	9.44	9.03	10.15	8.32	-0.31	-0.47	-0.16
Male	Jordan	145.37	207.53	104.19	332.77	409.93	264.22	13.99	20.00	10.21	8.96	11.05	7.14	-2.09	-2.31	-1.88

© 2020 Deng Y et al. *JAMA Network Open.*

Sex	Location	Disability adjusted life-years						Age-standardized DALY rate (per 100,000)						EAPC		
		1990(95%UI)			2017(95%UI)			1990(95%UI)			2017(95%UI)			1990-2017(95%CI)		
		DALYs (No.)	UL	LL	DALYs (No.)	UL	LL	95%UI	UL	LL	95%UI	UL	LL	EAPC	LL	UL
Male	Kazakhstan	1457.75	1851.32	1233.04	829.99	997.52	708.02	22.92	28.76	19.67	10.17	12.14	8.76	-4.13	-4.52	-3.73
Male	Kenya	298.53	358.39	225.89	1013.07	1250.63	796.79	5.27	6.26	4.01	7.18	8.86	5.64	1.32	1.09	1.55
Male	Kiribati	1.69	1.98	1.44	3.04	3.83	2.37	8.28	9.62	7.10	8.69	10.81	6.79	0.34	0.16	0.52
Male	Kuwait	53.94	60.88	47.55	148.38	179.02	122.74	9.19	10.25	8.19	8.63	10.35	7.15	1.06	0.42	1.71
Male	Kyrgyzstan	138.14	157.42	118.44	151.48	185.97	129.06	9.09	10.40	7.86	6.46	7.82	5.52	-0.93	-1.19	-0.66
Male	Laos	196.19	299.09	133.55	318.43	409.29	233.47	16.07	24.06	11.11	13.77	17.44	10.27	-0.64	-0.69	-0.58
Male	Latvia	138.39	157.22	122.63	176.20	218.36	139.14	9.85	11.09	8.79	12.85	15.84	10.16	0.91	0.40	1.42
Male	Lebanon	179.03	230.66	143.41	562.39	699.23	415.44	13.78	17.74	10.91	16.23	19.99	12.11	0.87	0.67	1.08
Male	Lesotho	46.49	61.62	35.61	71.50	98.29	49.42	8.83	11.75	6.78	11.83	16.17	8.47	1.53	1.26	1.80
Male	Liberia	45.36	57.04	34.17	71.16	96.98	50.42	6.72	8.48	5.08	5.99	8.12	4.27	-0.55	-0.75	-0.35
Male	Libya	95.76	124.22	73.07	267.31	344.54	198.69	6.98	8.97	5.31	8.66	11.05	6.60	1.07	0.88	1.26
Male	Lithuania	199.57	225.18	177.81	202.09	235.55	173.16	10.51	11.79	9.37	10.13	11.80	8.69	-1.18	-1.86	-0.50
Male	Luxembourg	58.06	65.16	51.84	76.99	95.90	61.59	24.60	27.64	22.02	17.77	22.06	14.31	-1.54	-1.76	-1.31
Male	Macedonia	112.42	130.46	97.37	125.81	152.44	102.28	11.38	13.13	9.94	8.07	9.75	6.60	-2.41	-3.02	-1.78
Male	Madagascar	453.26	562.32	355.76	980.80	1286.00	724.09	11.29	13.91	8.90	12.65	16.26	9.45	0.57	0.45	0.70
Male	Malawi	115.33	163.28	49.96	288.93	368.37	218.76	4.66	6.29	2.45	6.20	7.70	4.74	0.88	0.59	1.16
Male	Malaysia	871.69	1037.66	729.96	2196.32	2804.16	1754.54	15.18	17.98	12.76	15.38	19.72	12.41	-0.05	-0.17	0.07
Male	Maldives	25.08	41.90	16.07	50.13	60.87	41.18	42.69	68.47	28.76	30.73	37.66	25.19	-1.57	-1.74	-1.40
Male	Mali	248.67	301.31	203.66	490.99	661.33	334.25	9.58	11.57	7.95	8.74	11.58	5.87	-0.20	-0.38	-0.02
Male	Malta	21.77	24.27	19.51	41.98	48.15	36.56	11.10	12.34	9.96	11.65	13.27	10.22	0.16	-0.11	0.42
Male	Marshall Islands	1.79	2.50	1.24	4.15	5.89	2.77	16.42	23.32	11.42	20.31	28.15	13.95	0.79	0.57	1.01
Male	Mauritania	41.06	49.94	32.82	72.08	103.78	42.63	7.20	8.77	5.77	6.57	9.43	3.78	-0.35	-0.48	-0.22
Male	Mauritius	31.27	35.05	27.84	68.01	78.64	58.04	7.82	8.73	6.93	8.37	9.62	7.21	0.77	0.36	1.18
Male	Mexico	2632.73	2731.98	2540.29	7032.49	7456.02	6501.36	10.79	11.21	10.42	12.57	13.31	11.63	0.56	0.40	0.72

© 2020 Deng Y et al. *JAMA Network Open.*

Sex	Location	Disability adjusted life-years						Age-standardized DALY rate (per 100,000)						EAPC		
		1990(95%UI)			2017(95%UI)			1990(95%UI)			2017(95%UI)			1990-2017(95%CI)		
		DALYs (No.)	UL	LL	DALYs (No.)	UL	LL	95%UI	UL	LL	95%UI	UL	LL	EAPC	LL	UL
Male	Micronesia	3.97	5.25	3.03	6.17	8.45	4.16	13.29	17.49	10.20	15.56	20.64	10.98	0.64	0.48	0.80
Male	Moldova	240.94	281.42	206.49	291.67	338.61	251.57	11.64	13.51	10.03	12.10	13.91	10.45	0.39	-0.24	1.03
Male	Mongolia	61.86	73.13	51.44	151.45	188.68	118.87	10.78	12.67	8.97	12.09	15.05	9.65	0.41	0.13	0.70
Male	Montenegro	41.07	48.06	35.40	53.17	64.66	42.64	13.51	15.74	11.65	11.94	14.36	9.62	-0.68	-0.81	-0.56
Male	Morocco	1165.67	1421.32	945.42	2522.72	3305.92	1877.88	12.77	15.33	10.55	14.72	19.26	10.98	0.48	0.38	0.58
Male	Mozambique	562.50	754.44	405.23	1552.53	2186.41	1028.96	13.66	18.07	10.01	19.78	27.58	13.31	1.78	1.59	1.97
Male	Myanmar	2240.22	3449.90	1386.65	3285.64	4159.73	2636.54	16.92	25.85	10.71	15.27	19.03	12.51	-0.40	-0.44	-0.37
Male	Namibia	38.52	48.37	30.11	79.87	101.25	62.40	8.87	11.10	7.11	10.20	12.59	8.25	0.55	0.27	0.82
Male	Nepal	696.64	967.48	483.45	1367.83	1807.24	990.21	10.93	14.84	7.71	11.98	15.78	8.75	0.44	0.23	0.66
Male	Netherlands	788.18	863.07	718.34	1349.16	1560.92	1158.59	9.11	9.96	8.30	9.70	11.24	8.34	0.16	-0.12	0.44
Male	New Zealand	164.54	183.99	147.15	322.29	366.14	281.00	9.09	10.16	8.15	10.85	12.26	9.52	0.69	0.51	0.87
Male	Nicaragua	57.67	66.12	50.25	201.93	248.41	161.82	6.05	6.96	5.20	8.69	10.69	7.00	1.82	1.47	2.17
Male	Niger	121.68	158.68	83.43	232.64	312.51	169.06	6.36	8.19	4.32	5.37	7.17	3.87	-0.86	-1.01	-0.71
Male	Nigeria	1164.55	1695.05	788.81	2048.78	2908.83	1489.69	4.38	6.32	2.98	4.40	6.19	3.17	-0.20	-0.33	-0.07
Male	North Korea	732.88	981.51	526.85	1553.98	2182.96	1153.13	9.41	12.16	6.89	11.09	14.98	8.51	0.73	0.59	0.88
Male	Northern Mariana Islands	1.68	2.15	1.32	3.15	3.86	2.52	10.87	13.64	8.77	11.52	13.73	9.39	-0.02	-0.54	0.50
Male	Norway	284.59	299.55	269.88	385.65	438.31	348.21	10.42	10.99	9.89	9.86	11.25	8.90	0.08	-0.07	0.24
Male	Oman	50.55	70.60	37.05	197.94	264.57	140.55	8.64	11.67	6.41	10.18	13.41	7.29	0.94	0.61	1.27
Male	Pakistan	6898.77	8483.99	5493.97	17188.55	22789.54	12042.31	17.32	21.08	14.04	21.69	28.72	15.24	0.87	0.73	1.01
Male	Palestine	35.57	49.76	26.25	100.47	117.65	85.52	7.53	10.75	5.46	7.07	8.36	5.94	-0.28	-0.51	-0.05
Male	Panama	75.29	82.62	68.77	207.36	237.17	178.06	8.34	9.19	7.59	10.62	12.15	9.16	1.54	1.20	1.89
Male	Papua New Guinea	168.75	315.00	118.97	470.68	845.61	323.06	12.75	23.41	9.24	14.69	25.77	10.59	0.65	0.52	0.79
Male	Paraguay	128.03	151.56	105.28	433.57	558.58	327.44	10.76	12.65	8.92	15.71	20.11	11.83	1.51	1.02	1.99

© 2020 Deng Y et al. *JAMA Network Open.*

Sex	Location	Disability adjusted life-years						Age-standardized DALY rate (per 100,000)						EAPC		
		1990(95%UI)			2017(95%UI)			1990(95%UI)			2017(95%UI)			1990-2017(95%CI)		
		DALYs (No.)	UL	LL	DALYs (No.)	UL	LL	95%UI	UL	LL	95%UI	UL	LL	EAPC	LL	UL
Male	Peru	819.27	948.48	698.83	2057.02	2619.84	1570.04	11.59	13.46	9.83	13.51	17.21	10.34	0.77	0.58	0.97
Male	Philippines	3475.43	3972.07	3066.66	9698.94	11873.85	7753.14	18.69	21.33	16.49	25.30	30.87	20.48	1.29	1.06	1.52
Male	Poland	4059.22	4348.20	3779.01	2668.97	3079.95	2314.92	20.42	21.79	19.07	9.52	10.92	8.29	-3.50	-4.13	-2.86
Male	Portugal	744.82	814.15	670.25	951.24	1110.36	804.49	12.49	13.64	11.34	10.79	12.70	9.11	-0.44	-0.65	-0.22
Male	Puerto Rico	220.30	242.44	198.16	291.79	336.90	251.17	12.89	14.22	11.61	11.00	12.69	9.48	-0.38	-0.71	-0.04
Male	Qatar	11.38	15.26	8.59	81.10	105.71	60.44	10.02	12.82	7.88	7.25	9.30	5.46	-1.40	-2.05	-0.74
Male	Republic of Congo	55.61	77.20	40.92	115.71	162.89	81.30	9.13	12.44	6.96	7.84	10.76	5.53	-0.72	-0.86	-0.57
Male	Romania	1621.88	1774.90	1473.31	1559.00	1775.62	1378.71	12.21	13.29	11.12	10.68	12.13	9.45	-1.16	-1.56	-0.75
Male	Russia	7913.43	8868.84	7362.83	9864.03	10477.39	9293.55	10.98	12.20	10.23	11.13	11.84	10.49	-0.41	-0.89	0.07
Male	Rwanda	262.47	378.87	173.49	460.35	647.97	333.21	13.45	19.24	9.13	13.40	18.41	9.75	-0.26	-0.39	-0.12
Male	Saint Lucia	3.61	4.00	3.23	9.14	10.49	7.96	8.39	9.33	7.45	8.84	10.10	7.74	0.24	0.11	0.37
Male	Saint Vincent	3.17	3.53	2.82	9.55	11.02	8.16	9.01	10.12	7.97	13.78	15.83	11.85	1.69	1.56	1.82
Male	Samoa	8.48	10.75	6.63	10.63	14.51	7.01	15.76	19.87	12.25	14.02	18.46	9.50	-0.56	-0.63	-0.49
Male	Sao Tome and Principe	1.79	2.35	1.32	3.91	5.19	2.66	5.02	6.64	3.69	6.52	8.53	4.58	1.03	0.99	1.08
Male	Saudi Arabia	572.57	751.31	438.84	3076.74	4084.06	2207.20	11.22	14.67	8.49	20.16	25.30	15.40	3.15	2.74	3.56
Male	Senegal	138.36	165.38	113.47	277.47	364.78	211.31	7.13	8.50	5.85	7.12	9.37	5.44	-0.04	-0.17	0.09
Male	Serbia	580.52	712.10	467.72	729.22	864.57	605.47	10.26	12.49	8.42	10.83	12.90	8.95	0.70	0.55	0.85
Male	Seychelles	2.93	3.46	2.44	3.75	4.41	3.15	10.99	12.96	9.17	6.80	7.95	5.77	-2.04	-2.31	-1.76
Male	Sierra Leone	66.14	86.34	44.71	109.98	141.85	84.29	5.74	7.41	3.84	5.43	6.95	4.15	-0.30	-0.35	-0.25
Male	Singapore	122.98	134.87	112.09	217.79	258.62	182.39	9.69	10.66	8.82	6.60	7.81	5.57	-1.03	-1.24	-0.81
Male	Slovakia	347.76	395.82	301.95	323.75	383.12	265.33	13.02	14.78	11.34	8.44	9.93	6.92	-1.66	-1.81	-1.52
Male	Slovenia	127.03	141.94	114.94	166.80	193.13	142.90	12.10	13.39	11.01	9.81	11.24	8.43	-0.82	-0.97	-0.67
Male	Solomon Islands	11.91	18.91	8.61	28.28	40.34	20.52	11.93	18.77	8.63	13.49	19.11	10.00	0.55	0.38	0.72

© 2020 Deng Y et al. *JAMA Network Open.*

Sex	Location	Disability adjusted life-years						Age-standardized DALY rate (per 100,000)						EAPC		
		1990(95%UI)			2017(95%UI)			1990(95%UI)			2017(95%UI)			1990-2017(95%CI)		
		DALYs (No.)	UL	LL	DALYs (No.)	UL	LL	95%UI	UL	LL	95%UI	UL	LL	EAPC	LL	UL
Male	Somalia	236.42	433.42	87.99	816.65	1170.93	551.31	12.30	21.09	5.93	17.61	24.52	12.02	1.18	1.03	1.34
Male	South Africa	736.58	856.52	641.08	1359.06	1535.36	1167.96	6.46	7.71	5.49	6.46	7.28	5.55	-0.29	-0.85	0.27
Male	South Korea	1282.39	1390.80	1151.63	8272.35	10071.01	6924.92	8.50	9.23	7.64	21.04	25.64	17.64	5.52	4.50	6.55
Male	South Sudan	213.34	393.17	94.55	465.55	710.17	312.94	11.69	20.46	6.00	16.74	25.47	11.36	1.27	1.11	1.43
Male	Spain	2501.40	2690.83	2307.18	3823.83	4475.12	3293.58	10.72	11.54	9.90	10.23	12.03	8.73	-0.06	-0.14	0.03
Male	Sri Lanka	901.10	1053.32	775.18	1334.60	1768.42	973.65	14.62	17.18	12.52	11.44	15.00	8.41	-1.41	-2.04	-0.78
Male	Sudan	493.70	815.81	314.17	1037.52	1384.71	741.44	8.47	13.78	5.49	9.02	12.12	6.40	0.38	0.31	0.45
Male	Suriname	8.57	9.74	7.46	26.30	31.69	21.67	5.99	6.81	5.23	8.98	10.79	7.44	1.64	1.43	1.85
Male	Swaziland	17.70	22.02	14.10	42.39	59.42	29.20	10.17	12.74	8.12	13.69	19.13	9.44	1.60	1.17	2.03
Male	Sweden	551.04	597.63	505.24	699.16	789.02	614.39	9.05	9.80	8.28	8.62	9.72	7.59	0.32	0.07	0.58
Male	Switzerland	463.88	525.08	414.36	692.74	835.63	578.82	10.76	12.19	9.67	9.87	11.93	8.26	0.16	-0.03	0.35
Male	Syria	158.30	213.64	124.33	390.40	509.62	294.43	4.76	6.53	3.71	5.18	6.74	3.90	-0.04	-0.23	0.14
Male	Tajikistan	59.64	69.49	50.91	130.10	156.16	108.15	4.09	4.82	3.42	4.46	5.34	3.72	-0.05	-0.35	0.26
Male	Tanzania	739.37	1045.56	413.41	2060.15	2825.86	1505.69	10.36	14.33	6.55	13.45	18.33	9.76	0.86	0.76	0.97
Male	Thailand	2643.04	3031.41	2284.69	4110.34	4954.56	3420.87	13.17	15.26	11.39	9.13	10.92	7.61	-2.09	-2.40	-1.78
Male	Timor-Leste	23.75	31.51	15.96	58.73	86.51	36.42	11.68	15.31	7.66	13.64	19.98	8.60	0.65	0.50	0.80
Male	Tobago	45.16	49.96	41.06	88.83	114.78	66.88	10.15	11.25	9.19	10.23	13.15	7.75	-0.22	-0.66	0.23
Male	Togo	49.79	63.42	37.96	114.82	148.88	85.57	6.31	7.89	4.83	6.22	7.95	4.69	-0.16	-0.28	-0.05
Male	Tonga	3.09	3.82	2.46	5.95	7.92	4.32	10.15	12.53	8.09	14.62	19.56	10.63	1.55	1.35	1.74
Male	Trinidad	45.16	49.96	41.06	88.83	114.78	66.88	10.15	11.25	9.19	10.23	13.15	7.75	-0.22	-0.66	0.23
Male	Tunisia	255.08	304.56	206.66	557.36	745.95	403.79	8.23	9.86	6.63	9.19	12.20	6.68	0.18	0.02	0.34
Male	Turkey	2219.75	3296.06	1590.81	4398.63	5159.80	3705.15	11.18	16.54	8.05	10.44	12.23	8.81	-0.09	-0.35	0.16
Male	Turkmenistan	113.34	129.15	99.35	184.93	216.44	156.89	10.30	11.73	9.00	8.59	9.97	7.35	-0.72	-1.02	-0.42
Male	Uganda	385.10	517.95	272.50	2033.98	2652.63	1519.95	8.56	11.43	6.29	21.43	27.48	16.39	3.76	3.42	4.11

© 2020 Deng Y et al. *JAMA Network Open.*

Sex	Location	Disability adjusted life-years						Age-standardized DALY rate (per 100,000)						EAPC		
		1990(95%UI)			2017(95%UI)			1990(95%UI)			2017(95%UI)			1990-2017(95%CI)		
		DALYs (No.)	UL	LL	DALYs (No.)	UL	LL	95%UI	UL	LL	95%UI	UL	LL	EAPC	LL	UL
Male	UK	3141.59	3372.17	2920.86	3863.20	4139.13	3620.92	8.49	9.10	7.93	7.69	8.26	7.19	-0.21	-0.34	-0.07
Male	Ukraine	3035.36	4048.60	2457.53	4366.92	5035.64	3834.91	10.72	14.10	8.81	15.57	18.03	13.67	0.85	0.43	1.27
Male	United Arab Emirates	67.76	92.72	49.35	760.62	1191.23	465.69	10.06	13.75	6.70	12.80	21.43	7.40	1.02	0.93	1.12
Male	Uruguay	126.42	143.37	112.51	251.26	296.98	208.59	7.40	8.33	6.58	12.11	14.35	10.04	1.54	1.04	2.05
Male	USA	13059.12	13959.81	12285.95	26525.70	28835.68	24344.92	9.69	10.37	9.11	11.42	12.46	10.48	0.40	0.26	0.53
Male	Uzbekistan	209.74	275.65	175.19	566.03	669.29	474.09	3.70	5.04	3.02	5.28	6.16	4.46	1.31	1.18	1.44
Male	Vanuatu	7.21	11.46	4.00	21.39	34.82	11.96	15.53	24.44	8.61	20.82	33.97	11.60	1.27	1.13	1.41
Male	Venezuela	414.27	459.41	374.97	1554.56	1941.33	1207.10	7.67	8.52	6.92	10.98	13.73	8.55	1.00	0.76	1.24
Male	Vietnam	3907.74	4808.28	3191.69	12631.52	15855.43	9958.51	18.34	22.24	14.97	27.61	33.91	22.40	2.21	1.88	2.53
Male	Virgin Islands	4.89	5.75	4.17	12.33	15.10	9.46	10.87	12.72	9.43	15.38	18.53	12.11	1.80	1.64	1.95
Male	Yemen	274.25	540.10	136.15	746.45	1056.69	541.62	8.38	15.95	4.68	9.45	13.18	6.80	0.49	0.44	0.54
Male	Zambia	287.74	391.59	180.92	722.56	953.87	564.44	13.33	17.90	9.24	15.15	19.65	11.98	0.18	0.00	0.36
Male	Zimbabwe	316.95	385.07	258.19	786.73	967.00	618.16	12.10	14.67	9.81	18.73	23.12	15.11	1.74	0.89	2.60

Abbreviations: DALY, disability adjusted life-year; CI, confidence interval; EAPC, estimated annual percentage change; UI, uncertainty interval; LL, lower limit; UL, upper limit.

© 2020 Deng Y et al. *JAMA Network Open.*

eTable 6. The EAPC of thyroid cancer among SDI quintiles and 21 regions.

Location	Sex	Incidence (95%CI)			Death (95%CI)			DALYs (95%CI)		
		EAPCs	LL	UL	EAPCs	LL	UL	EAPCs	LL	UL
**Socio-demographic index**										
High SDI	Both	1.61	1.33	1.89	-0.68	-0.74	-0.62	-0.36	-0.48	-0.24
High SDI	Female	1.51	1.19	1.82	-1.03	-1.09	-0.97	-0.59	-0.73	-0.45
High SDI	Male	1.94	1.71	2.16	-0.09	-0.18	-0.01	0.03	-0.09	0.15
High-middle SDI	Both	1.81	1.64	1.98	-0.48	-0.58	-0.39	-0.56	-0.68	-0.43
High-middle SDI	Female	1.49	1.33	1.64	-1.18	-1.30	-1.07	-1.19	-1.32	-1.05
High-middle SDI	Male	2.89	2.63	3.14	0.72	0.56	0.87	0.44	0.30	0.58
Middle SDI	Both	2.65	2.54	2.75	0.33	0.25	0.41	0.25	0.16	0.33
Middle SDI	Female	2.28	2.21	2.36	-0.40	-0.44	-0.36	-0.44	-0.50	-0.37
Middle SDI	Male	3.57	3.35	3.79	1.51	1.35	1.67	1.30	1.16	1.44
Low-middle SDI	Both	1.91	1.84	1.98	0.28	0.23	0.33	0.23	0.15	0.30
Low-middle SDI	Female	1.75	1.68	1.82	-0.18	-0.26	-0.10	-0.14	-0.24	-0.03
Low-middle SDI	Male	2.25	2.12	2.37	1.00	0.93	1.07	0.84	0.77	0.91
Low SDI	Both	1.01	0.88	1.13	-0.23	-0.28	-0.17	-0.54	-0.58	-0.50
Low SDI	Female	0.83	0.68	0.98	-0.66	-0.76	-0.56	-0.98	-1.07	-0.89
Low SDI	Male	1.30	1.20	1.39	0.34	0.27	0.40	0.11	0.05	0.18
**Region**										
Central Sub-Saharan Africa	Both	0.02	-0.14	0.18	-0.56	-0.63	-0.49	-0.71	-0.79	-0.64
Central Sub-Saharan Africa	Female	-0.11	-0.28	0.07	-0.85	-0.93	-0.78	-0.98	-1.06	-0.91
Central Sub-Saharan Africa	Male	0.31	0.14	0.48	-0.23	-0.33	-0.12	-0.30	-0.40	-0.20
Eastern Sub-Saharan Africa	Both	-0.01	-0.15	0.14	-0.95	-1.05	-0.86	-1.30	-1.42	-1.17
Eastern Sub-Saharan Africa	Female	-0.41	-0.58	-0.24	-1.76	-1.89	-1.63	-2.04	-2.20	-1.88

© 2020 Deng Y et al. *JAMA Network Open.*

Location	Sex	Incidence (95%CI)			Death (95%CI)			DALYs (95%CI)		
		EAPCs	LL	UL	EAPCs	LL	UL	EAPCs	LL	UL
Eastern Sub-Saharan Africa	Male	0.92	0.80	1.03	0.15	0.08	0.22	-0.19	-0.28	-0.10
Southern Sub-Saharan Africa	Both	-0.10	-0.58	0.39	0.09	-0.43	0.60	-0.10	-0.71	0.51
Southern Sub-Saharan Africa	Female	-0.36	-0.86	0.13	-0.09	-0.61	0.43	-0.33	-0.94	0.30
Southern Sub-Saharan Africa	Male	0.54	0.11	0.96	0.28	-0.20	0.77	0.21	-0.38	0.81
Western Sub-Saharan Africa	Both	0.39	0.30	0.47	-0.55	-0.62	-0.48	-0.70	-0.79	-0.62
Western Sub-Saharan Africa	Female	0.08	0.02	0.15	-1.05	-1.12	-0.97	-1.25	-1.34	-1.17
Western Sub-Saharan Africa	Male	0.79	0.69	0.89	0.13	0.06	0.19	0.00	-0.08	0.09
Oceania	Both	0.73	0.57	0.90	-0.02	-0.05	0.02	-0.02	-0.09	0.05
Oceania	Female	0.79	0.67	0.91	-0.13	-0.19	-0.07	-0.16	-0.19	-0.13
Oceania	Male	0.55	0.27	0.84	0.18	0.00	0.36	0.23	0.04	0.41
North Africa and Middle East	Both	2.98	2.86	3.11	-0.15	-0.23	-0.08	0.06	-0.01	0.13
North Africa and Middle East	Female	2.86	2.75	2.98	-0.68	-0.74	-0.62	-0.32	-0.38	-0.26
North Africa and Middle East	Male	3.54	3.35	3.73	0.62	0.52	0.73	0.66	0.56	0.75
Andean Latin America	Both	3.69	3.29	4.09	1.08	0.85	1.31	0.95	0.71	1.20
Andean Latin America	Female	3.72	3.32	4.12	0.99	0.78	1.19	0.85	0.64	1.06
Andean Latin America	Male	3.60	3.18	4.03	1.30	0.97	1.62	1.17	0.84	1.50
Central Latin America	Both	1.93	1.81	2.05	-0.35	-0.51	-0.20	-0.27	-0.43	-0.11
Central Latin America	Female	1.88	1.75	2.01	-0.54	-0.70	-0.39	-0.43	-0.59	-0.26
Central Latin America	Male	1.88	1.75	2.01	-0.08	-0.23	0.08	-0.06	-0.22	0.11
High-income North America	Both	1.32	1.06	1.57	0.29	0.20	0.38	0.37	0.25	0.50
High-income North America	Female	1.38	1.10	1.65	0.17	0.07	0.27	0.38	0.23	0.52
High-income North America	Male	1.24	0.99	1.49	0.47	0.35	0.58	0.39	0.26	0.52
Southern Latin America	Both	0.85	0.66	1.04	-1.12	-1.30	-0.93	-1.13	-1.34	-0.91
Southern Latin America	Female	0.78	0.57	0.99	-1.32	-1.54	-1.10	-1.26	-1.51	-1.01
Southern Latin America	Male	1.09	0.93	1.25	-0.78	-0.94	-0.62	-0.89	-1.07	-0.71
Tropical Latin America	Both	1.19	1.00	1.38	-0.68	-0.75	-0.62	-0.64	-0.73	-0.55

© 2020 Deng Y et al. *JAMA Network Open.*

Location	Sex	Incidence (95%CI)			Death (95%CI)			DALYs (95%CI)		
		EAPCs	LL	UL	EAPCs	LL	UL	EAPCs	LL	UL
Tropical Latin America	Female	0.91	0.71	1.11	-1.24	-1.31	-1.17	-1.17	-1.26	-1.07
Tropical Latin America	Male	1.85	1.67	2.02	0.15	0.04	0.25	0.10	-0.01	0.21
Central Europe	Both	0.40	0.27	0.54	-2.15	-2.35	-1.96	-2.03	-2.24	-1.83
Central Europe	Female	0.55	0.44	0.67	-2.20	-2.36	-2.04	-1.96	-2.12	-1.80
Central Europe	Male	0.11	-0.14	0.37	-2.08	-2.38	-1.79	-2.10	-2.38	-1.82
Eastern Europe	Both	2.19	1.88	2.51	-0.23	-0.63	0.17	0.14	-0.25	0.54
Eastern Europe	Female	2.39	2.04	2.73	-0.16	-0.56	0.25	0.32	-0.08	0.72
Eastern Europe	Male	1.81	1.53	2.08	-0.28	-0.68	0.12	-0.03	-0.44	0.38
Western Europe	Both	0.58	0.36	0.80	-1.45	-1.49	-1.41	-1.12	-1.21	-1.04
Western Europe	Female	0.14	-0.05	0.33	-1.99	-2.10	-1.88	-1.60	-1.66	-1.54
Western Europe	Male	1.58	1.26	1.90	-0.53	-0.65	-0.41	-0.42	-0.62	-0.22
Australasia	Both	2.87	2.68	3.05	0.71	0.51	0.91	1.03	0.85	1.21
Australasia	Female	2.88	2.66	3.10	0.54	0.31	0.77	0.93	0.73	1.13
Australasia	Male	2.85	2.70	2.99	0.98	0.81	1.15	1.19	1.02	1.35
Caribbean	Both	1.53	1.30	1.77	0.10	-0.10	0.30	0.17	-0.04	0.38
Caribbean	Female	1.26	0.98	1.54	-0.32	-0.59	-0.05	-0.20	-0.48	0.08
Caribbean	Male	2.29	2.17	2.42	0.74	0.63	0.84	0.76	0.64	0.88
Central Asia	Both	-0.11	-0.56	0.34	-1.11	-1.40	-0.83	-1.59	-1.88	-1.30
Central Asia	Female	-0.05	-0.49	0.39	-1.11	-1.35	-0.87	-1.54	-1.80	-1.27
Central Asia	Male	-0.28	-0.79	0.23	-1.07	-1.46	-0.68	-1.66	-2.01	-1.31
East Asia	Both	2.66	2.31	3.01	0.23	-0.04	0.50	-0.26	-0.57	0.05
East Asia	Female	1.52	1.20	1.85	-1.62	-1.83	-1.41	-2.03	-2.35	-1.72
East Asia	Male	5.01	4.56	5.47	2.32	1.94	2.70	1.79	1.44	2.13
South Asia	Both	2.30	2.16	2.43	0.56	0.48	0.64	0.46	0.37	0.55
South Asia	Female	2.15	2.00	2.30	0.18	0.08	0.27	0.13	0.02	0.23
South Asia	Male	2.50	2.35	2.65	1.07	0.97	1.17	0.97	0.86	1.08

© 2020 Deng Y et al. *JAMA Network Open.*

Location	Sex	Incidence (95%CI)			Death (95%CI)			DALYs (95%CI)		
		EAPCs	LL	UL	EAPCs	LL	UL	EAPCs	LL	UL
Southeast Asia	Both	2.00	1.95	2.04	-0.16	-0.24	-0.08	-0.27	-0.33	-0.21
Southeast Asia	Female	1.98	1.93	2.03	-0.56	-0.64	-0.48	-0.64	-0.70	-0.58
Southeast Asia	Male	2.18	2.13	2.23	0.61	0.52	0.69	0.47	0.40	0.54
High-income Asia Pacific	Both	4.43	3.69	5.18	0.29	0.01	0.56	1.45	0.99	1.91
High-income Asia Pacific	Female	4.45	3.64	5.26	-0.03	-0.34	0.27	1.36	0.83	1.90
High-income Asia Pacific	Male	4.79	4.19	5.39	0.90	0.64	1.17	1.79	1.40	2.19

Abbreviations: DALY, disability adjusted life-year; CI, confidence interval; EAPC, estimated annual percentage change; UI, uncertainty interval; LL, lower limit; UL, upper limit.

© 2020 Deng Y et al. *JAMA Network Open.*
